# Illuminating Dark Chemical Matter Using the Cell Painting Assay

**DOI:** 10.1021/acs.jmedchem.4c00160

**Published:** 2024-04-30

**Authors:** Axel Pahl, Jie Liu, Sohan Patil, Soheila Rezaei Adariani, Beate Schölermann, Jens Warmers, Jana Bonowski, Sandra Koska, Yasemin Akbulut, Carina Seitz, Sonja Sievers, Slava Ziegler, Herbert Waldmann

**Affiliations:** † Max-Planck Institute of Molecular Physiology, Department of Chemical Biology, Otto-Hahn-Strasse 11, Dortmund 44227, Germany; ‡ Technical University Dortmund, Faculty of Chemistry and Chemical Biology, Otto-Hahn-Strasse 6, Dortmund 44227, Germany

## Abstract

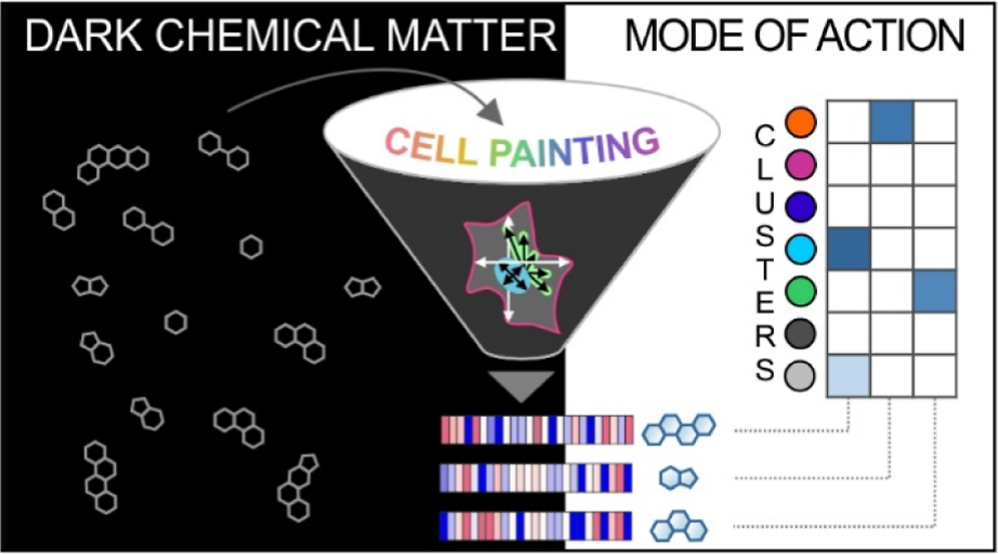

Screening for small-molecule modulators of disease-relevant targets and phenotypes is the first step on the way to new drugs. Large compound libraries have been synthesized by academia and, particularly, pharmaceutical companies to meet the need for novel chemical entities that are as diverse as possible. Screening of these compound libraries revealed a portion of small molecules that is inactive in more than 100 different assays and was therefore termed “dark chemical matter” (DCM). Deorphanization of DCM promises to yield very selective compounds as they are expected to have less off-target effects. We employed morphological profiling using the Cell Painting assay to detect bioactive DCM. Within the DCM collection, we identified bioactive compounds and confirmed several modulators of microtubules, DNA synthesis, and pyrimidine biosynthesis. Profiling approaches are, therefore, powerful tools to probe compound collections for bioactivity in an unbiased manner and are particularly suitable for deorphanization of DCM.

## Introduction

Screening of large compound collections using target- or phenotype-based assays frequently is the initial step in the discovery of biologically active small molecules. Typically, 0.1–1% of these compounds score as hits in such screens, and higher hit rates are undesirable as they are often linked to the detection of promiscuous compounds (frequent hitters), e.g., polypharmacological or pan-assay interference compounds (PAINS).^1,2^ This raises the question of what are the right small molecules to synthesize with regard to bioactivity. Different organic synthesis design principles proved powerful for the identification of small-molecule modulators of biological targets, like biology-oriented synthesis (BIOS),^3,4^ diversity-oriented synthesis (DOS),^5^ or the pseudonatural product (PNP) concept.^6,7^ Generally, however, screening collections are often compiled based on chemical diversity and attractiveness, considering physicochemical and/or drug-like properties.^8^ Analysis of the biological profiles of compound collections at Novartis, i.e., one of the leading pharmaceutical companies, identified a set of compounds from the Novartis and NIH Molecular Libraries Program screening collections that were consistently inactive in at least 100 biological assays.^8^ This set of compounds was termed “dark chemical matter” (DCM), a term initially coined by Pope.^9^ Interestingly, DCM does not comprise separate scaffold classes that have been found to be inactive in all these assays. In contrast, DCM compounds are often close analogues of biologically active small molecules, have drug-like physicochemical properties, tend to have higher aqueous solubility, and are less hydrophobic than active compounds.^8^ Hence, DCM is endowed with drug-like features for modulation of biological targets. Among the possible reasons, the lack of bioactivity may be the focus on a narrow drug target space, mostly considering mammalian targets and inappropriate screening concentrations.^8^ Conducting an increasing number of focused screening assays could identify some active DCM,^10^ however, these assays are biased toward the target or phenotype of interest. Profiling approaches, in contrast, can capture the modulation of multiple targets simultaneously and identify perturbed targets in a less biased manner. In fact, Wassermann et al. detected changes in the expression of 61 selected genes by DCM^8^, indicating that, indeed, DCM does not represent completely biologically inert compounds. Therefore, unbiased profiling appears to be particularly suitable for the analysis of DCM.

Here, we report on the use of morphological profiling by means of the Cell Painting assay (CPA) for the deorphanization of DCM. A set of 7677 DCM compounds was profiled using CPA and suggested modes of action for various active small molecules. In particular, we identified inhibitors of microtubule dynamics, cell cycle, or de novo pyrimidine synthesis, thus confirming that DCM may not be biologically inert. Hence, morphological profiling can indeed be used for the deorphanization of DCM.

## Results and Discussion

We employed CPA to profile a subset of DCM compounds (see below). CPA is a morphological profiling approach,^11,12^ in which cells are exposed to perturbagens prior to staining with different dyes for visualization of different cell components and compartments, i.e., DNA, RNA and nucleoli, endoplasmic reticulum, actin cytoskeleton, Golgi, plasma membrane, and mitochondria. High-content imaging and analysis lead to the extraction of hundreds of morphological features using CellProfiler (https://cellprofiler.org/^13^). The morphological feature profiles are normalized to DMSO controls, and feature values are calculated as *Z*-scores. In our setup, CPA profiles are composed of 579 features.^14^ The percentage of significantly changed features is defined as induction and used as a measure of bioactivity (compounds with induction ≥5% are considered to be active). Profiles sharing a similarity (also termed biosimilarity) of ≥75% are considered similar. By comparing the profiles of active compounds to those of reference compounds with annotated activities, target hypotheses can be derived (details in the “Experimental Section” section). In addition, a similarity to 13 biological clusters thus far can be determined by comparing CP subprofiles.^15,16^

**Figure 1:**
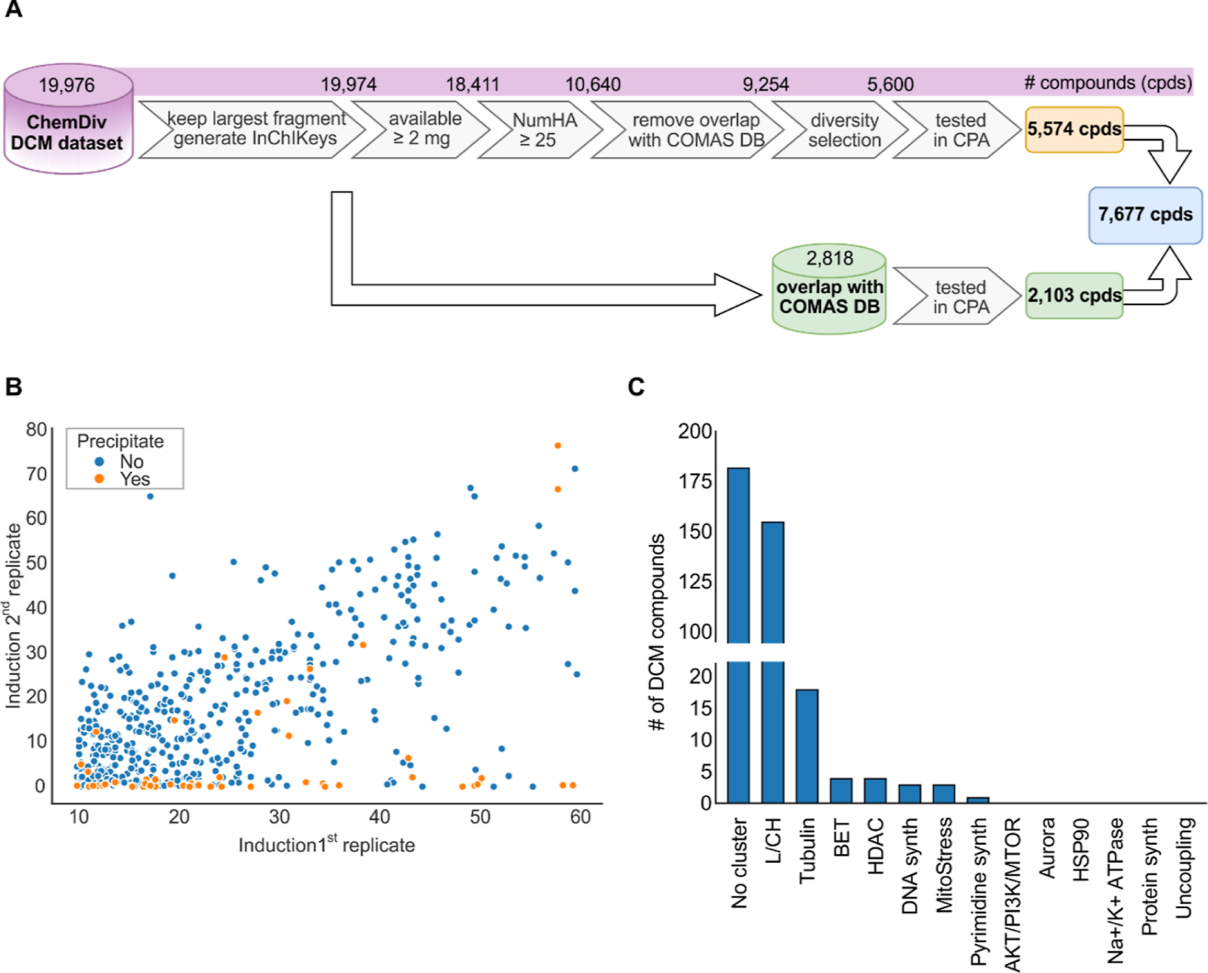
Selection of DCM compounds for the morphological profiling. (A) Summary of the DCM selection process. See also Figure S1. (B) Reproducibility of the induced changes in CPA as determined by the induction value of two replicates for 549 DCM compounds that were initially identified as active in CPA. Correlation between the replicates: *r*^2^ = 0.0246 (Pearson correlation 0.593). (C) Subprofile analysis. Similarities for the 370 CPA-active DCM compounds were found for the 13 defined bioactivity clusters. Subprofile biosimilarity ≥80% is considered. Synth: synthesis. See also Figures S1–S3.

ChemDiv offers a DCM library^17^ that represents the overlap of the 140,000 DCM compounds published by Wassermann et al. and the ChemDiv compound stock. At the time of this investigation, the DCM library contained 19,976 compounds (currently, more than 25,000 compounds). The characteristics of the library using descriptors like molecular weight, log *P*, topological polar surface area (TPSA), fraction of sp^3^-hybridized carbons (FrCsp3), number of hydrogen acceptors (NumHAcc), and number of hydrogen donors (NumHDon) are shown in Figure S1. Using Python and cheminformatics toolkit RDKit (https://www.rdkit.org), the set of 19,976 compounds of the ChemDiv DCM library was standardized for the largest fragment using the provided SD file, and InChIKeys were added. Two InChIKeys could not be generated (leaving 19,974 compounds). In addition to the structure, the original file contained an identifier and the available amount in milligram (“available”). The structural overlap of the full ChemDiv data set with our internal compound database (“COMAS DB”, 194,832 compounds) was determined by InChIKey to be 2818 compounds (Figure 1A). The full ChemDiv DCM data set was filtered for compounds with sufficient availability (“available” ≥ 2 mg), leaving 18,411 compounds. The number of heavy atoms (“NumHA”) and molecular weight (“MW”) were calculated, and smaller structures with less than 25 NumHA, which are known to have a low probability of activity in the CPA,^18^ were removed, leaving 10,640 compounds. The structures overlapping with the COMAS DB were removed, leaving 9254 entries. Using RDKit, a diversity selection based on Morgan fingerprints was performed (Figure S2), and 5600 compounds were ordered. 5574 of those were tested together with the available overlapping internal compounds (2103) in the CPA. In total, 7677 compounds were subjected to the morphological profiling at a concentration of 10 μM (Figures 1A and S1 for the physicochemical characterization of the selection).

Of the 7677 tested compounds, 942 (12%) showed a significant morphological change compared to the DMSO controls (induction ≥5%). For comparison, we detected CPA activity for 34% of all tested reference compounds (1463 of 4307) and 31% (3983 out of 12,951) for our in-house compound collection, which include uncharacterized small molecules, e.g., natural product-inspired compounds^19,20^ and pseudonatural products.^7,21,22^ 549 compounds with induction ≥10% were tested again in CPA. Surprisingly, the activity of only 370 of the initial active compounds was confirmed. This rather low reproducibility is in stark contrast with the CP data with our in-house compounds. When comparing the induction values between two biological replicates, the correlation for our internal compounds was *r*^2^ = 0.617 (Pearson correlation 0.812), whereas for DCM, *r*^2^ of 0.0246 (Pearson correlation 0.593) was obtained (see Figures 1B and S3A and S3B). This difference was even more striking when analyzing the profile biosimilarity of the biological replicates: the median biosimilarity was 87% for the internal data set and 78% for DCM. Within our internal CP data, profile biosimilarity of the replicates was ≥80% for 78% of the compounds, while only 43% of the DCM set displayed profile similarity of the replicates ≥80% (see Figure S3C). By inspection of compound solutions using Tube Auditor, we noticed precipitates for a substantial proportion (9.6%) of the initially active DCM compounds that most likely account for the low reproducibility in CPA (Figures 1B and S3A). For comparison, precipitation affected only 0.4% of our internal compounds. Of note, freshly dissolved DCM compounds apparently did not show precipitates, whereas precipitation occurred after a freeze–thaw cycle. Therefore, the inactivity of at least some of the DCM compounds may be attributed to the low DMSO solubility.

The CPA profiles of the 370 confirmed compounds were submitted to a cluster subprofile analysis to map similarity to modulation of AKT/PI3K/MTOR, Aurora kinases, BET, DNA synthesis, HDAC, HSP90, lysosomotropism/cholesterol homeostasis (L/CH), mitochondrial stress (MitoStress), Na^+^/K^+^ ATPase, protein synthesis, pyrimidine synthesis, tubulin, or uncoupling of the mitochondrial proton gradient.^15,16^ For this, bioactivity clusters were defined based on profile similarity of mostly annotated compounds. Common features of the defining profiles were extracted to construct a median profile for each cluster, i.e., cluster subprofile which has a reduced number of features as compared to the full profile.^15^ Importantly, we consider subprofiles to be biosimilar for biosimilarity ≥80% as subprofiles contain less features than the full profiles.^15^ Most of the CPA-active DCM compounds were similar to the lysosomotropism/cholesterol homeostasis cluster (in total, 155 small molecules after the second biological replicate). Moreover, the profiles of several compounds were biosimilar to the clusters of tubulin, DNA synthesis, pyrimidine synthesis, MitoStress, or BET modulators (Figure 1C, see also Table S1). The L/CH cluster compiles small molecules that impair cholesterol homeostasis by either specific target modulation or due to their lysosomotropic activity.^23^ Lysosomotropic compounds are weakly basic, lipophilic molecules that share similar physicochemical properties, i.e., logP >2 and p*K*_a_ between 6.5 and 11.^24^ Indeed, 67% of DCM that is similar to L/CH shares properties of lysosomotropic compounds (Figure S4).

**Figure 2:**
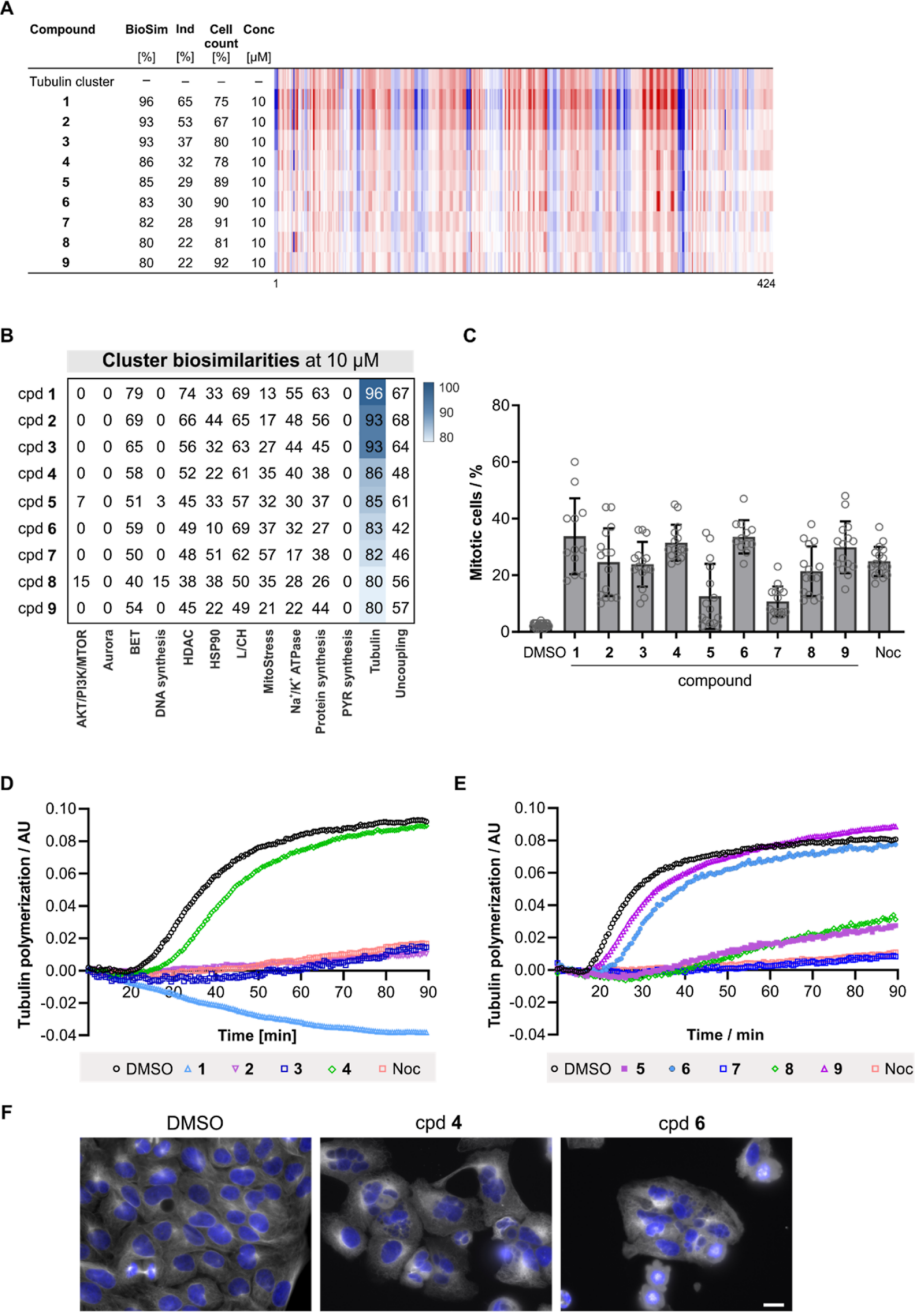
Identification of DCM targeting tubulin. (A) Similarity of compound (cpd) **1**–**9** to the tubulin cluster subprofile. The top line profile is set as a reference profile (100% biological similarity, BioSim) to which the following profiles are compared. Blue color: decreased feature and red color: increased feature. BioSim: biosimilarity; Ind: induction; and conc: concentration. (B) Cluster biosimilarity heatmap for compounds **1**–**9**. PYR: pyrimidine. (C) Influence of compounds **1**–**9** on the number of mitotic cells. U2OS cells were treated with the compounds (30 μM) or 0.1 μM nocodazole (Noc) for 24 h prior to detection of mitotic cells using antiphospho-histone H3. Data are mean values ±SD of all technical and biological replicates (*N* = 3, *n* ≥ 4). (D and E) Influence of the compounds on the in vitro tubulin polymerization at 20 μM. Nocodazole was used as a control (2 μM). Data representative of *n* = 3. (F) Influence of microtubules in cells. U2OS cells were treated with 30 μM of cpd **4** or cpd **6** or DMSO as a controls for 24 h prior to staining with antitubulin antibody (white) or DAPI (blue) to visualize the DNA. Scale bar: 20 μm. See also Figure S5.

Several DCM compounds displayed similarity to the tubulin cluster (Table 1 and Figure 2A and B). To confirm the target hypothesis, the influence of the compounds on mitosis was explored, as impairment of microtubule dynamics is linked to mitotic arrest. Indeed, the compounds increased the percentage of metaphase cells which were detected using phospho-histone H3 as a marker (Figure 2C). Moreover, most compounds almost completely suppressed in vitro tubulin polymerization (Figure 2D and E) and disturbed the microtubule cytoskeleton in cells (Figures 2F and S5). Of note, target prediction based on chemical similarity using the Similarity Ensemble Approach (SEA, https://sea.bkslab.org)^25^ revealed tubulin as a potential target for compounds **1**, **2**, and **3**. However, for compounds **1** and **3**, tubulin was not among the top 20 predictions. Only for compound **2**, tubulin was ranked among the top 5 predicted targets and would have been considered for validation experiments. The tubulin-targeting chemical space is very broad, and often only minor structural modifications turn a compound into a tubulin modulator^26^ which hampers target prediction based on chemical similarity. These results demonstrate that prediction based on similar morphological profiles and particularly using cluster biosimilarity reliably identifies microtubule targeting agents in compound collections.

**Table 1:**
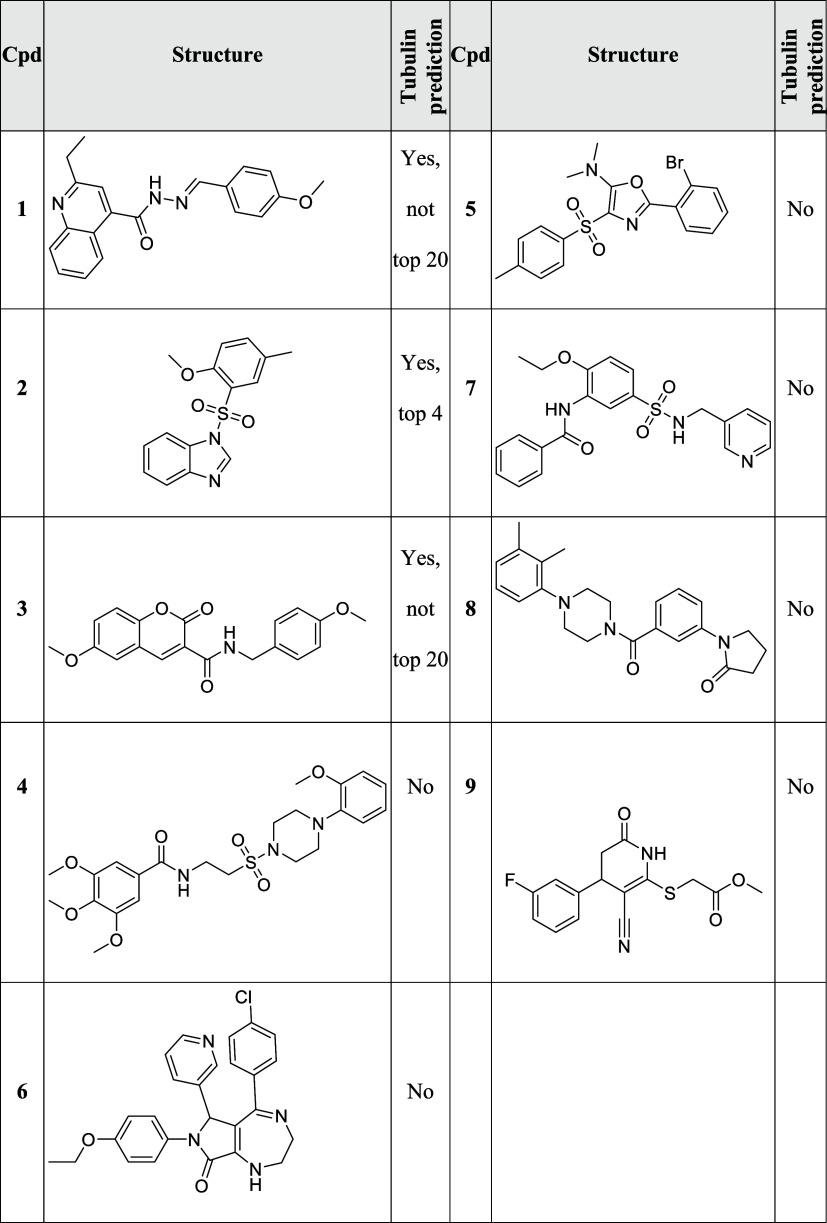
Prediction of Tubulin as a Target for DCM Compounds (cpds) **1–9**^a^

a The tool SEA (Similarity Ensemble Approach)^25^ was used to detect tubulin as a potential target.

**Figure 3:**
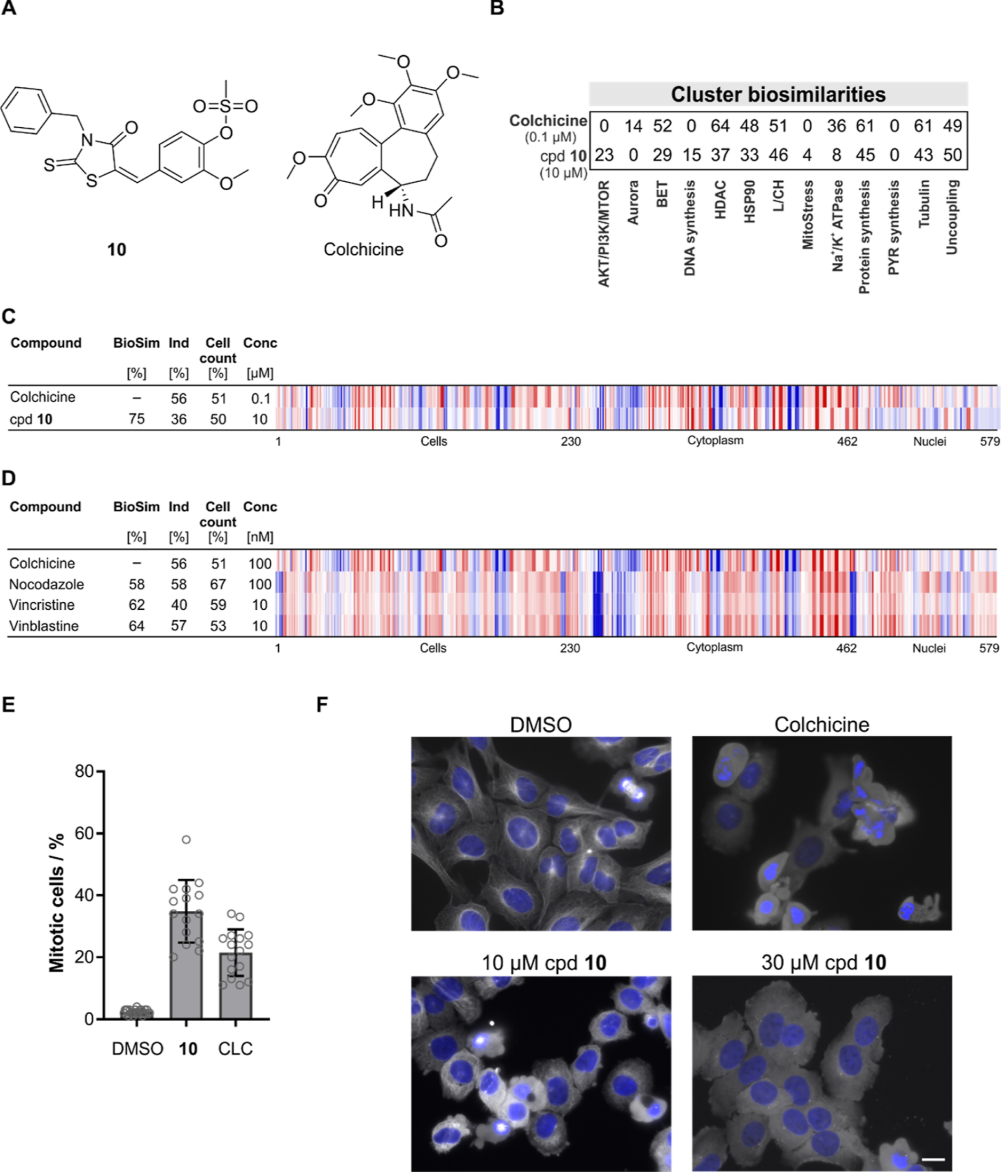
Compound **10** is a microtubule depolymerizer. (A) Structures of compound (cpd) **10** and colchicine. (B) Cluster biosimilarity heatmap for colchicine and compound **10**. PYR: pyrimidine. (C) Profile similarity of compound **10** to colchicine. (D) Profile similarity of colchicine to nocodazole, vincristine, and vinblastine. (C,D) Top line profile is set as a reference profile (100% biological similarity, BioSim) to which the following profiles are compared. Blue color: decreased feature and red color: increased feature. BioSim: biosimilarity; Ind: induction; and Conc: concentration. (E) Influence of compound **10** on the number of mitotic cells. U2OS cells were treated with **10** (10 μM) for 24 h prior to detection of mitotic cells using antiphospho-histone H3. DMSO and colchicine (CLC, 0.01 μM) were used as controls. Data are mean values ±SD (*N* = 3, *n* > 4). (F) Influence on microtubules in cells. U2OS cells were treated with 10 or 30 μM **10** or DMSO and colchicine (0.01 μM) as controls for 24 h prior to staining with antitubulin antibody (white) or DAPI (blue) to visualize the DNA. Scale bar: 20 μm. See also Figure S6.

We noticed a profile similarity between compound **10** and microtubule destabilizer colchicine (Figure 3A−C). Although affecting microtubule dynamics, the CPA profile of colchicine (100 nM) is not similar to the profiles recorded for prototypic tubulin inhibitors nocodazole, vinblastine, or vincristine (Figure 3D). This is in line with the low similarity of the profile of colchicine to the tubulin cluster subprofile (61%, Figure 3B). Instead, colchicine displays biosimilarity to epothilone B, which is a microtubule stabilizer,^27^ and to parbendazole, which suppresses tubulin polymerization^28^ (Figure S6A). Compound **10** did not inhibit in vitro tubulin polymerization but increased the number of mitotic cells and led to almost complete depolymerization of microtubules in cells (Figure 3E,F). A similar phenotype was observed for 100 nM colchicine (Figure 3F). These findings reveal that different expressions of impaired microtubule dynamics can be captured by CPA that most likely depend on the severity of microtubule disturbance. Increasing the compound concentrations for the explored microtubule-targeting agents reduces the cell count below a threshold of 50% (usually, only profiles with cell count >50% are analyzed). When CPA profiles at higher concentration and a cell count <50% were considered, profile biosimilarity between colchicine and nocodazole, vinblastine, and vincristine was observed (Figure S6B), and biosimilarity to the tubulin cluster as well to the BET and HDAC clusters became evident for colchicine (Figure S6C). This is most likely attributed to a cell death phenotype, as already suggested by the lower cell count.

**Figure 4:**
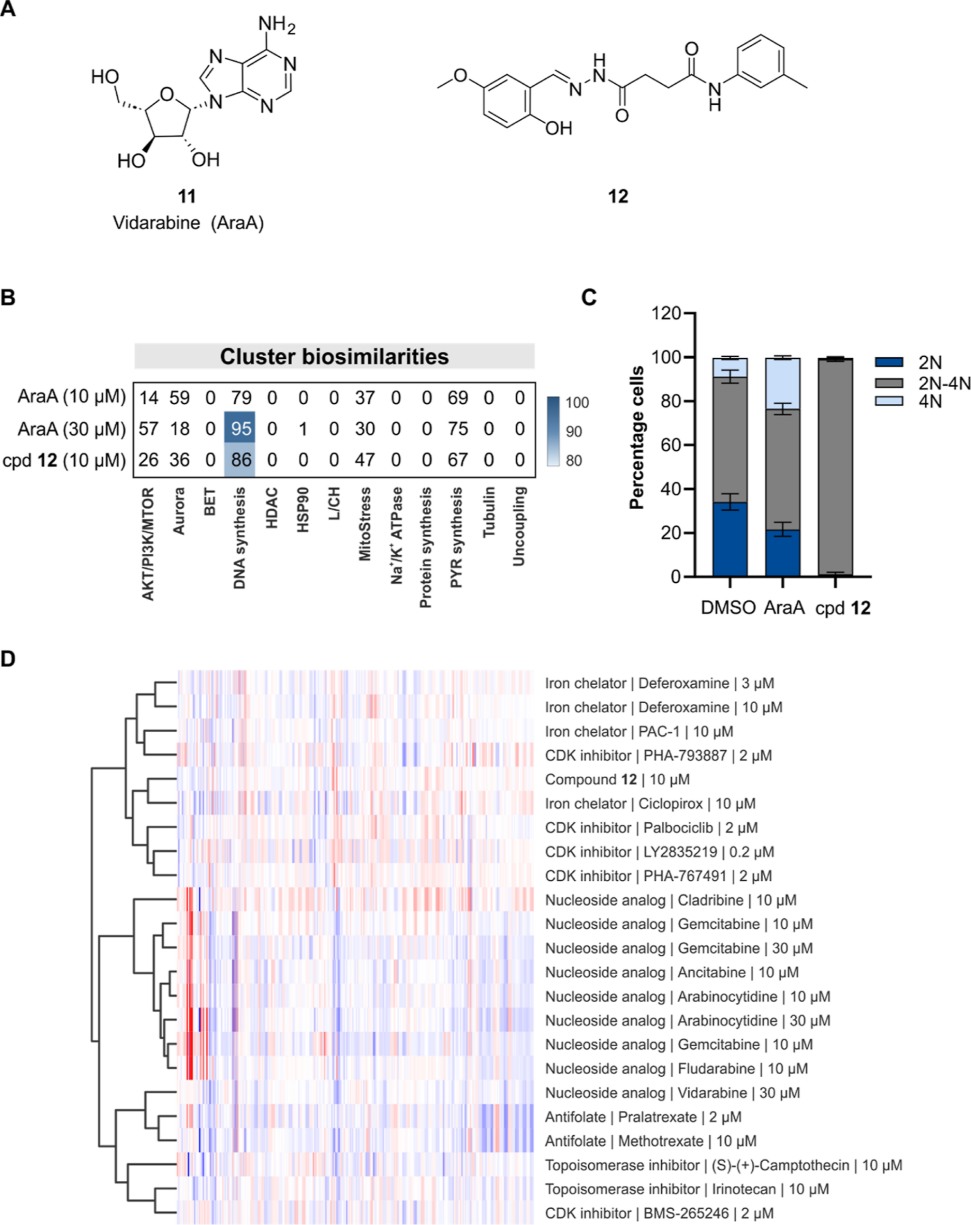
DCM compounds impair DNA synthesis. (A) Structure of DCM compounds **11** and **12** that display biosimilarity to the DNA synthesis cluster. (B) Cluster biosimilarity for vidarabine (AraA, **11**) and compound **12**. PYR: pyrimidine. (C) Influence on DNA content after treatment of U2OS cells for 22 h, followed by an EdU pulse for 2 h. DMSO was used as a control. Compound concentration 30 μM. Data are mean values (*n* = 3). (D) Hierarchical clustering using the non-DNA cluster features for compounds of the DNA synthesis cluster and compound **12**. Only the 291 noncluster features were used. See also Figure S7.

The nucleoside vidarabine (compound **11**) and DCM compound **12** displayed similarity to the DNA synthesis cluster (Figures 4A,B and S7A). Vidarabine (AraA, Figure 4A) is an antiviral agent isolated from *Streptomyces antibioticus* and is known to inhibit viral DNA polymerases.^29^ According to Wassermann et al., vidarabine is inactive in more than hundred biochemical assays.^8^ In contrast, its epimer adenosine (Figure S7B) is an endogenous nucleoside that plays a role in various processes, e.g., inflammation, vasodilation, and angiogenesis.^30^ Besides being a building block for nucleic acids and component of ATP, adenosine activates adenosine receptors.^31^ We recently reported bioactivity for vidarabine in CPA and profile similarity to the iron chelator deferoxamine that originates from inhibition of DNA synthesis or cell cycle arrest in general.^32^ At 10 and 30 μM vidarabine, induction of 13 and 27%, respectively, was detected in CPA with high biosimilarity to the DNA synthesis cluster at 30 μM (Figures 4B and S7A). Of note, adenosine is inactive in CPA up to a concentration of 30 μM (induction <1%). We failed to detect a profile related to activation of adenosine receptors also for adenosine receptor agonists (Figure S7C and Table S2). DNA content analysis using flow cytometry revealed accumulation of cells with 4N DNA content for vidarabine, whereas compound **12** increased the number of cells in the S phase of the cell cycle (corresponding to a DNA content of 2N–4N) (Figure 4C). Hierarchical clustering using the non-DNA cluster features grouped compound **12** with iron chelators (Figure 4D).

**Figure 5:**
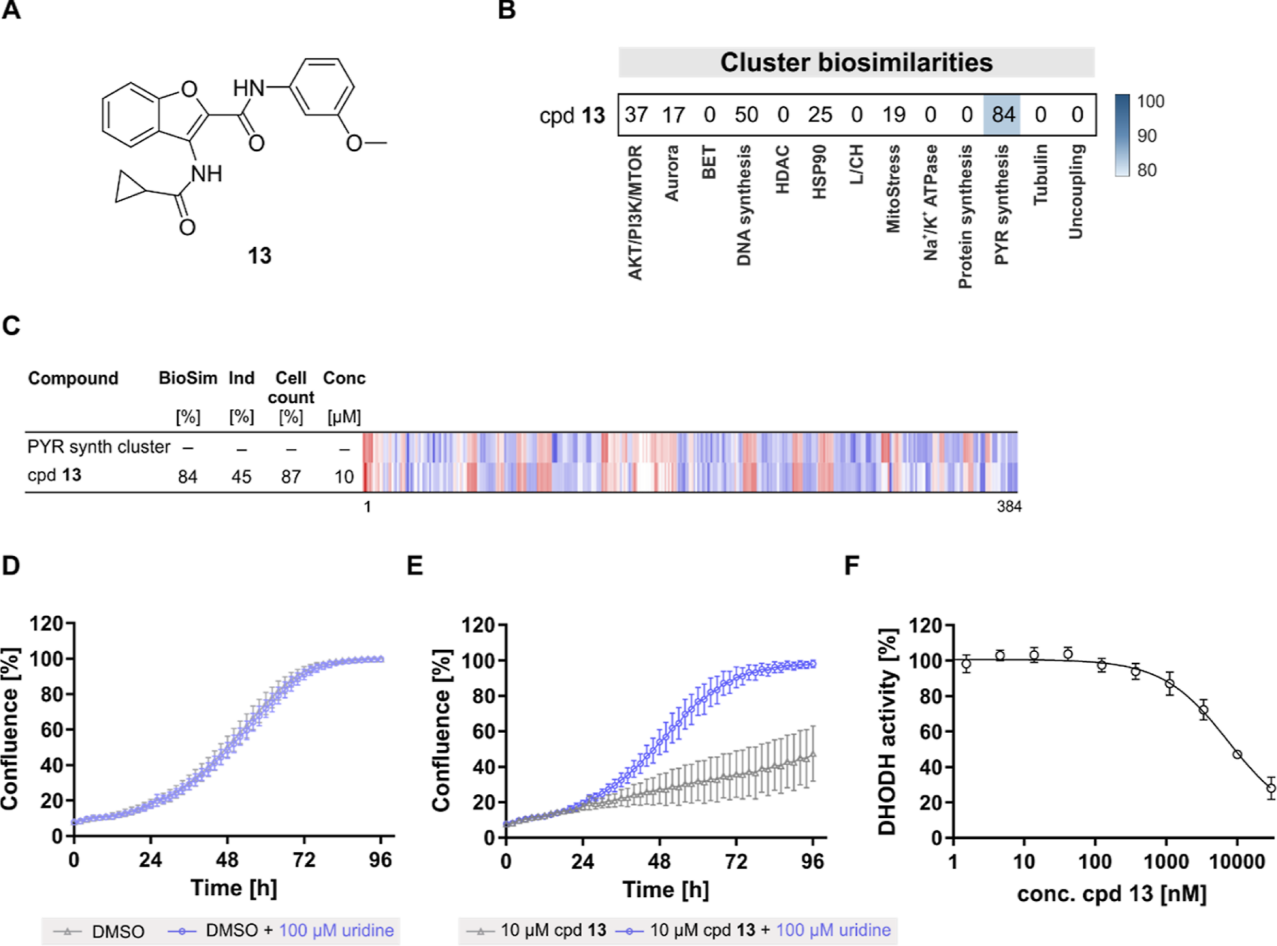
Compound **13** is a DHODH inhibitor. (A) Structure of compound **13**. (B) Similarity of compound (cpd) **13** to the pyrimidine (PYR) synthesis cluster subprofile. The top line profile is set as a reference profile (100% biological similarity, BioSim) to which the following profiles are compared. Blue color: decreased feature and red color: increased feature. BioSim: biosimilarity; Ind: induction; and Conc: concentration. (C) Cluster biosimilarity for compound **13** (10 μM). PYR: pyrimidine. (D,E) Influence on the growth of HCT-116 cells in the absence and presence of 100 μM uridine for DMSO (C) and compound (cpd) **13** (D). Cell confluence was used as a measure of cell growth. Data are mean values ± SD (*n* = 3). (F) Influence of **13** on the enzymatic activity of DHODH. Data are mean values ± SD (*n* = 3).

CPA identified DCM compound **13** as a potential inhibitor of de novo pyrimidine biosynthesis as it displayed 84% similarity to the pyrimidine synthesis cluster (Figure 5A–C). As recently reported, this cluster contains modulators of enzymes in pyrimidine biosynthesis like dihydroorotate dehydrogenase (DHODH) and UMP synthase (UMPS) as well as inhibitors of mitochondrial complex III.^33^**13** suppressed the growth of HCT-116 cells that rely on de novo pyrimidine biosynthesis. This influence was rescued in the presence of uridine, which feeds into the salvage pathway and makes de novo pyrimidine synthesis dispensable (Figure 5D,E). Moreover, **13** dose dependently inhibited DHODH activity in vitro with an approximate IC_50_ value of 7.5 μM (Figure 5F), thus confirming the predicted target. Notably, target prediction based on chemical similarity using SEA revealed DHODH among the predicted targets for compound **13** but only at position 109. Thus, while SEA did not prioritize DHODH as a potential target for further validation, CPA successfully yielded a valuable target hypothesis.

Of the 370 DCM compounds that were reproducibly active in CPA, 182 showed no significant similarity to any of the defined clusters (see Figure 1C). The percentage of DCM compounds that showed activity in CPA but whose profiles cannot be assigned to any of the 13 clusters is 49%. A similar value was obtained for our reference set (i.e., 52%). A UMAP analysis of the full profiles of these DCM compounds together with the profiles of representative compounds from the 13 clusters confirms that these bioactive compounds cover diverse morphological space (Figure S8). Hierarchical clustering was performed for this set of compounds to detect small molecules with similar profiles (Figure S9). Of note, 110 of these compounds (i.e., 60%) lead to induction values <15% (Figure S8), i.e., they have limited activity at 10 μM, which additionally impedes the profile analysis. We disclose the most biosimilar references for these compounds to enable further analysis (GitHub, 10.5281/zenodo.10527972).

**Figure 6:**
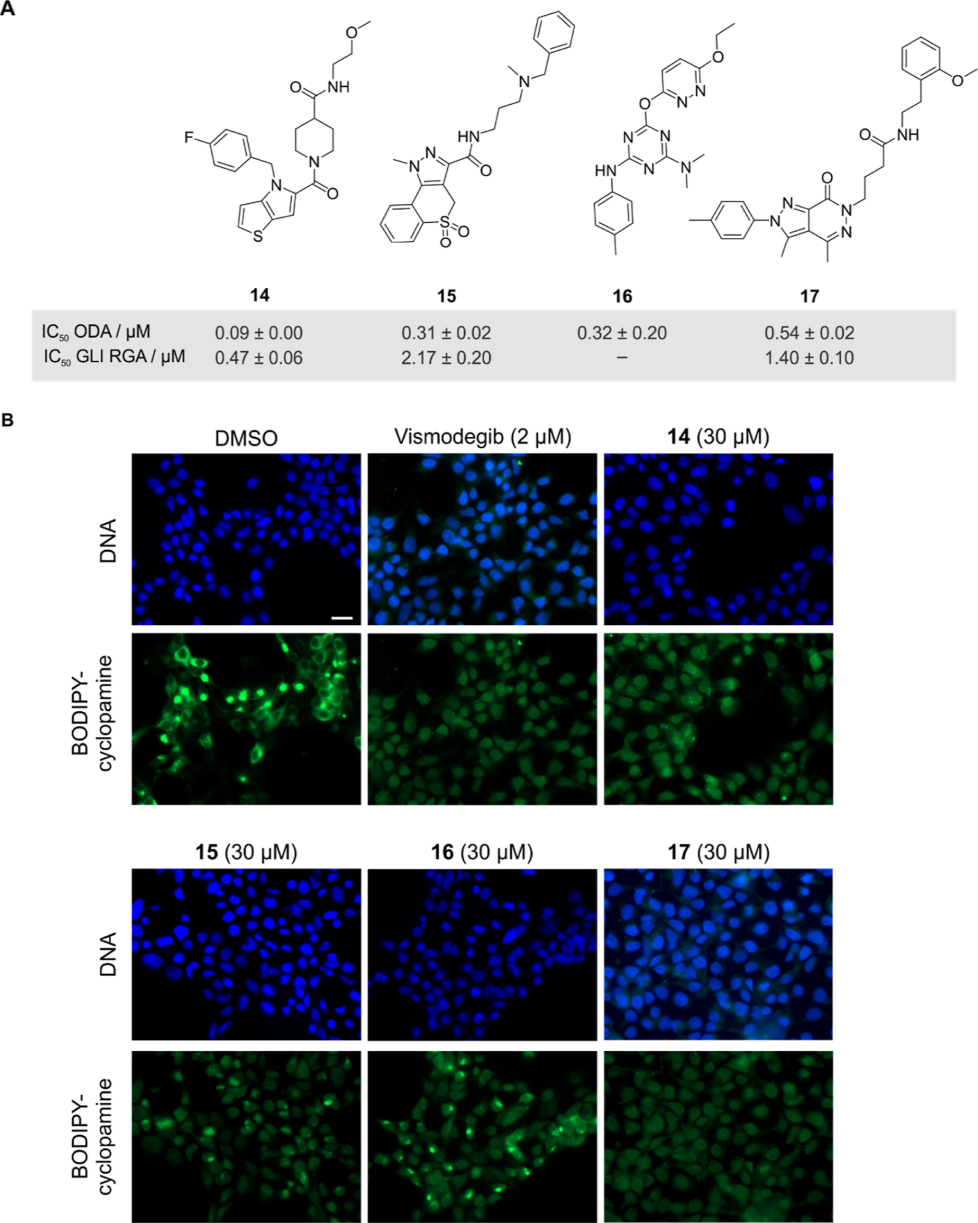
DCM compounds inhibit Hedgehog-dependent osteogenesis. (A) Structure of compounds **14**–**17** and the corresponding IC_50_ values for inhibition of purmorphamine-induced osteoblast differentiation (ODA) and GLI-responsive reporter gene assay (GLI RGA). Data are mean values ±SD (*n* = 3). (B) Competition with BODIPY-cyclopamine for binding to SMO. HEK293T cells expressing SMO were fixed and treated with BODIPY-cyclopamine (green) and the compounds for 4 h. SMO antagonist vismodegib was used as a control. DAPI was used to visualize the DNA. Images are representative of three biological replicates (*n* = 3). Scale bar: 20 μm. See also Table S3.

The biological activity of these small molecules can be further assessed in different target- or cell-based assays. As DCM per definition is composed of compounds that are inactive in at least 100 assays, the choice of the proper setup for assay #101 should be carefully made. In particular, phenotypic assays allow for detection of target modulation in cells. Choosing the right system (primary cells), exogenous stimulus (if appropriate), and a suitable biomarker as a readout (endogenous marker) are essential for the physiological relevance of the assay (and are known as the phenotypic screening “rule of 3”).^34^ To gain further insight into MoA for these 182 CP-active DCM compounds, we selected a Hedgehog-dependent osteogenesis differentiation assay using the murine mesenchymal stem cells, C3H10T1/2, which employs stimulation with the Hedgehog agonist purmorphamine^35^ and complies with this “rule of 3”. Osteoblast differentiation was monitored using the activity of endogenous marker alkaline phosphatase. Twenty-one compounds reduced the activity of alkaline phosphatase without being toxic to the cells with IC_50_ values ranging from 0.5 to 7 μM (see Figure 6A and Table S3). To evaluate a direct effect on the Hedgehog (Hh) pathway, we employed a GLI-dependent reporter gene assay using the Shh-LIGHT2 cells.^36^ Seven compounds (compounds **14**, **15**, and **17**–**21**) dose dependently reduced GLI-mediated gene expression; hence, they directly influence Hh signaling. Most small molecules that modulate Hh signaling target seven-pass transmembrane protein Smoothened (SMO),^37^ and target prediction using the SwissTargetPred tool (http://swisstargetprediction.ch/)^38^ suggested SMO as a target for compound **14**.

We used the SMO antagonist cyclopamine labeled with BODIPY to assess potential binding of DCM compounds to SMO. Similar to SMO antagonist vismodegib, compounds **14**, **15**, and **17** prevented BODIPY-cyclopamine from binding to SMO, indicating that they modulate Hh signaling by antagonizing SMO (Figure 6B). These results are in line with the observed inhibition of GLI-dependent transcription. In contrast, compound **16** did not effectively compete with BODIPY-cyclopamine. This finding, together with the lack of influence on GLI-dependent reporter gene expression, suggests that **16** inhibits purmorphamine-mediated osteogenesis but may not directly target canonical Hh signaling.

## Discussion and Conclusions

Our results demonstrate that phenotypic screening and profiling approaches may be an advantageous strategy to uncover biologically active DCM. Subjecting DCM to cellular assays rather than target-based approaches exposes DCM compounds to multiple targets that go beyond protein targets. Moreover, the unbiased nature of the employed morphological profiling identified different bioactivities for the various DCM compounds. Testing DCM in an increasing number of assays can identify bioactive DCM as demonstrated by Wassermann et al.^10^ and emphasize that, most likely, these DCM compounds have not been exposed yet to the “right” assay. Surprisingly, several CPA-active DCM compounds target microtubules, which are one of the most frequently addressed (off-) targets of small molecules.^26^ Most likely, previous screening activities employing these compounds did not focus on tubulin, and this mechanism of action has remained undetected for this DCM. We identified DCM compounds that interfere with DNA synthesis and the cell cycle. Inhibition of DNA replication can be achieved by various mechanisms that target proteins, DNA directly, or through chelation of Fe(II)/Fe(III), and CPA is particularly suitable for the detection of this mode of action.^32^ This also applies to the modulation of de novo pyrimidine biosynthesis: CPA reliably detects DHODH inhibitors, and many of them show only moderate potency in an in vitro DHODH activity assay and may remain below the activity threshold limit of such an assay.^33^ These examples emphasize the power of morphological profiling to deorphanize DCM as bioactivity is detected in an unbiased manner, which was also demonstrated for gene expression profiling.^8,10^ Employing physiologically relevant phenotypic assays for screening will further support the bioactivity annotation of DCM as exemplified by the Hedgehog-induced osteogenesis assay, which was employed here. Therefore, DCM should not be regarded as inert chemical entities and should be included in screening campaigns as they are expected to be more selective and to show less polypharmacology.^8^

The DCM set used by us was analyzed in Cell Painting irrespective of the structural properties as these compounds were deemed inactive in more than 100 assays and, therefore, are not expected to be promiscuous. Several compounds may, however, bear PAINS structures and a PAINS filter flagged compounds **10** and **12** due to the presence of rhodanine and hydroxyphenyl hydrazone moieties, respectively. Of note, Baell et al. recommend retaining hydroxyphenyl hydrazine in compound libraries.^2,39^ Since a specific activity for both compounds, i.e., interference with microtubule dynamics and DNA synthesis, was detected in CPA and these compounds were inactive in more than 100 assays, they apparently are not PAINS. We analyzed our DCM set of 7677 compounds for the presence of further compounds bearing rhodanine or hydroxyphenyl hydrazone. Six of the 21 rhodanine derivatives and one of six hydroxyphenyl hydrazones were active in CPA (Tables S4 and S5). Hence, the presence of these PAINS moieties itself does not make a compound pan-assay interfering.

All DCM compounds with a validated mode of action should not be considered DCM anymore. Our findings lead to a subset of DCM compounds that are active in CPA and whose profiles are not biosimilar to the 13 previously defined clusters and, therefore, address a different target space. Subjecting this smaller DCM subset to physiologically relevant phenotypic assays may identify novel bioactivities and point toward their mode of action. Furthermore, 12% of the DCM compounds tested by us induced morphological changes that can be mapped by CPA. This hit rate is lower than the rate observed for the references or our in-house compounds (34 and 31%, respectively) and may indicate that indeed part of the DCM may be inert. Inactivity in CPA, however, may also be due to missing target expression, lack of morphological phenotype upon modulation of the target, or lack of a stimulus. We therefore encourage continuing screening of DCM in further assays.

In summary, we employed morphological profiling by means of CPA to explore the apparently biologically inert DCM. Cell Painting proved to be particularly suitable for deorphanization of DCM and identified modulators of microtubule dynamics, DNA synthesis, and DHODH. Phenotypic screening of CPA-active compounds in a physiologically relevant osteoblast differentiation assay additionally identified inhibitors of Hedgehog signaling that target Smoothened. Therefore, exploration of DCM collections in less target-biased phenotypic assays or unbiased profiling approaches promises to detect biologically active DCM compounds, which are expected to exhibit less polypharmacology and may deliver highly selective compounds for chemical biology and medicinal chemistry research.

## Experimental Section

### Materials

Shh-LIGHT2 cells^36^ were maintained in high-glucose DMEM, 10% heat-inactivated fetal calf serum, 1 mM sodium pyruvate, 3 mM l-glutamine, 5 mM Hepes pH 7.4, 100 U/mL penicillin, and 0.1 mg/mL streptomycin. Human kidney cell line HEK293T (ATCC, CRL-11268; RRID:CVCL_1926), human osteosarcoma U2OS cells (CLS #300364; RRID:CVCL_0042), and human colorectal cancer cell line HCT-116 (DSMZ #581; RRID:CVCL_0291) were cultured in Dulbecco’s modified Eagle’s medium (DMEM with 4.5 g/L glucose, l-glutamine, and 3.7 g/L sodium bicarbonate; PAN Biotech, #P04-03550) supplemented with 10% fetal bovine serum (FBS, Invitrogen, cat# 10500-084), 1 mM sodium pyruvate (PAN, #P04-43100), and 1% MEM-nonessential amino acids (PAN, #P08-32100). The cell lines were cultured in a humidified atmosphere at 37 °C and 5% CO_2_. All cells were regularly checked for mycoplasma contaminations, and cells were found to be free of contaminations at all times.

### Cell Painting Assay

The Cell Painting assay follows closely the method described by Bray et al.^12^ as recently reported.^15^ “Initially, 5 μL of U2OS medium was added to each well of a 384-well plate (PerkinElmer CellCarrier-384 Ultra). Subsequently, U2OS cells were seeded with a density of 1600 cells per well in 20 μL of medium. The plate was incubated for 10 min at the ambient temperature, followed by an additional 4 h incubation (37 °C, 5% CO_2_). Compound treatment was performed with an Echo 520 acoustic dispenser (Labcyte). Different concentrations of DMSO were used as controls dependent on the used compound concentration; e.g., 0.1% DMSO was used as a control for the profiling of compounds at 10 μM. Samples at a given compound concentration were compared to the DMSO sample of the same DMSO concentration. Incubation with the compound was performed for 20 h (37 °C, 5% CO_2_). Subsequently, mitochondria were stained with Mito Tracker Deep Red (Thermo Fisher Scientific, Cat. no. M22426). The Mito Tracker Deep Red stock solution (1 mM) was diluted to a final concentration of 100 nM in a prewarmed medium. The medium was removed from the plate leaving 10 μL of residual volume, and 25 μL of the Mito Tracker solution was added to each well. The plate was incubated for 30 min in darkness (37 °C, 5% CO_2_). To fix the cells, 7 μL of 18.5% formaldehyde in PBS was added, resulting in a final formaldehyde concentration of 3.7%. Subsequently, the plate was incubated for another 20 min in darkness (RT) and washed three times with 70 μL of PBS (Biotek Washer Elx405). Cells were permeabilized by addition of 25 μL of 0.1% Triton X-100 to each well, followed by 15 min incubation (RT) in darkness. The cells were washed three times with PBS leaving a final volume of 10 μL. To each well, 25 μL of a staining solution was added, which contains 1% BSA, 5 μL/mL phalloidin (Alexa594 conjugate, Thermo Fisher Scientific, A12381), 25 μg/mL concanavalin A (Alexa488 conjugate, Thermo Fisher Scientific, Cat. no. C11252), 5 μg/mL Hoechst 33342 (Sigma, Cat. no. B2261-25 mg), 1.5 μg/mL WGA-Alexa594 conjugate (Thermo Fisher Scientific, Cat. no. W11262), and 1.5 μM SYTO 14 solution (Thermo Fisher Scientific, Cat. no. S7576). The plate is incubated for 30 min (RT) in darkness and washed three times with 70 μL of PBS. After the final washing step, the PBS was not aspirated. The plates were sealed and centrifuged for 1 min at 500 rpm.

The plates were prepared in triplicates with shifted layouts to reduce plate effects and imaged using a Micro XL High-Content Screening System (Molecular Devices) in 5 channels (DAPI: Ex350–400/Em410–480; FITC: Ex470–500/Em510–540; Spectrum Gold: Ex520–545/Em560–585; TxRed: Ex535–585/Em600–650; and Cy5: Ex605–650/Em670–715) with 9 sites per well and 20× magnification (binning 2).

The generated images were processed with the CellProfiler package (https://cellprofiler.org/, version 3.0.0)^13^ on a computing cluster of the Max Planck Society to extract 1716 cell features per microscope site. The data was then further aggregated as medians per well (9 sites →1 well), then over the three replicates.

Further analysis was performed with custom Python (https://www.python.org/) scripts using the Pandas (https://pandas.pydata.org/) and Dask (https://dask.org/) data processing libraries as well as the Scientific Python (https://scipy.org/) package.

From the total set of 1716 features, a subset of highly reproducible and robust features was determined using the procedure described by Woehrmann et al.^40^ in the following way:

Two biological repeats of one plate containing reference compounds were analyzed. For every feature, its full profile over each whole plate was calculated. If the profiles from the two repeats showed a similarity ≥0.8 (see below), the feature was added to the set.

This procedure was only performed once and resulted in a set of 579 robust features out of the total of 1716 that was used for all further analyses.

The phenotypic profiles were compiled from the *Z*-scores of all individual cellular features, where the *Z*-score is a measure of how far away a data point is from a median value.

Specifically, *Z*-scores of test compounds were calculated relative to the median of DMSO controls. Thus, the *Z*-score of a test compound defines how many MADs (median absolute deviations) the measured value is away from the median of the controls, as illustrated by the following formula:
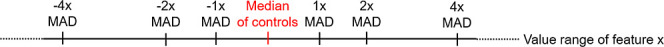

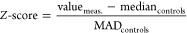


The phenotypic compound profile is then determined as the list of *Z*-scores of all features for one compound.

In addition to the phenotypic profile, an induction value was determined for each compound as the fraction of significantly changed features, in percent
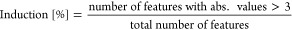


Similarities of phenotypic profiles (termed Biosimilarity) were calculated from the correlation distances (CD) between two profiles (https://docs.scipy.org/doc/scipy/reference/generated/scipy.spatial.distance.correlation.html)^21^
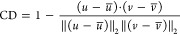
where *x̅* is the mean of the elements of *x*, *x*·*y* is the dot product of *x* and *y*, and ∥*x*∥_2_ is the Euclidean norm of *x*
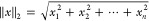


The biosimilarity is then defined as
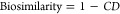


Biosimilarity values smaller than 0 are set to 0, and the biosimilarity is expressed in percent (0–100).”

### Subprofile Analysis

Cluster subprofiles were generated as recently described.^15^ First, biosimilar profiles that describe each cluster were evaluated. For a set of profiles, dominating features were extracted, and the counter for negative or positive values was determined. For all cluster-defining profiles, the maximum of the two counters was divided by the total number of defining profiles. Features were added to a cluster subprofile if the value for a given feature has the same sign (i.e., positive or negative feature values) for 85% of the defining profiles. A median subprofile for each cluster was then calculated. This median subprofile was then used to calculate the biosimilarity of the test compounds to the defined cluster subprofiles. The cluster biosimilarity threshold was set to 80% since cluster subprofiles are shorter than the full profiles.

### Immunocytochemistry

5000 U2OS cells were seeded per well in 96-well plates (Cellvis P96–1-N, 0.13–0.16 mm thickness) and incubated overnight (37 °C, 5% CO_2_). Cells were treated with compounds or DMSO as a control for 24 h. Cells were then fixed using 3.7% paraformaldehyde in PBS, permeabilized with 0.1% Triton X100 (in PBS), and then washed with PBS-T. Subsequently, 100 μL of 2% BSA in PBS-T was added to the cells and incubated for 1 h prior to staining with DAPI (Sigma-Aldrich, D9542-10MG, 1:1000 dilution) to visualize DNA and antitubulin-FITC antibody (Thermo Fisher, MA119581, 1:500 dilution) or antiphospho-histone H3 antibody (Cell Signaling, #8481, 1:500 dilution) overnight at 4 °C. Images were acquired using Observer Z1 (Carl Zeiss, Germany) using 40× objectives (LD Plan-Neofluar). Moreover, an Axiovert 200 M microscope (Carl Zeiss, Germany) equipped with 10× objective was used to detect and quantify phospho-histone H3-positive cells using software MetaMorph 7. For the automated image analysis, the percentage of phospho-histone H3-positive cells was determined using the DNA stain to assess the total number of cells by software CellProfiler. Data presented are mean values (*N* = 3, *n* > 3) ± SD and are representative of three independent experiments.

### In Vitro Tubulin Polymerization Assay

In vitro tubulin polymerization assay was performed as described previously by Akbarzadeh et al.^41^ Porcine α/β-tubulin (cytoskeleton, T240-B) was diluted in a general buffer containing 80 mM PIPES (pH 6.9), 2 mM MgCl_2_, and 0.5 mM EGTA. Next, α/β-tubulin (final concentration 10 μM) was added to a solution glutamate (Sigma-Aldrich, 49621-250G) with a final concentration of 0.8 mM on a 96-well plate (Corning 3696). Subsequently, compounds at a final concentration of 20 μM were added to the tubulin solution and incubated at room temperature for 20 min. The plate was then incubated on ice for another 20 min, followed by the addition of GTP (Thermo Fisher, R0461) to a final concentration of 500 μM. Tubulin polymerization was monitored for 60 min by means of turbidity measurements at 340 nm using an Infinite M200 plate reader (Tecan). Data shown is representative of three independent experiments.

### Flow Cytometry

The Click-it Plus EdU Alexa Fluor 488 Flow Cytometry Assay Kit (Cat. no. C10632, Thermo Fisher Scientific) was used according to the manufacturer’s protocol for analyzing the DNA content by means of flow cytometry. For this, 1.25 × 10^5^ U-2OS cells were seeded per well in 6-well plates and incubated overnight. The following day, cells were treated with the compounds or DMSO as a control for 22 h. Afterward, cells were pulsed with 10 μM EdU (5-ethynyl-2′-deoxyuridine) or medium as a control for another 2 h. Cells were washed with PBS, detached using trypsin, resuspended in PBS, and centrifuged at 300*g* for 7 min at room temperature. After another washing step with 1% BSA in PBS, cells were fixed with 4% PFA in PBS, permeabilized, and subjected to a click reaction for labeling the incorporated EdU. All centrifugation steps after fixation were performed at 900*g* for 7 min at room temperature. DNA was stained with propidium iodide (100 μg/mL propidium iodide, 0.1% (v/v) Triton X-100, and 100 μg/mL DNase-free RNase A in PBS) for 30 min at room temperature. Before analysis, cell suspensions were filtered into FACS tubes through a nylon mesh. For each sample, 10,000 cells were analyzed by the BD LSRII analyzer (Becton Dickinson, USA). FlowJo 10.7.2 software was used for the analysis and quantification of all data. For every analysis, FSC and SSC gating was performed to exclude debris and to select single cells. All experiments were performed in three biological replicates.

### DHODH Enzymatic Assay

The N-truncated form of hDHODH (aa31–395) was expressed and purified as recently reported by Schölermann et al.^33^ The in vitro DHODH assay was performed according to Schölermann et al.^33^ Briefly, the assay monitored the reduction of 2,6-dichlorophenolindophenol (DCPIP) that was coupled to the oxidation of dihydroorotate by DHODH. 40 μL of purified DHODH (aa31–395, final concentration 1.25 μg/mL) in assay buffer (50 mM Tris pH 8.0, 150 mM KCl, and 0.1% Triton X-100) was incubated with 10 μL of the compounds for 30 min at 37 °C, followed by 15 min at room temperature. The reaction was initiated by adding 2 mM l-dihydroorotic acid, 0.2 mM decylubiquinone, and 0.12 mM 2,6-dichlorophenolindophenol (DCPIP) in assay buffer. DCPIP reduction was monitored at 600 nm using a Tecan Spark plate reader for 30 min. The slope of the linear curves over 5 min was calculated to determine the inhibitory activity and IC_50_ values. Values were normalized to the DMSO control, and the data were analyzed using GraphPad Prism 9 software.

### Cell Growth Determination and Uridine Rescue

2000 HCT-116 cells were seeded per well in a clear 96-well plate and incubated overnight. The next day, the medium was replaced by medium containing the compound or DMSO in the presence or absence of 100 μM uridine. Cell growth was monitored by means of real-time live-cell analysis with IncuCyte S3 (Essen BioScience). Images were acquired every 2 h for a period of 96 h after treatment. The cell confluence was quantified as a measure of cell growth using IncuCyte S3 software (Essen BioScience).

### Osteoblast Differentiation Assay

For assaying signal transduction through the Hh pathway, mouse embryonic mesoderm fibroblast C3H10T1/2 cells were used. These multipotent mesenchymal progenitor cells differentiate into osteoblasts upon treatment with the SMO agonist purmorphamine.^42^ During differentiation, osteoblast-specific genes such as alkaline phosphatase, which plays an essential role in bone formation, are highly expressed. Activity of alkaline phosphatase can be directly monitored by following substrate hydrolysis, yielding a highly luminescent product. Inhibition of the pathway results in a reduction of luminescence. Shortly, 800 cells per well were seeded in white 384 well plates (Greiner) in 25 μL of medium (high glucose DMEM, 10% heat-inactivated fetal calf serum, 1 mM sodium pyruvate, 6 mM l-glutamine, 100 U/mL penicillin, and 0.1 mg/mL streptomycin) and allowed to grow overnight. The next day, compounds were added to a final concentration of 10 μM using an acoustic nanoliter dispenser, ECHO 520 (Beckman). After 1 h, 10 μL of purmorphamine in medium was added to a final concentration of 1.5 μM using Multidrop Combi (Thermo Fisher Scientific); control cells were treated with DMSO. After 4 days, the cell culture medium was aspirated using the Elx405 cell washer (Biotek), and 25 μL of a commercial luminogenic ALK substrate (CDP-Star, Roche) was added. After 1 h, luminescence was read. To identify and exclude toxic compounds that also lead to a reduction in the luminescence signal, cell viability measurements were carried out in parallel. The cell viability assay followed the same workflow as the Hedgehog assay except that only 200 cells per well were seeded. Cell culture medium alone served as a control for the cell viability assay. For the measurement of cell viability, 15 μL of CellTiterGlo reagent (Promega) which determines the cellular ATP content was added after aspiration of the medium. All data was normalized to DMSO-treated cells. Dose–response analysis was done using a 3-fold dilution curve starting from 10 μM. IC_50_ values were calculated using the Quattro software suite (Quattro Research GmbH).

### GLI-Dependent Reporter Gene Assay

For evaluation of a direct effect on the Hh signaling pathway, a reporter gene assay using Shh-LIGHT2 cells was carried out.^36^ Shortly, 7500 cells per well were seeded in white 384-well plates (Greiner) in 25 μL of medium (high-glucose DMEM, 10% heat-inactivated fetal calf serum, 1 mM sodium pyruvate, 3 mM l-glutamine, 5 mM Hepes pH 7.4, 100 U/mL penicillin, and 0.1 mg/mL streptomycin) and allowed to grow overnight. The next day, compounds were added to a final concentration of 10 μM using the acoustic nanoliter dispenser, ECHO 520 (Beckman). After 1 h, 10 μL of purmorphamine in medium was added to a final concentration of 3 μM using Multidrop Combi (Thermo Fisher Scientific); control cells were treated with DMSO. After 48 h, 35 μL of OneGlo reagent (Promega) was added, and luminescence was read. Cell viability measurements were carried out in parallel using 750 cells/well and read out by CellTiterGlo reagent. All data was normalized to DMSO-treated cells. Dose–response analysis was done using a 3-fold dilution curve starting from 10 μM. IC_50_ values were calculated using the Quattro software suite (Quattro Research GmbH).

### Smoothened Binding Assay

A previously described method was modified to use the Smoothened binding assay.^43^ In a 24-well plate with poly-d-lysine-coated coverslips (Neuvitro, 12 mm, #GG-12–1.5-PDL), 6 × 10^4^ HEK293T cells were seeded for 24 h at 37 °C with 5% CO_2_. According to the manufacturer’s instructions, the cells were transfected with the SMO-expressing plasmid (pGEN_mSMO, Addgene #37673^36^) in OptiMEM medium using FuGENE HD transfection reagent (Promega, # E2311). The plate was then kept at 37 °C in 5% CO_2_ for 48 h. The cells were then washed once with PBS, fixed with 3.7% paraformaldehyde in PBS for 10 min at room temperature, and treated for 5 min with PBS containing 10 mM glycine and 0.2% sodium azide. The fixed cells were then washed with PBS (3 times for 5 min) and treated with compounds vismodegib (Selleckchem #1082) and DMSO in DMEM containing 0.5% FBS (assay medium) and 5 nM BODIPY-cyclopamine S26 (Carbosynth Limited, FB18988) for 4 h at room temperature in the dark. After that, coverslips were washed with PBS and incubated for 10 min at room temperature with 1 g/mL 4′,6 diamidino-2-phenylindole (DAPI, Sigma-Aldrich, Roche, #10236276001) in PBS. Coverslips were then washed again and mounted onto glass slides using Aqua Polymount (Polysciences). Fluorescence microscopy, Zeiss Observer Z1 (Carl Zeiss, Germany) was used to acquire the images using a Plan-Apochromat 63*x*/1.40 Oil DIC M27 objective.

### Target Prediction Based on Chemical Similarity

Similarity Ensemble Approach (SEA, https://sea.bkslab.org)^25^ and SwissTargetPred tool (http://swisstargetprediction.ch/)^38^ were used to predict targets for the DCM compounds based on chemical similarity.

### Pan-Assay Interference and Compound Purity

The screened compounds represent a subset of the Dark Chemical Matter that is defined as compounds that are inactive in more than 100 assays,^8^ and therefore, pan-assay interference can be excluded. The PAINS filter by Saubern et al. was used to detect problematic moieties.^44^ All tested compounds were obtained from commercial sources, mostly from ChemDiv. ChemDiv guarantees at least 90% purity. The purity of the compounds that were studied in detail is >95% besides compounds **8**, **12**, and **24** with 87, 85, and 94%, respectively.

### Quantification and Statistical Analysis

Data were either representative of independent experiments or expressed as mean ± SD. All statistical details of the conducted experiments can be found in the respective figure caption. n: number of biological replicates. N: number of technical replicates.

## Data Availability

The data underlying this study are available in the published article and the Supporting Information. The CPA profiles for the 549 compounds that were tested twice and their biosimilarity to the 13 clusters are openly available at GitHub (DOI: 10.5281/zenodo.10527972). The data for the 182 DCM compounds whose profiles do not show similarity to any of the 13 defined clusters is available at GitHub (DOI: 10.5281/zenodo.10527972).

## Supporting Information Available

Characterization of the DCM set, diversity selection, reproducibility for DCM in Cell Painting, calculated logP and bpKa values for CPA-active DCM, influence of compounds **1**–**3**, **5**, and **7**–**9** on microtubule cytoskeleton, CPA analysis for colchicine, CPA analysis for compounds with similarity to the DNA synthesis cluster, UMAP plot of the profile of the 182 DCM compounds that are not similar to the 13 defined clusters, CPA data for adenosine receptor agonists, DCM compounds that inhibit Hedgehog-induced osteogenesis, studied rhodanine-bearing DCM compounds, hydroxyphenyl hydrazone compounds in the studied DCM set, and hierarchical clustering for DCM compounds whose profiles are not biosimilar to the 13 defined clusters (PDF)
Hierarchical clustering (PDF)
DCM active in CPA (XLSX)
Molecular formula strings (CSV)

## Notes

CP data for the 182 compounds whose profiles lack similarity to the 13 previously defined clusters (GitHub, DOI: 10.5281/zenodo.10527972).

## Abbreviations USED

Ara A: 9-β-d-arabinofuranosyladenine
BET: bromodomain and extra-terminal motif
BioSim: biosimilarity
CDK: cyclin-dependent kinase
CLC: colchicine
CPA: Cell Painting assay
cpd: compound
DAPI: 4′,6-diamidino-2-phenylindole)
DCM: dark chemical matter
DHODH: dihydroorotate dehydrogenase
EdU: 5-ethynyl-2′-deoxyuridine)
Gem: gemcitabine
GLI: glioma-associated oncogene
HDAC: histone deacetylase
Hh: Hedgehog
HSP90: heat shock protein 90
L/CH: lysosomotropism/cholesterol homeostasis
MTOR: mammalian/mechanistic target of rapamycin
Noc: nocodazole
ODA: osteoblast differentiation assay
PAINS: pan-assay interference compounds
PI3K: phosphatidylinositol 3-kinase
PYR: pyrimidine
RGA: reporter gene assay
SEA: similarity ensemble approach
SHH: Sonic hedgehog
SMO: Smoothened
Synth: synthesis
UMPS: UMP synthase

## References

[ref1] Thorne N.; Auld D. S.; Inglese J. Apparent Activity in High-Throughput Screening: Origins of Compound-Dependent Assay Interference. Curr. Opin. Chem. Biol. 2010, 14, 315–324. 10.1016/j.cbpa.2010.03.020. 20417149 PMC2878863

[ref2] Baell J. B.; Holloway G. A. New Substructure Filters for Removal of Pan Assay Interference Compounds (Pains) from Screening Libraries and for Their Exclusion in Bioassays. J. Med. Chem. 2010, 53, 2719–2740. 10.1021/jm901137j. 20131845

[ref3] Wetzel S.; Bon R. S.; Kumar K.; Waldmann H. Biology-Oriented Synthesis. Angew. Chem., Int. Ed. Engl. 2011, 50, 10800–10826. 10.1002/anie.201007004. 22038946

[ref4] van Hattum H.; Waldmann H. Biology-Oriented Synthesis: Harnessing the Power of Evolution. J. Am. Chem. Soc. 2014, 136, 11853–11859. 10.1021/ja505861d. 25074019

[ref5] Schreiber S. L. Organic Chemistry: Molecular Diversity by Design. Nature 2009, 457, 153–154. 10.1038/457153a. 19129834

[ref6] Grigalunas M.; Brakmann S.; Waldmann H. Chemical Evolution of Natural Product Structure. J. Am. Chem. Soc. 2022, 144, 3314–3329. 10.1021/jacs.1c11270. 35188375 PMC8895405

[ref7] Karageorgis G.; Foley D. J.; Laraia L.; Waldmann H. Principle and Design of Pseudo-Natural Products. Nat. Chem. 2020, 12, 227–235. 10.1038/s41557-019-0411-x. 32015480

[ref8] Wassermann A. M.; Lounkine E.; Hoepfner D.; Le Goff G.; King F. J.; Studer C.; Peltier J. M.; Grippo M. L.; Prindle V.; Tao J.; Schuffenhauer A.; Wallace I. M.; Chen S.; Krastel P.; Cobos-Correa A.; Parker C. N.; Davies J. W.; Glick M. Dark Chemical Matter as a Promising Starting Point for Drug Lead Discovery. Nat. Chem. Biol. 2015, 11, 958–966. 10.1038/nchembio.1936. 26479441

[ref9] Pope A. Screening Heursitcs and Chemical Propery Bias; New Directions for Lead Identification and Optimization. In Presented at the Society for Laboratory Automation and Screening (SLAS) Meeting, San Diego, CA, February 4–8, 2012, 2012. https://www.slideshare.net/andypopeuk/screening-heuristics-popefinal.

[ref10] Wassermann A. M.; Tudor M.; Glick M. Deorphanization Strategies for Dark Chemical Matter. Drug Discovery Today: Technol. 2017, 23, 69–74. 10.1016/j.ddtec.2016.11.004. 28647088

[ref11] Gustafsdottir S. M.; Ljosa V.; Sokolnicki K. L.; Anthony Wilson J.; Walpita D.; Kemp M. M.; Petri Seiler K.; Carrel H. A.; Golub T. R.; Schreiber S. L.; Clemons P. A.; Carpenter A. E.; Shamji A. F. Multiplex Cytological Profiling Assay to Measure Diverse Cellular States. PLoS One 2013, 8, e80999 10.1371/journal.pone.0080999. 24312513 PMC3847047

[ref12] Bray M. A.; Singh S.; Han H.; Davis C. T.; Borgeson B.; Hartland C.; Kost-Alimova M.; Gustafsdottir S. M.; Gibson C. C.; Carpenter A. E. Cell Painting, a High-Content Image-Based Assay for Morphological Profiling Using Multiplexed Fluorescent Dyes. Nat. Protoc. 2016, 11, 1757–1774. 10.1038/nprot.2016.105. 27560178 PMC5223290

[ref13] Carpenter A. E.; Jones T. R.; Lamprecht M. R.; Clarke C.; Kang I. H.; Friman O.; Guertin D. A.; Chang J. H.; Lindquist R. A.; Moffat J.; Golland P.; Sabatini D. M. Cellprofiler: Image Analysis Software for Identifying and Quantifying Cell Phenotypes. Genome Biol. 2006, 7, R100 10.1186/gb-2006-7-10-r100. 17076895 PMC1794559

[ref14] Christoforow A.; Wilke J.; Binici A.; Pahl A.; Ostermann C.; Sievers S.; Waldmann H. Design, Synthesis, and Phenotypic Profiling of Pyrano-Furo-Pyridone Pseudo Natural Products. Angew. Chem., Int. Ed. Engl. 2019, 58, 14715–14723. 10.1002/anie.201907853. 31339620 PMC7687248

[ref15] Pahl A.; Scholermann B.; Lampe P.; Rusch M.; Dow M.; Hedberg C.; Nelson A.; Sievers S.; Waldmann H.; Ziegler S. Morphological Subprofile Analysis for Bioactivity Annotation of Small Molecules. Cell Chem. Biol. 2023, 30, 839–853. 10.1016/j.chembiol.2023.06.003. 37385259

[ref16] Rezaei Adariani S.; Agne D.; Koska S.; Burhop A.; Warmers J.; Janning P.; Metz M.; Pahl A.; Sievers S.; Waldmann H.; Ziegler S. Detection of a Mitochondrial Stress Phenotype Using the Cell Painting Assay. bioRxiv 2023, 10.1101/2023.11.08.565491. PMC11320566 39018123

[ref17] Https://Www.Chemdiv.Com/Catalog/Focused-and-Targeted-Libraries/Dark-Chemical-Matter-Library/.

[ref18] Zinken S.; Pahl A.; Grigalunas M.; Waldmann H. Phenotypic Profiling Enables the Targeted Design of a Novel Pseudo-Natural Product Class. Tetrahedron 2023, 143, 133553 10.1016/j.tet.2023.133553.

[ref19] Laraia L.; Waldmann H. Natural Product Inspired Compound Collections: Evolutionary Principle, Chemical Synthesis, Phenotypic Screening, and Target Identification. Drug Discovery Today: Technol. 2017, 23, 75–82. 10.1016/j.ddtec.2017.03.003. 28647090

[ref20] Kumar K.; Waldmann H. Synthesis of Natural Product Inspired Compound Collections. Angew. Chem., Int. Ed. 2009, 48, 3224–3242. 10.1002/anie.200803437. 19267376

[ref21] Grigalunas M.; Burhop A.; Zinken S.; Pahl A.; Gally J. M.; Wild N.; Mantel Y.; Sievers S.; Foley D. J.; Scheel R.; Strohmann C.; Antonchick A. P.; Waldmann H. Natural Product Fragment Combination to Performance-Diverse Pseudo-Natural Products. Nat. Commun. 2021, 12, 1883 10.1038/s41467-021-22174-4. 33767198 PMC7994817

[ref22] Grigalunas M.; Burhop A.; Christoforow A.; Waldmann H. Pseudo-Natural Products and Natural Product-Inspired Methods in Chemical Biology and Drug Discovery. Curr. Opin. Chem. Biol. 2020, 56, 111–118. 10.1016/j.cbpa.2019.10.005. 32362382

[ref23] Schneidewind T.; Brause A.; Scholermann B.; Sievers S.; Pahl A.; Sankar M. G.; Winzker M.; Janning P.; Kumar K.; Ziegler S.; Waldmann H. Combined Morphological and Proteome Profiling Reveals Target-Independent Impairment of Cholesterol Homeostasis. Cell Chem. Biol. 2021, 28, 1780–1794.e5. 10.1016/j.chembiol.2021.06.003. 34214450

[ref24] Nadanaciva S.; Lu S. Y.; Gebhard D. F.; Jessen B. A.; Pennie W. D.; Will Y. A High Content Screening Assay for Identifying Lysosomotropic Compounds. Toxicol. in Vitro 2011, 25, 715–723. 10.1016/j.tiv.2010.12.010. 21184822

[ref25] Keiser M. J.; Roth B. L.; Armbruster B. N.; Ernsberger P.; Irwin J. J.; Shoichet B. K. Relating Protein Pharmacology by Ligand Chemistry. Nat. Biotechnol. 2007, 25, 197–206. 10.1038/nbt1284. 17287757

[ref26] Comess K. M.; McLoughlin S. M.; Oyer J. A.; Richardson P. L.; Stockmann H.; Vasudevan A.; Warder S. E. Emerging Approaches for the Identification of Protein Targets of Small Molecules - a Practitioners’ Perspective. J. Med. Chem. 2018, 61, 8504–8535. 10.1021/acs.jmedchem.7b01921. 29718665

[ref27] Altmann K. H.; Wartmann M.; O’Reilly T. Epothilones and Related Structures-a New Class of Microtubule Inhibitors with Potent in Vivo Antitumor Activity. Biochim. Biophys. Acta 2000, 1470, M79–91. 10.1016/S0304-419X(00)00009-3. 10799747

[ref28] Lacey E.; Watson T. R. Structure-Activity-Relationships of Benzimidazole Carbamates as Inhibitors of Mammalian Tubulin, Invitro. Biochem. Pharmacol. 1985, 34, 1073–1077. 10.1016/0006-2952(85)90611-2. 3985991

[ref29] Shipman C. Jr; Smith S. H.; Carlson R. H.; Drach J. C. Antiviral Activity of Arabinosyladenine and Arabinosylhypoxanthine in Herpes Simplex Virus-Infected Kb Cells: Selective Inhibition of Viral Deoxyribonucleic Acid Synthesis in Synchronized Suspension Cultures. Antimicrob. Agents Chemother. 1976, 9, 120–127. 10.1128/AAC.9.1.120. 176927 PMC429485

[ref30] Fredholm B. B. Adenosine, an Endogenous Distress Signal, Modulates Tissue Damage and Repair. Cell Death Differ. 2007, 14, 1315–1323. 10.1038/sj.cdd.4402132. 17396131

[ref31] Chen J. F.; Eltzschig H. K.; Fredholm B. B. Adenosine Receptors as Drug Targets - What Are the Challenges?. Nat. Rev. Drug Discovery 2013, 12, 265–286. 10.1038/nrd3955. 23535933 PMC3930074

[ref32] Schneidewind T.; Brause A.; Pahl A.; Burhop A.; Mejuch T.; Sievers S.; Waldmann H.; Ziegler S. Morphological Profiling Identifies a Common Mode of Action for Small Molecules with Different Targets. Chembiochem 2020, 21, 3197–3207. 10.1002/cbic.202000381. 32618075 PMC7754162

[ref33] Schölermann B.; Bonowski J.; Grigalunas M.; Burhop A.; Xie Y.; Hoock J. G. F.; Liu J.; Dow M.; Nelson A.; Nowak C.; Pahl A.; Sievers S.; Ziegler S. Identification of Dihydroorotate Dehydrogenase Inhibitors Using the Cell Painting Assay. Chembiochem 2022, 23, e202200475 10.1002/cbic.202200475. 36134475 PMC9828254

[ref34] Vincent F.; Loria P.; Pregel M.; Stanton R.; Kitching L.; Nocka K.; Doyonnas R.; Steppan C.; Gilbert A.; Schroeter T.; Peakman M. C. Developing Predictive Assays: The Phenotypic Screening ″Rule of 3″. Sci. Transl. Med. 2015, 7, 293ps215 10.1126/scitranslmed.aab1201. 26109101

[ref35] Flegel J.; Shaaban S.; Jia Z. J.; Schulte B.; Lian Y.; Krzyzanowski A.; Metz M.; Schneidewind T.; Wesseler F.; Flegel A.; Reich A.; Brause A.; Xue G.; Zhang M.; Dotsch L.; Stender I. D.; Hoffmann J. E.; Scheel R.; Janning P.; Rastinejad F.; Schade D.; Strohmann C.; Antonchick A. P.; Sievers S.; Moura-Alves P.; Ziegler S.; Waldmann H. The Highly Potent Ahr Agonist Picoberin Modulates Hh-Dependent Osteoblast Differentiation. J. Med. Chem. 2022, 65, 16268–16289. 10.1021/acs.jmedchem.2c00956. 36459434 PMC9791665

[ref36] Taipale J.; Chen J. K.; Cooper M. K.; Wang B.; Mann R. K.; Milenkovic L.; Scott M. P.; Beachy P. A. Effects of Oncogenic Mutations in Smoothened and Patched Can Be Reversed by Cyclopamine. Nature 2000, 406, 1005–1009. 10.1038/35023008. 10984056

[ref37] Wu F.; Zhang Y.; Sun B.; McMahon A. P.; Wang Y. Hedgehog Signaling: From Basic Biology to Cancer Therapy. Cell Chem. Biol. 2017, 24, 252–280. 10.1016/j.chembiol.2017.02.010. 28286127 PMC7442121

[ref38] Daina A.; Michielin O.; Zoete V. Swisstargetprediction: Updated Data and New Features for Efficient Prediction of Protein Targets of Small Molecules. Nucleic Acids Res. 2019, 47, W357–W364. 10.1093/nar/gkz382. 31106366 PMC6602486

[ref39] Baell J. B.; Nissink J. W. M. Seven Year Itch: Pan-Assay Interference Compounds (Pains) in 2017-Utility and Limitations. ACS Chem. Biol. 2018, 13, 36–44. 10.1021/acschembio.7b00903. 29202222 PMC5778390

[ref40] Woehrmann M. H.; Bray W. M.; Durbin J. K.; Nisam S. C.; Michael A. K.; Glassey E.; Stuart J. M.; Lokey R. S. Large-Scale Cytological Profiling for Functional Analysis of Bioactive Compounds. Mol. BioSyst. 2013, 9, 2604–2617. 10.1039/c3mb70245f. 24056581

[ref41] Akbarzadeh M.; Deipenwisch I.; Schoelermann B.; Pahl A.; Sievers S.; Ziegler S.; Waldmann H. Morphological Profiling by Means of the Cell Painting Assay Enables Identification of Tubulin-Targeting Compounds. Cell Chem. Biol. 2022, 29, 1053–1064.e3. 10.1016/j.chembiol.2021.12.009. 34968420

[ref42] Wu X.; Ding S.; Ding Q.; Gray N. S.; Schultz P. G. A Small Molecule with Osteogenesis-Inducing Activity in Multipotent Mesenchymal Progenitor Cells. J. Am. Chem. Soc. 2002, 124, 14520–14521. 10.1021/ja0283908. 12465946

[ref43] Sinha S.; Chen J. K. Purmorphamine Activates the Hedgehog Pathway by Targeting Smoothened. Nat. Chem. Biol. 2006, 2, 29–30. 10.1038/nchembio753. 16408088

[ref44] Saubern S.; Guha R.; Baell J. B. Knime Workflow to Assess Pains Filters in Smarts Format. Comparison of Rdkit and Indigo Cheminformatics Libraries. Molecular Informatics 2011, 30, 847–850. 10.1002/minf.201100076. 27468104

