# Supplementary material for: Illuminating Dark Chemical Matter Using the Cell Painting Assay
Source: J Med Chem. 2024 Apr 30;67(11):8862–76. doi: 10.1021/acs.jmedchem.4c00160 (PMC11181314; doi:10.1021/acs.jmedchem.4c00160)
Supplement: Supplementary file 1 — jm4c00160_si_001.pdf [file jm4c00160_si_001.pdf]

Supporting Information

Illuminating Dark Chemical Matter using the Cell Painting Assay

Axel Pahl,[1] Jie Liu,[1] Sohan Patil,[1] Soheila Rezaei Adariani,[1,2] Beate Schölermann,[1] Jens Warmers,[1,2] Jana Bonowski,[1] Sandra Koska,[1] Yasemin Akbulut,[1] Carina Seitz,[1] Sonja Sievers,[1] Slava Ziegler,*[1] Herbert Waldmann*[1,2]

[1] Max-Planck Institute of Molecular Physiology, Department of Chemical Biology, Otto-Hahn-Strasse 11, Dortmund 44227, Germany

E-mail: slava.ziegler@mpi-dortmund.mpg.de; herbert.waldmann@mpi-dortmund.mpg.de

[2] Technical University Dortmund, Faculty of Chemistry and Chemical Biology, Otto-Hahn-Strasse 6, Dortmund 44227, Germany

Table of contents

Supporting Figures 2

Supporting Tables 11

HPLC traces 18

References 121

Supporting Figures

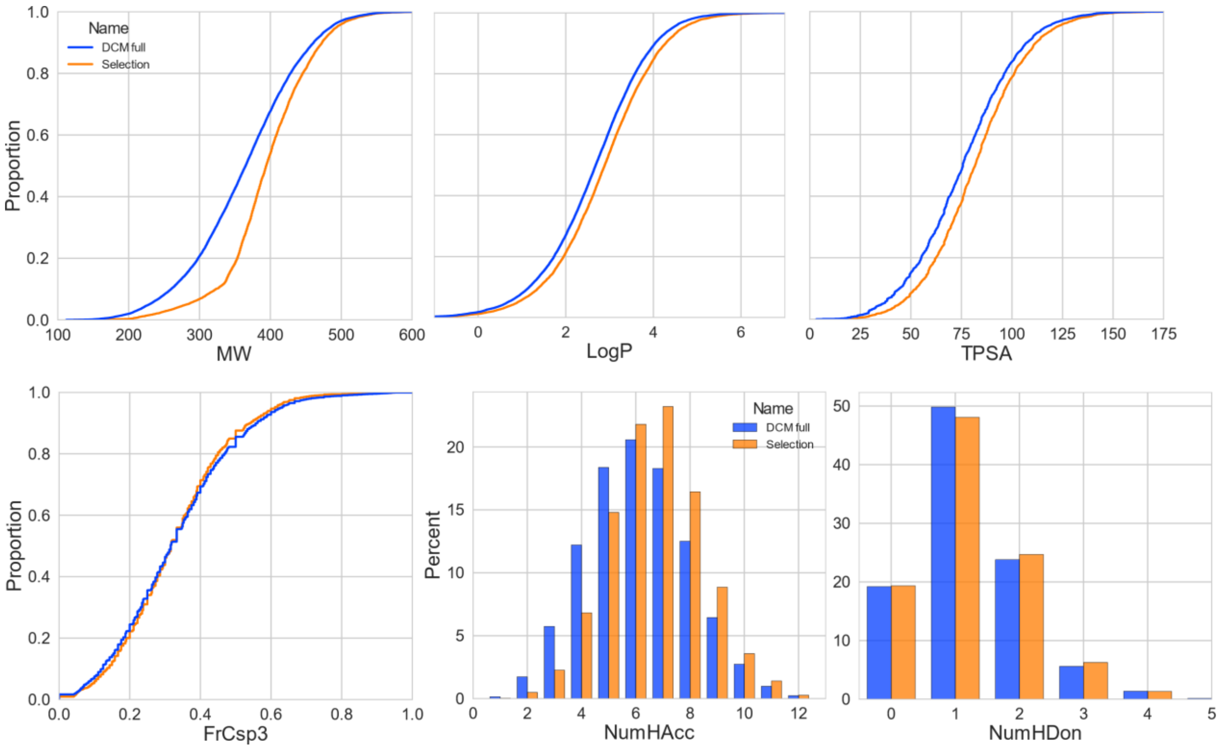

Figure S1 (related to Figure 1). Distributions (ECDF resp. histogram) of six descriptors for the full DCM dataset of 19,976 compounds from ChemDiv and the investigated selection of 7,677 compounds; MW: molecular weight, logP: base-10 logarithm of the octanol/water coefficient, TPSA: topological polar surface area, FrCsp3: fraction of sp^3-hybridized carbons, NumHAcc: number of hydrogen acceptors, NumHDon: number of hydrogen donors; All properties were calculated using RDKit.

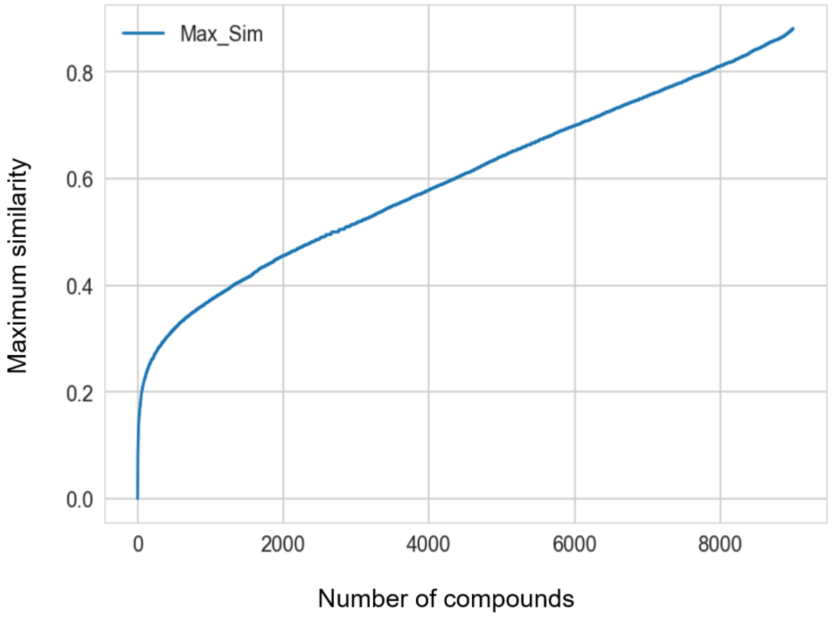

Figure S2 (related to Figure 1). Increasing maximum chemical similarity of compounds added to the set during the diversity selection. The similarity was determined by Tanimoto similarity of the RDKit Morgan fingerprints (radius 2).

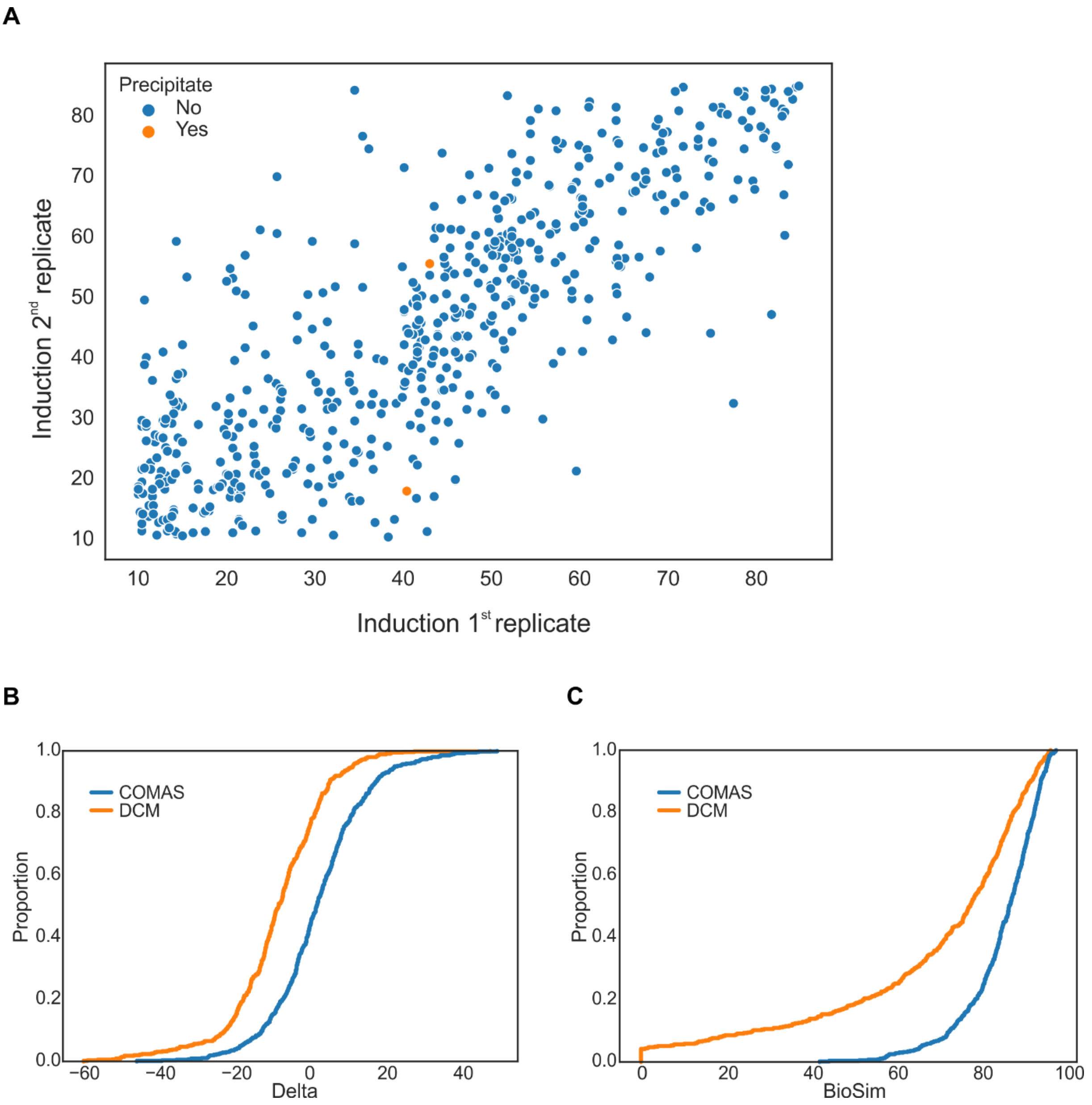

Figure S3 (related to Figure 1). Reproducibility for DCM in the Cell painting. (A) Reproducibility of the induced changes as determined by the induction value for two replicates for 562 CPA-active internal compounds. Correlation between the replicates: $r^2=0.617$. (B) Empirical cumulative distribution function for differences in induction (2nd replicate – 1st replicate) of 562 internal compounds and the 549 DCM compounds. (C) Empirical cumulative distribution functions for the profile similarity (biosimilarity, BioSim) between the two biological replicates for the 562 internal and 549 DCM compounds.

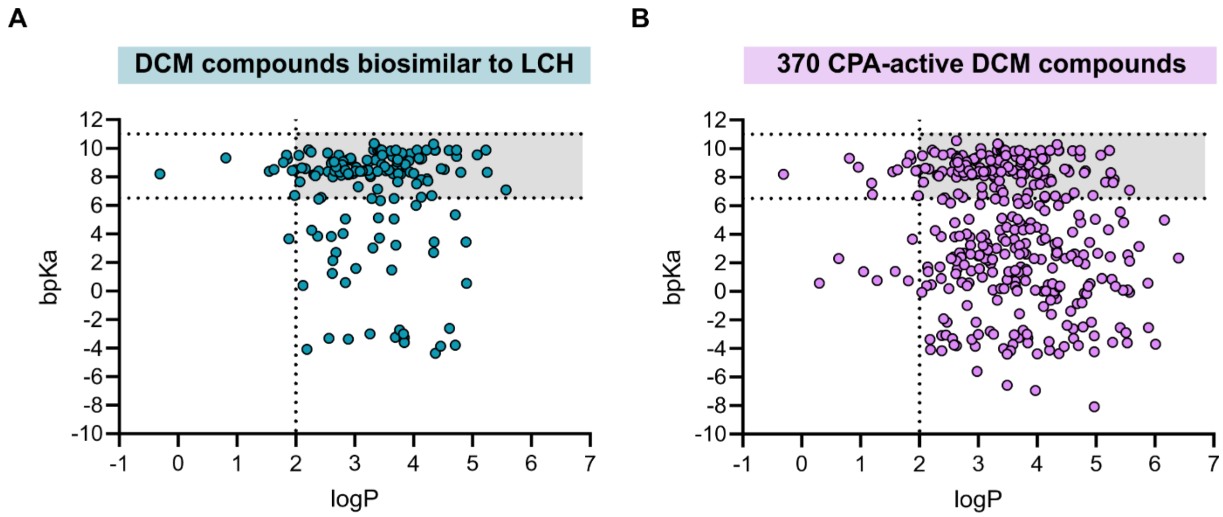

Figure S4 (related to Figure 1). Physicochemical properties for CPA-active DCM compounds. (A) Calculated logP and bpKa values for CPA-active DCM compounds whose profiles show highest similarity to the lysosomotropism/cholesterol homeostasis cluster (L/CH) and L/CH cluster biosimilarity ≥ 80%. (B) logP and bpKa values for the 370 DCM compounds that showed induction ≥ 5 % in the second biological replicate. The grey region corresponds to logP > 2 and bpKa between 6.5 and 11 and, thus, to properties of lysosomotropic compounds[1].

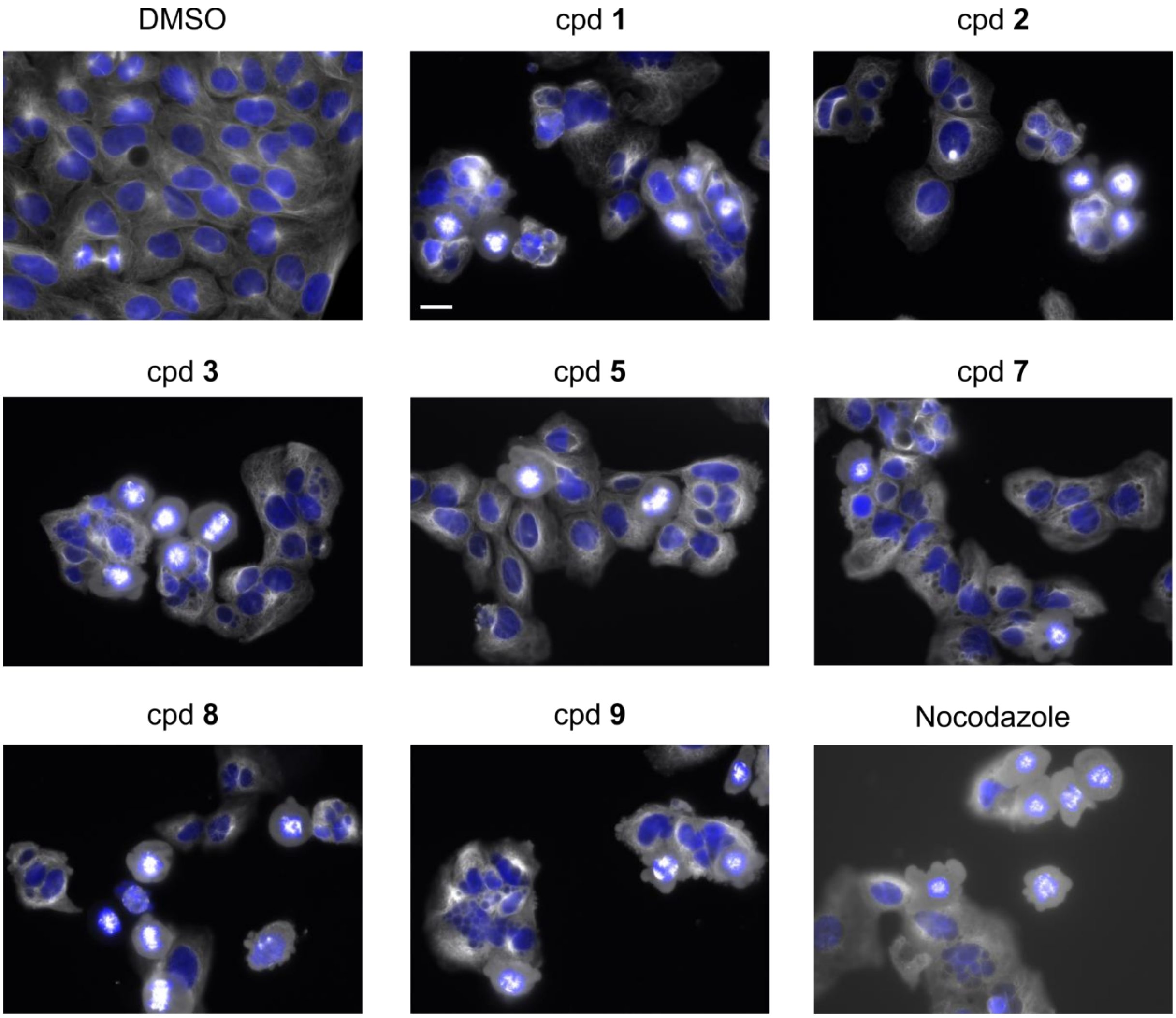

Figure S5 (related to Figure 2). Influence of compounds on the microtubule cytoskeleton. U2OS cells were treated with 30 µM of the compound or DMSO and nocodazole (0.1 µM) as controls for 24 h prior to staining with anti-tubulin antibody (white) or DAPI (blue) to visualize the DNA. Scale bar: 20 µm.

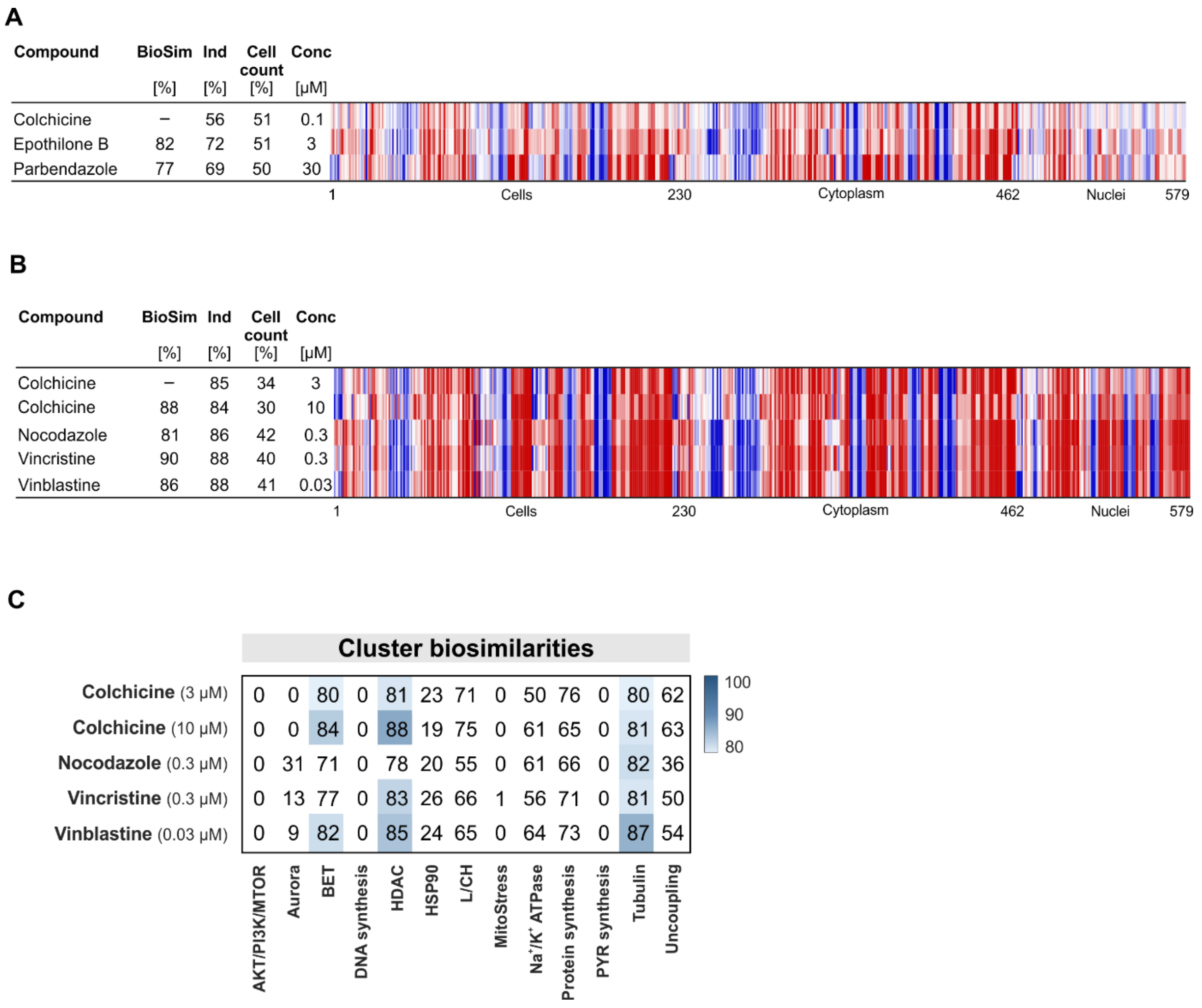

Figure S6 (related to Figure 3). CPA analysis for Colchicine. (A and B) Biosimilarity of colchicine to epothilone and parbendazole (A) or to nocodazole, vincristine and vinblastine at concentration with cell count < 50%. (B). The top line profile is set as a reference profile (100 % biological similarity, BioSim) to which the following profiles are compared. Blue color: decreased feature, red color: increased feature. BioSim: biosimilarity, Ind: induction, Conc: concentration. (C) Cluster biosimilarity heatmap for colchicine, nocodazole, vincristine and vinblastine at a concentration with cell count < 50%.

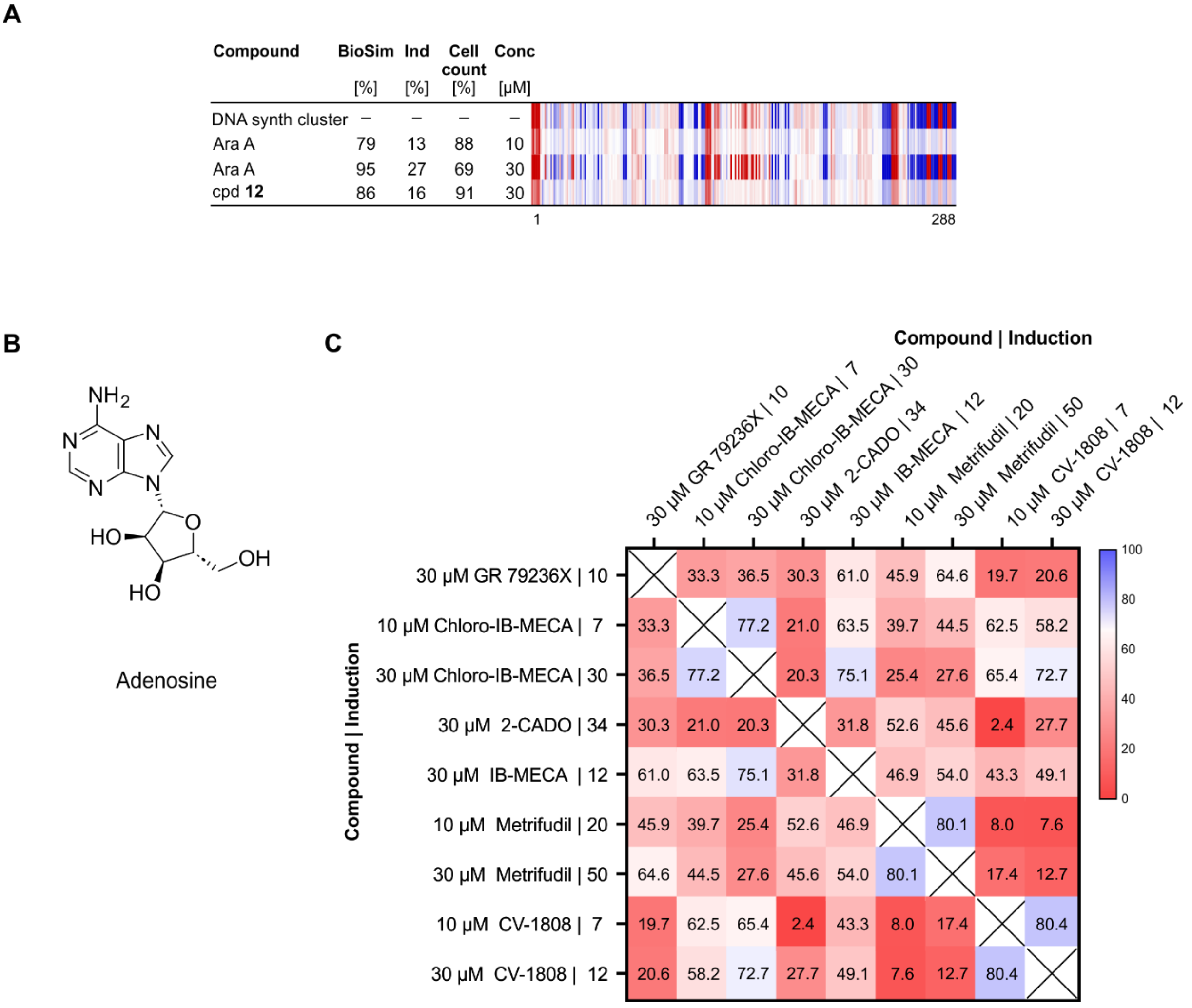

Figure S7 (related to Figure 4): DCM compound with similarity to the DNA synthesis cluster. (A) Biosimilarity to the DNA synthesis (synth) cluster profile. The top line profile is set as a reference profile (100 % biological similarity, BioSim) to which the following profiles are compared. Blue color: decreased feature, red color: increased feature. BioSim: biosimilarity, Ind: induction, Conc: concentration. (B) Structure of adenosine. (C) Compound profile cross-similarity for adenosine receptor agonist that are active in CPA. Induction is given in %.

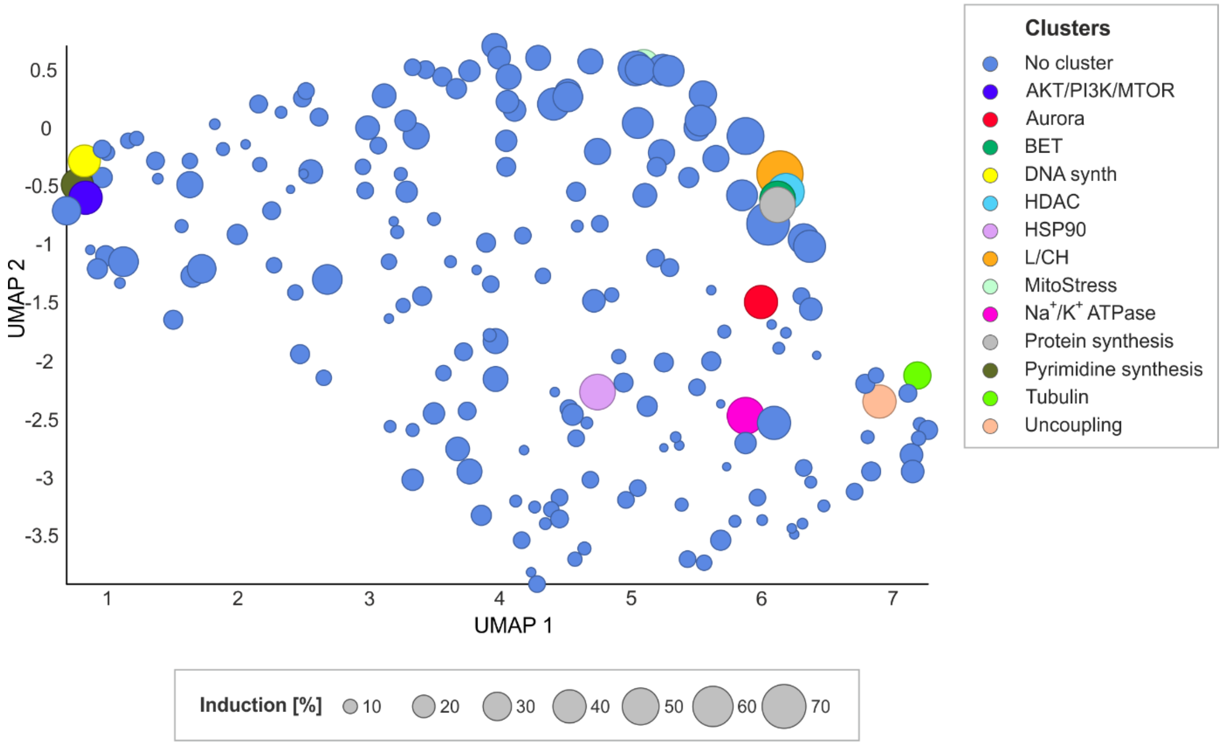

Figure S8: UMAP plot of the full profiles of the 182 CPA-active DCM compounds that could not be assigned to any biological cluster. Representative profiles from each of the 13 clusters are displayed as well. The circle size corresponds to the induction value. Generated using umap-learn (https://github.com/lmcinnes/umap), 15 neighbors, no normalization.

Figure S9. Hierarchical clustering for the 182 active DCM compounds whose profiles lack similarity to the 13 previously defined clusters. One representative compound profile of each of the 13 previously defined clusters is displayed as well. See separate PDF file.

Supporting Tables

Table S1: See separate XLS file

Table S2 (related to Figure 4): CPA induction values for adenosine receptor agonists.

Trivial name	Conc [μM]	Induction [%]	Cell_Count [%]	Known activity
Adenosine	10	0	100	A1, A2A, A2B, and A3 agonists
Adenosine	30	0.5	106	A1, A2A, A2B, and A3 agonists
2-CADO	10	1.7	93	adenosine receptor agonist with selectivity for A1 over A2
2-CADO	30	34.5	84	adenosine receptor agonist with selectivity for A1 over A2
2-CADO,2-Chloroadenosine, 6-Amino-2-chloropurine riboside	2	0	96	A1 adenosine receptor agonist
2-Phenylaminoadenosine, CV-1808	10	6.7	105	A2 adenosine receptor agonist
2-Phenylaminoadenosine, CV-1808	30	12.3	105	A2 adenosine receptor agonist
CGS 21680 hydrochloride	2	0.5	108	A2A adenosine receptor agonist
CHA	10	0	96	A1 adenosine receptor agonist
CHA	30	0	97	A1 adenosine receptor agonist
Chloro-IB-MECA	10	7.1	107	A3 adenosine receptor agonist
Chloro-IB-MECA	30	29.7	87	A3 adenosine receptor agonist
GR 79236X	10	0	105	A1 adenosine receptor agonist
GR 79236X	30	9.8	86	A1 adenosine receptor agonist
HEMADO	10	0.2	100	A3 adenosine receptor agonist
HEMADO	30	2.1	107	A3 adenosine receptor agonist
IB-MECA	10	0.5	103	A3 adenosine receptor agonist
IB-MECA	30	11.6	96	A3 adenosine receptor agonist
IB-MECA, CF 101	2	0	98	A3 adenosine receptor agonist
Metrifudil	10	20	88	adenosine receptor agonist
Metrifudil	30	49.9	103	adenosine receptor agonist
N6-Cyclohexyladenosine	2	0	104	A1 adenosine receptor agonist
N6-Cyclopentyladenosine	2	0	107	A1 adenosine receptor agonist
N6-Cyclopentyladenosine, CPA	10	0	102	A1 adenosine receptor agonist
N6-Cyclopentyladenosine, CPA	30	0.7	97	A1 adenosine receptor agonist
N6-Phenyladenosine	10	0.5	97	A1 adenosine receptor agonist
N6-Phenyladenosine	30	1	94	A1 adenosine receptor agonist
PSB-0777	2	0	100	A2A adenosine receptor agonist
R(-)-N6-(2-Phenylisopropyl)adenosine, R(-)-PIA	10	0	99	A1 adenosine receptor agonist
R(-)-N6-(2-Phenylisopropyl)adenosine, R(-)-PIA	30	0.2	87	A1 adenosine receptor agonist
R-PIA	2	0.2	100	A1 adenosine receptor agonist

Table S3 Related to Figure 5. DCM compounds that suppress Hedgehog-induced osteogenesis. IC_{50} values ± SD (n=3) for inhibition of Hedgehog-induced osteogenesis in C3H10T1/2 cells or GLI-dependent reporter gene assay in Shh-LIGHT2 cells (GLI RGA).

**Cpd**	**Structure**	**Osteogenesis inhibition IC_{50} ± SD**	**GLI RGA inhibition IC_{50} ± SD**
**14**	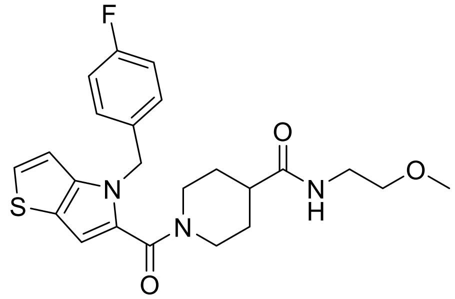	0.09 ± 0	0.47 ± 0.06
**15**	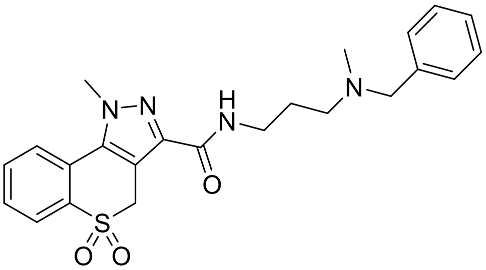	0.31 ± 0.02	2.17 ± 0.2
**16**	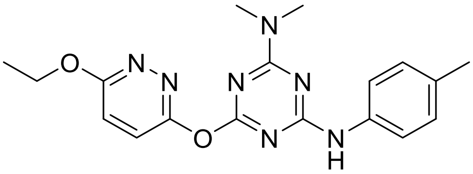	0.32 ± 0.2	
**17**	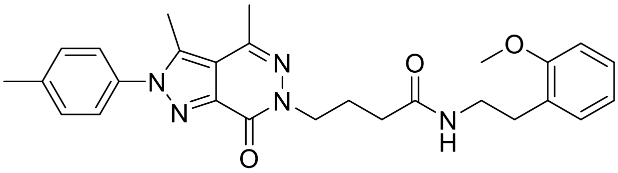	0.54 ± 0.02	1.40 ± 0.1
**18**	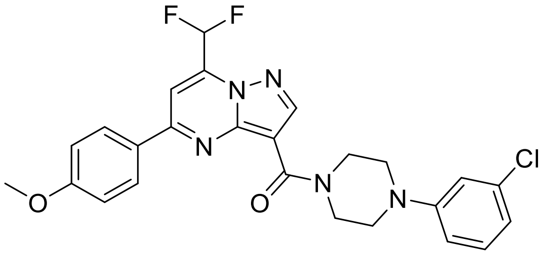	0.92 ± 0.29	1.51 ± 0.2
**19**	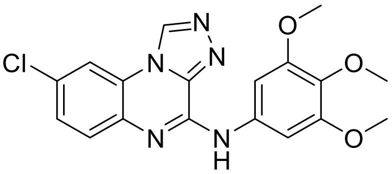	1.16 ± 0.1	1.02 ± 0.2

Cpd	Structure	Osteogenesis inhibition $IC_{50} \pm SD$	GLI RGA inhibition $IC_{50} \pm SD$
**20**	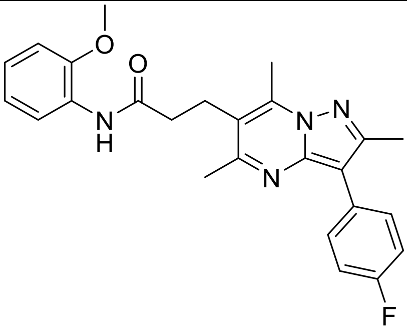	1.28 ± 0.4	6.90 ± 0.8
**21**	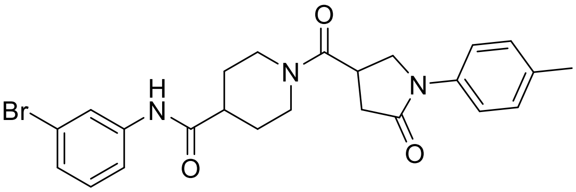	1.38 ± 0.3	7.99 ± 0.0
**22**	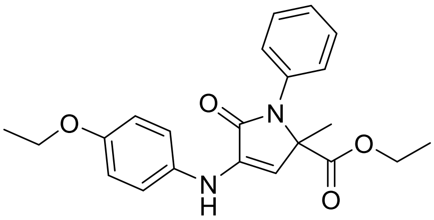	1.46 ± 0.4	
**23**	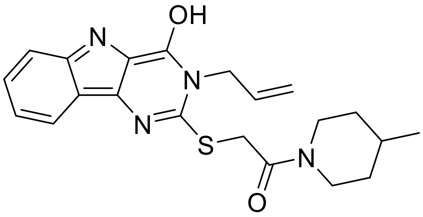	2.02 ± 0.3	
**24**	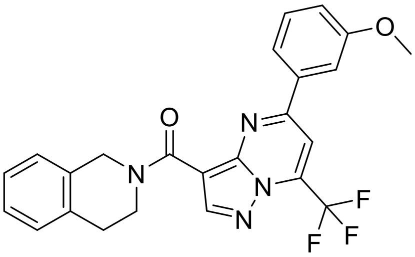	2.13 ± 0.3	
**25**	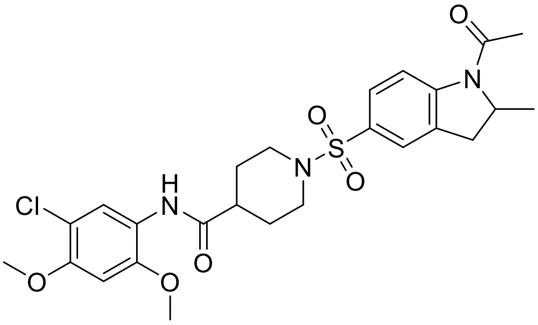	2.35 ± 0.2	

Cpd	Structure	Osteogenesis inhibition $IC_{50} \pm SD$	GLI RGA inhibition $IC_{50} \pm SD$
**26**	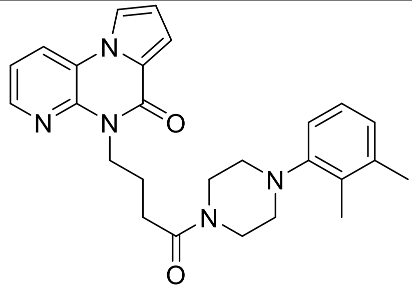	3.36 ± 0.2	
**27**	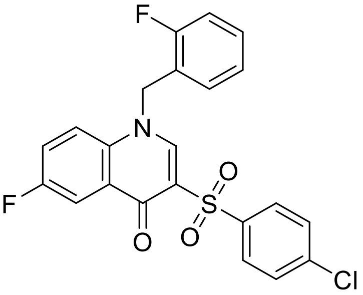	3.89 ± 0.6	
**28**	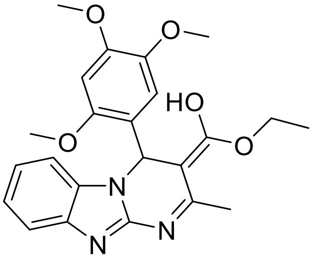	4.01 ± 0.6	
**29**	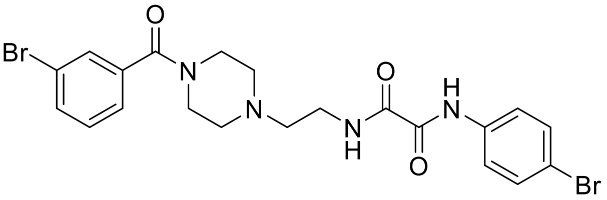	4.25 ± 0.4	
**30**	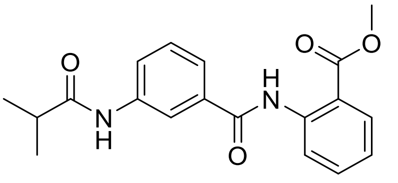	4.36 ± 1.3	
**31**	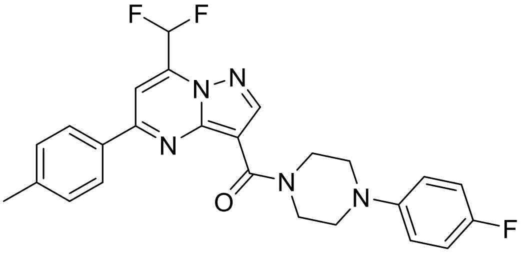	4.55 ± 1.4	
**32**	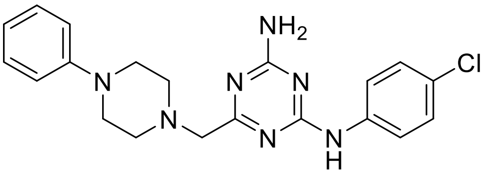	5.95 ± 0.6	

Cpd	Structure	Osteogenesis inhibition IC_{50} ± SD	GLI RGA inhibition IC_{50} ± SD
**33**	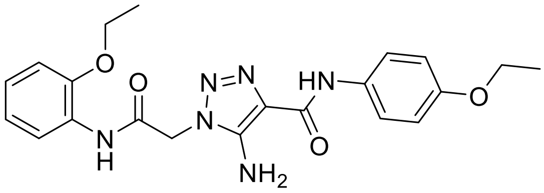	6.87 ± 1.3	
**34**	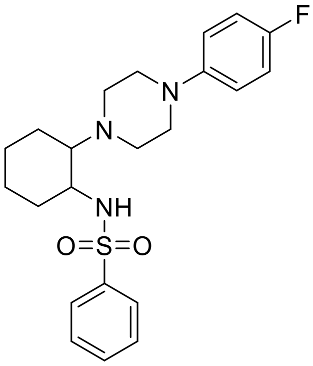	7.00 ± 0.6	

Table S4: Rhodanine-bearing DCM compounds tested in CPA. Ind: induction.

Structure	Ind [%]	Cell count [%]
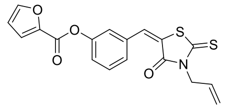	5	94
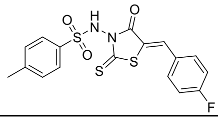	0.2	105
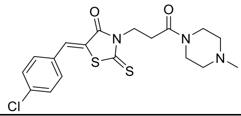	27.3	71
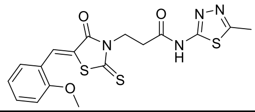	0.5	104
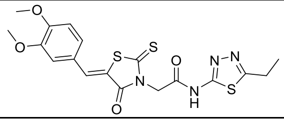	0.7	86
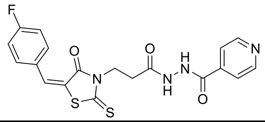	0	104
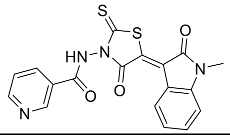	0.5	107
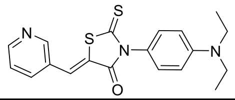	1.7	100
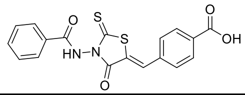	0.9	111
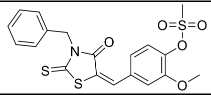	43.5	54
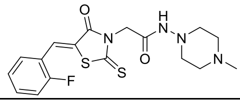	0	96

Structure	Ind [%]	Cell count [%]
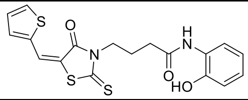	6.7	87
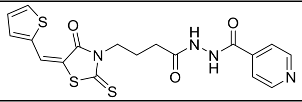	1	98
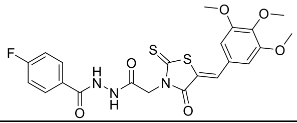	0	98
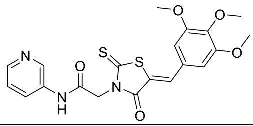	0.7	99
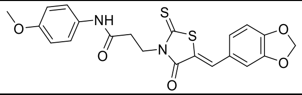	0.3	95
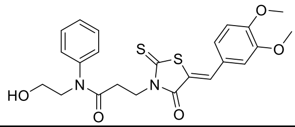	0.3	99
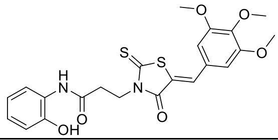	2.4	90
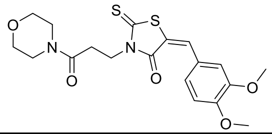	4.1	94
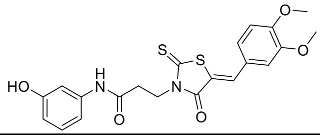	47.2	96
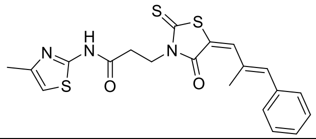	5.5	100

Table S5: Hydroxyphenyl hydrazone-bearing DCM compounds tested in CPA.

Structure	Induction [%]	Cell count [%]
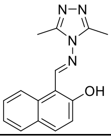	1.2	100
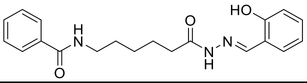	1.6	90
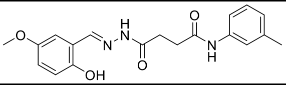	15.9	91
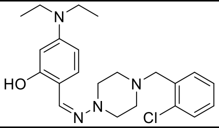	0.3	107
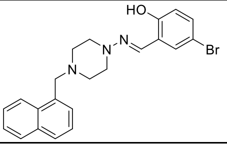	0.2	102
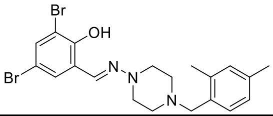	1.6	98

HPLC traces

HPLC Method

Samples were taken from the DMSO stock solutions and diluted 1:20 in H_2O : ACN (90:10; v/v). All used solvents were LC-MS Grade and in case of H_2O ultrapure. The measurement was carried out on an Ultra High Performance Liquid Chromatography (1290 Infinity II LC System; Agilent) system equipped with a filter-column (Ghost-Guard-LC 30 x 4.6 mm; MZ-Analysentechnik), a column (Poroshell 120 EC-C18, 1.9 µm, 2.1 x 50 mm; Agilent) and the corresponding pre-column (Poroshell 120 EC-C18, 3 x 5 mm, 2.7 µm; Agilent), column oven was set to 40 °C. Mobile phases were **A** (H_2O + 2.5 % FA (v/v)) and **B** (ACN + 2.5 %FA (v/v)). Chromatographic separation was performed with either a 4 min (10-90 % B in 4 min) or 6 min (0.0 - 0.3 min 10 % B, 0.3 - 6.0 min 10-90 % B) gradient with an injection volume of 0.1 µL and a flow rate of 0.5 mL/min. The eluents were monitored by DAD (G7117B; Agilent) in a wavelength range from 190 nm - 450 nm and then electro-sprayed (Dual AJS ESI; Agilent; parameters: VCap 3000 V, Nozzle Voltage 0 V, Fragmentor 125 V, Skimmer1 65 V, OctopoleRFPeak 750 V) into a TOF mass spectrometer (G6230B; Agilent). All MS spectra were acquired with the following parameters: positive polarity, m/z range 100 - 3000, Scan Rate 2 spectra/sec, Gas Temp 250 °C, Gas Flow 9.5 l/min, Nebulizer 40 psig, SheathGasTemp 300 °C, SheathGasFlow 10 l/min. A qualitative method was used to pre-evaluate the samples; small peaks that were not recognized by the integration algorithm were integrated manually (Software: MassHunter Workstation V.10; Agilent). Single Sample Reports were generated as PDFs (Software: MassHunter Analytical Studio Reviewer V. B.02.01)

HPLC traces

HPLC traces (**1**)

Analytical Studio Reviewer Report

Sample Name: 413653:01:03 **Acquired:** 7/21/2022 2:01 AM **Instrument:** Agilent TOF **Submitter:**
Location: 1,9:G,14 **Filename:** 1046303353-413653-01-03.d **User:** **Job Code:**

				Area %						Area Abs					
Peak #	Time	Target ...	Found	TIC(+)	TIC(+)	TIC(+)	DAD	UV254	Base ...	TIC(+)	TIC(+)	TIC(+)	DAD	UV254	Base Peak
1	1.821	333.1477	Yes	0.0	0.0	99.0	98.7	55.5	99.9	0	0	1.50E07	143.99	3.51	1.10E07
2	2.241	333.1477	NA	0.0	0.0	0.0	0.2	4.8	0.0	0	0	0	0.30	0.30	0
3	2.327	333.1477	NA	0.0	0.0	0.3	0.0	0.0	0.0	0	0	4.65E04	0	0	0
4	2.835	333.1477	NA	0.0	0.0	0.5	1.1	39.8	0.0	0	0	7.05E04	1.55	2.52	0
5	3.648	333.1477	NA	0.0	0.0	0.1	0.0	0.0	0.1	0	0	2.24E04	0	0	1.54E04
6	3.843	333.1477	NA	0.0	0.0	0.1	0.0	0.0	0.0	0	0	1.30E04	0	0	0

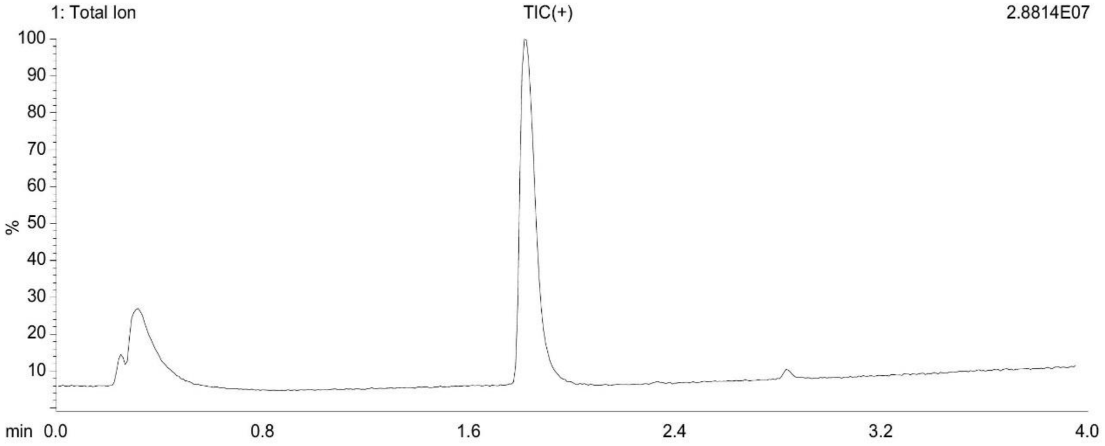

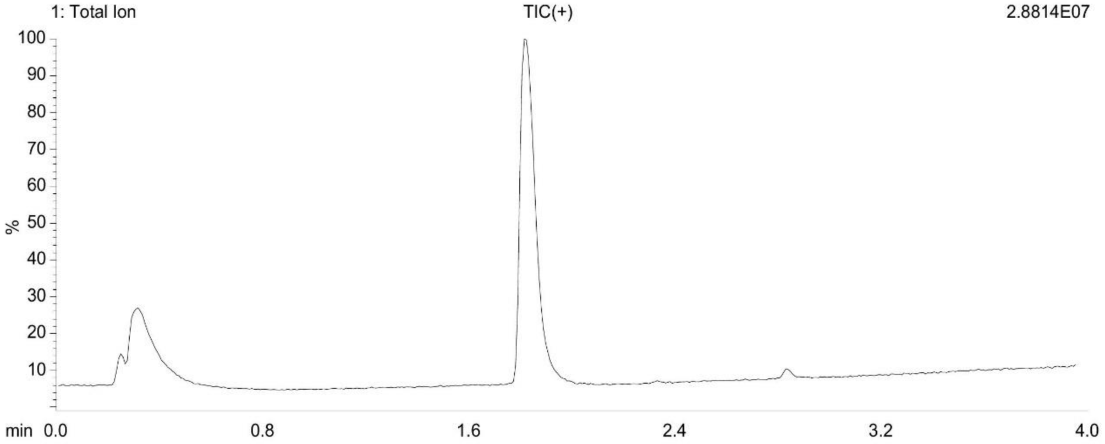

Page 1 of 5 Printed: 1/8/2024 3:19 PM

Analytical Studio Reviewer Report

Sample Name: 413653:01:03 **Acquired:** 7/21/2022 2:01 AM **Instrument:** Agilent TOF **Submitter:**
Location: 1,9:G,14 **Filename:** 1046303353-413653-01-03.d **User:** **Job Code:**

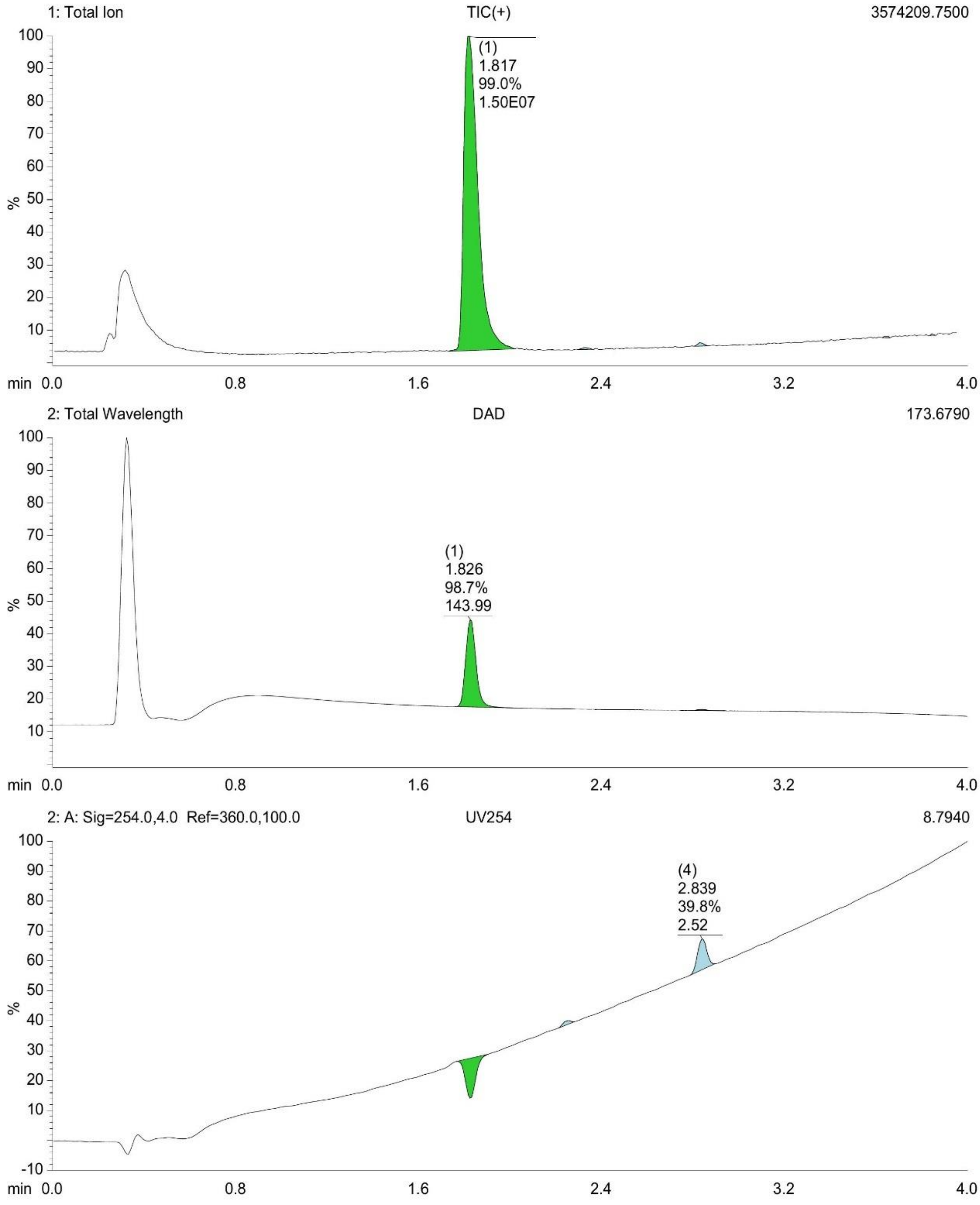

Page 2 of 5 Printed: 1/8/2024 3:19 PM

Analytical Studio Reviewer Report

Sample Name: 413653:01:03 **Acquired:** 7/21/2022 2:01 AM **Instrument:** Agilent TOF **Submitter:**
Location: 1,9:G,14 **Filename:** 1046303353-413653-01-03.d **User:** **Job Code:**

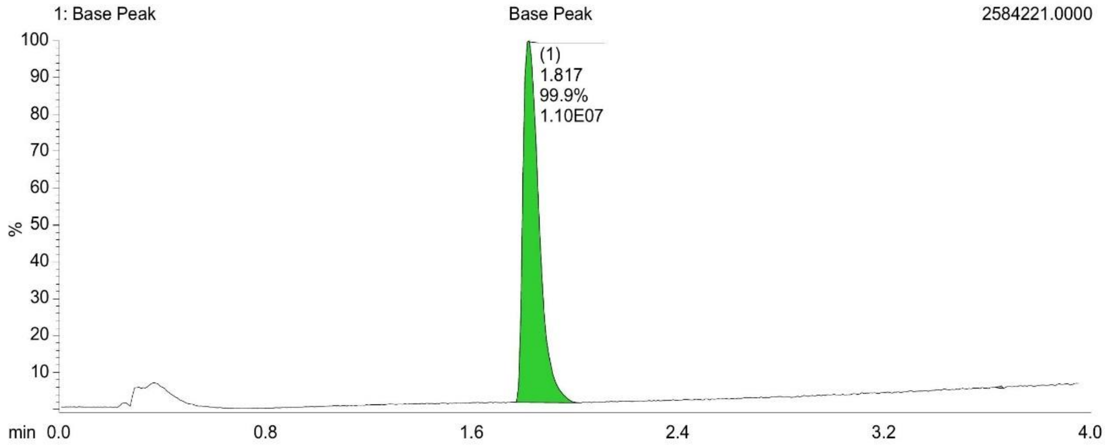

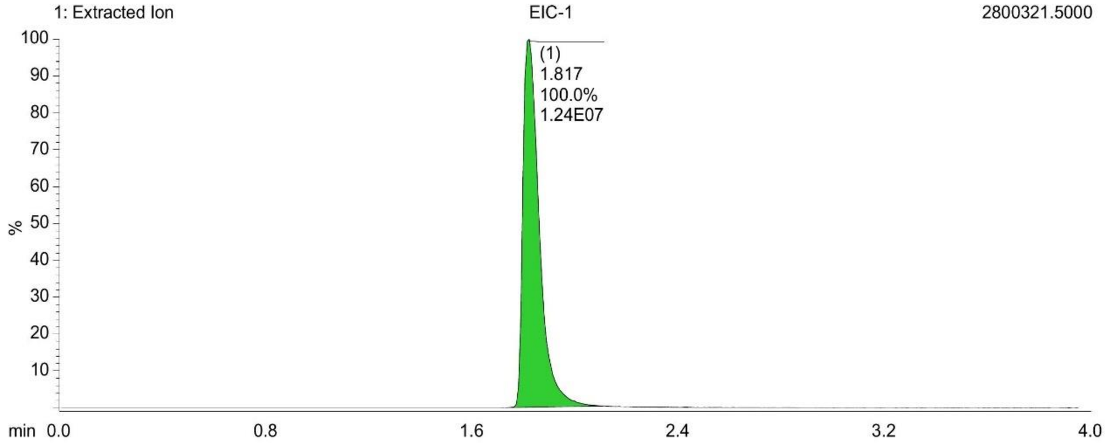

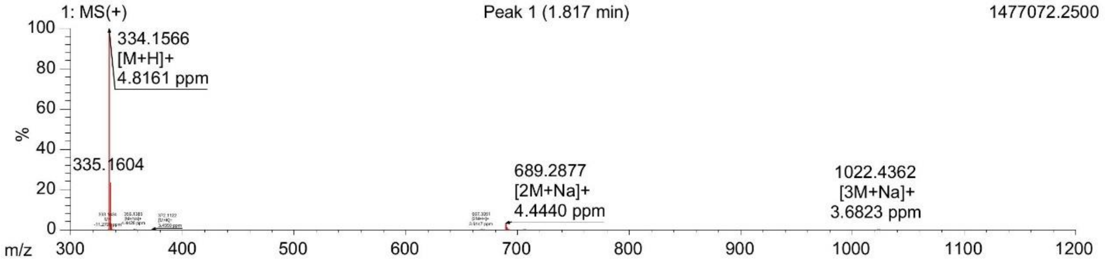

BPM	Error PPM	Error mDa	Target
334.1566	3.6823	3.7649	333.1…

HPLC traces (**2**)

Analytical Studio Reviewer Report

Sample Name: 190466:01:06 **Acquired:** 3/21/2022 5:04 PM **Instrument:** Agilent TOF **Submitter:**
Location: 1,3:M,4 **Filename:** 1046264779-190466-01-06.d **User:** **Job Code:**

				Area %						Area Abs					
Peak #	Time	Target ...	Found	TIC(+)	TIC(+)	TIC(+)	UV254	DAD	Base ...	TIC(+)	TIC(+)	TIC(+)	UV254	DAD	Base Peak
1	2.181	302.0725	Yes	0.0	0.0	0.0	0.0	0.0	0.0	0	0	0	0	0	0
2	2.394	302.0725	Yes	0.0	0.0	0.0	0.0	0.0	0.0	0	0	0	0	0	0
3	2.743	302.0725	NA	0.0	0.0	0.4	0.0	0.0	0.2	0	0	9.70E05	0	0	2.60E05
4	2.917	302.0725	NA	0.0	0.0	0.0	0.0	0.0	0.0	0	0	0	0.37	0.34	0
5	3.028	302.0725	Yes	0.0	0.0	0.0	0.0	0.0	0.0	0	0	0	0	0	0
6	3.154	302.0725	NA	0.0	0.0	0.0	0.0	0.1	0.0	0	0	0	0.23	1.08	0
7	3.358	302.0725	NA	0.0	0.0	0.4	0.0	0.0	0.3	0	0	9.01E05	0.31	0.24	5.04E05
8	3.681	302.0725	NA	0.0	0.0	2.1	0.0	0.1	1.4	0	0	4.67E06	0.27	0.74	2.10E06
9	3.780	302.0725	Yes	0.0	0.0	82.6	99.2	98.6	0.0	0	0	1.80E08	819.52	832.34	0
10	3.849	302.0725	NA	0.0	0.0	0.0	0.0	0.0	89.7	0	0	0	0	0	1.40E08
11	4.324	302.0725	Yes	0.0	0.0	2.5	0.2	0.0	2.0	0	0	5.35E06	1.28	0	3.09E06
12	4.427	302.0725	NA	0.0	0.0	10.0	0.3	0.9	5.6	0	0	2.19E07	2.38	7.67	8.73E06
13	4.529	302.0725	NA	0.0	0.0	0.0	0.0	0.1	0.0	0	0	0	0	0.85	0
14	4.772	302.0725	NA	0.0	0.0	1.9	0.2	0.2	0.8	0	0	4.16E06	1.51	1.31	1.32E06

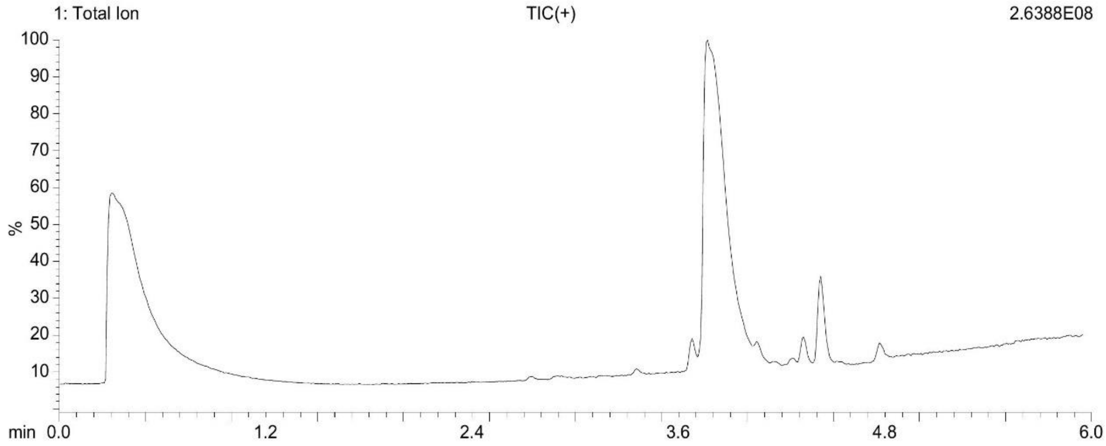

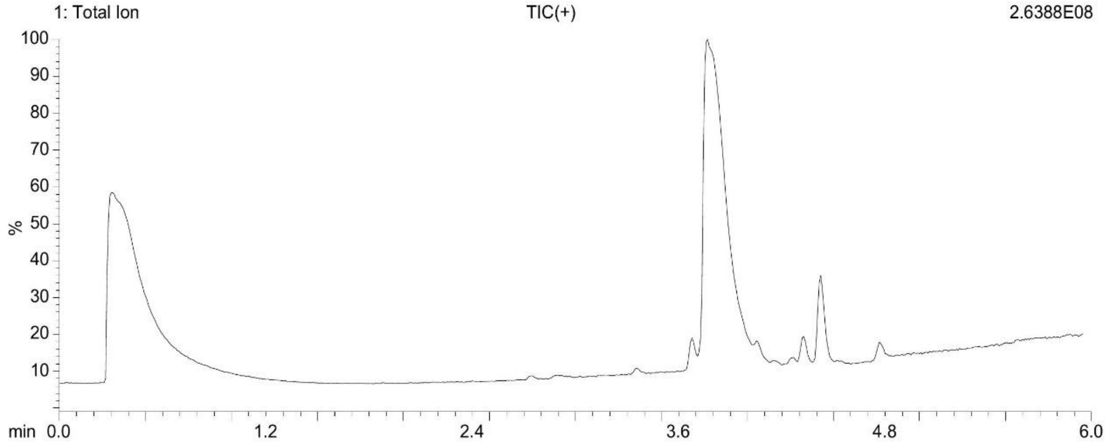

 Printed: 1/8/2024 3:19 PM

Analytical Studio Reviewer Report

Sample Name: 190466:01:06 **Acquired:** 3/21/2022 5:04 PM **Instrument:** Agilent TOF **Submitter:**
Location: 1,3:M,4 **Filename:** 1046264779-190466-01-06.d **User:** **Job Code:**

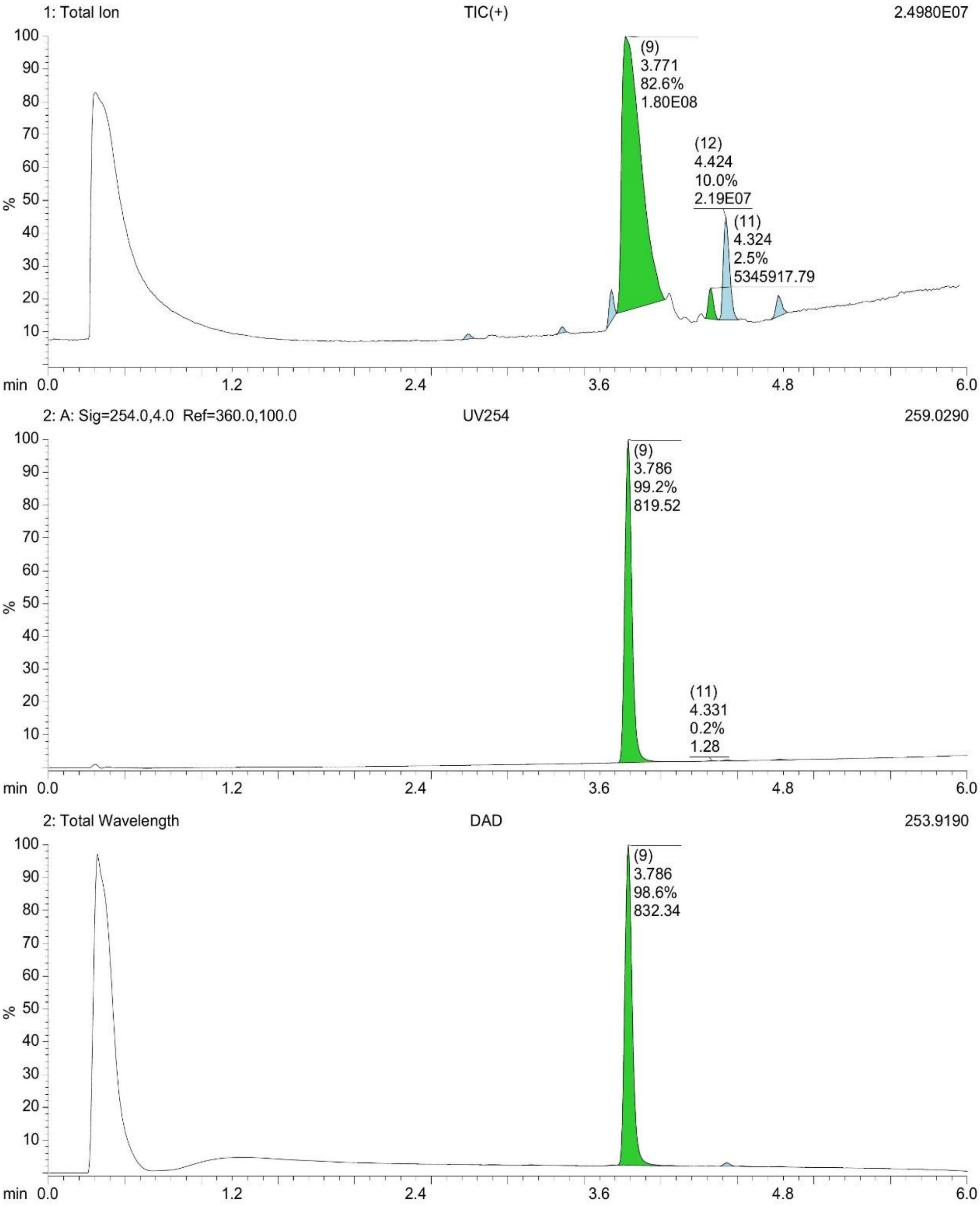

 Printed: 1/8/2024 3:19 PM

Analytical Studio Reviewer Report

**Sample Name:** 190466:01:06	**Acquired:** 3/21/2022 5:04 PM	**Instrument:** Agilent TOF	**Submitter:**
**Location:** 1,3:M,4	**Filename:** 1046264779-190466-01-06.d	**User:**	**Job Code:**

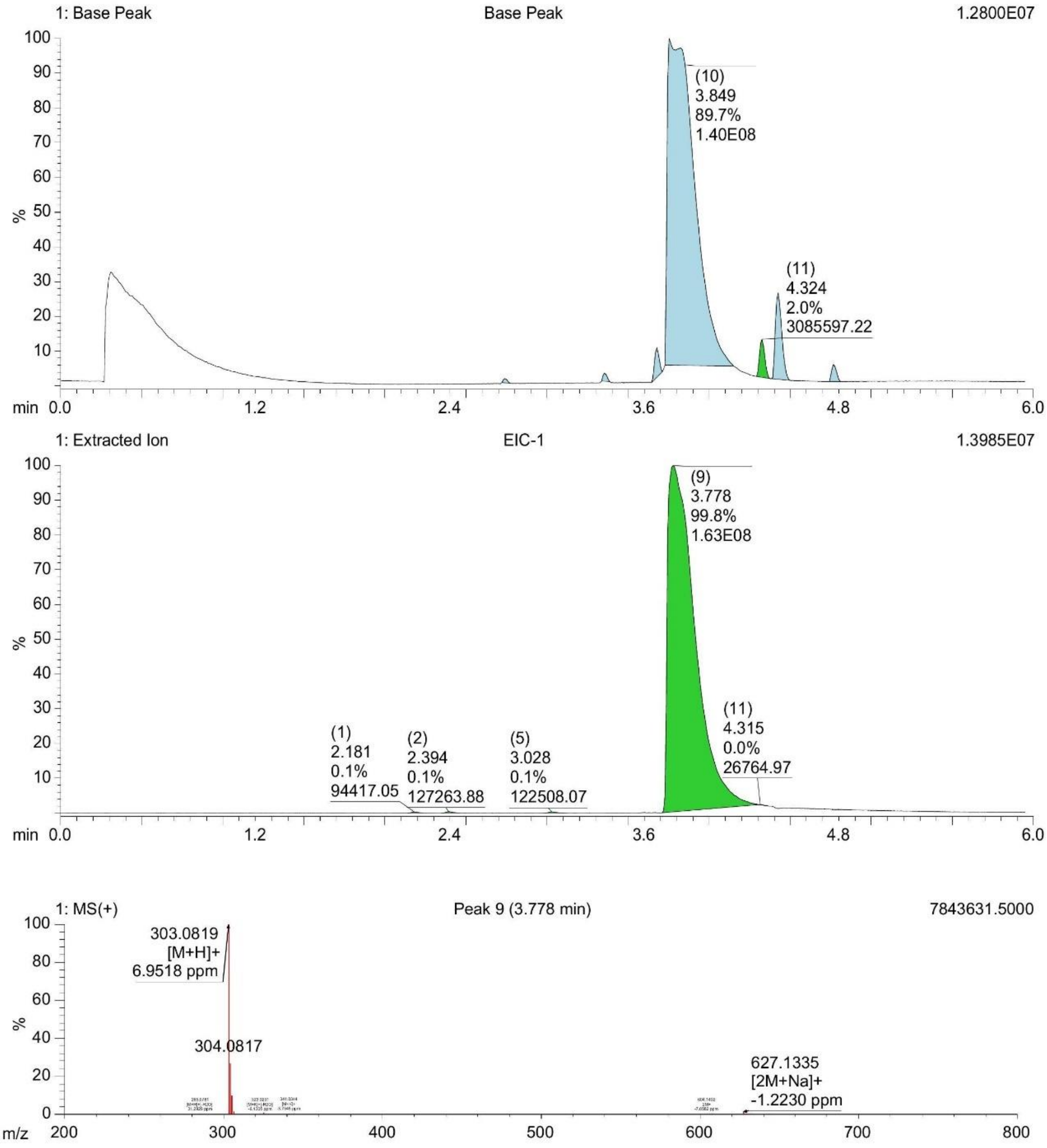

BPM	Error PPM	Error mDa	Target
303.0819	4.8007	3.0874	285.0…

HPLC traces (**3**)

Analytical Studio Reviewer Report

Sample Name: 415151:01:03 **Acquired:** 7/21/2022 3:59 AM **Instrument:** Agilent TOF **Submitter:**
Location: 1,9:I,3 **Filename:** 1046303417-415151-01-03.d **User:** **Job Code:**

				Area %						Area Abs					
Peak #	Time	Target ...	Found	TIC(+)	TIC(+)	TIC(+)	DAD	UV254	Base ...	TIC(+)	TIC(+)	TIC(+)	DAD	UV254	Base Peak
1	2.766	339.1107	Yes	0.0	0.0	82.3	96.7	10.1	0.0	0	0	384756.64	9.90	0.20	0
2	2.834	339.1107	NA	0.0	0.0	10.5	0.0	89.9	0.0	0	0	49152.27	0	1.77	0
3	2.945	339.1107	NA	0.0	0.0	0.0	3.3	0.0	0.0	0	0	0	0.33	0	0
4	3.458	339.1107	NA	0.0	0.0	3.5	0.0	0.0	0.0	0	0	16138.23	0	0	0
5	3.577	339.1107	NA	0.0	0.0	2.0	0.0	0.0	100.0	0	0	9404.00	0	0	11407.18
6	3.924	339.1107	NA	0.0	0.0	1.7	0.0	0.0	0.0	0	0	8053.24	0	0	0

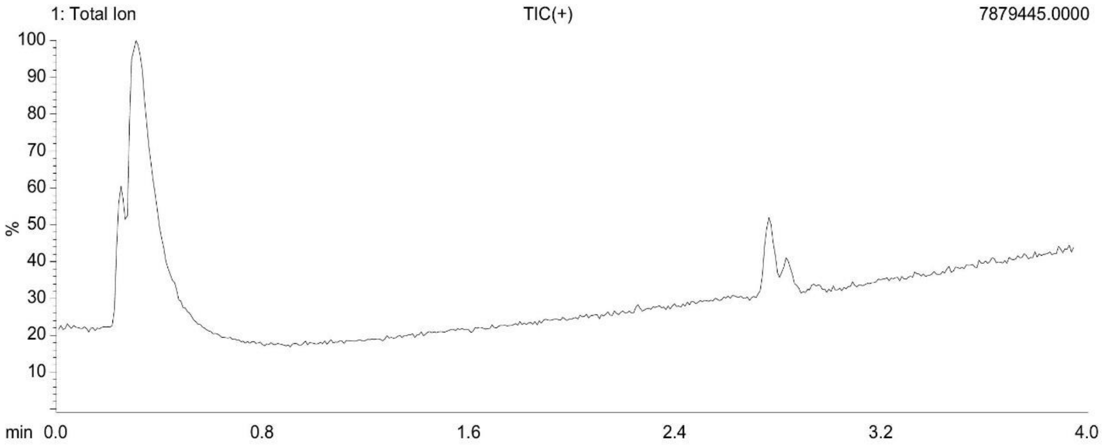

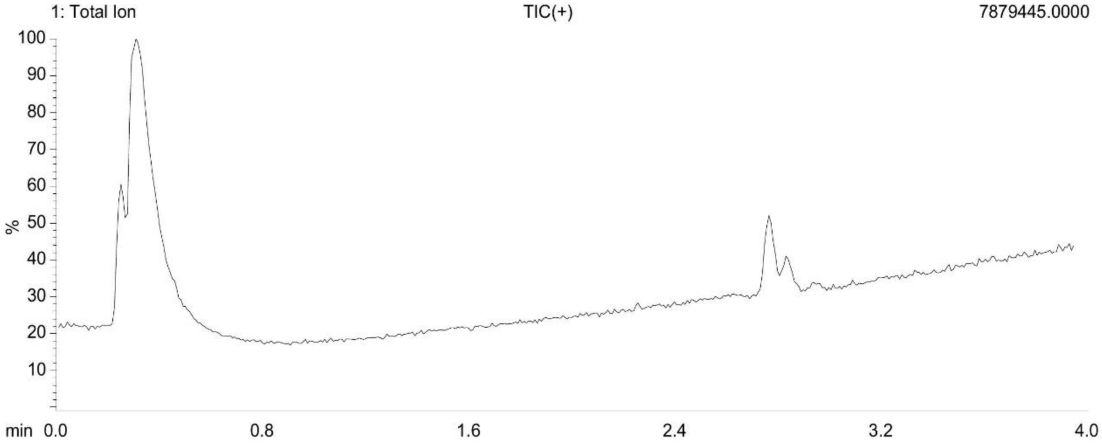

 Printed: 1/8/2024 3:19 PM

Analytical Studio Reviewer Report

Sample Name: 415151:01:03 **Acquired:** 7/21/2022 3:59 AM **Instrument:** Agilent TOF **Submitter:**
Location: 1,9:I,3 **Filename:** 1046303417-415151-01-03.d **User:** **Job Code:**

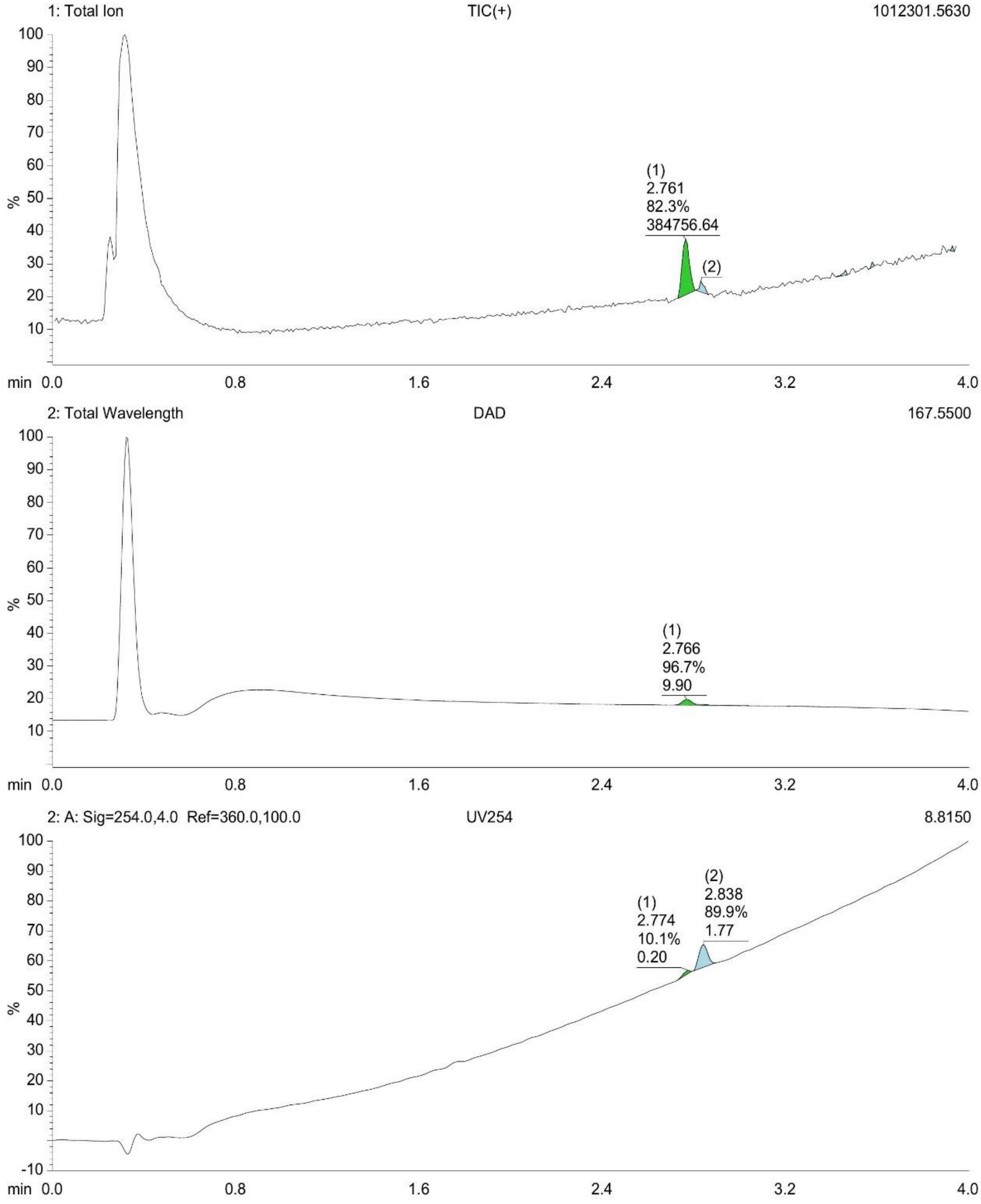

 Printed: 1/8/2024 3:19 PM

Analytical Studio Reviewer Report

Sample Name: 415151:01:03 **Acquired:** 7/21/2022 3:59 AM **Instrument:** Agilent TOF **Submitter:**
Location: 1,9:I,3 **Filename:** 1046303417-415151-01-03.d **User:** **Job Code:**

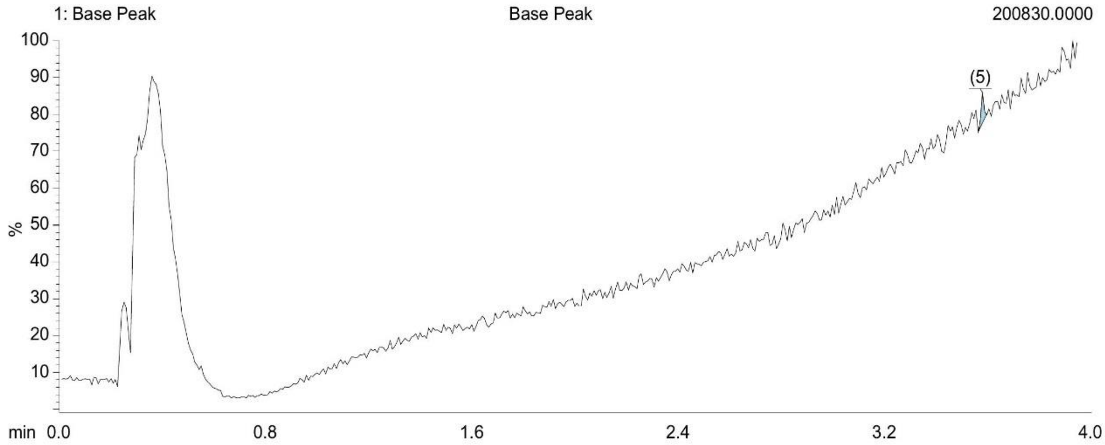

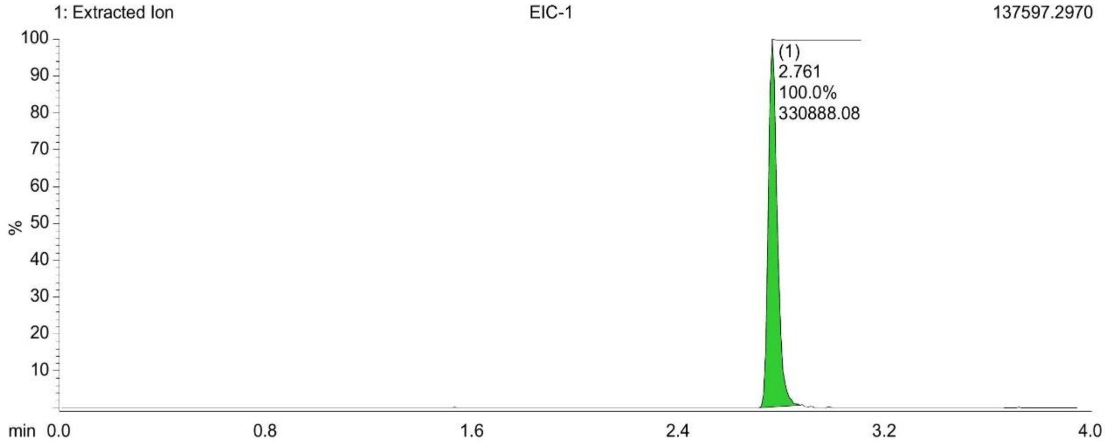

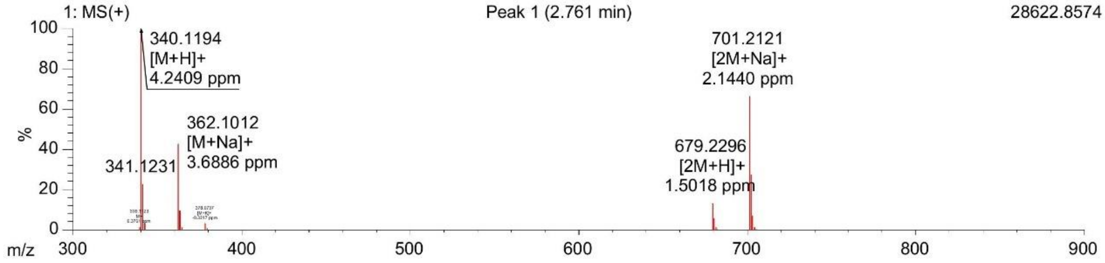

BPM	Error PPM	Error mDa	Target
340.1194	2.1440	1.5034	339.1…

HPLC traces (**4**)

Analytical Studio Reviewer Report

Sample Name: 417969:01:01 **Acquired:** 5/31/2023 1:08 AM **Instrument:** Agilent TOF **Submitter:**
Location: 1,1:O,1 **Filename:** 1046184166-417969-01-01.d **User:** **Job Code:**

				Area %						Area Abs					
Peak #	Time	Target ...	Found	TIC(+)	TIC(+)	TIC(+)	UV254	DAD	Base ...	TIC(+)	TIC(+)	TIC(+)	UV254	DAD	Base Peak
1	3.315	493.1883	Yes	0.0	0.0	3.8	0.9	1.1	3.7	0	0	2.76E06	1.17	0.88	1.67E06
2	3.473	493.1883	Yes	0.0	0.0	95.9	99.1	98.9	96.3	0	0	6.90E07	122.98	77.55	4.39E07
3	3.805	493.1883	NA	0.0	0.0	0.1	0.0	0.0	0.0	0	0	8.67E04	0	0	0
4	4.037	493.1883	NA	0.0	0.0	0.1	0.0	0.0	0.0	0	0	5.46E04	0	0	0
5	5.751	493.1883	NA	0.0	0.0	0.1	0.0	0.0	0.0	0	0	3.67E04	0	0	0

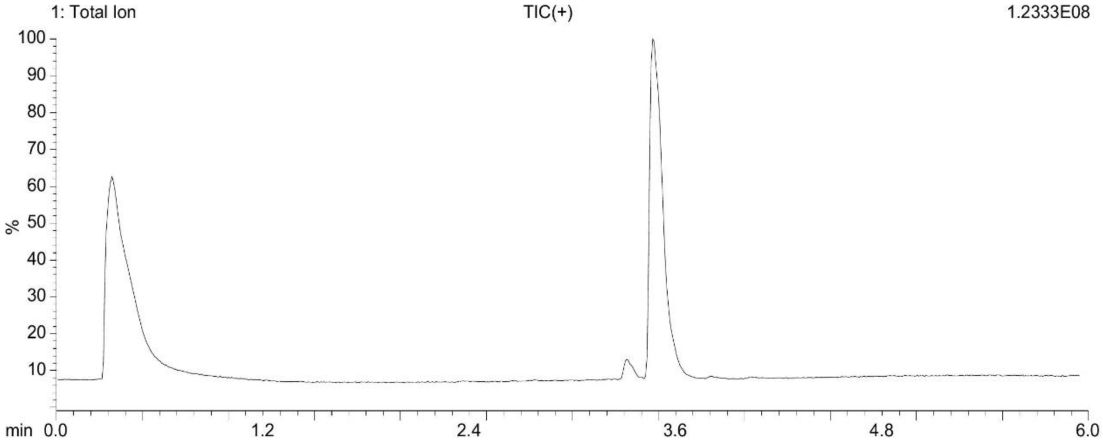

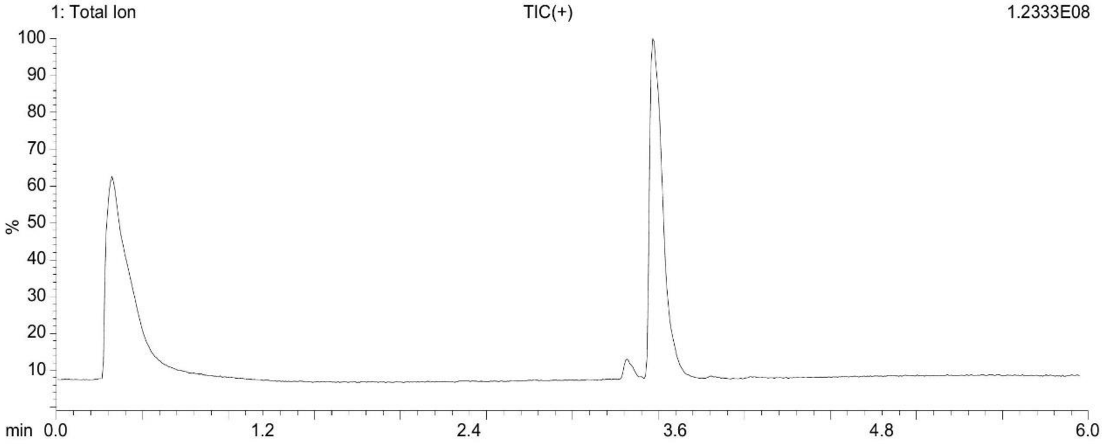

 Printed: 1/8/2024 3:18 PM

Analytical Studio Reviewer Report

Sample Name: 417969:01:01 **Acquired:** 5/31/2023 1:08 AM **Instrument:** Agilent TOF **Submitter:**
Location: 1,1:O,1 **Filename:** 1046184166-417969-01-01.d **User:** **Job Code:**

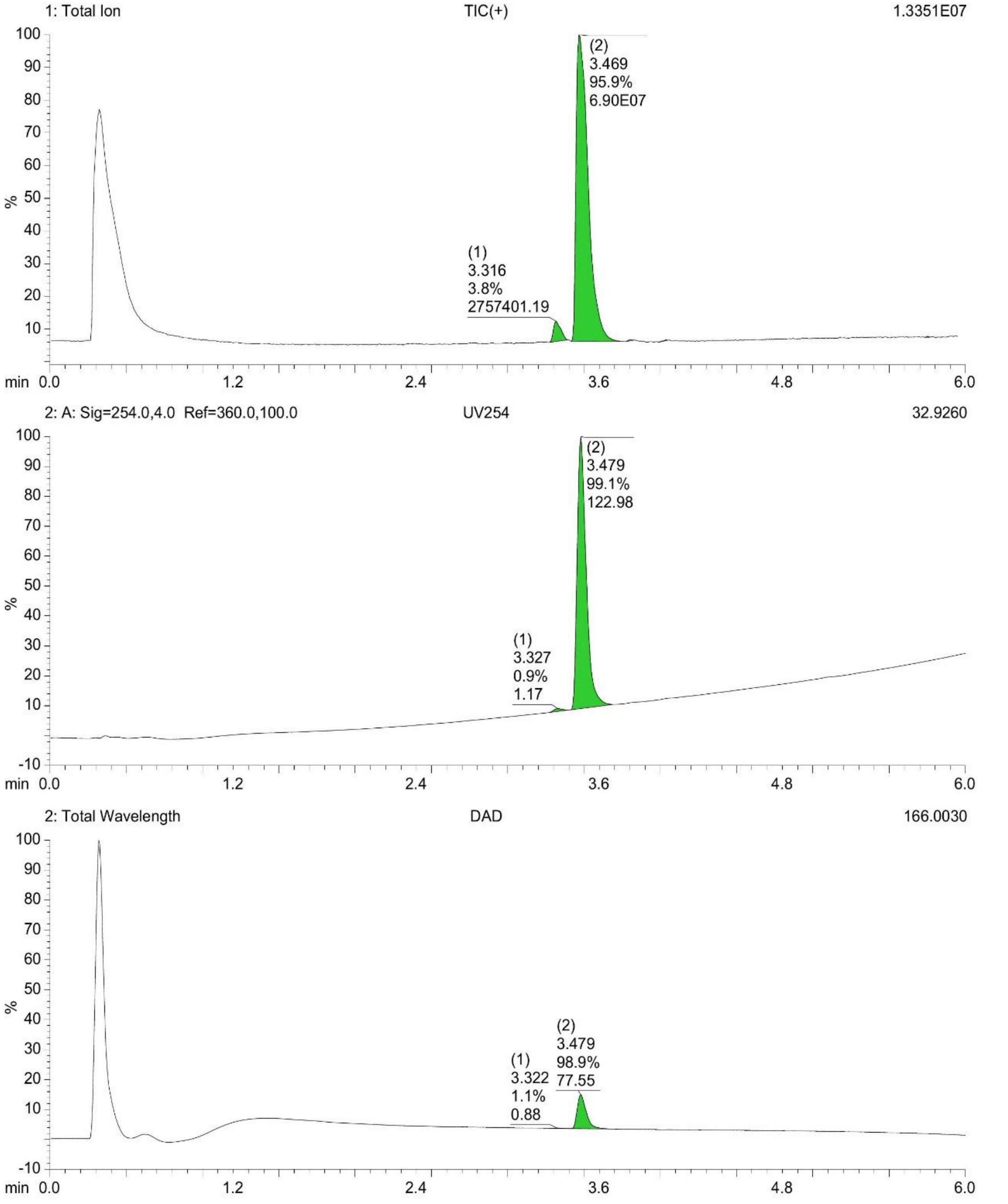

Page 2 of 5 Printed: 1/8/2024 3:18 PM

Analytical Studio Reviewer Report

Sample Name: 417969:01:01 **Acquired:** 5/31/2023 1:08 AM **Instrument:** Agilent TOF **Submitter:**
Location: 1,1:O,1 **Filename:** 1046184166-417969-01-01.d **User:** **Job Code:**

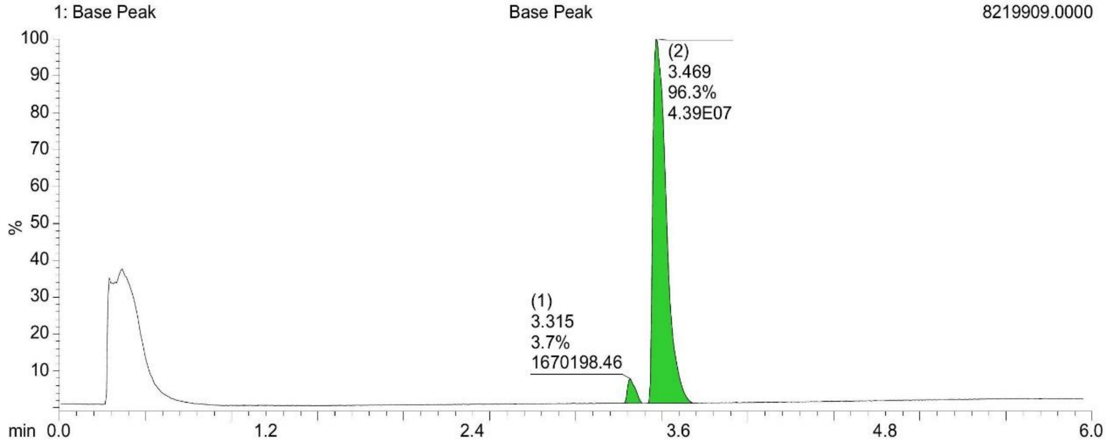

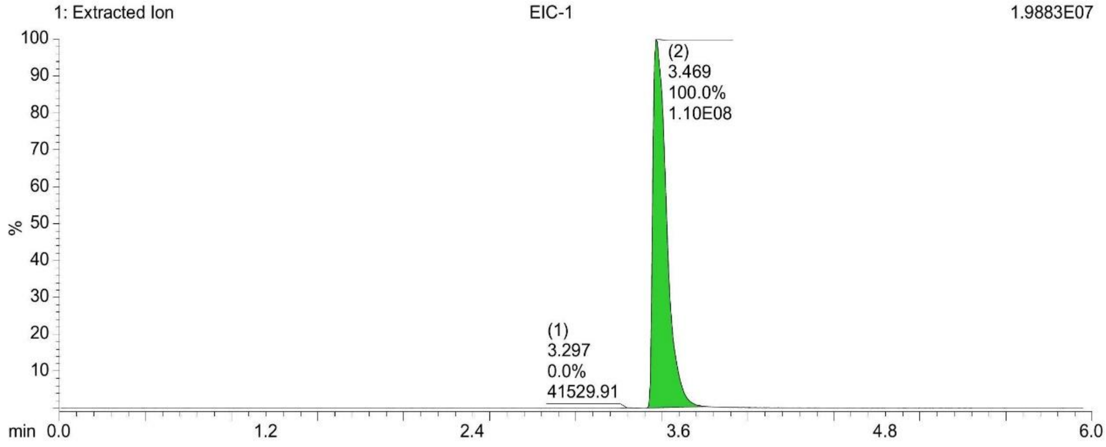

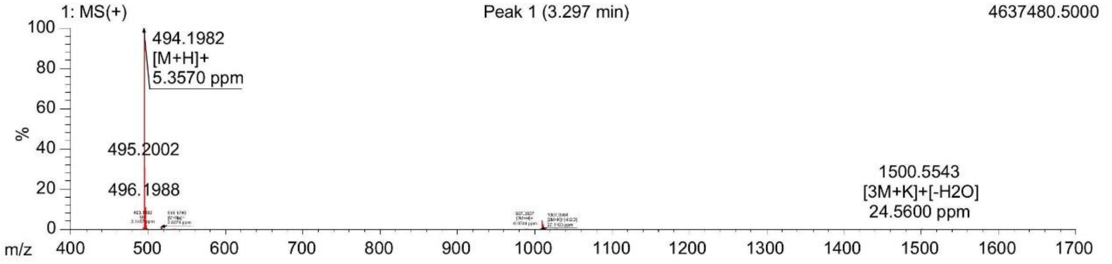

BPM	Error PPM	Error mDa	Target
494.1982	24.5600	36.8527	493.1…

Analytical Studio Reviewer Report

Sample Name: 417969:01:01 **Acquired:** 5/31/2023 1:08 AM **Instrument:** Agilent TOF **Submitter:**
Location: 1,1:O,1 **Filename:** 1046184166-417969-01-01.d **User:** **Job Code:**

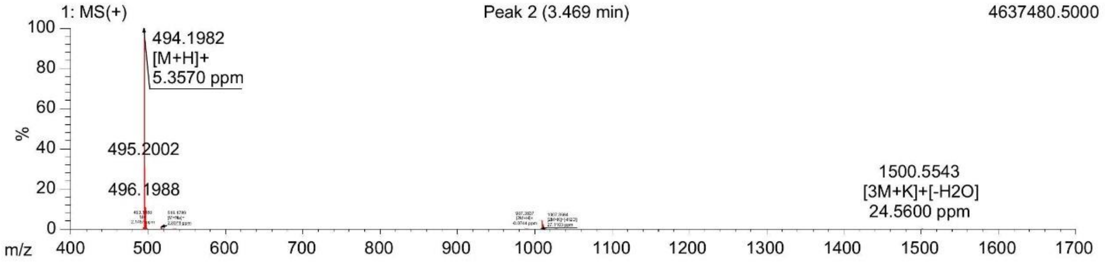

BPM	Error PPM	Error mDa	Target
494.1982	24.5600	36.8527	493.1…

HPLC traces (**5**)

Analytical Studio Reviewer Report

Sample Name: 414518:01:01 **Acquired:** 5/30/2023 11:52 PM **Instrument:** Agilent TOF **Submitter:**
Location: 1,1:C,3 **Filename:** 1046304670-414518-01-01.d **User:** **Job Code:**

				Area %						Area Abs					
Peak #	Time	Target ...	Found	TIC(+)	TIC(+)	TIC(+)	DAD	UV254	Base ...	TIC(+)	TIC(+)	TIC(+)	DAD	UV254	Base Peak
1	1.690	420.0143	NA	0.0	0.0	0.5	0.2	0.0	0.0	0	0	6.07E05	1.29	0	0
2	3.429	420.0143	NA	0.0	0.0	2.6	0.3	0.6	0.0	0	0	2.93E06	1.76	0.85	0
3	4.748	420.0143	NA	0.0	0.0	0.8	0.0	0.0	1.6	0	0	8.71E05	0	0	3.60E05
4	4.804	420.0143	Yes	0.0	0.0	0.0	0.0	0.0	98.4	0	0	0	0	0	2.28E07
5	4.814	420.0143	Yes	0.0	0.0	0.0	99.5	99.4	0.0	0	0	0	625.85	141.81	0
6	4.843	420.0143	Yes	0.0	0.0	96.1	0.0	0.0	0.0	0	0	1.08E08	0	0	0
7	5.267	420.0143	Yes	0.0	0.0	0.0	0.0	0.0	0.0	0	0	0	0	0	0

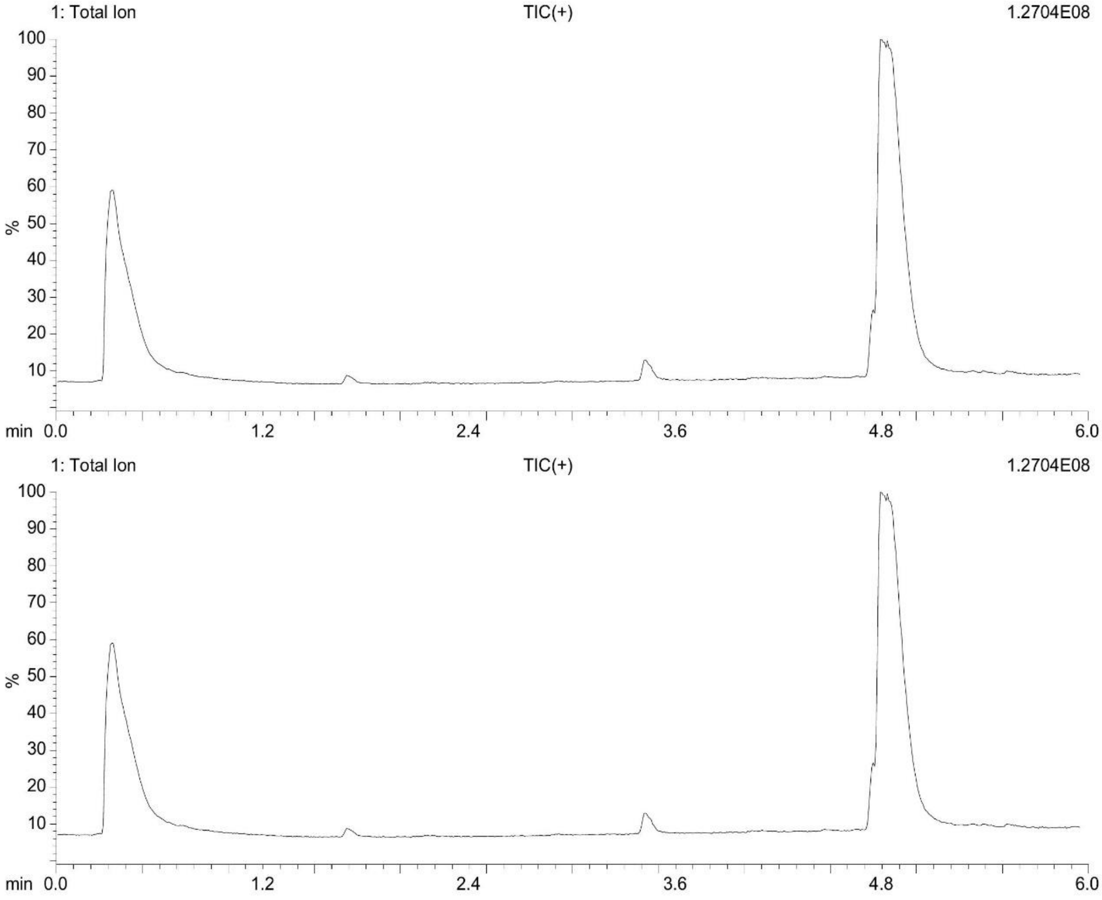

 Printed: 1/8/2024 3:18 PM

Analytical Studio Reviewer Report

Sample Name: 414518:01:01 **Acquired:** 5/30/2023 11:52 PM **Instrument:** Agilent TOF **Submitter:**
Location: 1,1:C,3 **Filename:** 1046304670-414518-01-01.d **User:** **Job Code:**

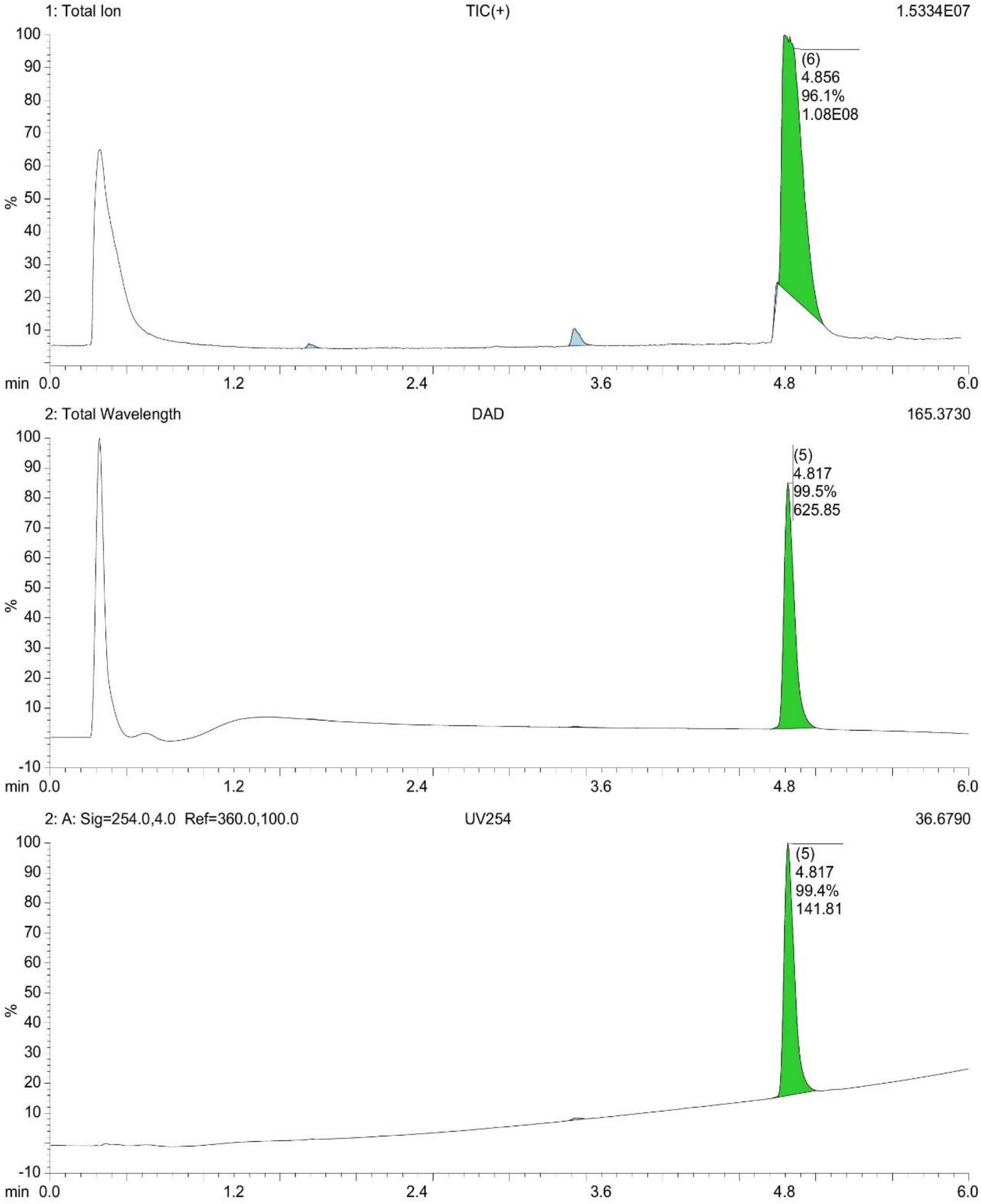

 Printed: 1/8/2024 3:18 PM

Analytical Studio Reviewer Report

Sample Name: 414518:01:01
Location: 1,1:C,3
Acquired: 5/30/2023 11:52 PM
Filename: 1046304670-414518-01-01.d
Instrument: Agilent TOF
User:
Submitter:
Job Code:

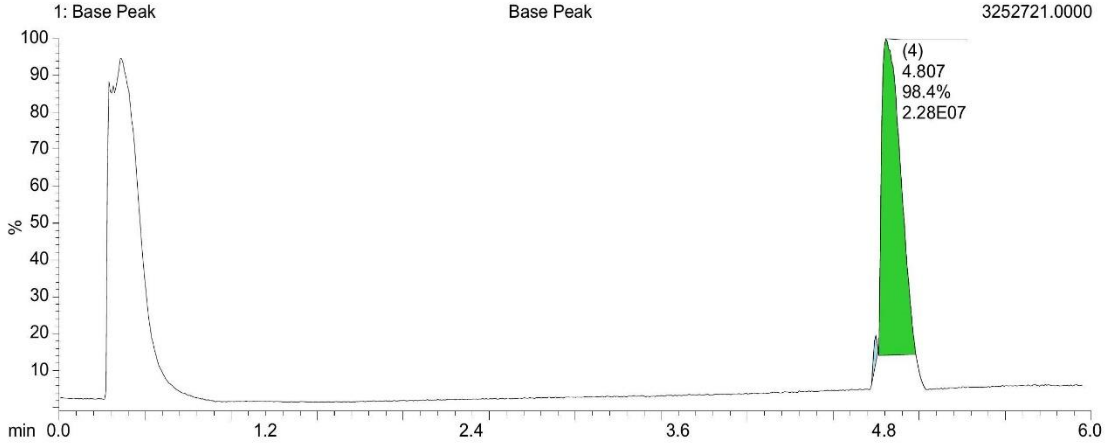

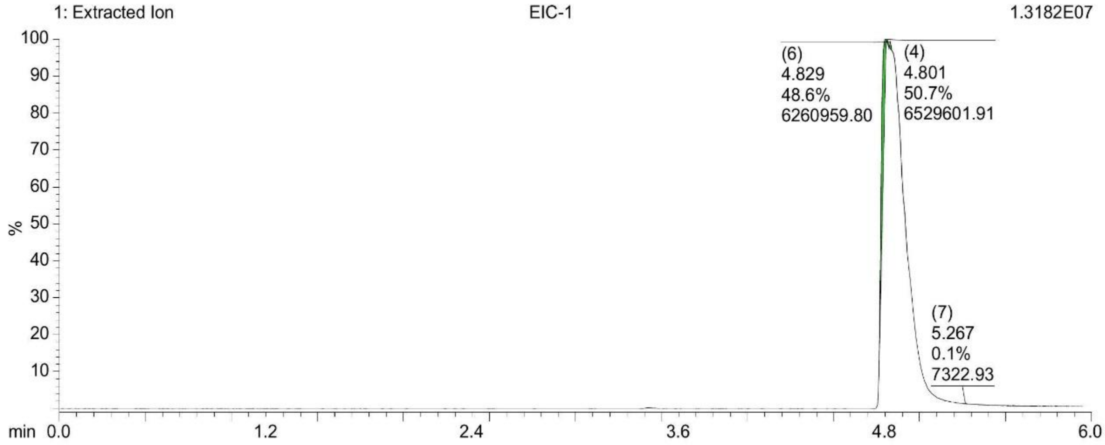

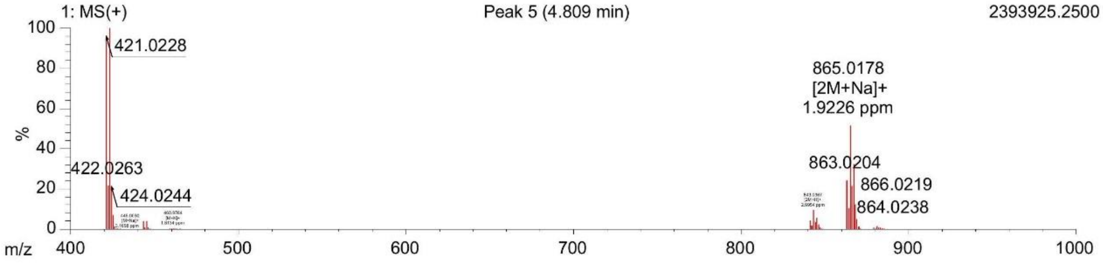

BPM	Error PPM	Error mDa	Target
423.0209	0.7933	0.6989	423.0…

HPLC traces (**6**)

Analytical Studio Reviewer Report

Sample Name: 415349:01:01 **Acquired:** 5/31/2023 12:58 AM **Instrument:** Agilent TOF **Submitter:**
Location: 1,1:M,1 **Filename:** 1046304843-415349-01-01.d **User:** **Job Code:**

				Area %						Area Abs					
Peak #	Time	Target ...	Found	TIC(+)	TIC(+)	TIC(+)	DAD	UV254	Base ...	TIC(+)	TIC(+)	TIC(+)	DAD	UV254	Base Peak
1	1.982	458.1510	NA	0.0	0.0	0.1	0.0	0.0	0.0	0	0	5.14E04	0	0	0
2	2.357	458.1510	Yes	0.0	0.0	99.8	100.0	100.0	100.0	0	0	7.52E07	164.19	31.63	2.11E07
3	4.608	458.1510	NA	0.0	0.0	0.0	0.0	0.0	0.0	0	0	2.22E04	0	0	0
4	4.644	458.1510	NA	0.0	0.0	0.1	0.0	0.0	0.0	0	0	5.95E04	0	0	0
5	5.924	458.1510	NA	0.0	0.0	0.0	0.0	0.0	0.0	0	0	2.66E04	0	0	0

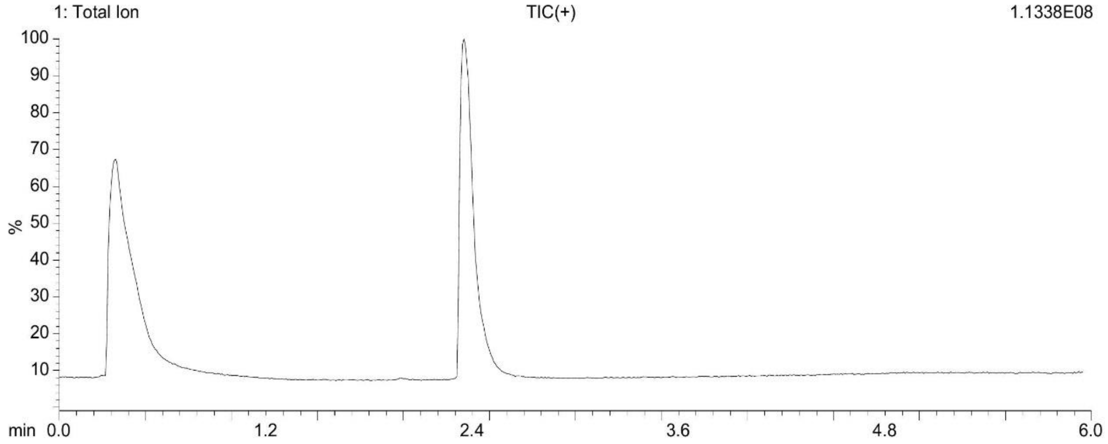

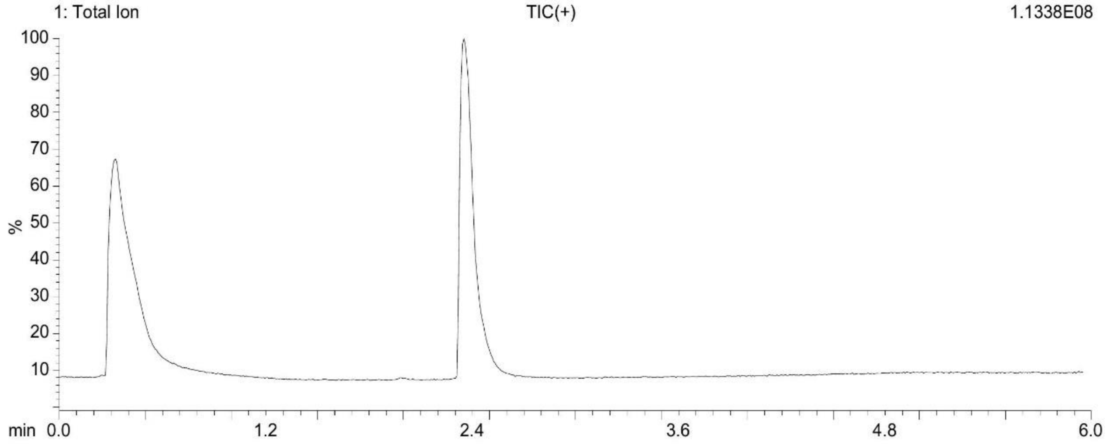

Page 1 of 5 Printed: 1/8/2024 3:18 PM

Analytical Studio Reviewer Report

Sample Name: 415349:01:01 **Acquired:** 5/31/2023 12:58 AM **Instrument:** Agilent TOF **Submitter:**
Location: 1,1:M,1 **Filename:** 1046304843-415349-01-01.d **User:** **Job Code:**

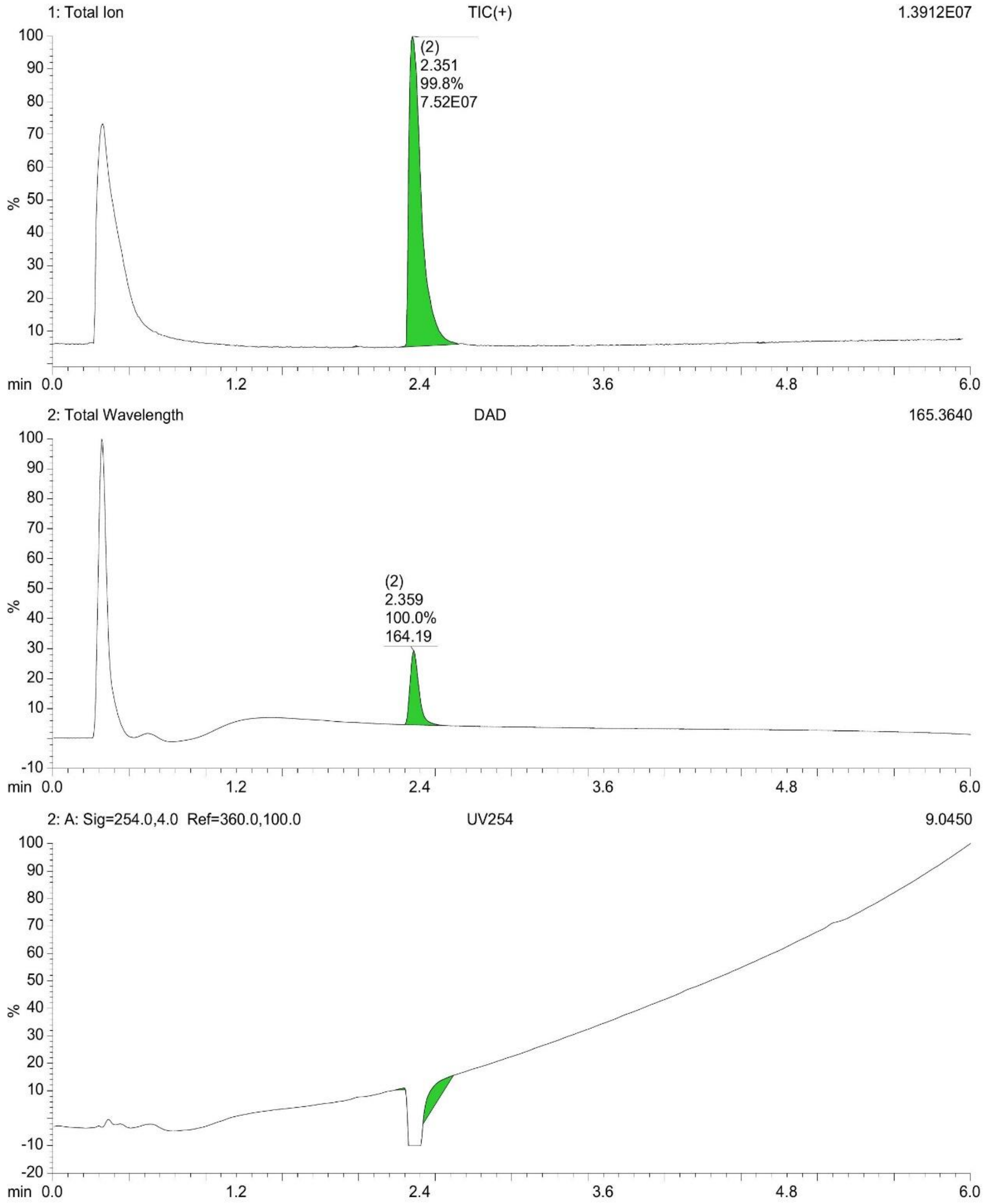

 Printed: 1/8/2024 3:18 PM

Analytical Studio Reviewer Report

Sample Name: 415349:01:01 **Acquired:** 5/31/2023 12:58 AM **Instrument:** Agilent TOF **Submitter:**
Location: 1,1:M,1 **Filename:** 1046304843-415349-01-01.d **User:** **Job Code:**

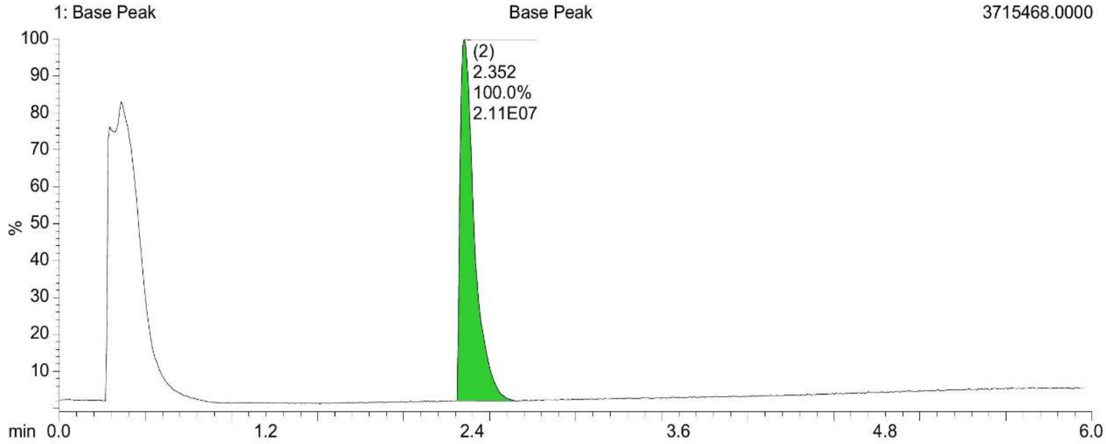

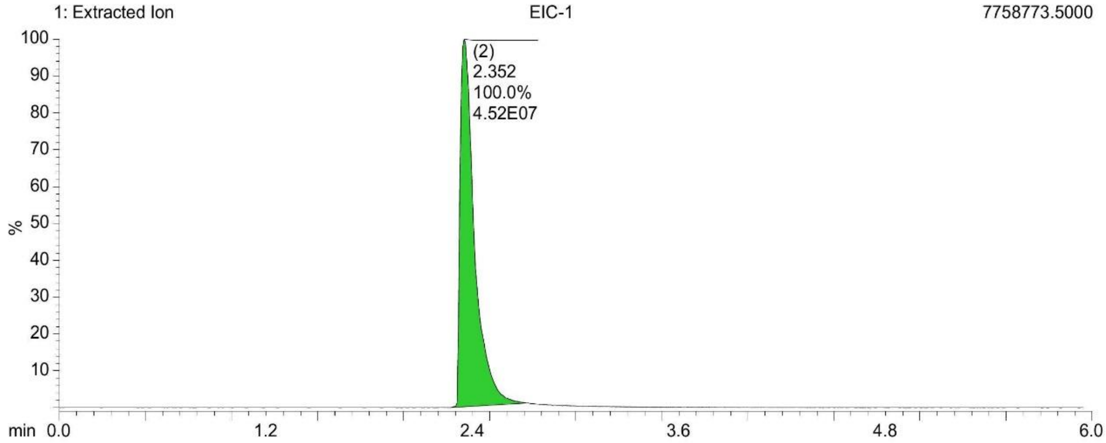

Analytical Studio Reviewer Report

Sample Name: 415349:01:01 **Acquired:** 5/31/2023 12:58 AM **Instrument:** Agilent TOF **Submitter:**
Location: 1,1:M,1 **Filename:** 1046304843-415349-01-01.d **User:** **Job Code:**

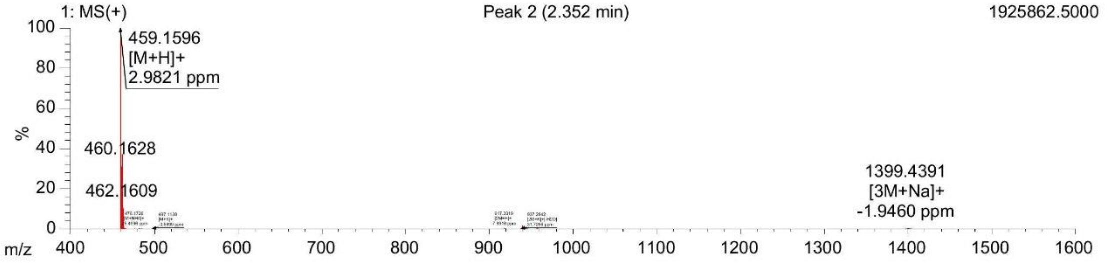

BPM	Error PPM	Error mDa	Target
459.1596	-1.9460	-2.7233	459.1…

HPLC traces (**7**)

Analytical Studio Reviewer Report

Sample Name: 419023:01:03 **Acquired:** 7/21/2022 11:48 PM **Instrument:** Agilent TOF **Submitter:**
Location: 1,10:D,7 **Filename:** 1046306031-419023-01-03.d **User:** **Job Code:**

				Area %						Area Abs					
Peak #	Time	Target ...	Found	TIC(+)	TIC(+)	TIC(+)	UV254	DAD	Base ...	TIC(+)	TIC(+)	TIC(+)	UV254	DAD	Base Peak
1	1.790	411.1253	Yes	0.0	0.0	97.9	100.0	100.0	100.0	0	0	########	16.59	8.23	########
2	3.448	411.1253	NA	0.0	0.0	0.8	0.0	0.0	0.0	0	0	17332.68	0	0	0
3	3.928	411.1253	NA	0.0	0.0	1.2	0.0	0.0	0.0	0	0	25931.80	0	0	0

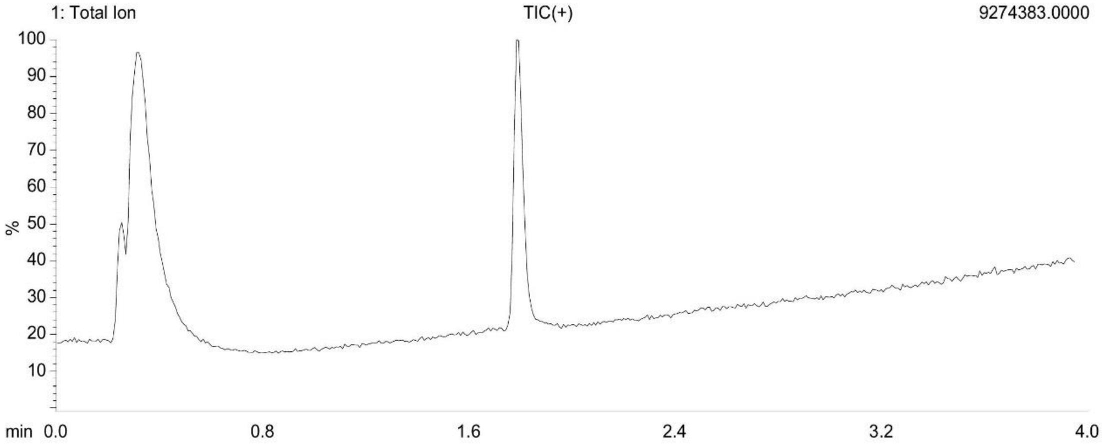

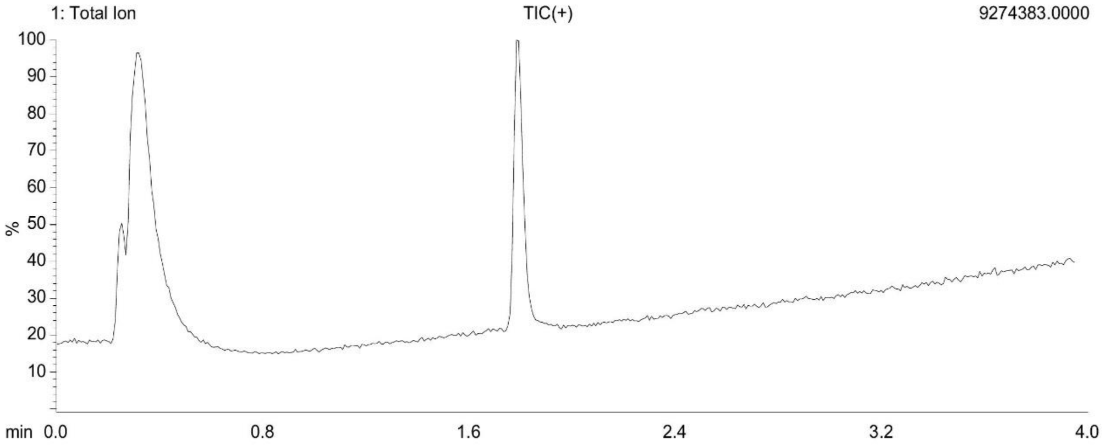

Analytical Studio Reviewer Report

Sample Name: 419023:01:03 **Acquired:** 7/21/2022 11:48 PM **Instrument:** Agilent TOF **Submitter:**
Location: 1,10:D,7 **Filename:** 1046306031-419023-01-03.d **User:** **Job Code:**

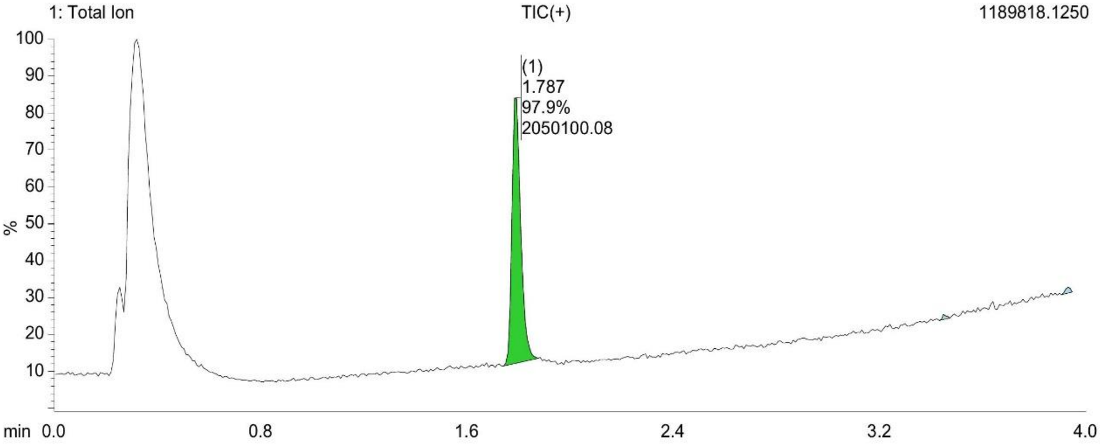

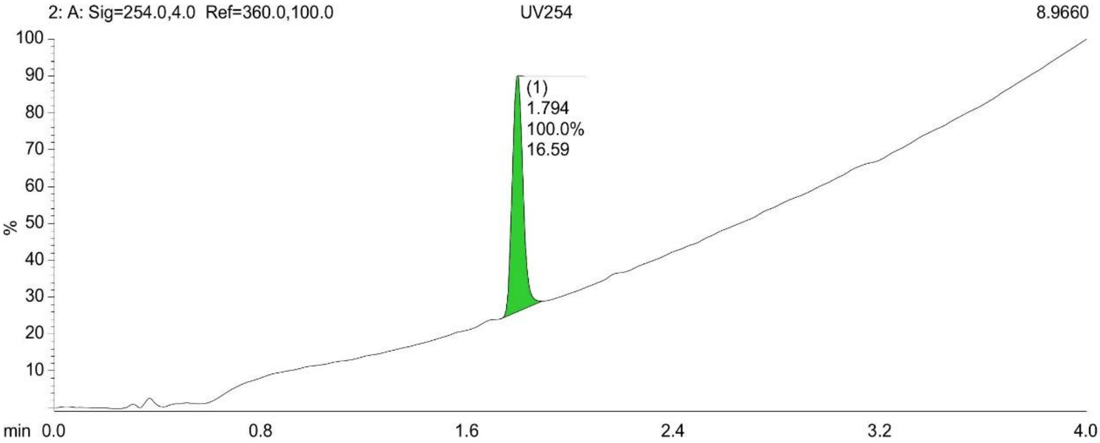

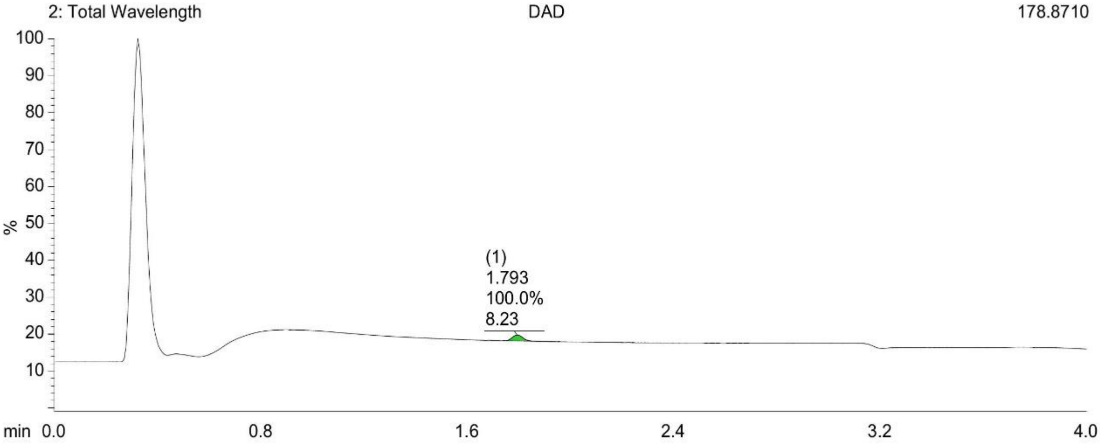

 Printed: 1/9/2024 4:22 PM

Analytical Studio Reviewer Report

Sample Name: 419023:01:03 **Acquired:** 7/21/2022 11:48 PM **Instrument:** Agilent TOF **Submitter:**
Location: 1,10:D,7 **Filename:** 1046306031-419023-01-03.d **User:** **Job Code:**

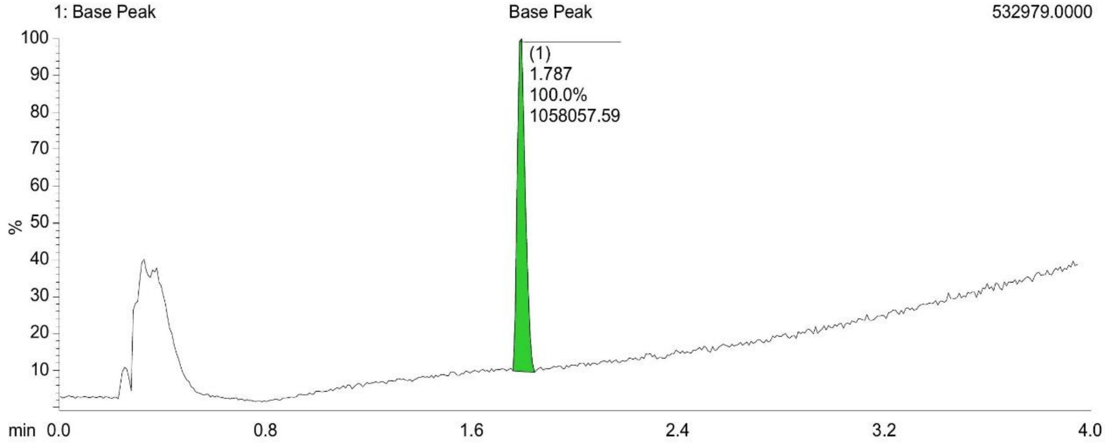

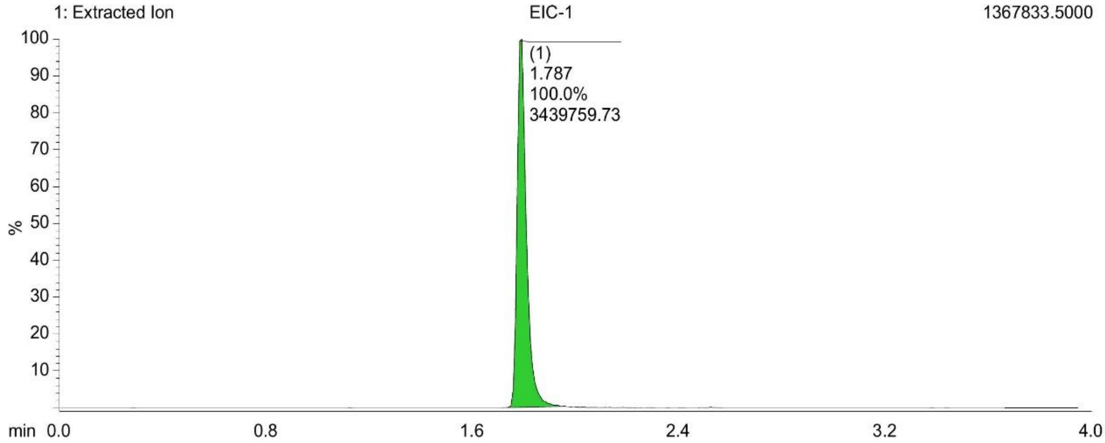

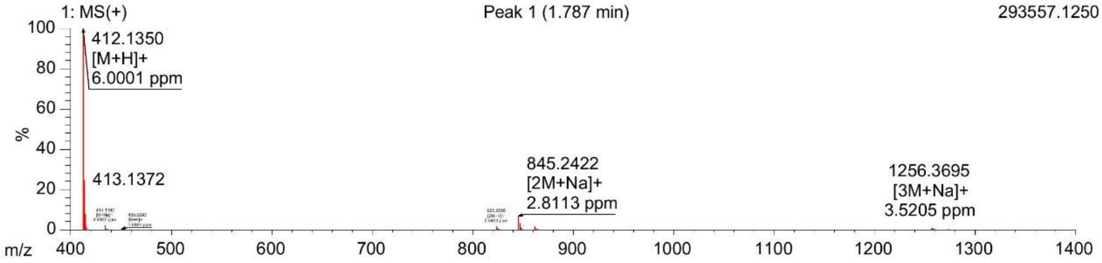

BPM	Error PPM	Error mDa	Target
412.1350	5.2940	6.7358	412.1…

HPLC traces (**8**)

Analytical Studio Reviewer Report

Sample Name: 416113:01:03 **Acquired:** 7/22/2022 6:13 PM **Instrument:** Agilent TOF **Submitter:**
Location: 1,10:K,3 **Filename:** 1046303146-416113-01-03.d **User:** **Job Code:**

				Area %						Area Abs					
Peak #	Time	Target ...	Found	TIC(+)	TIC(+)	TIC(+)	UV254	DAD	Base ...	TIC(+)	TIC(+)	TIC(+)	UV254	DAD	Base Peak
1	1.269	377.2103	NA	0.0	0.0	0.0	0.7	0.0	0.0	0	0	0	0.37	0	0
2	1.353	377.2103	NA	0.0	0.0	3.6	0.0	0.0	3.6	0	0	287647.29	0	0	116046.86
3	1.835	377.2103	NA	0.0	0.0	4.2	3.2	3.8	0.0	0	0	336058.61	1.64	0.88	0
4	2.033	377.2103	NA	0.0	0.0	6.7	2.1	4.3	4.3	0	0	533160.34	1.07	0.98	137602.95
5	2.156	377.2103	NA	0.0	0.0	0.0	0.9	0.0	0.0	0	0	0	0.47	0	0
6	2.365	377.2103	NA	0.0	0.0	4.2	2.8	4.2	0.0	0	0	337636.11	1.45	0.95	0
7	2.475	377.2103	NA	0.0	0.0	1.6	0.0	0.0	0.0	0	0	124631.69	0	0	0
8	2.637	377.2103	NA	0.0	0.0	0.0	0.0	1.1	0.0	0	0	0	0	0.25	0
9	2.914	377.2103	Yes	0.0	0.0	79.8	90.2	86.6	92.1	0	0	########	46.11	19.75	########

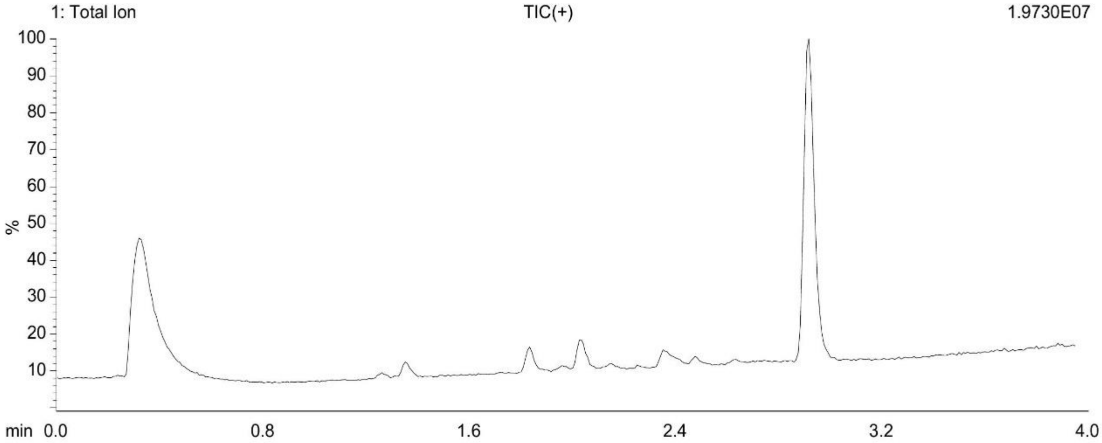

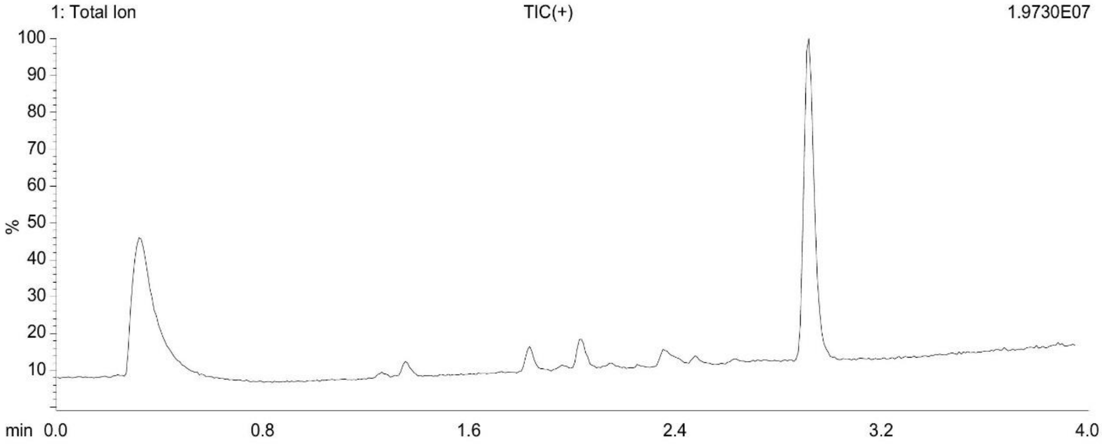

Page 1 of 6 Printed: 1/8/2024 3:19 PM

Analytical Studio Reviewer Report

Sample Name: 416113:01:03 **Acquired:** 7/22/2022 6:13 PM **Instrument:** Agilent TOF **Submitter:**
Location: 1,10:K,3 **Filename:** 1046303146-416113-01-03.d **User:** **Job Code:**

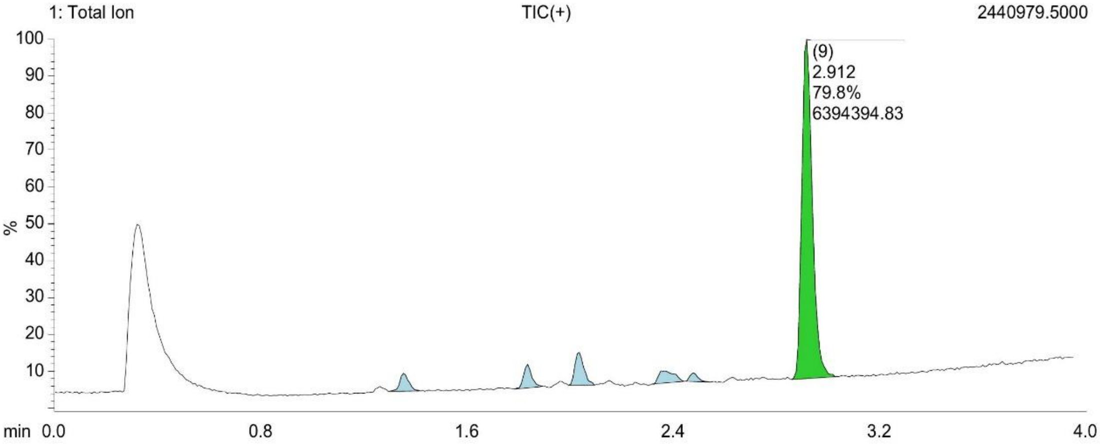

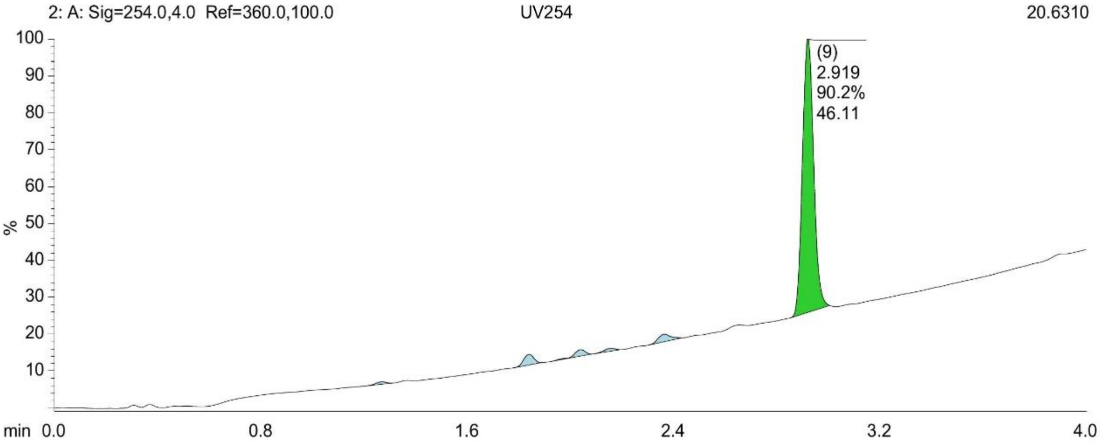

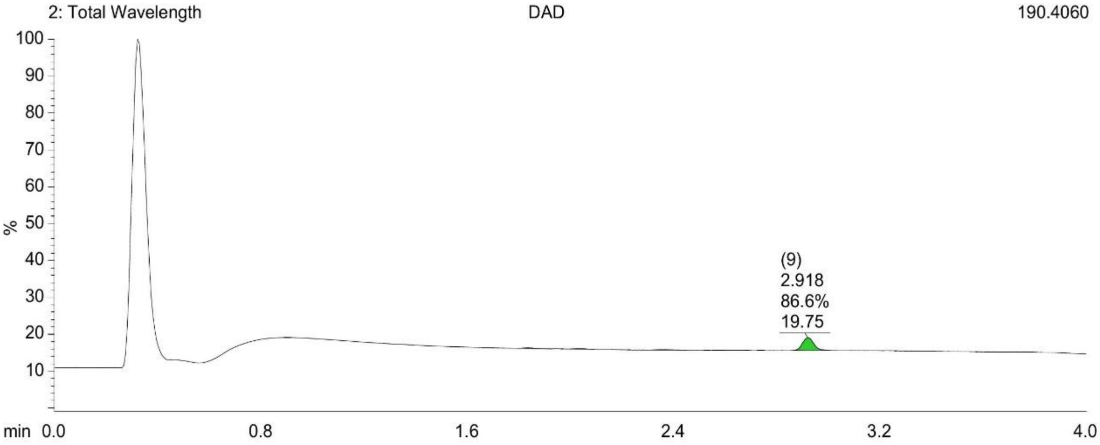

 Printed: 1/8/2024 3:19 PM

Analytical Studio Reviewer Report

Sample Name: 416113:01:03 **Acquired:** 7/22/2022 6:13 PM **Instrument:** Agilent TOF **Submitter:**
Location: 1,10:K,3 **Filename:** 1046303146-416113-01-03.d **User:** **Job Code:**

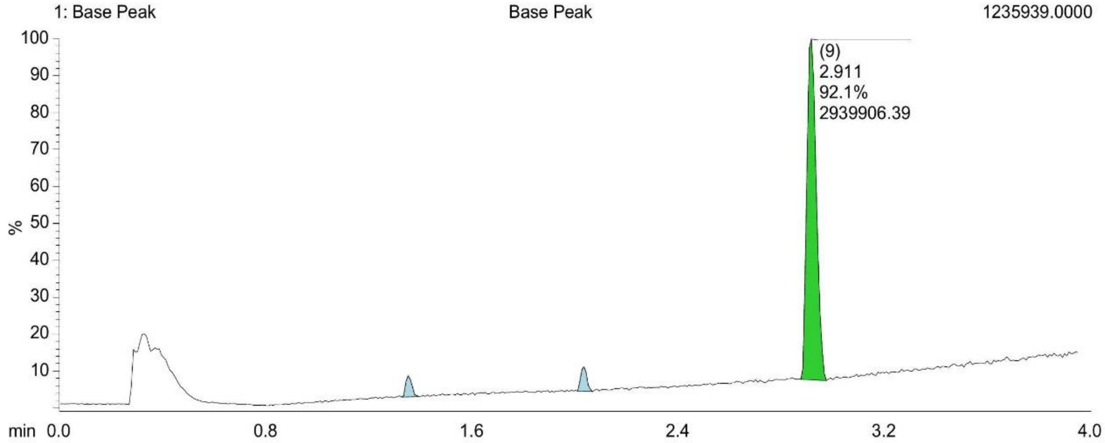

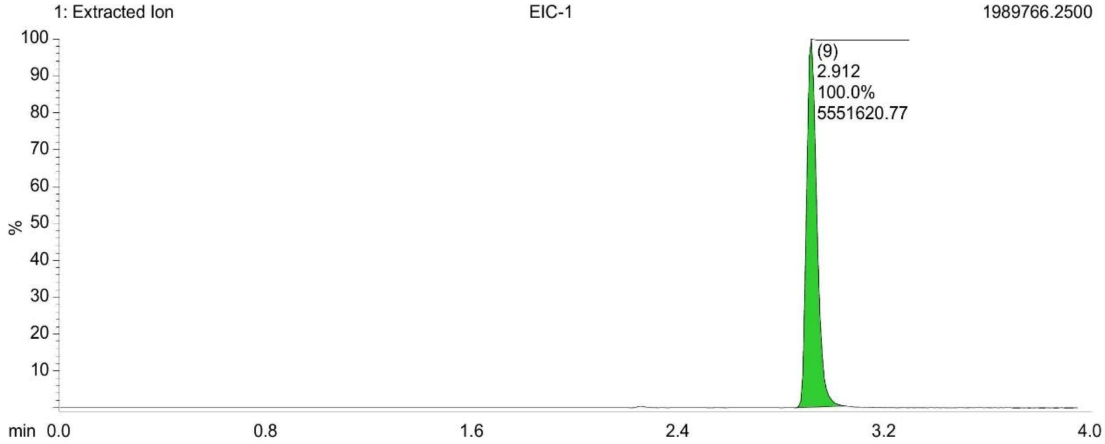

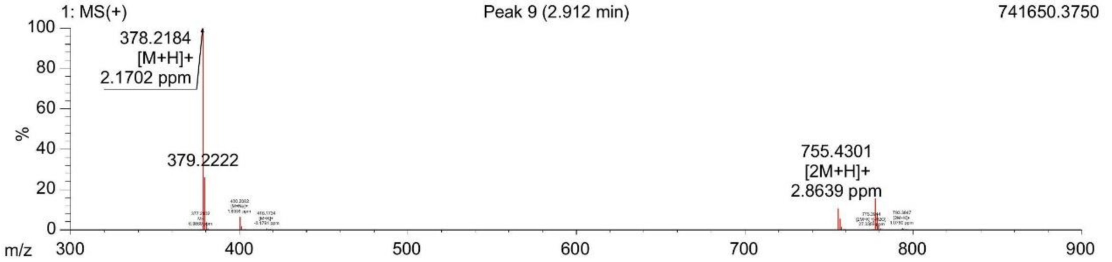

BPM	Error PPM	Error mDa	Target
378.2184	1.0760	0.8537	377.2…

HPLC traces (**9**)

Analytical Studio Reviewer Report

Sample Name: 190521:01:06
Location: 1,3:D,21
Acquired: 7/30/2022 6:52 PM
Filename: 1046266007-190521-01-06.d
Instrument: Agilent TOF
User:
Submitter:
Job Code:

				Area %						Area Abs					
Peak #	Time	Target ...	Found	TIC(+)	TIC(+)	TIC(+)	UV254	DAD	Base ...	TIC(+)	TIC(+)	TIC(+)	UV254	DAD	Base Peak
1	2.191	320.0631	Yes	0.0	0.0	100.0	100.0	100.0	0.0	0	0	11701.25	5.64	4.46	0

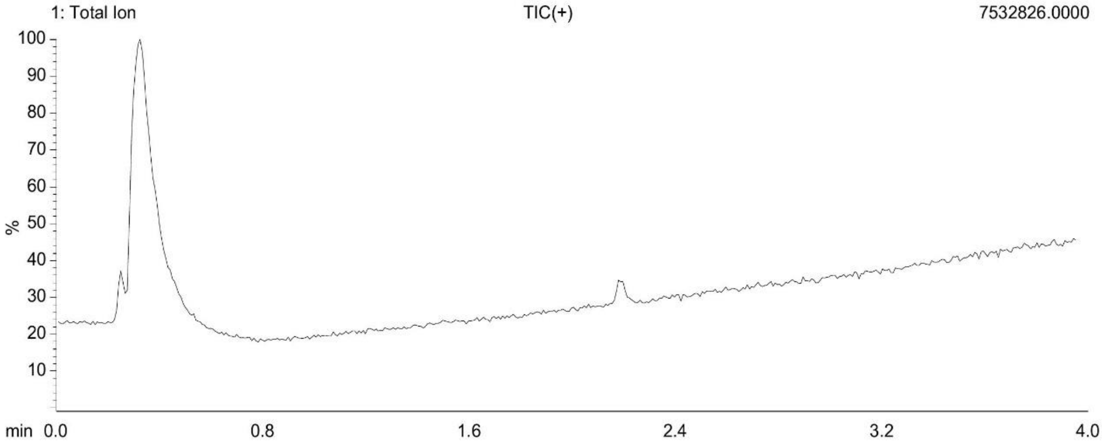

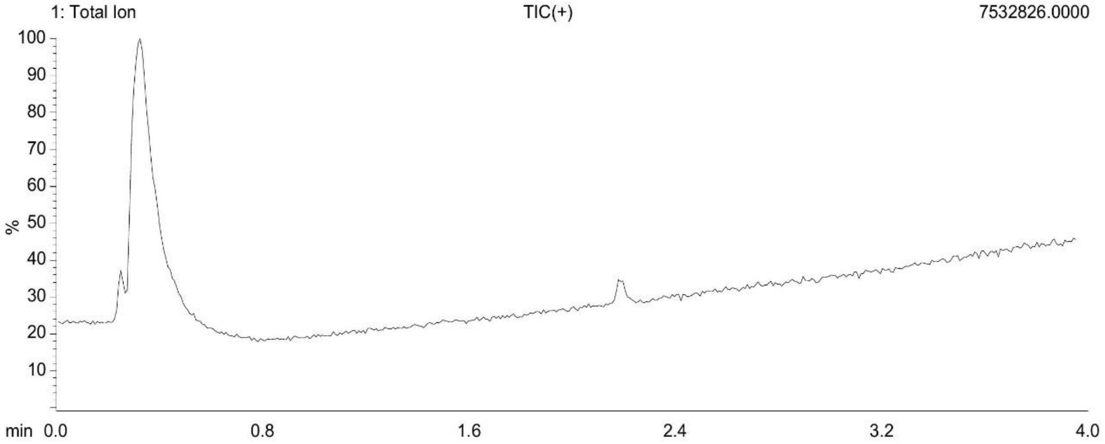

Printed: 1/8/2024 3:19 PM

Analytical Studio Reviewer Report

Sample Name: 190521:01:06 **Acquired:** 7/30/2022 6:52 PM **Instrument:** Agilent TOF **Submitter:**
Location: 1,3:D,21 **Filename:** 1046266007-190521-01-06.d **User:** **Job Code:**

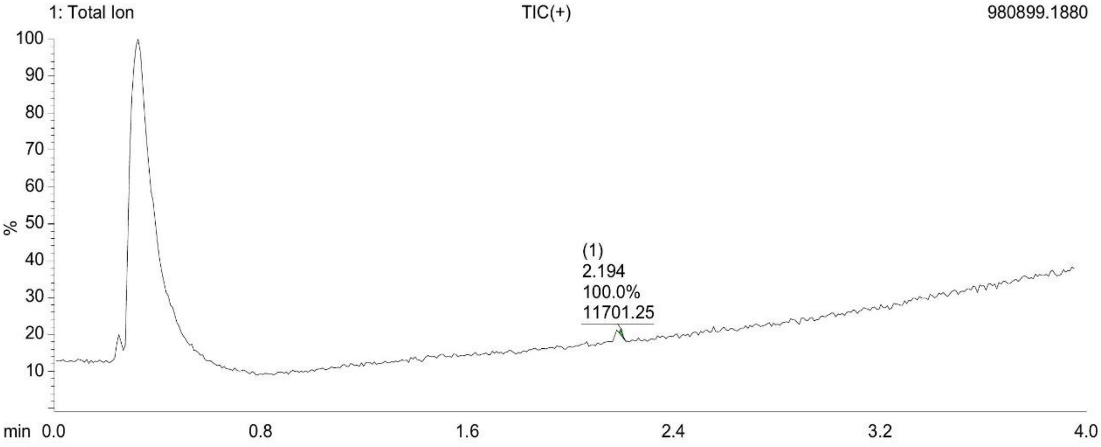

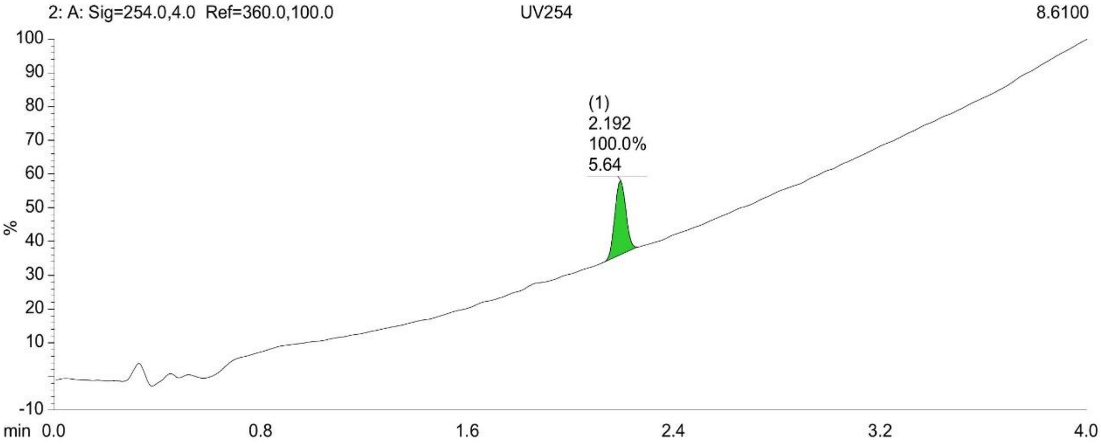

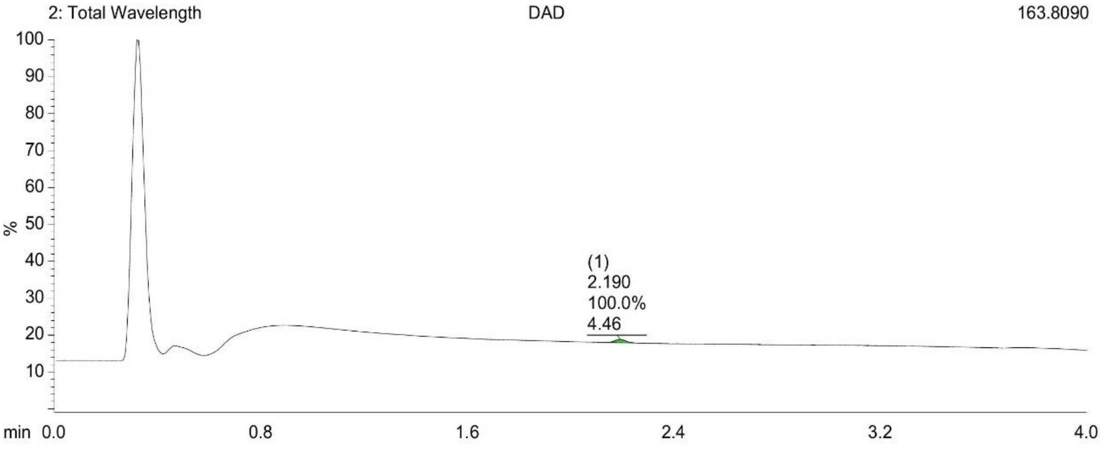

 Printed: 1/8/2024 3:19 PM

Analytical Studio Reviewer Report

Sample Name: 190521:01:06 **Acquired:** 7/30/2022 6:52 PM **Instrument:** Agilent TOF **Submitter:**
Location: 1,3:D,21 **Filename:** 1046266007-190521-01-06.d **User:** **Job Code:**

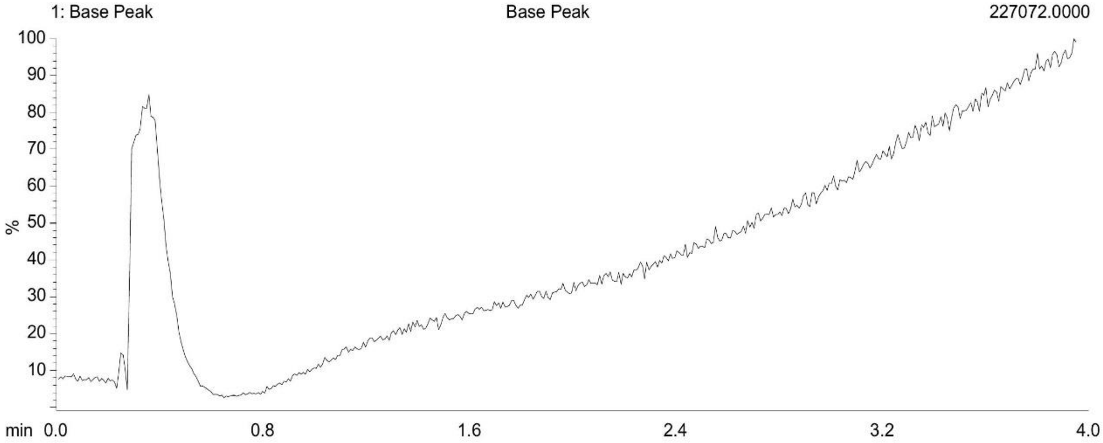

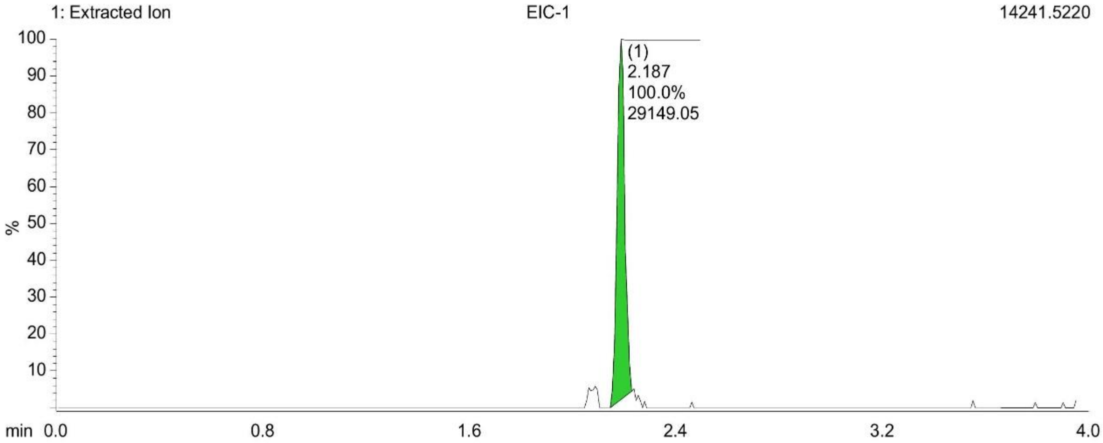

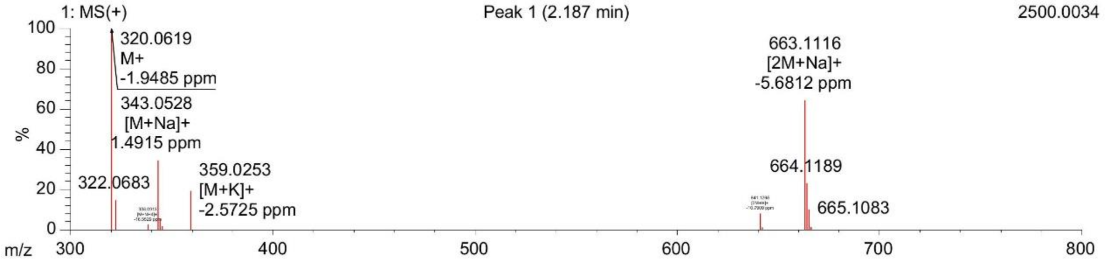

BPM	Error PPM	Error mDa	Target
320.0619	-5.6812	-3.7673	320.0…

Printed: 1/8/2024 3:19 PM

HPLC traces (**10**)

Analytical Studio Reviewer Report

Sample Name: 413857:01:03 **Acquired:** 7/21/2022 7:58 PM **Instrument:** Agilent TOF **Submitter:**
Location: 1,10:B,21 **Filename:** 1046304491-413857-01-03.d **User:** **Job Code:**

				Area %						Area Abs					
Peak #	Time	Target ...	Found	TIC(+)	TIC(+)	TIC(+)	UV254	DAD	Base ...	TIC(+)	TIC(+)	TIC(+)	UV254	DAD	Base Peak
1	1.788	435.0269	NA	0.0	0.0	0.0	9.2	3.2	0.0	0	0	0	1.08	0.55	0
2	2.899	435.0269	NA	0.0	0.0	34.1	0.0	0.0	0.0	0	0	23544.01	0	0	0
3	3.352	435.0269	NA	0.0	0.0	42.0	0.0	0.0	0.0	0	0	29004.28	0	0	0
4	3.416	435.0269	NA	0.0	0.0	23.9	0.0	0.0	0.0	0	0	16498.08	0	0	0
5	3.686	435.0269	Yes	0.0	0.0	0.0	90.8	96.8	0.0	0	0	0	10.72	16.75	0

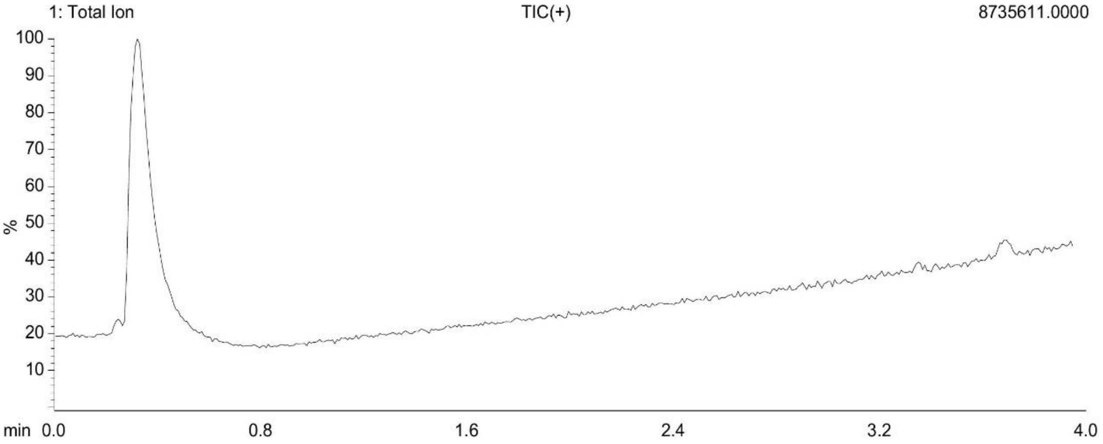

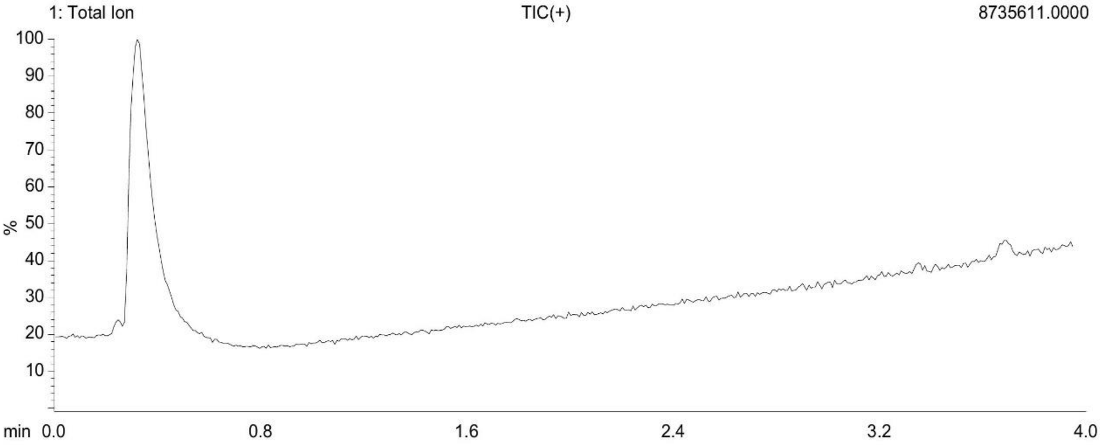

Page 1 of 5 Printed: 1/9/2024 4:22 PM

Analytical Studio Reviewer Report

Sample Name: 413857:01:03 **Acquired:** 7/21/2022 7:58 PM **Instrument:** Agilent TOF **Submitter:**
Location: 1,10:B,21 **Filename:** 1046304491-413857-01-03.d **User:** **Job Code:**

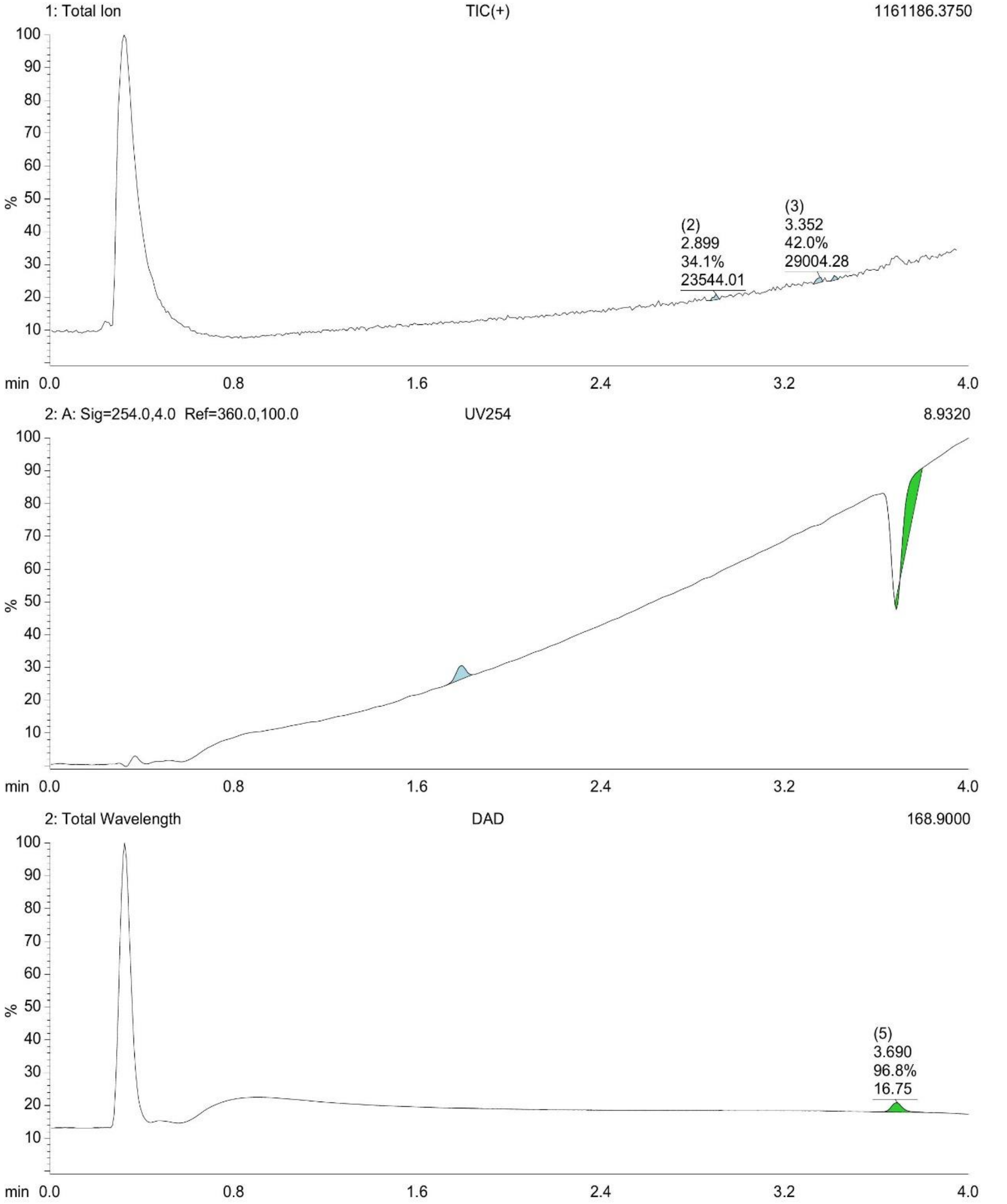

 Printed: 1/9/2024 4:22 PM

Analytical Studio Reviewer Report

Sample Name: 413857:01:03
Location: 1,10:B,21
Acquired: 7/21/2022 7:58 PM
Filename: 1046304491-413857-01-03.d
Instrument: Agilent TOF
User:
Submitter:
Job Code:

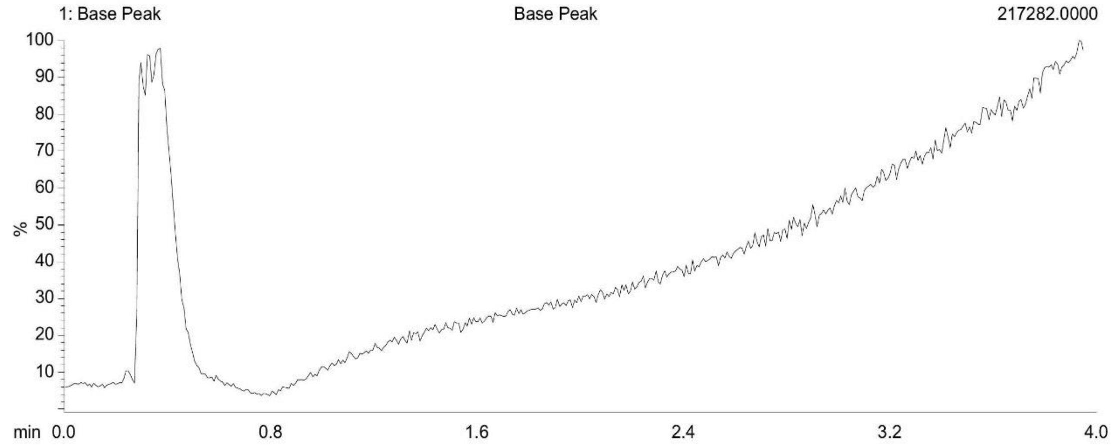

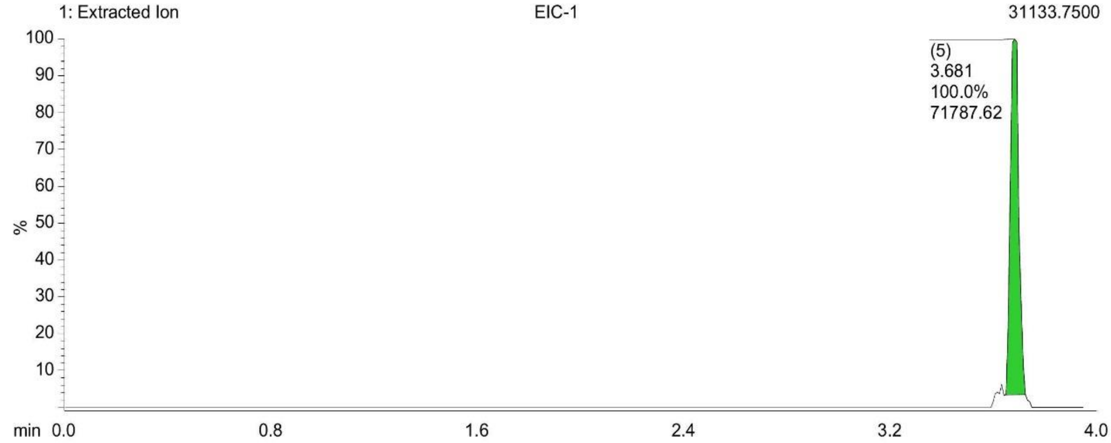

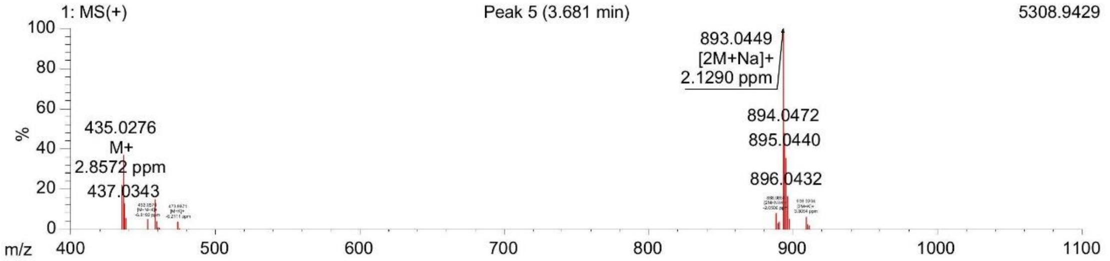

BPM	Error PPM	Error mDa	Target
893.0449	3.8054	3.4592	435.0…

HPLC traces (**11**)

Analytical Studio Reviewer Report

Sample Name: 247284:01:12 **Acquired:** 11/21/2022 12:06 PM **Instrument:** Agilent TOF **Submitter:**
Location: 1,7:H,2 **Filename:** 1046240696-247284-01-12.d **User:** **Job Code:**

				Area %						Area Abs					
Peak #	Time	Target ...	Found	TIC(+)	TIC(+)	TIC(+)	UV254	DAD	Base ...	TIC(+)	TIC(+)	TIC(+)	UV254	DAD	Base Peak
1	0.308	267.0968	Yes	0.0	0.0	0.0	100.0	0.0	0.0	0	0	0	73.26	0	0

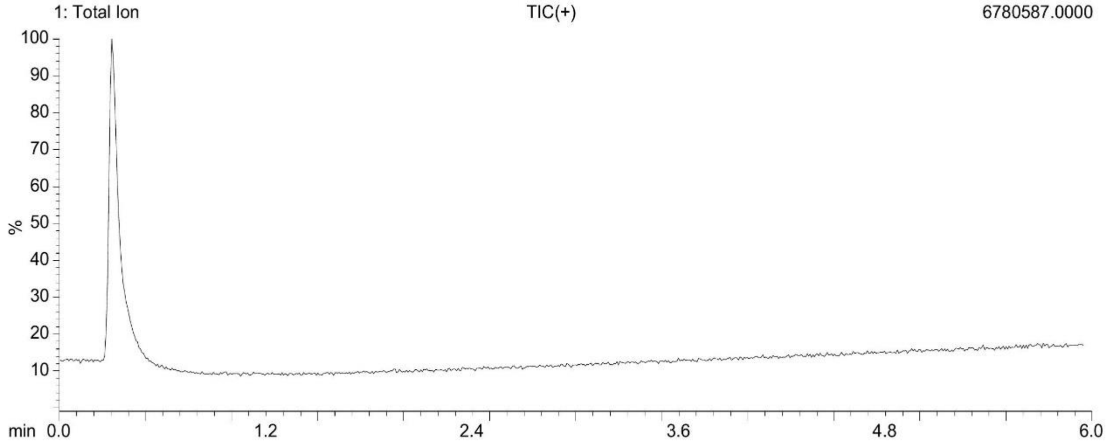

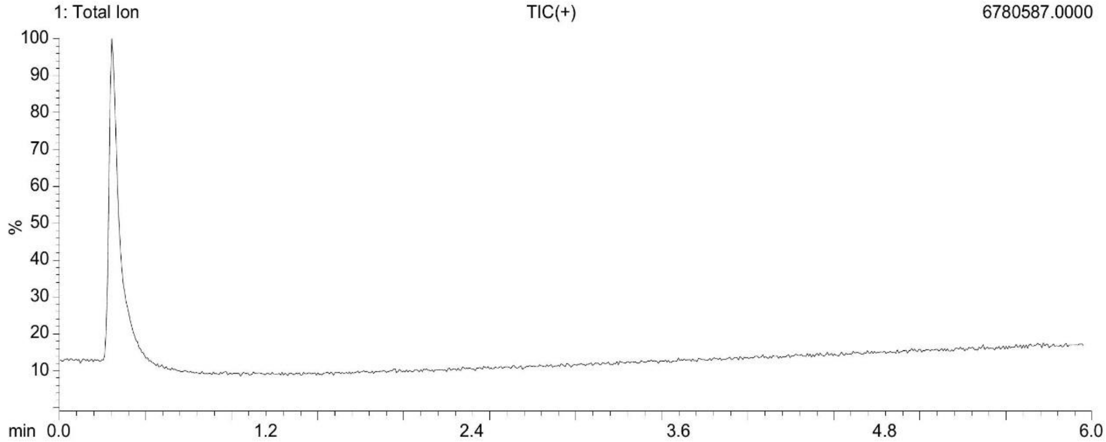

Page 1 of 3 Printed: 1/8/2024 3:18 PM

Analytical Studio Reviewer Report

Sample Name: 247284:01:12 **Acquired:** 11/21/2022 12:06 PM **Instrument:** Agilent TOF **Submitter:**
Location: 1,7:H,2 **Filename:** 1046240696-247284-01-12.d **User:** **Job Code:**

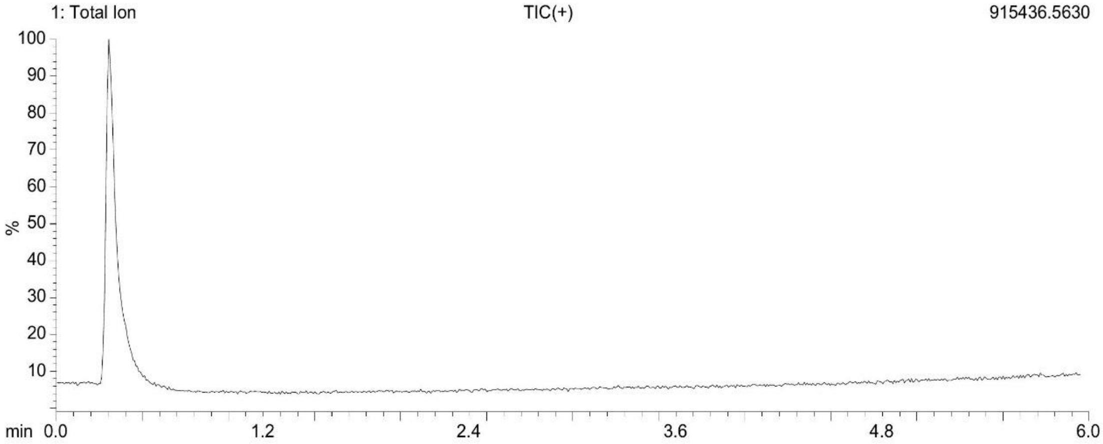

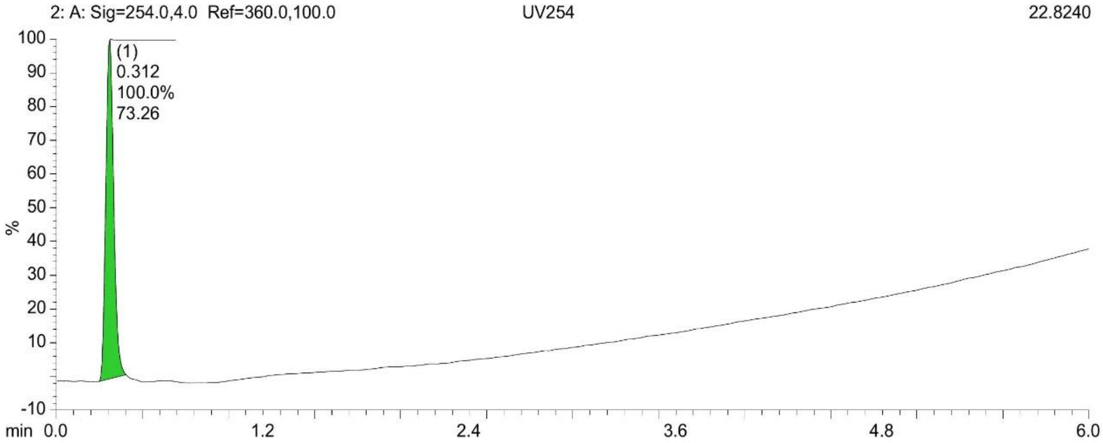

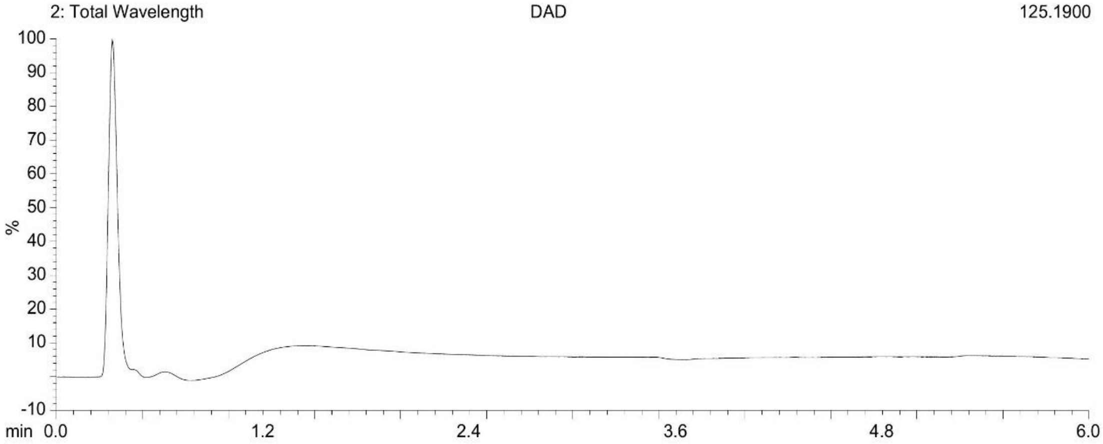

 Printed: 1/8/2024 3:18 PM

Analytical Studio Reviewer Report

Sample Name: 247284:01:12 **Acquired:** 11/21/2022 12:06 PM **Instrument:** Agilent TOF **Submitter:**
Location: 1,7:H,2 **Filename:** 1046240696-247284-01-12.d **User:** **Job Code:**

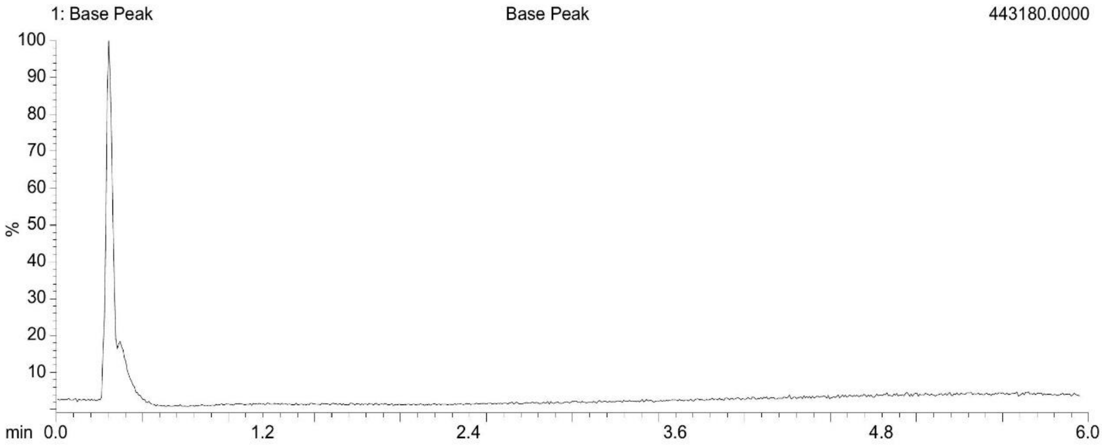

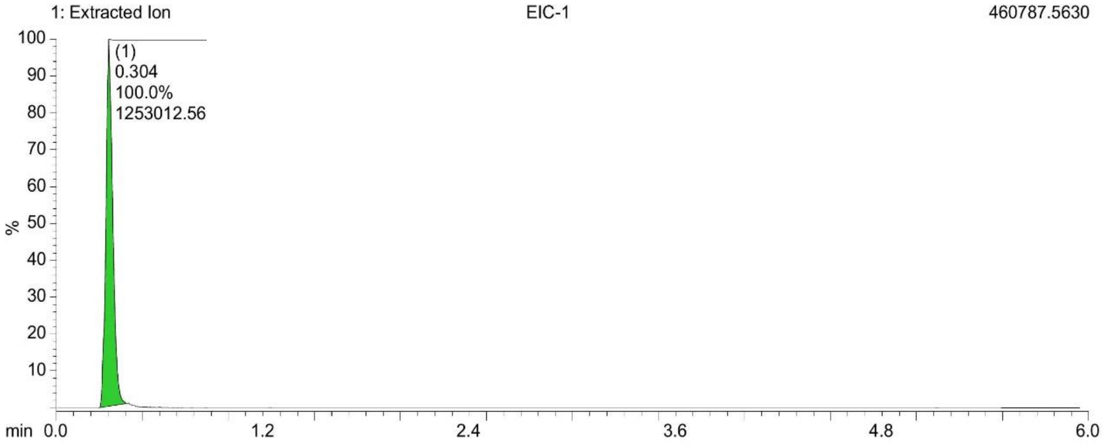

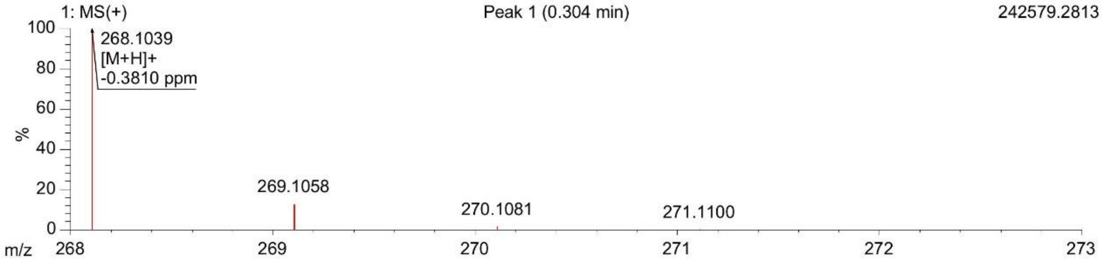

BPM	Error PPM	Error mDa	Target
268.1039	-0.3810	-0.1021	########

 Printed: 1/8/2024 3:18 PM

HPLC traces (**12**)

Analytical Studio Reviewer Report

Sample Name: 413687:01:03 **Acquired:** 7/21/2022 10:02 AM **Instrument:** Agilent TOF **Submitter:**
Location: 1,9:M,7 **Filename:** 1046303497-413687-01-03.d **User:** **Job Code:**

				Area %						Area Abs					
Peak #	Time	Target ...	Found	TIC(+)	TIC(+)	TIC(+)	DAD	UV254	Base ...	TIC(+)	TIC(+)	TIC(+)	DAD	UV254	Base Peak
1	0.819	355.1532	NA	0.0	0.0	1.2	0.0	0.0	0.0	0	0	12167.93	0	0	0
2	1.816	355.1532	NA	0.0	0.0	0.0	4.0	3.2	0.0	0	0	0	0.52	0.64	0
3	2.068	355.1532	NA	0.0	0.0	0.0	1.4	3.3	0.0	0	0	0	0.19	0.65	0
4	2.344	355.1532	Yes	0.0	0.0	89.3	85.0	90.6	100.0	0	0	869940.74	11.04	17.90	59118.25
5	2.553	355.1532	NA	0.0	0.0	0.0	4.3	1.5	0.0	0	0	0	0.55	0.29	0
6	2.662	355.1532	NA	0.0	0.0	5.3	0.0	0.0	0.0	0	0	51474.12	0	0	0
7	2.864	355.1532	NA	0.0	0.0	0.0	5.3	1.4	0.0	0	0	0	0.69	0.27	0
8	3.638	355.1532	NA	0.0	0.0	2.1	0.0	0.0	0.0	0	0	20863.62	0	0	0
9	3.687	355.1532	NA	0.0	0.0	2.1	0.0	0.0	0.0	0	0	20267.63	0	0	0

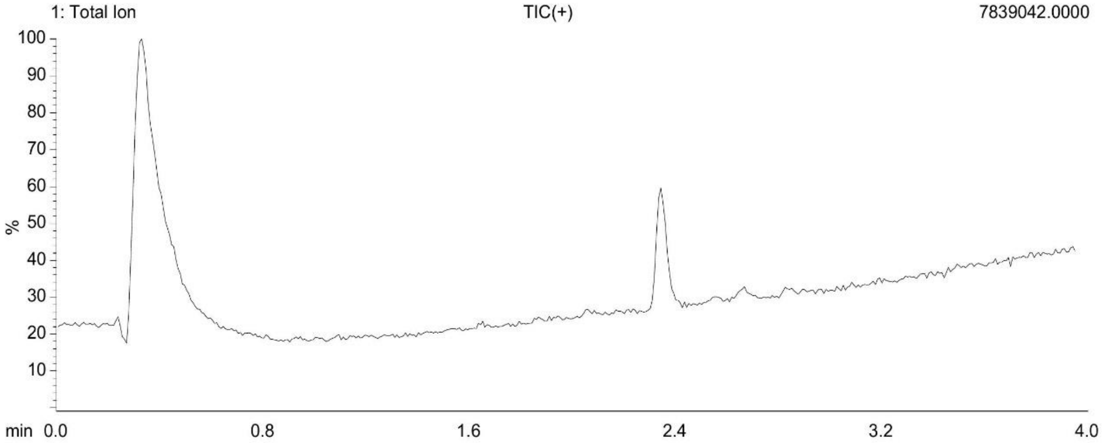

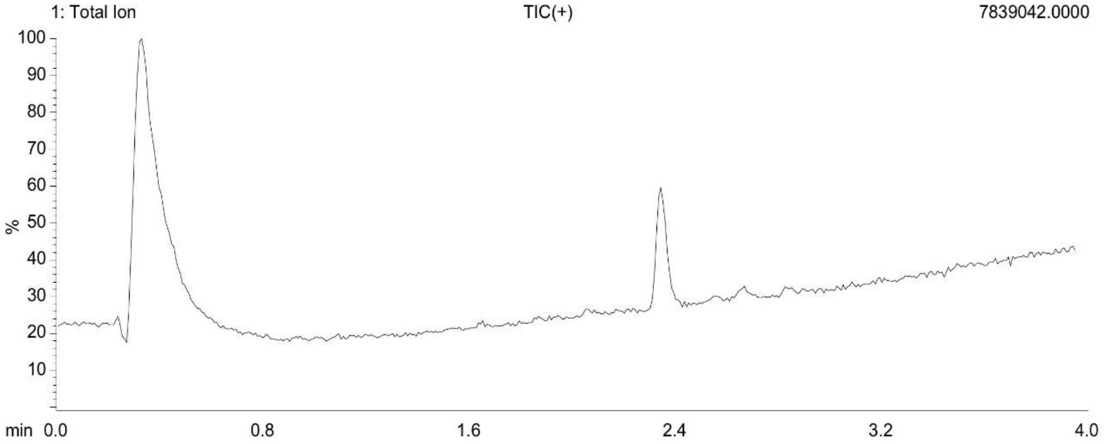

Page 1 of 6 Printed: 1/8/2024 3:19 PM

Analytical Studio Reviewer Report

Sample Name: 413687:01:03 **Acquired:** 7/21/2022 10:02 AM **Instrument:** Agilent TOF **Submitter:**
Location: 1,9:M,7 **Filename:** 1046303497-413687-01-03.d **User:** **Job Code:**

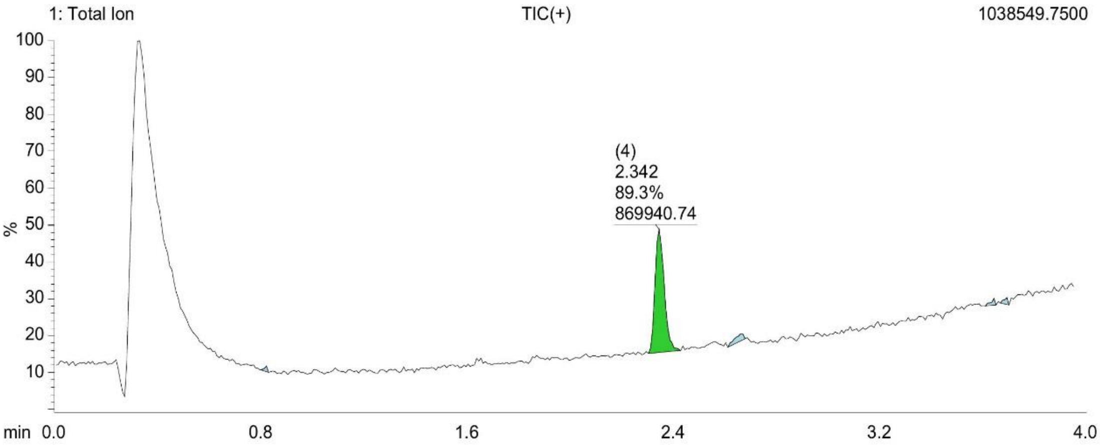

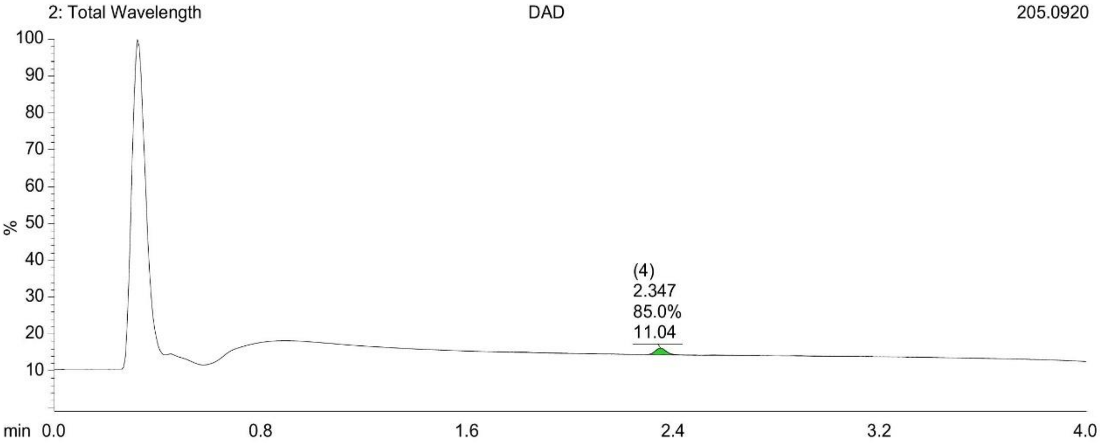

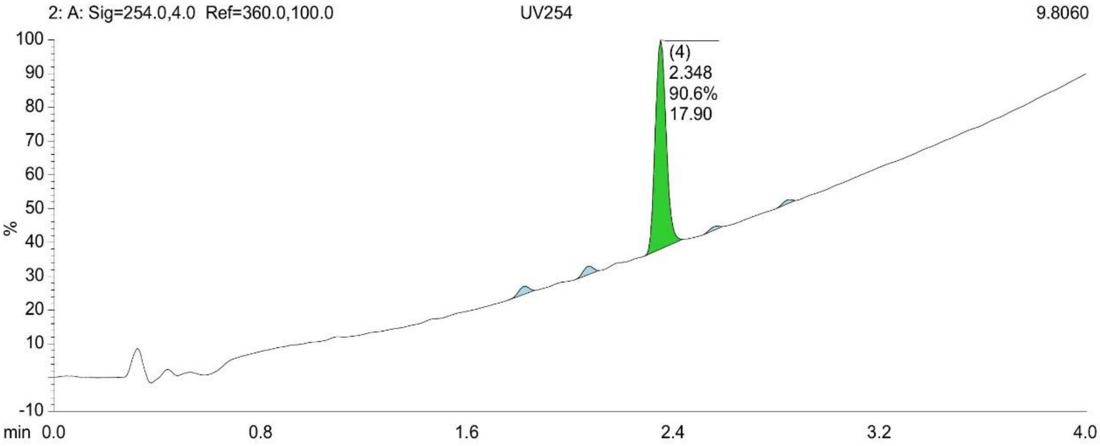

 Printed: 1/8/2024 3:19 PM

Analytical Studio Reviewer Report

Sample Name: 413687:01:03 **Acquired:** 7/21/2022 10:02 AM **Instrument:** Agilent TOF **Submitter:**
Location: 1,9:M,7 **Filename:** 1046303497-413687-01-03.d **User:** **Job Code:**

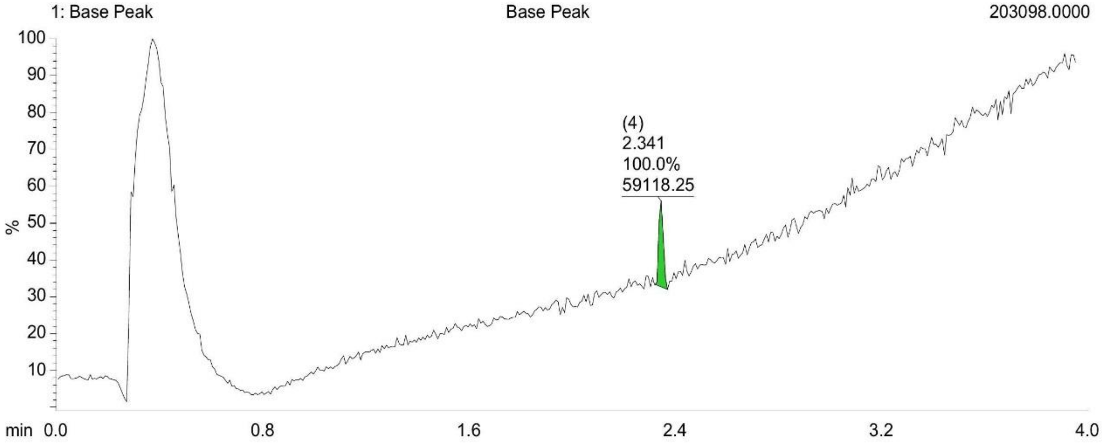

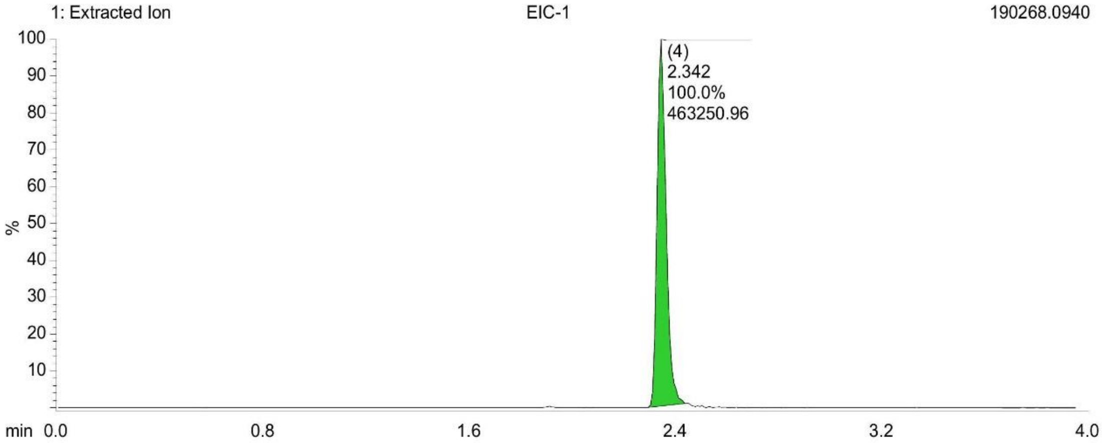

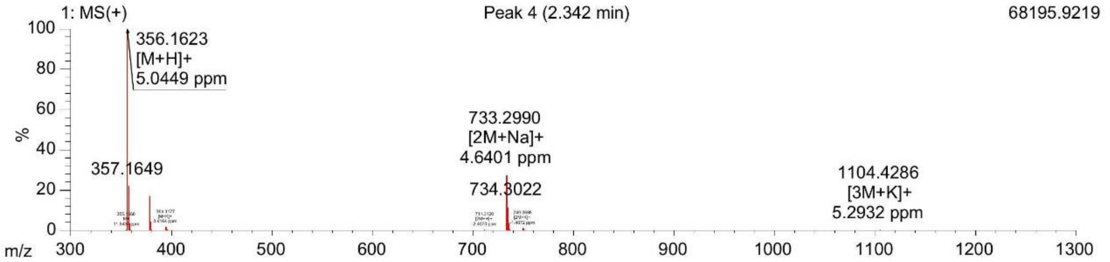

BPM	Error PPM	Error mDa	Target
356.1623	5.2932	5.8459	355.1…

HPLC traces (**13**)

Analytical Studio Reviewer Report

Sample Name: 416564:01:03 **Acquired:** 7/22/2022 5:21 PM **Instrument:** Agilent TOF **Submitter:**
Location: 1,10:J,20 **Filename:** 1046305777-416564-01-03.d **User:** **Job Code:**

				Area %						Area Abs					
Peak #	Time	Target ...	Found	TIC(+)	TIC(+)	TIC(+)	UV254	DAD	Base ...	TIC(+)	TIC(+)	TIC(+)	UV254	DAD	Base Peak
1	1.999	350.1267	NA	0.0	0.0	0.3	0.0	0.0	0.0	0	0	7837.08	0	0	0
2	3.013	350.1267	NA	0.0	0.0	0.9	5.9	0.2	0.0	0	0	22870.50	0.42	0.15	0
3	3.194	350.1267	Yes	0.0	0.0	98.5	94.1	99.8	100.0	0	0	########	6.61	62.99	291232.20
4	3.778	350.1267	NA	0.0	0.0	0.4	0.0	0.0	0.0	0	0	9906.47	0	0	0

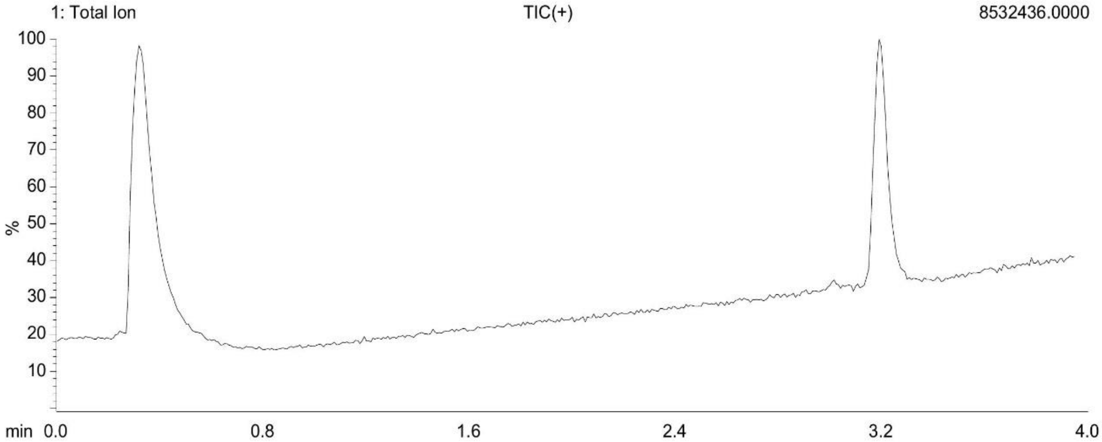

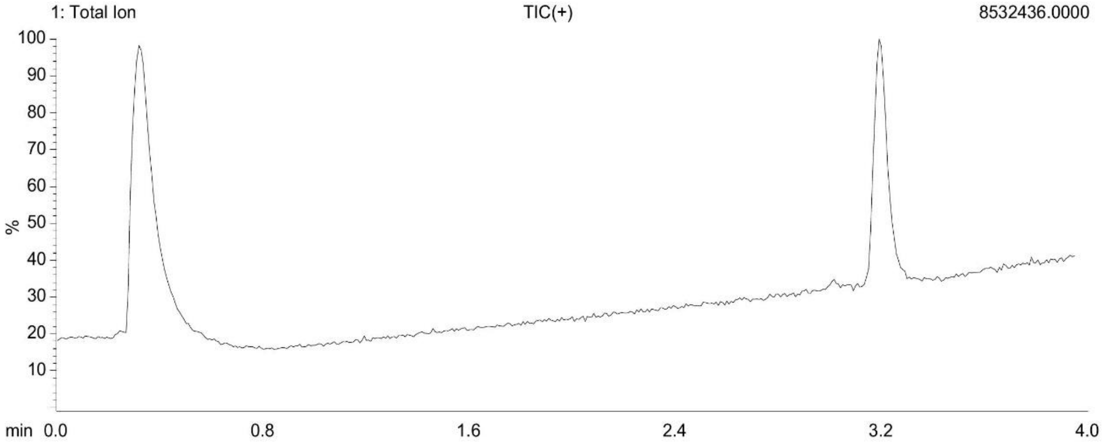

Page 1 of 4 Printed: 1/8/2024 3:19 PM

Analytical Studio Reviewer Report

Sample Name: 416564:01:03 **Acquired:** 7/22/2022 5:21 PM **Instrument:** Agilent TOF **Submitter:**
Location: 1,10:J,20 **Filename:** 1046305777-416564-01-03.d **User:** **Job Code:**

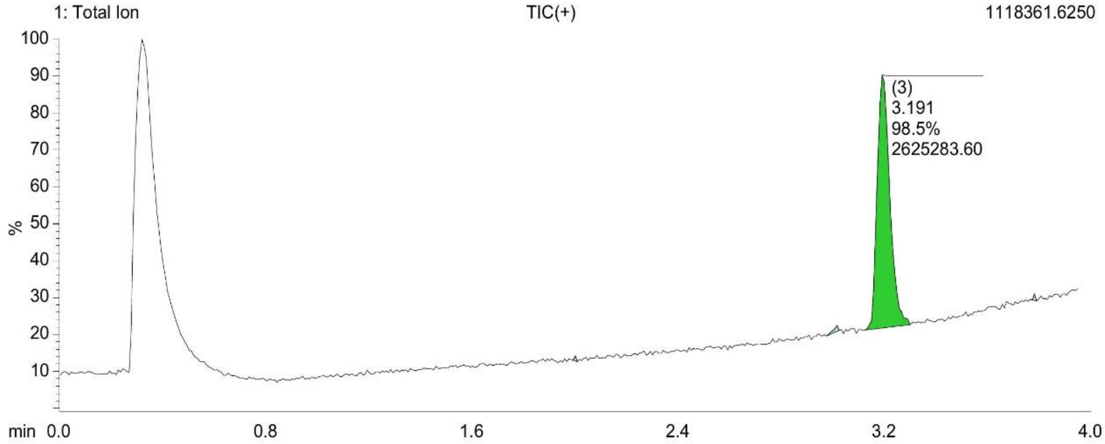

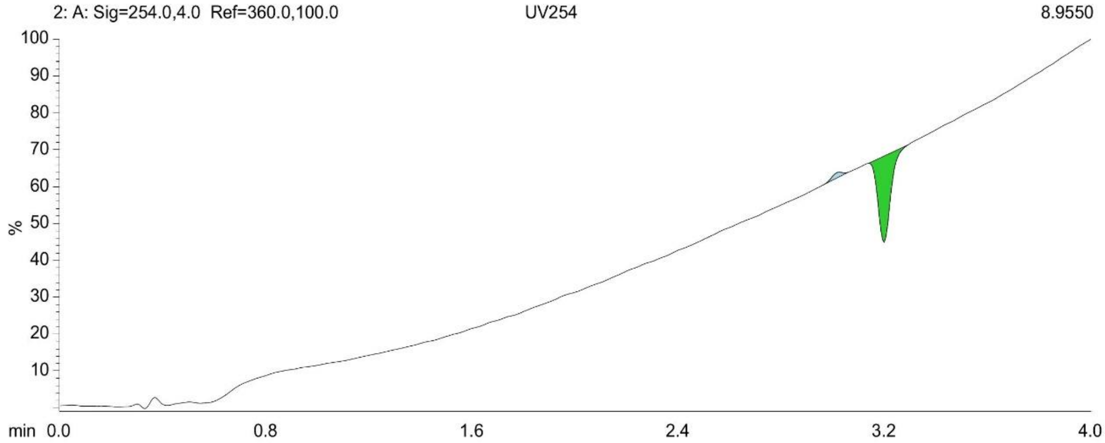

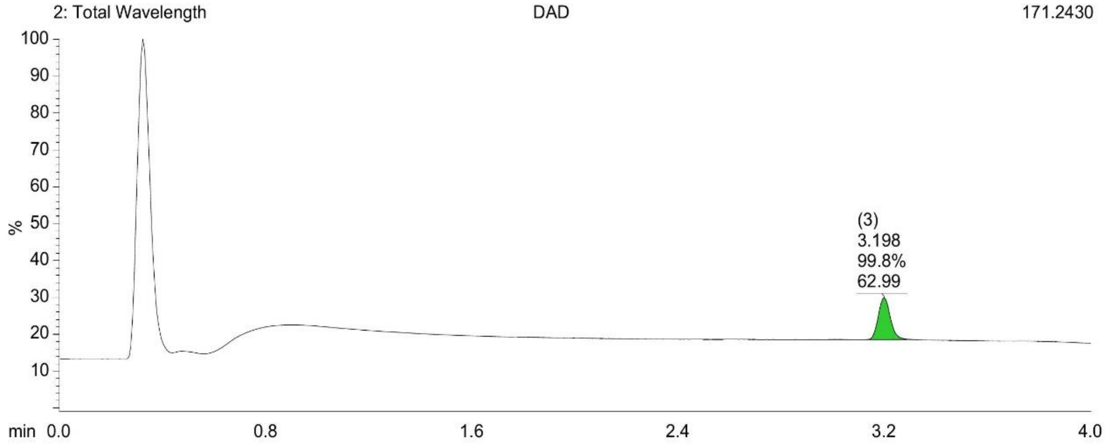

 Printed: 1/8/2024 3:19 PM

Analytical Studio Reviewer Report

Sample Name: 416564:01:03 **Acquired:** 7/22/2022 5:21 PM **Instrument:** Agilent TOF **Submitter:**
Location: 1,10:J,20 **Filename:** 1046305777-416564-01-03.d **User:** **Job Code:**

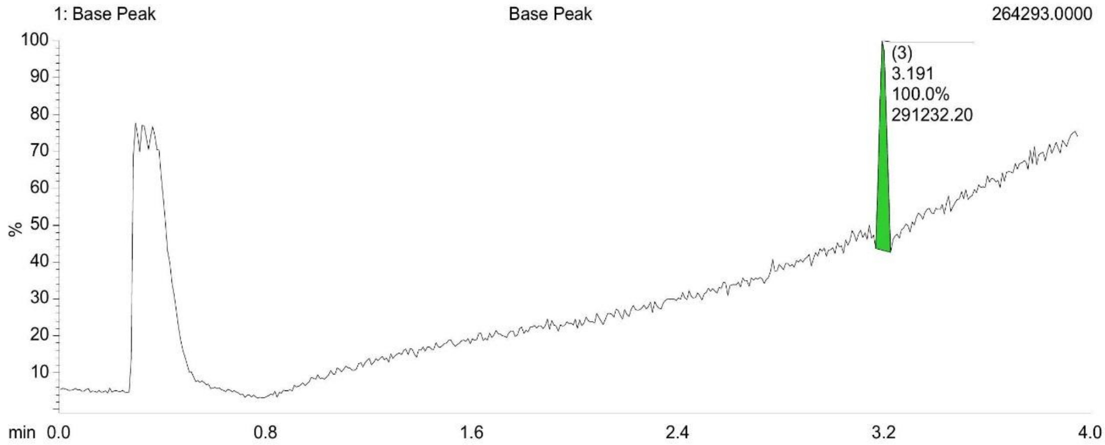

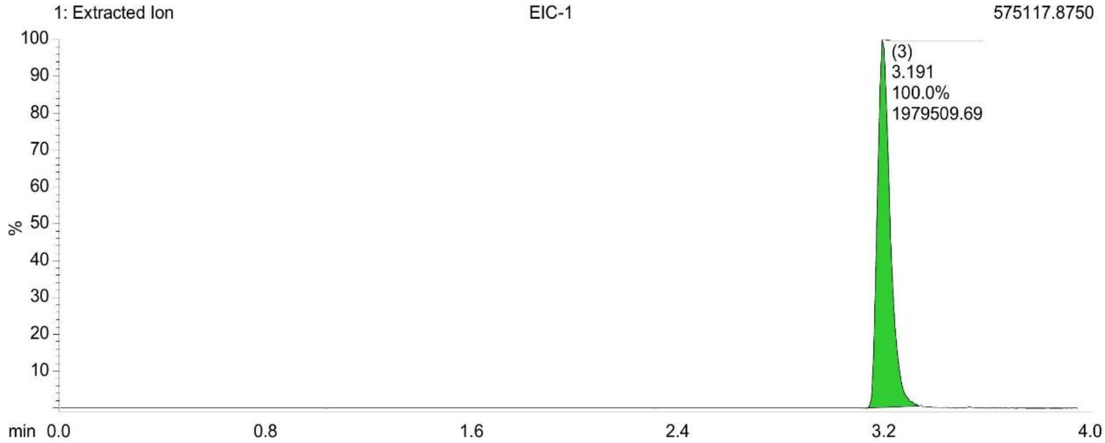

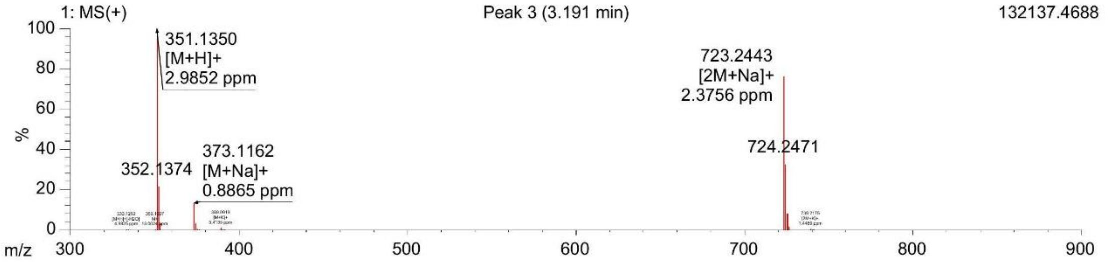

BPM	Error PPM	Error mDa	Target
351.1350	1.4486	1.0709	333.1…

HPLC traces (**14**)

Analytical Studio Reviewer Report

Sample Name: 416682:01:03 **Acquired:** 7/22/2022 11:46 PM **Instrument:** Agilent TOF **Submitter:**
Location: 1,10:M,4 **Filename:** 1046305694-416682-01-03.d **User:** **Job Code:**

				Area %						Area Abs					
Peak #	Time	Target ...	Found	TIC(+)	TIC(+)	TIC(+)	UV254	DAD	Base ...	TIC(+)	TIC(+)	TIC(+)	UV254	DAD	Base Peak
1	1.723	443.1679	NA	0.0	0.0	6.0	0.7	0.2	0.0	0	0	184226.29	0.19	0.05	0
2	2.452	443.1679	Yes	0.0	0.0	94.0	99.3	99.8	100.0	0	0	########	27.78	23.40	448123.50

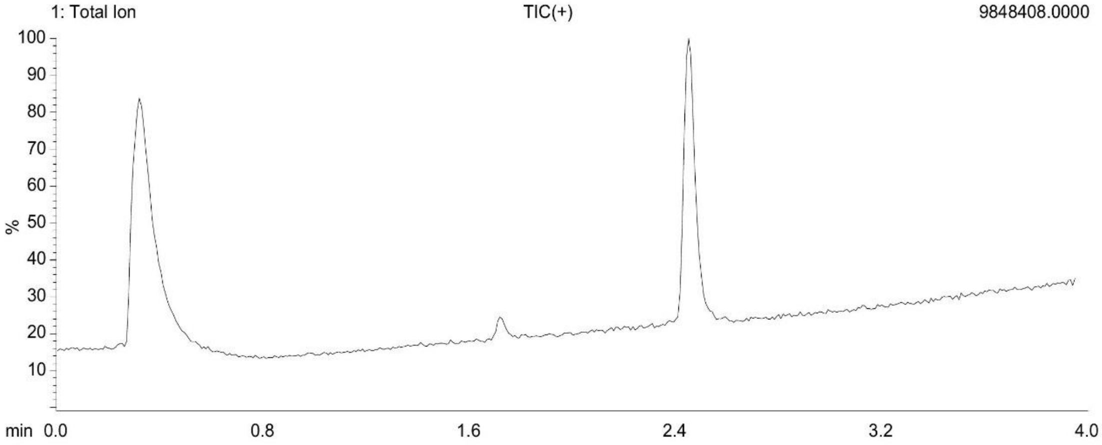

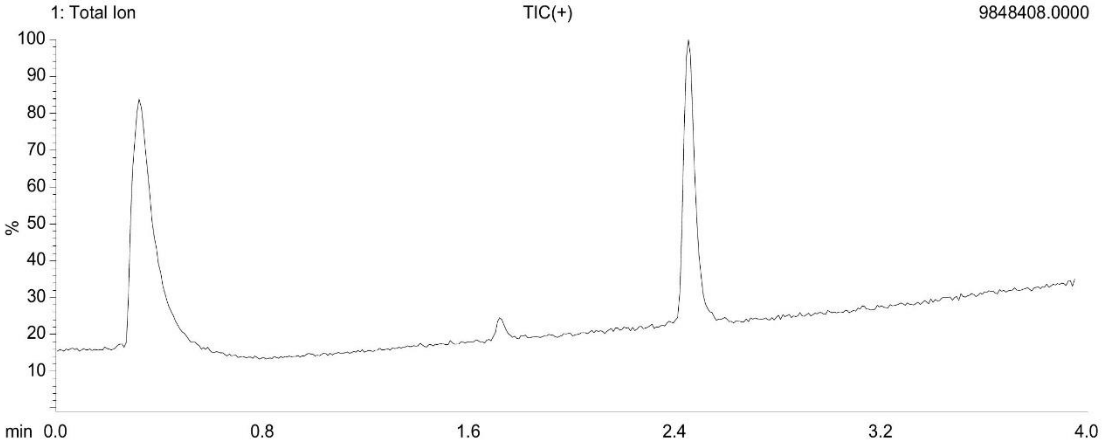

Page 1 of 4 Printed: 1/8/2024 3:19 PM

Analytical Studio Reviewer Report

Sample Name: 416682:01:03 **Acquired:** 7/22/2022 11:46 PM **Instrument:** Agilent TOF **Submitter:**
Location: 1,10:M,4 **Filename:** 1046305694-416682-01-03.d **User:** **Job Code:**

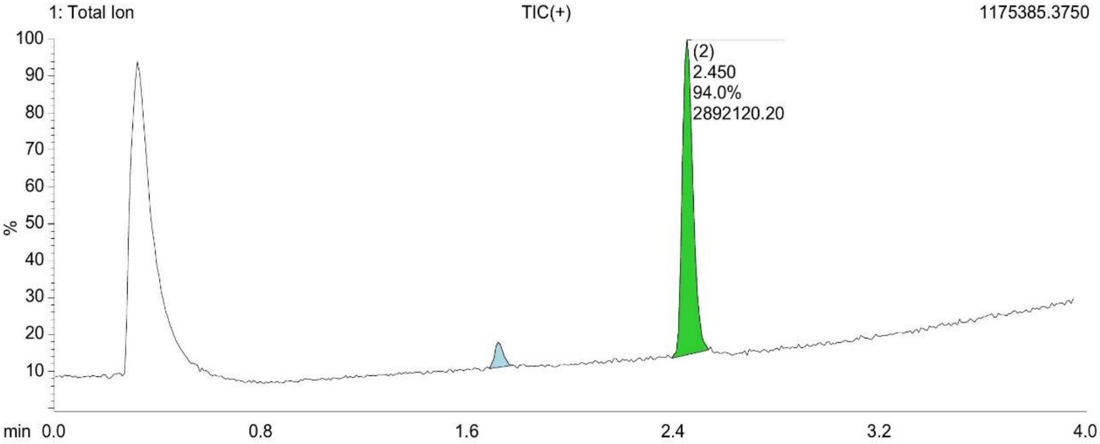

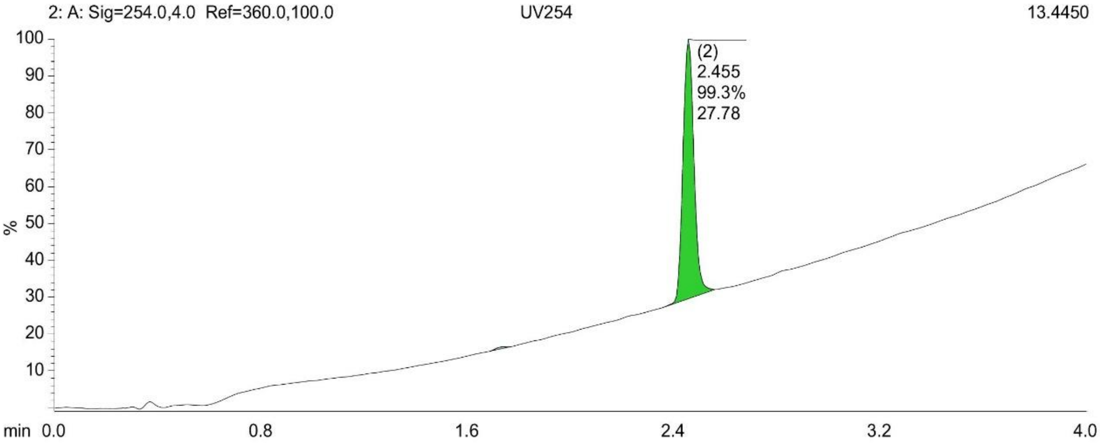

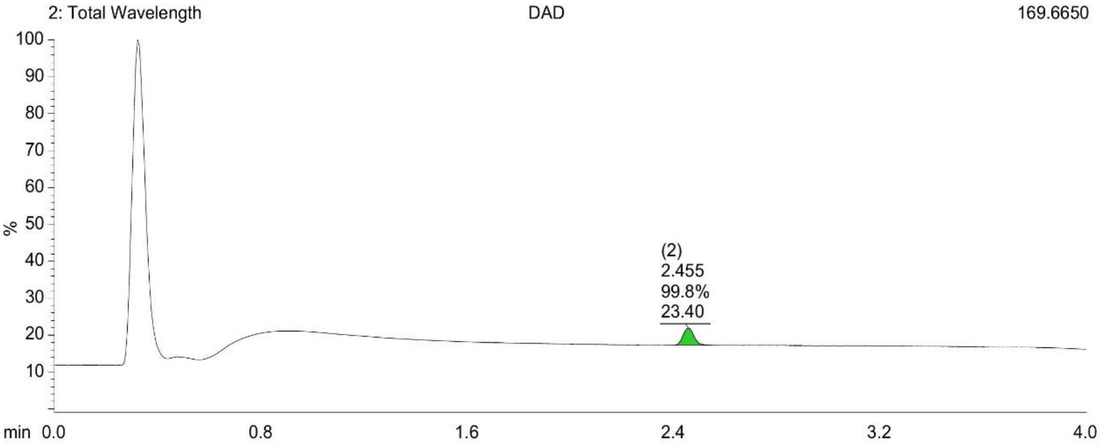

Analytical Studio Reviewer Report

Sample Name: 416682:01:03 **Acquired:** 7/22/2022 11:46 PM **Instrument:** Agilent TOF **Submitter:**
Location: 1,10:M,4 **Filename:** 1046305694-416682-01-03.d **User:** **Job Code:**

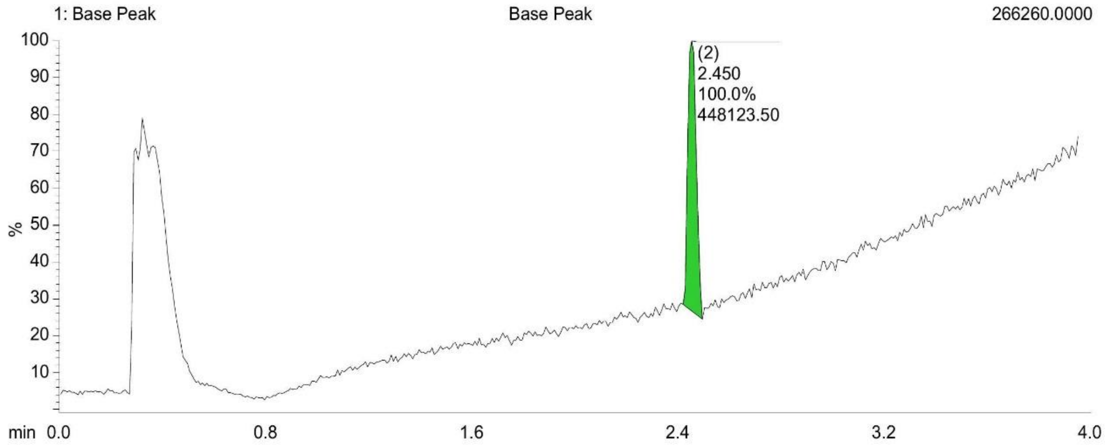

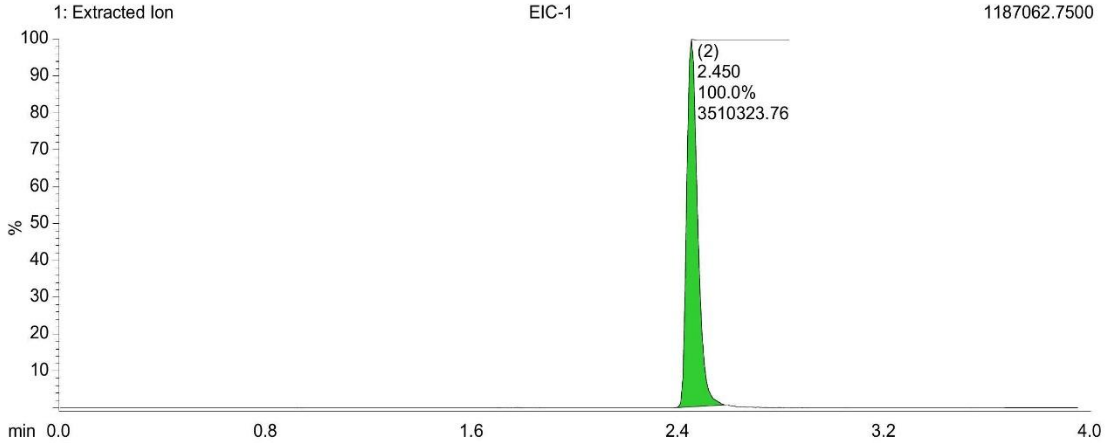

Analytical Studio Reviewer Report

Sample Name: 416682:01:03 **Acquired:** 7/22/2022 11:46 PM **Instrument:** Agilent TOF **Submitter:**
Location: 1,10:M,4 **Filename:** 1046305694-416682-01-03.d **User:** **Job Code:**

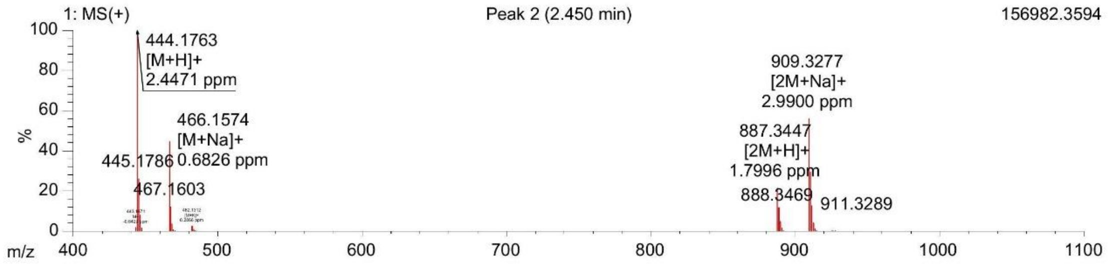

BPM	Error PPM	Error mDa	Target
444.1763	3.6666	3.3927	443.1…

HPLC traces (**15**)

Analytical Studio Reviewer Report

Sample Name: 417288:01:01 **Acquired:** 5/30/2023 11:43 PM **Instrument:** Agilent TOF **Submitter:**
Location: 1,1:C,1 **Filename:** 1046304981-417288-01-01.d **User:** **Job Code:**

				Area %						Area Abs					
Peak #	Time	Target ...	Found	TIC(+)	TIC(+)	TIC(+)	UV254	DAD	Base ...	TIC(+)	TIC(+)	TIC(+)	UV254	DAD	Base Peak
1	1.294	438.1726	NA	0.0	0.0	0.0	0.0	0.0	0.0	0	0	3.74E04	0	0	0
2	1.889	438.1726	NA	0.0	0.0	0.6	0.1	0.0	0.2	0	0	5.32E05	0.16	0	1.04E05
3	2.021	438.1726	NA	0.0	0.0	0.1	0.0	0.0	0.0	0	0	9.27E04	0	0	0
4	2.098	438.1726	Yes	0.0	0.0	99.2	99.8	100.0	99.8	0	0	8.76E07	123.02	118.37	6.34E07
5	2.310	438.1726	NA	0.0	0.0	0.0	0.1	0.0	0.0	0	0	0	0.12	0	0
6	2.510	438.1726	NA	0.0	0.0	0.0	0.0	0.0	0.0	0	0	4.19E04	0	0	0

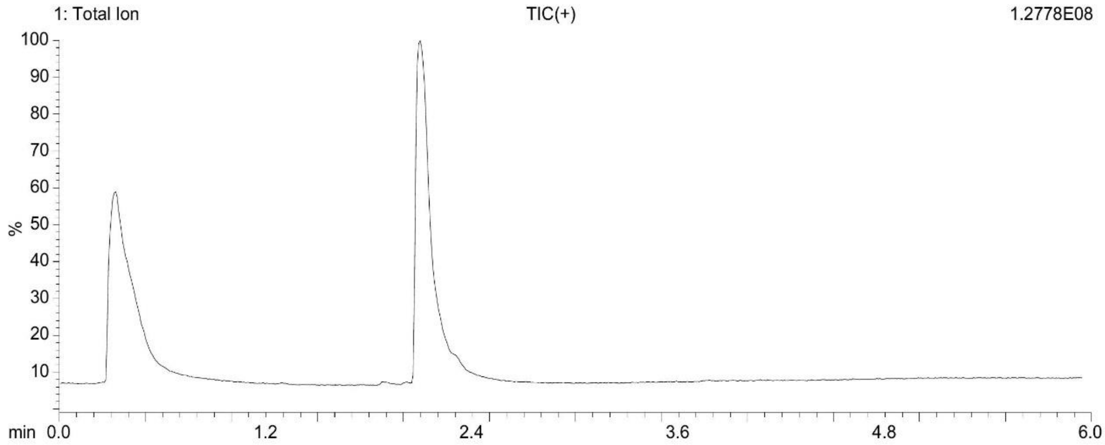

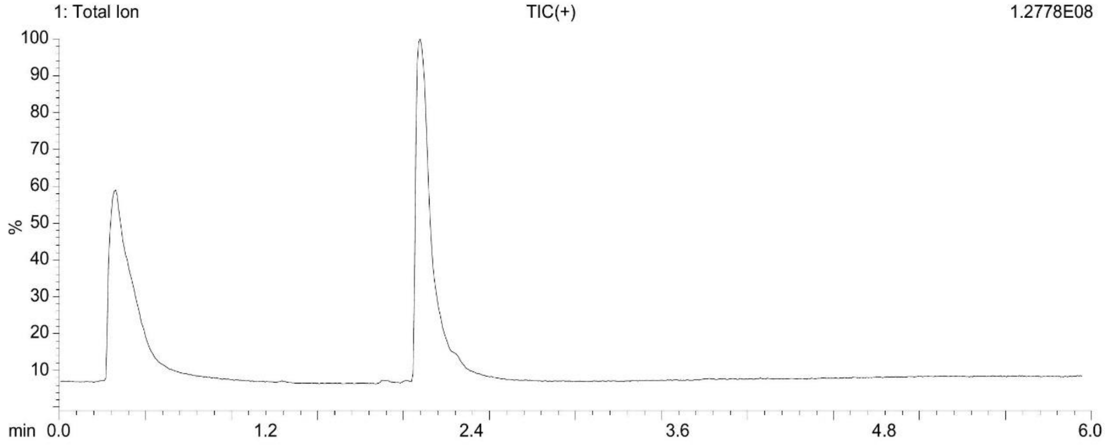

Page 1 of 5 Printed: 1/8/2024 3:18 PM

Analytical Studio Reviewer Report

Sample Name: 417288:01:01
Location: 1,1:C,1
Acquired: 5/30/2023 11:43 PM
Filename: 1046304981-417288-01-01.d
Instrument: Agilent TOF
User:
Submitter:
Job Code:

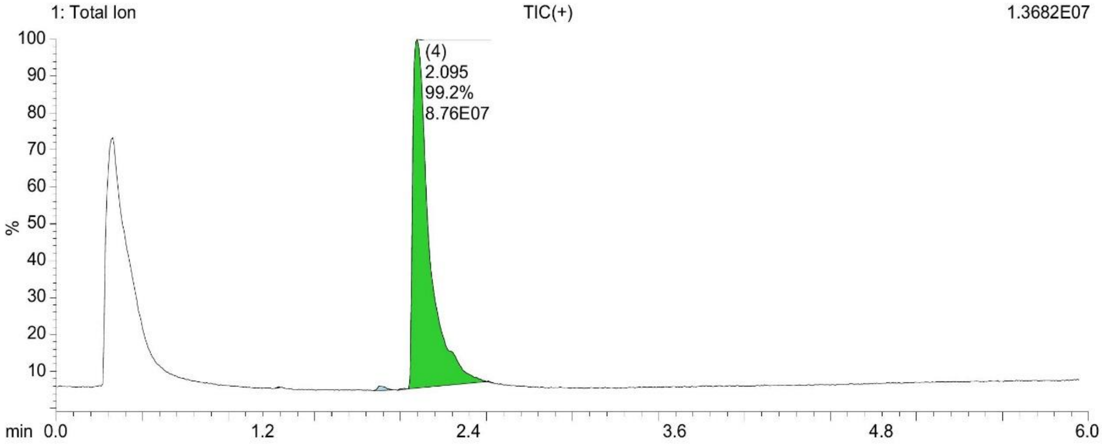

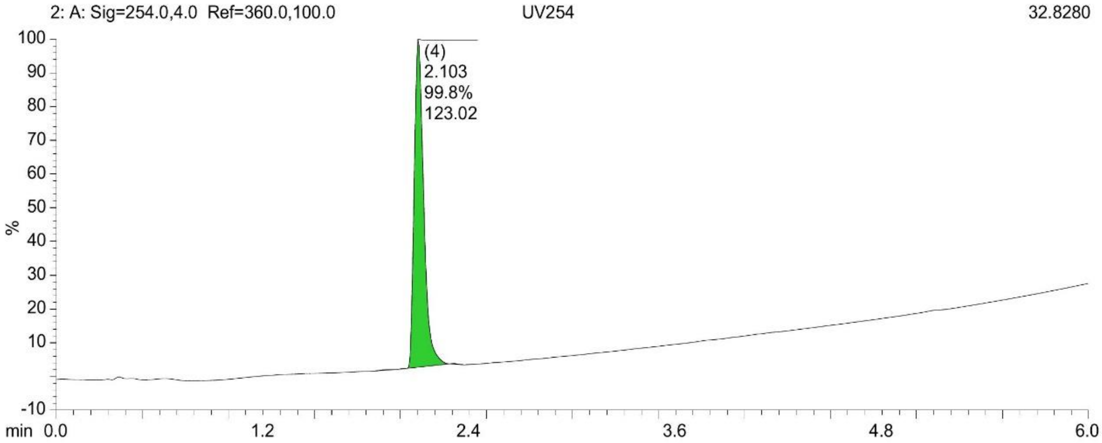

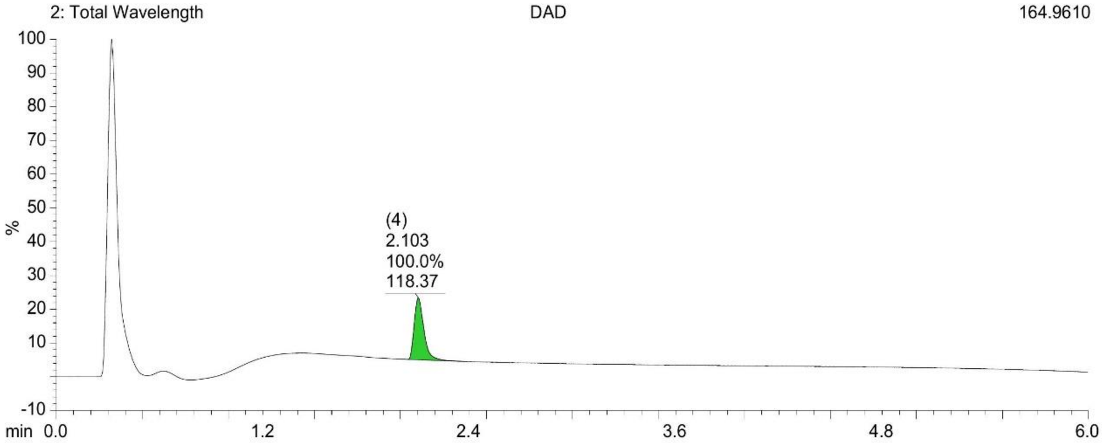

Printed: 1/8/2024 3:18 PM

Analytical Studio Reviewer Report

Sample Name: 417288:01:01 **Acquired:** 5/30/2023 11:43 PM **Instrument:** Agilent TOF **Submitter:**
Location: 1,1:C,1 **Filename:** 1046304981-417288-01-01.d **User:** **Job Code:**

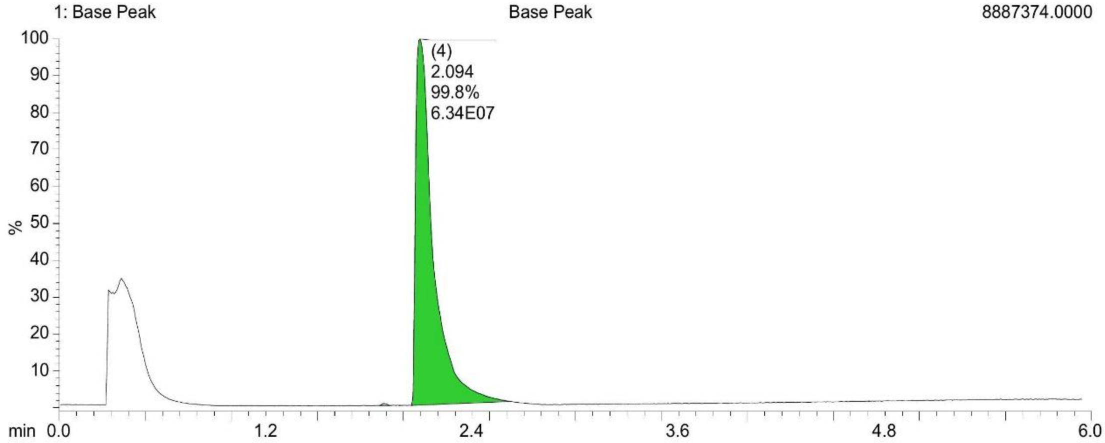

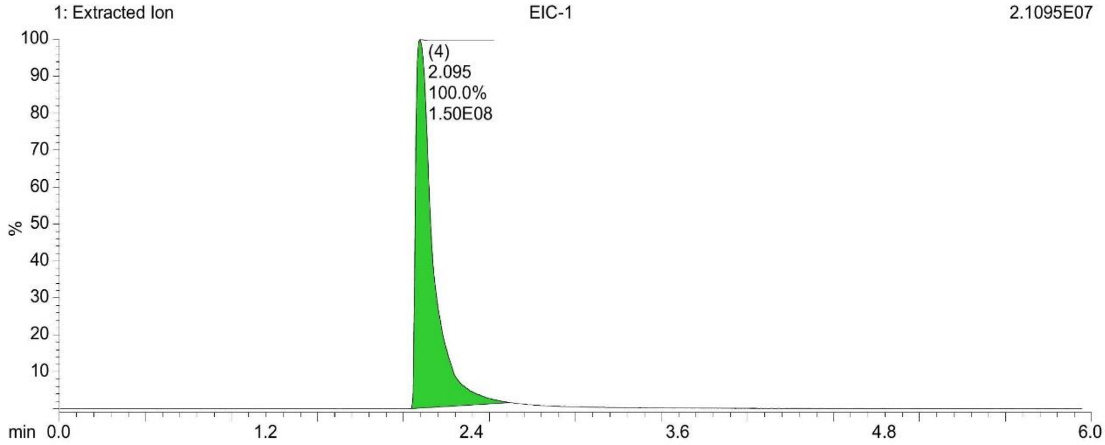

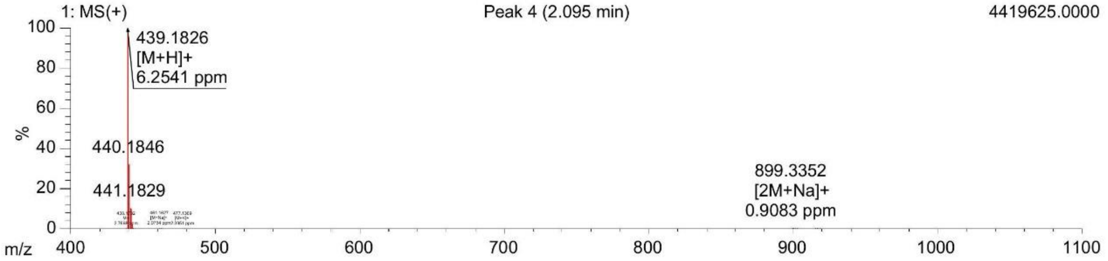

BPM	Error PPM	Error mDa	Target
439.1826	0.0280	0.0256	438.1…

HPLC traces (**16**)

Analytical Studio Reviewer Report

Sample Name: 416996:01:01 **Acquired:** 5/30/2023 11:24 PM **Instrument:** Agilent TOF **Submitter:**
Location: 1,1:A,1 **Filename:** 1046307889-416996-01-01.d **User:** **Job Code:**

				Area %						Area Abs					
Peak #	Time	Target ...	Found	TIC(+)	TIC(+)	TIC(+)	UV254	DAD	Base ...	TIC(+)	TIC(+)	TIC(+)	UV254	DAD	Base Peak
1	0.950	367.1757	NA	0.0	0.0	0.2	0.0	0.0	0.0	0	0	4.42E04	0	0	0
2	1.862	367.1757	NA	0.0	0.0	2.5	1.4	0.0	1.3	0	0	5.79E05	0.41	0	2.28E05
3	2.056	367.1757	NA	0.0	0.0	0.2	0.0	0.0	0.0	0	0	4.28E04	0	0	0
4	3.936	367.1757	Yes	0.0	0.0	96.9	97.7	99.1	98.7	0	0	2.27E07	28.73	14.11	1.73E07
5	4.286	367.1757	Yes	0.0	0.0	0.2	0.9	0.9	0.0	0	0	5.21E04	0.26	0.13	0

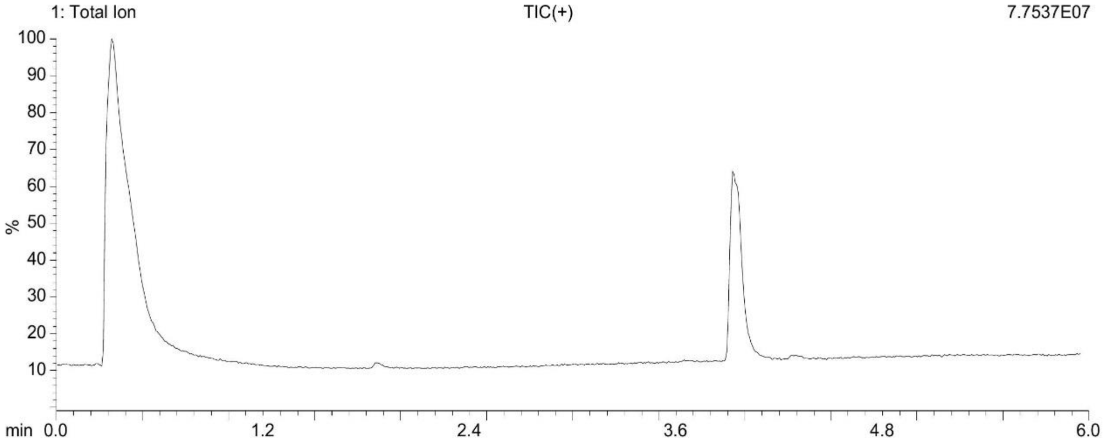

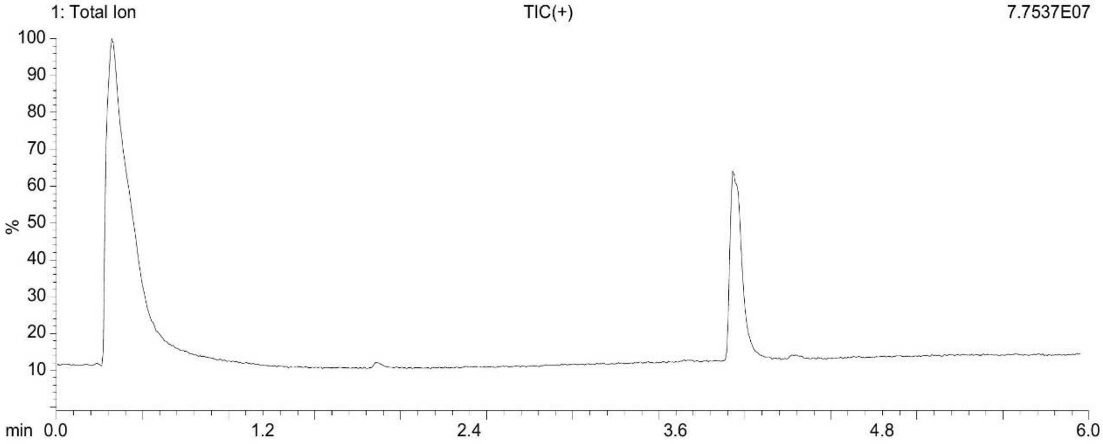

Analytical Studio Reviewer Report

Sample Name: 416996:01:01 **Acquired:** 5/30/2023 11:24 PM **Instrument:** Agilent TOF **Submitter:**
Location: 1,1:A,1 **Filename:** 1046307889-416996-01-01.d **User:** **Job Code:**

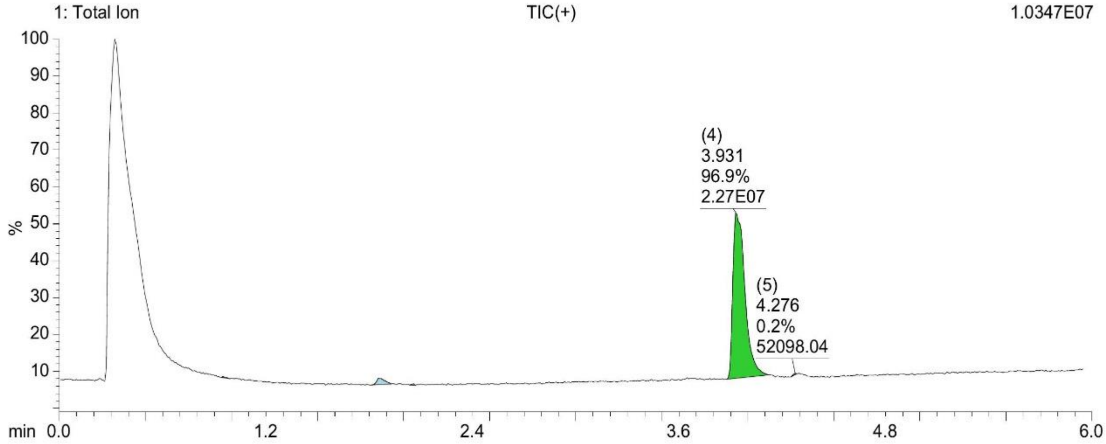

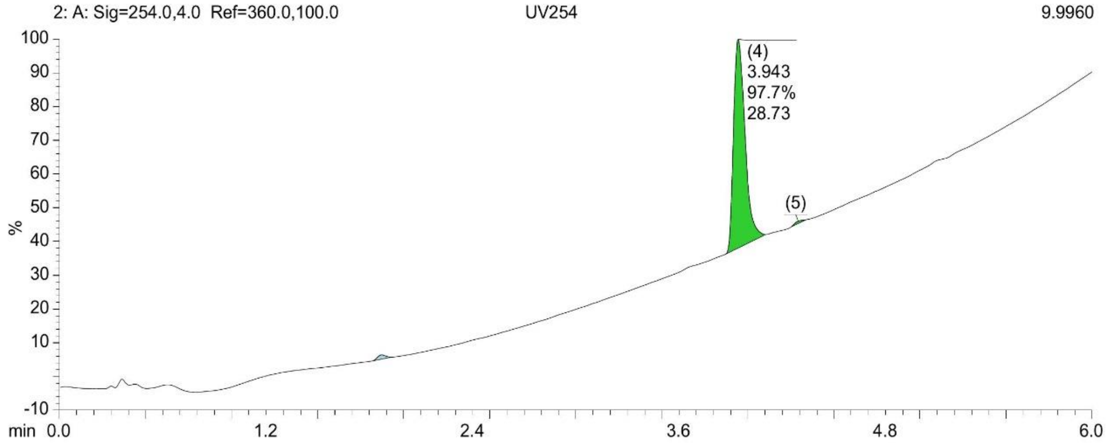

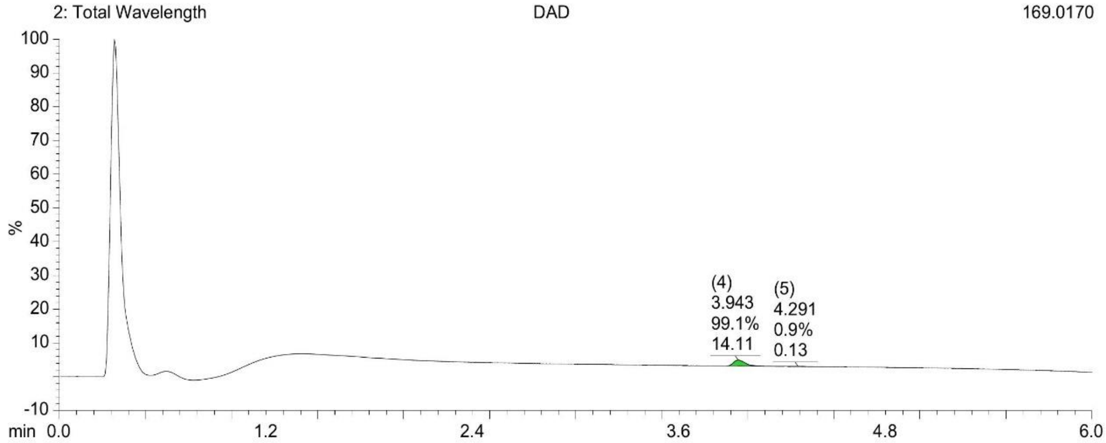

Analytical Studio Reviewer Report

Sample Name: 416996:01:01 **Acquired:** 5/30/2023 11:24 PM **Instrument:** Agilent TOF **Submitter:**
Location: 1,1:A,1 **Filename:** 1046307889-416996-01-01.d **User:** **Job Code:**

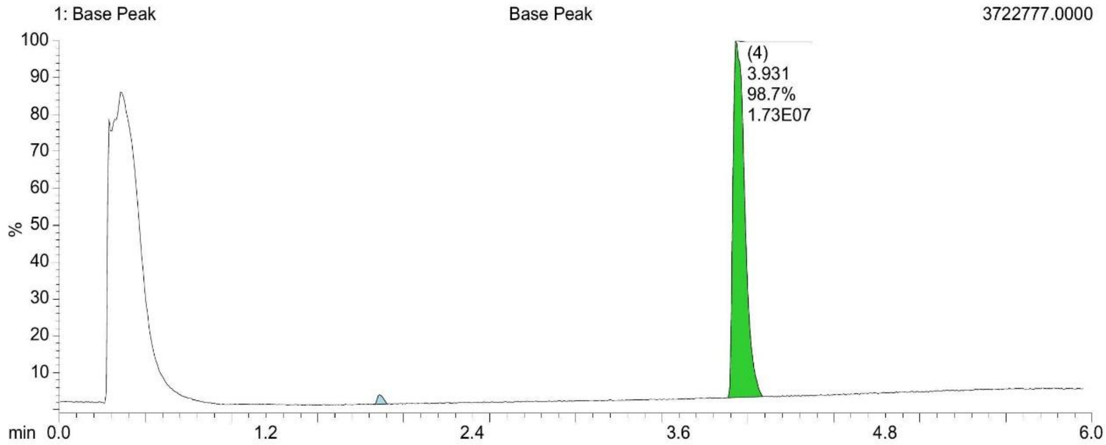

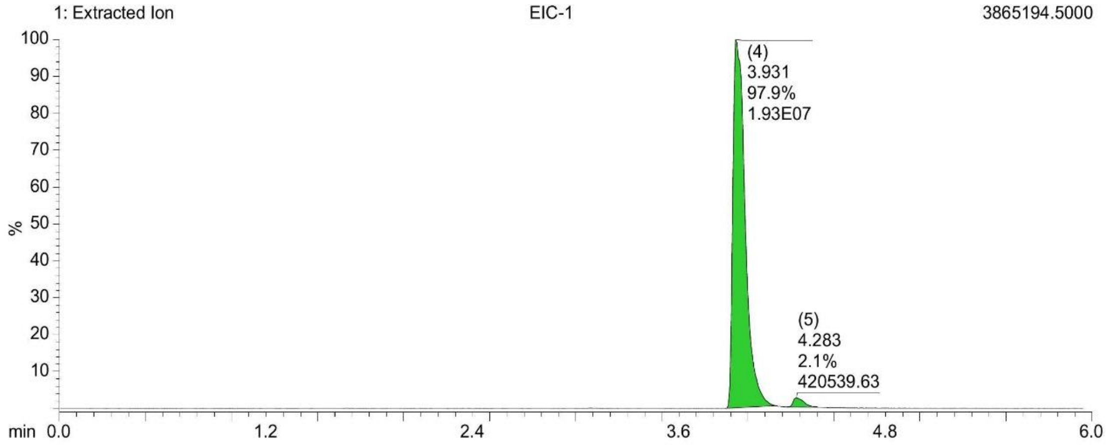

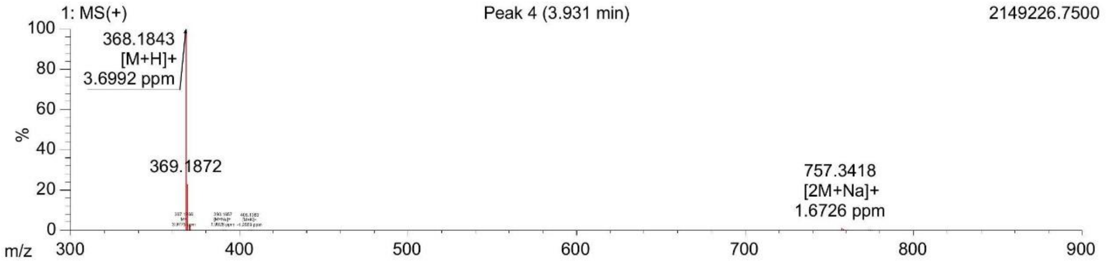

BPM	Error PPM	Error mDa	Target
368.1843	1.0545	0.8155	367.1…

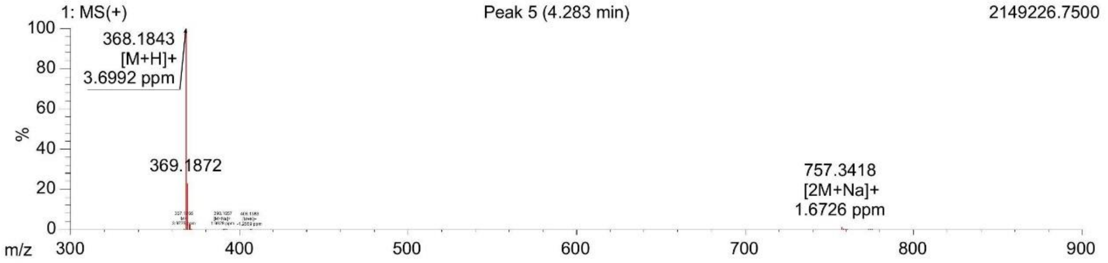

BPM	Error PPM	Error mDa	Target
368.1843	1.0545	0.8155	367.1…

HPLC traces (**17**)

Analytical Studio Reviewer Report

Sample Name: 417666:01:01 **Acquired:** 5/30/2023 11:33 PM **Instrument:** Agilent TOF **Submitter:**
Location: 1,1:A,3 **Filename:** 1046305780-417666-01-01.d **User:** **Job Code:**

				Area %						Area Abs					
Peak #	Time	Target ...	Found	TIC(+)	TIC(+)	TIC(+)	UV254	DAD	Base ...	TIC(+)	TIC(+)	TIC(+)	UV254	DAD	Base Peak
1	2.647	473.2427	NA	0.0	0.0	0.2	0.2	0.0	0.0	0	0	2.04E05	0.51	0	0
2	2.738	473.2427	NA	0.0	0.0	0.1	0.2	0.3	0.3	0	0	1.21E05	0.40	0.45	7.70E04
3	3.789	473.2427	NA	0.0	0.0	1.1	0.0	0.2	0.5	0	0	9.68E05	0	0.34	1.36E05
4	3.909	473.2427	Yes	0.0	0.0	98.5	99.6	99.4	99.3	0	0	8.87E07	208.15	141.10	2.87E07
5	5.408	473.2427	NA	0.0	0.0	0.0	0.0	0.0	0.0	0	0	3.48E04	0	0	0

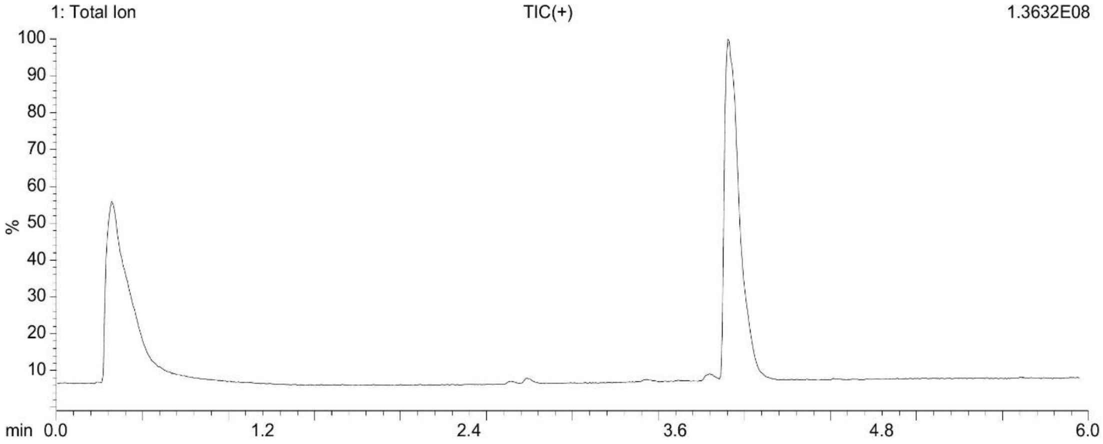

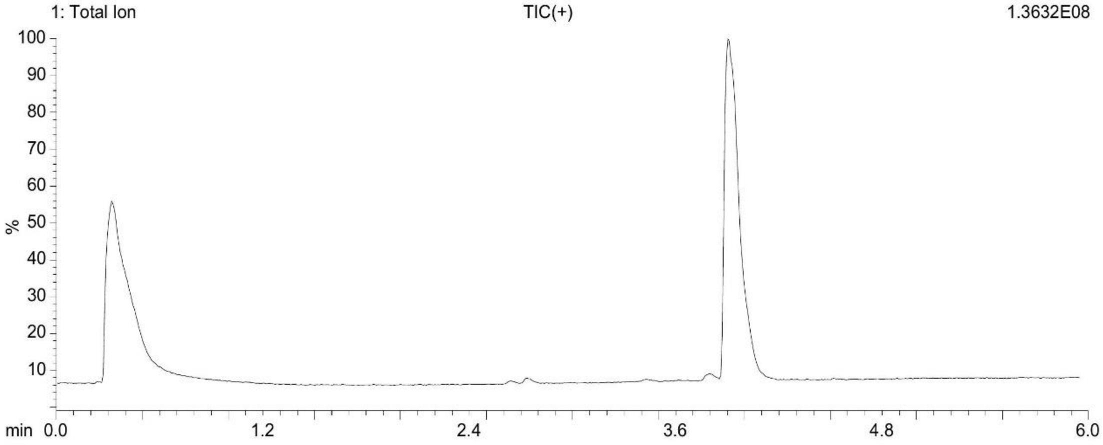

 Printed: 1/8/2024 3:18 PM

Analytical Studio Reviewer Report

Sample Name: 417666:01:01 **Acquired:** 5/30/2023 11:33 PM **Instrument:** Agilent TOF **Submitter:**
Location: 1,1:A,3 **Filename:** 1046305780-417666-01-01.d **User:** **Job Code:**

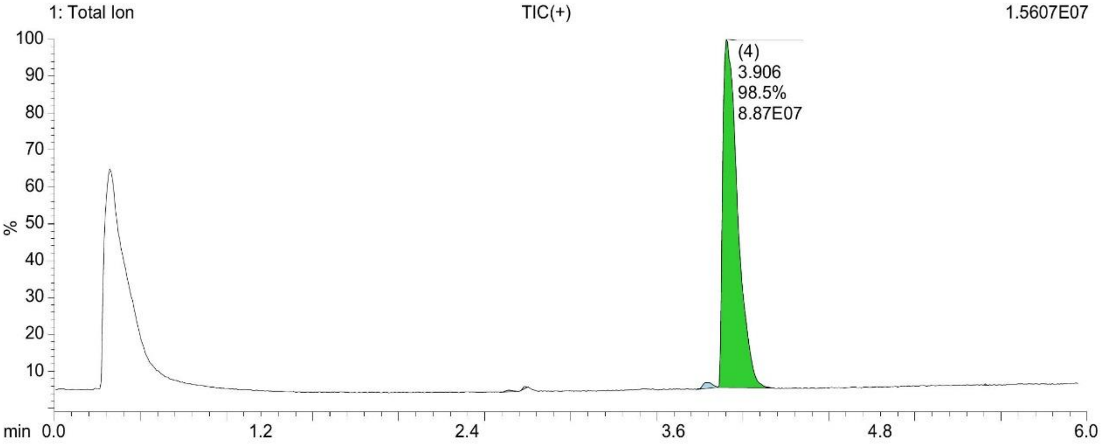

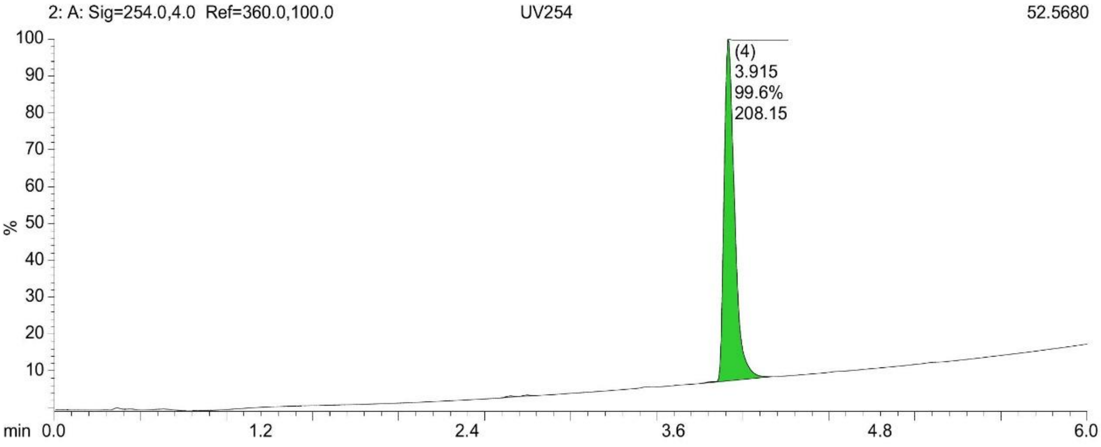

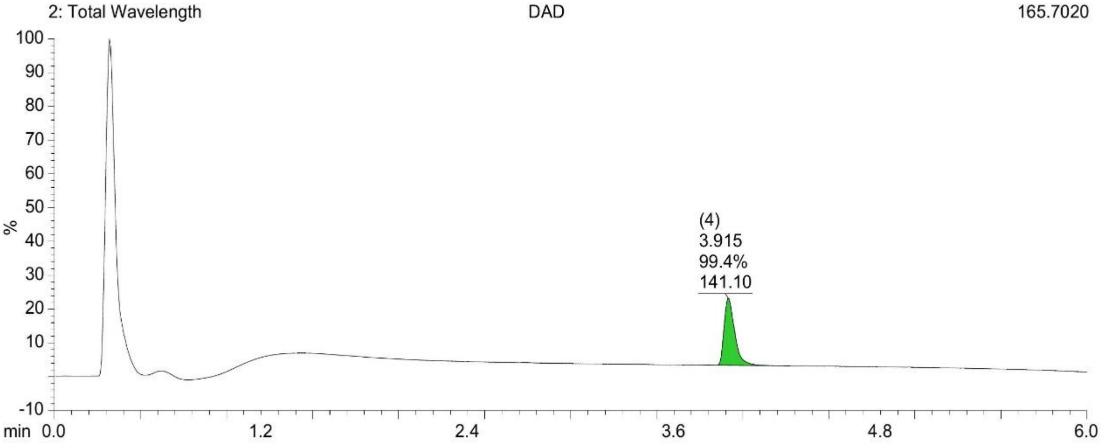

 Printed: 1/8/2024 3:18 PM

Analytical Studio Reviewer Report

Sample Name: 417666:01:01 **Acquired:** 5/30/2023 11:33 PM **Instrument:** Agilent TOF **Submitter:**
Location: 1,1:A,3 **Filename:** 1046305780-417666-01-01.d **User:** **Job Code:**

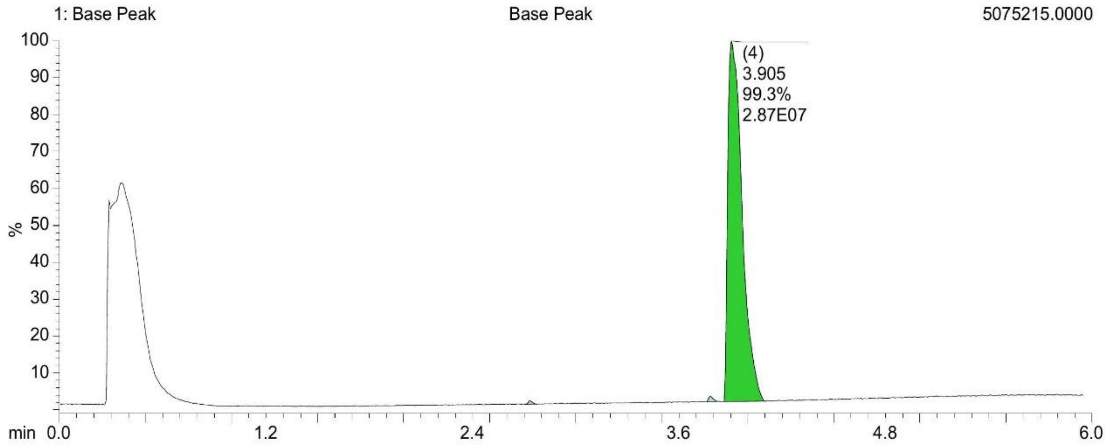

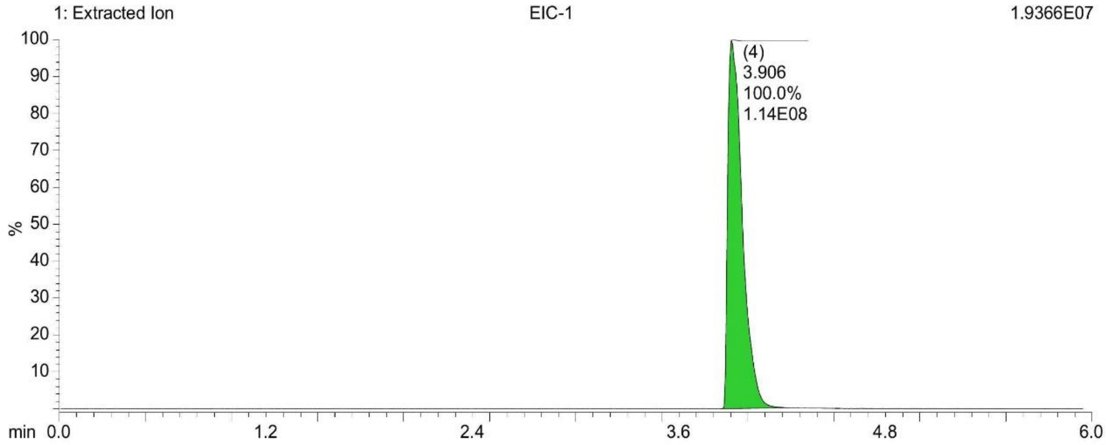

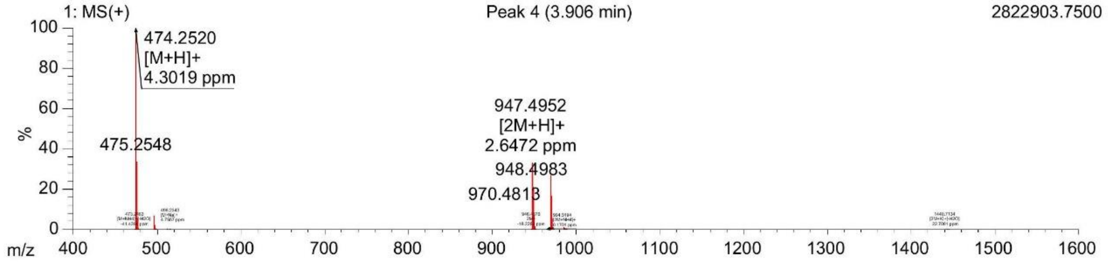

BPM	Error PPM	Error mDa	Target
474.2520	2.5037	3.6121	473.2…

HPLC traces (**18**)

Analytical Studio Reviewer Report

Sample Name: 414839:01:01 **Acquired:** 5/31/2023 12:11 AM **Instrument:** Agilent TOF **Submitter:**
Location: 1,1:E,3 **Filename:** 1046306579-414839-01-01.d **User:** **Job Code:**

				Area %						Area Abs					
Peak #	Time	Target ...	Found	TIC(+)	TIC(+)	TIC(+)	DAD	UV254	Base ...	TIC(+)	TIC(+)	TIC(+)	DAD	UV254	Base Peak
1	0.870	497.1430	NA	0.0	0.0	0.1	0.0	0.0	0.0	0	0	2.96E04	0	0	0
2	4.441	497.1430	Yes	0.0	0.0	0.0	0.0	0.0	0.0	0	0	0	0	0	0
3	4.586	497.1430	NA	0.0	0.0	0.9	0.0	0.0	0.0	0	0	2.34E05	0	0	0
4	4.800	497.1430	NA	0.0	0.0	0.1	0.0	0.0	0.0	0	0	2.95E04	0	0	0
5	4.940	497.1430	Yes	0.0	0.0	98.8	100.0	100.0	100.0	0	0	2.68E07	34.71	57.68	########
6	5.748	497.1430	NA	0.0	0.0	0.2	0.0	0.0	0.0	0	0	4.39E04	0	0	0

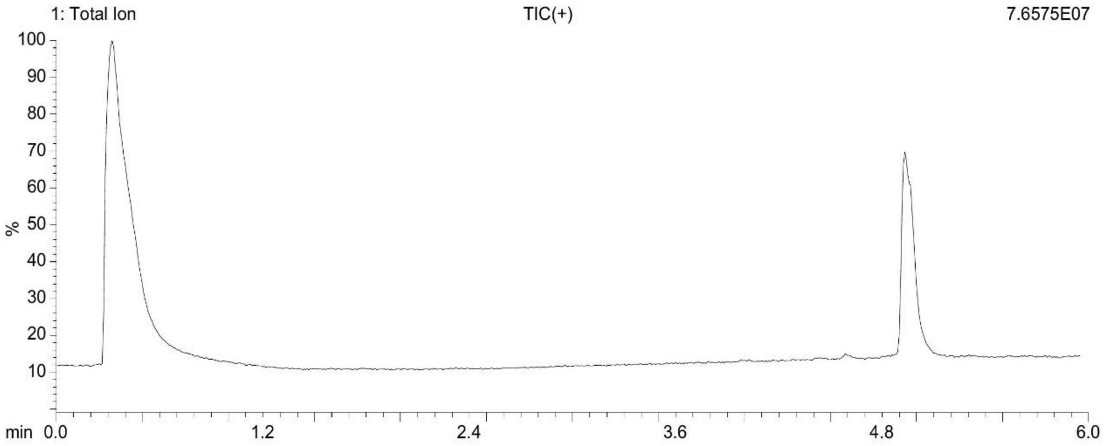

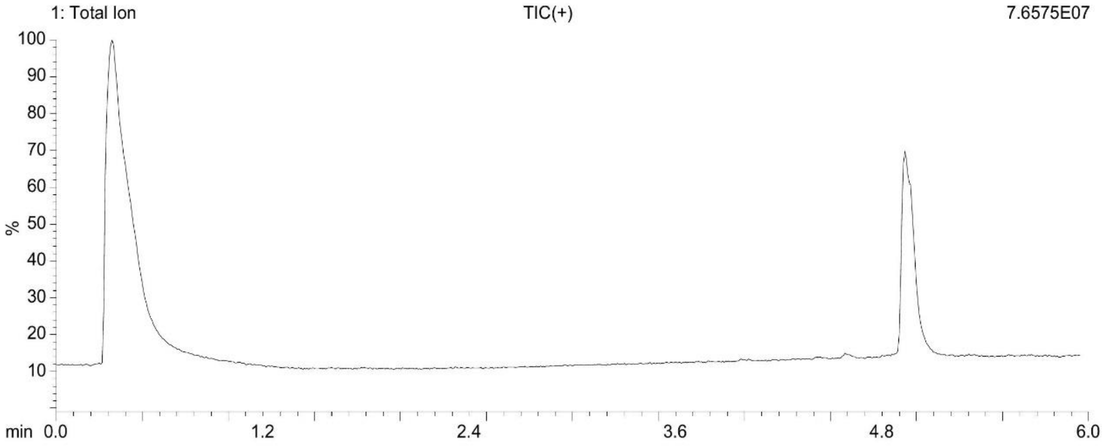

Analytical Studio Reviewer Report

Sample Name: 414839:01:01 **Acquired:** 5/31/2023 12:11 AM **Instrument:** Agilent TOF **Submitter:**
Location: 1,1:E,3 **Filename:** 1046306579-414839-01-01.d **User:** **Job Code:**

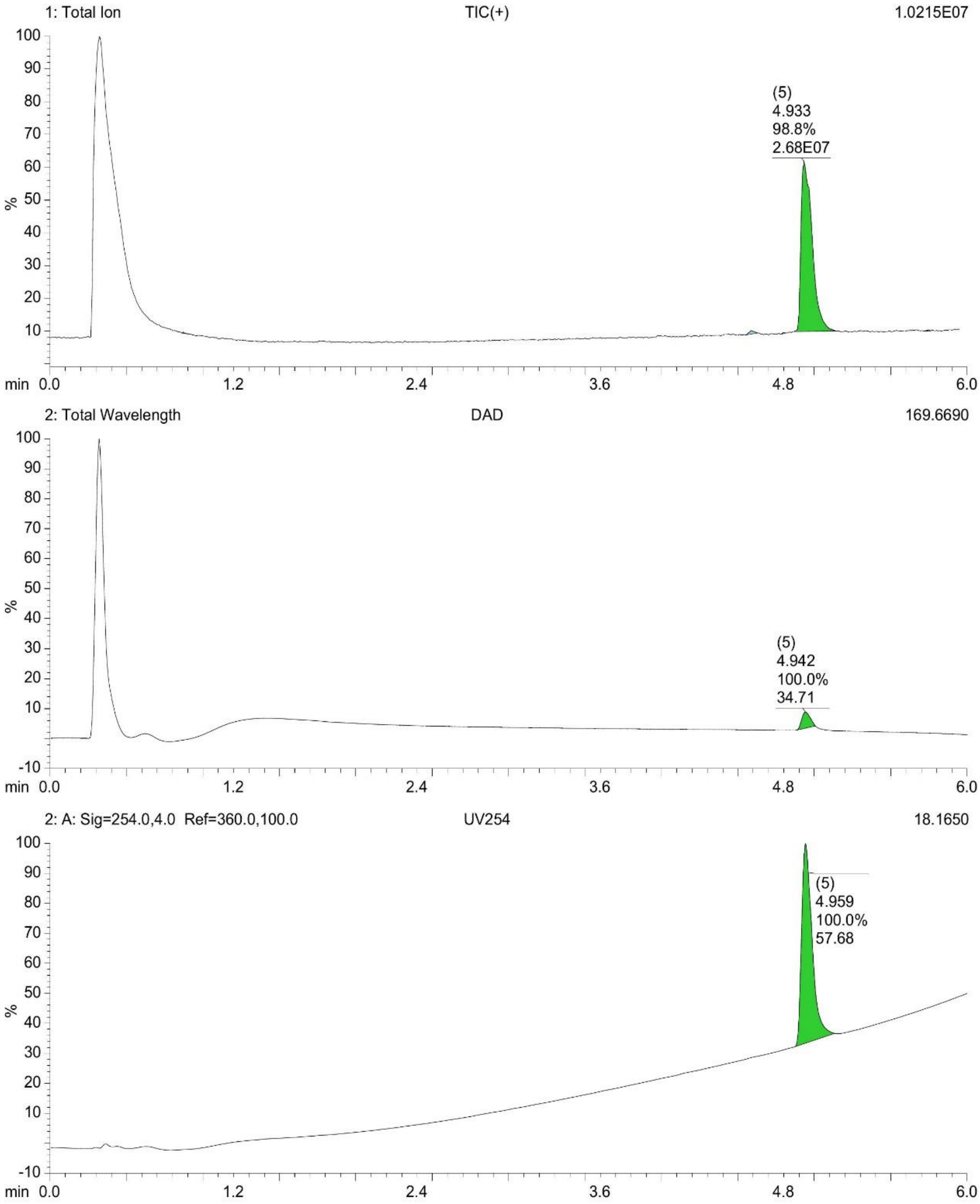

 Printed: 1/8/2024 3:18 PM

Analytical Studio Reviewer Report

Sample Name: 414839:01:01 **Acquired:** 5/31/2023 12:11 AM **Instrument:** Agilent TOF **Submitter:**
Location: 1,1:E,3 **Filename:** 1046306579-414839-01-01.d **User:** **Job Code:**

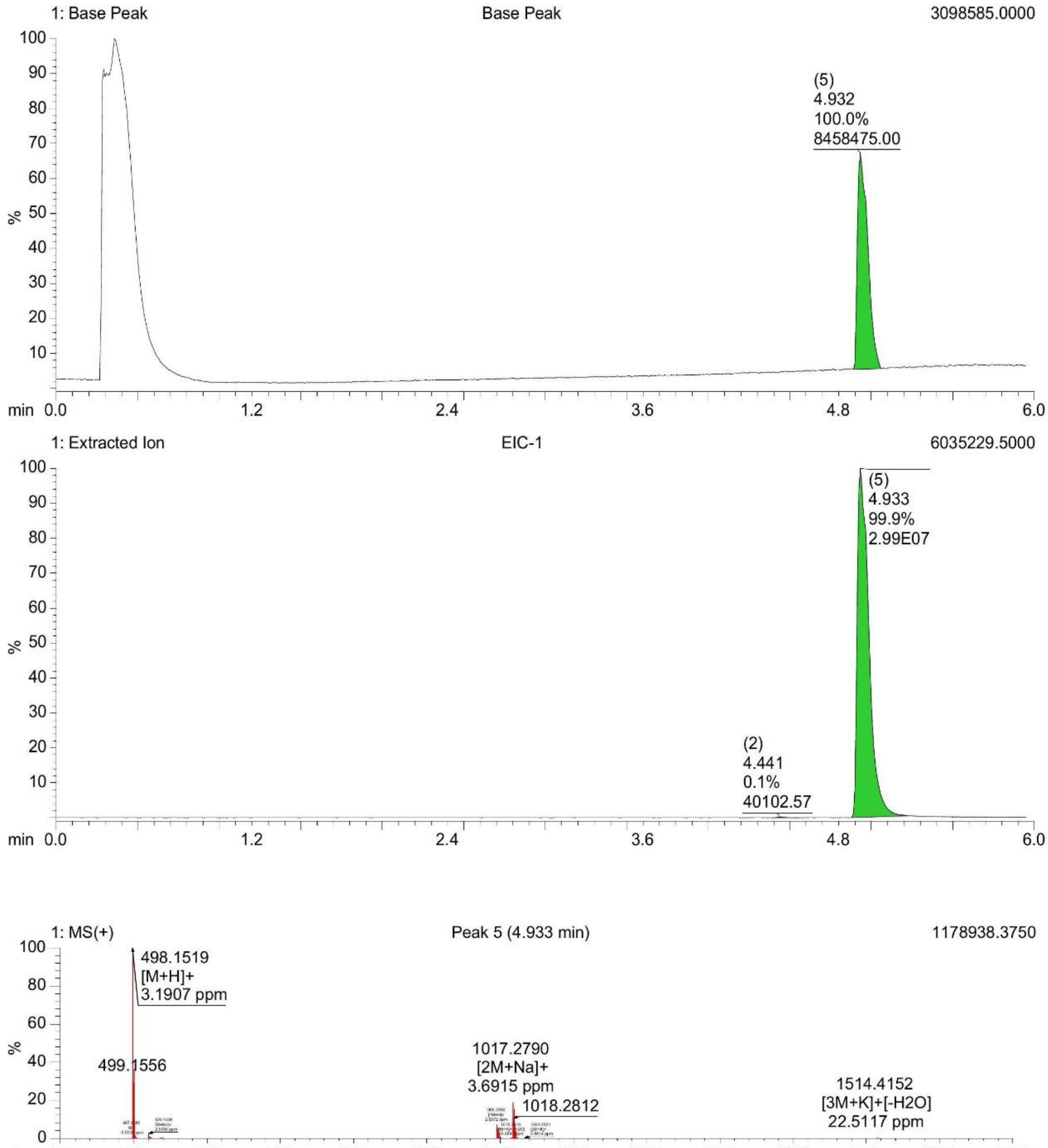

BPM	Error PPM	Error mDa	Target
498.1519	22.5117	34.0913	497.1…

HPLC traces (**19**)

Analytical Studio Reviewer Report

Sample Name: 415959:01:03 **Acquired:** 7/21/2022 6:57 AM **Instrument:** Agilent TOF **Submitter:**
Location: 1,9:K,4 **Filename:** 1046306221-415959-01-03.d **User:** **Job Code:**

				Area %						Area Abs					
Peak #	Time	Target ...	Found	TIC(+)	TIC(+)	TIC(+)	DAD	UV254	Base ...	TIC(+)	TIC(+)	TIC(+)	DAD	UV254	Base Peak
1	2.048	385.0942	NA	0.0	0.0	0.3	0.0	0.0	0.0	0	0	26715.29	0	0	0
2	2.746	385.0942	Yes	0.0	0.0	99.6	100.0	81.4	100.0	0	0	########	232.86	2.96	########
3	2.842	385.0942	NA	0.0	0.0	0.0	0.0	18.6	0.0	0	0	0	0	0.68	0
4	2.924	385.0942	Yes	0.0	0.0	0.0	0.0	0.0	0.0	0	0	0	0	0	0
5	2.943	385.0942	Yes	0.0	0.0	0.0	0.0	0.0	0.0	0	0	0	0	0	0
6	3.808	385.0942	NA	0.0	0.0	0.1	0.0	0.0	0.0	0	0	9253.80	0	0	0

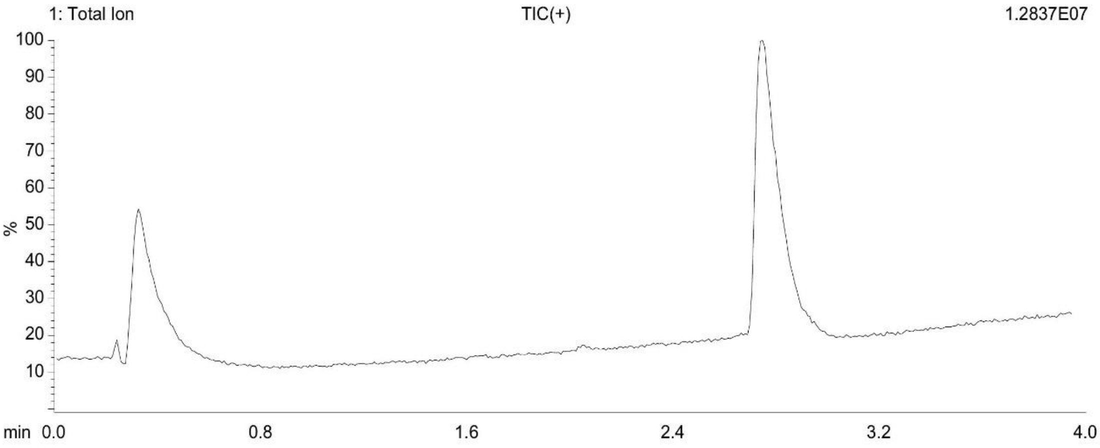

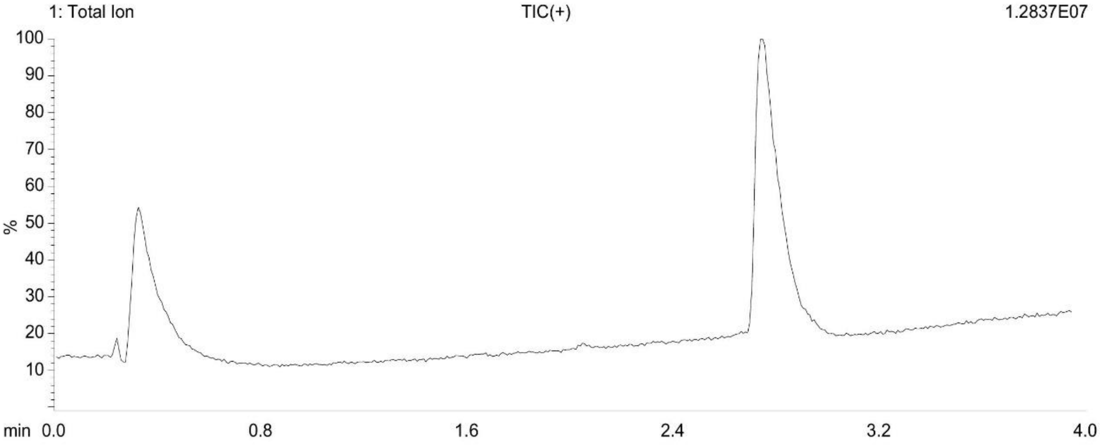

Analytical Studio Reviewer Report

Sample Name: 415959:01:03 **Acquired:** 7/21/2022 6:57 AM **Instrument:** Agilent TOF **Submitter:**
Location: 1,9:K,4 **Filename:** 1046306221-415959-01-03.d **User:** **Job Code:**

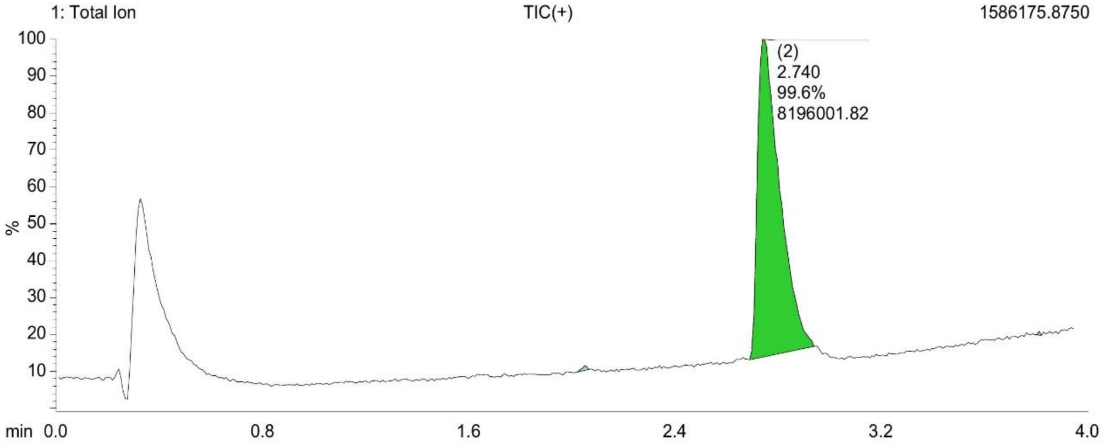

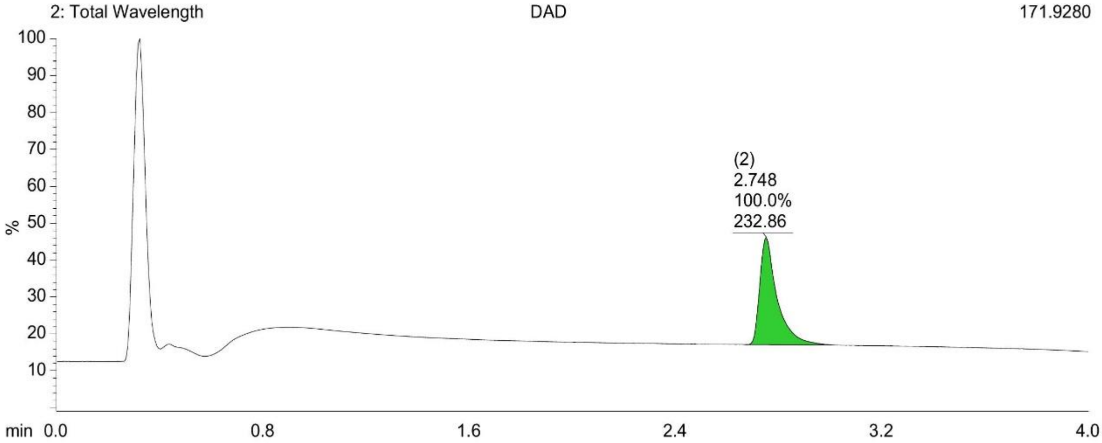

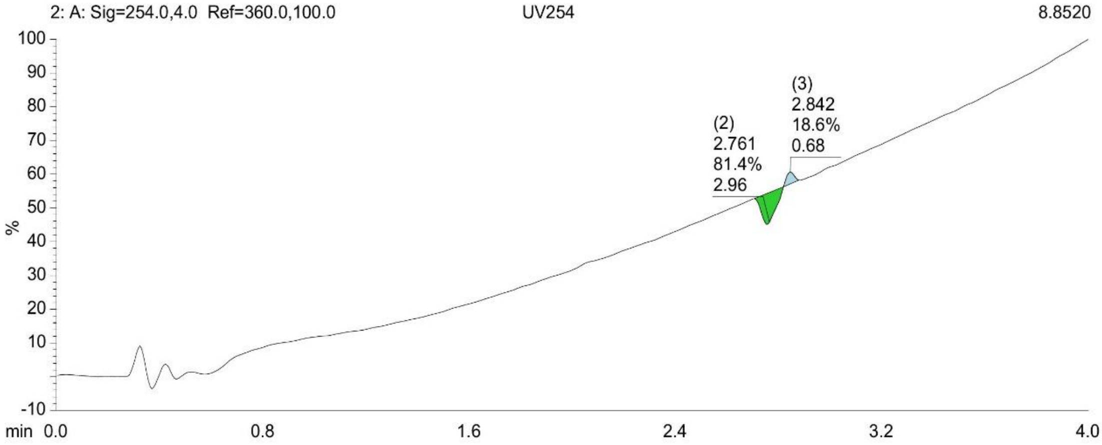

 Printed: 1/8/2024 3:19 PM

Analytical Studio Reviewer Report

Sample Name: 415959:01:03 **Acquired:** 7/21/2022 6:57 AM **Instrument:** Agilent TOF **Submitter:**
Location: 1,9:K,4 **Filename:** 1046306221-415959-01-03.d **User:** **Job Code:**

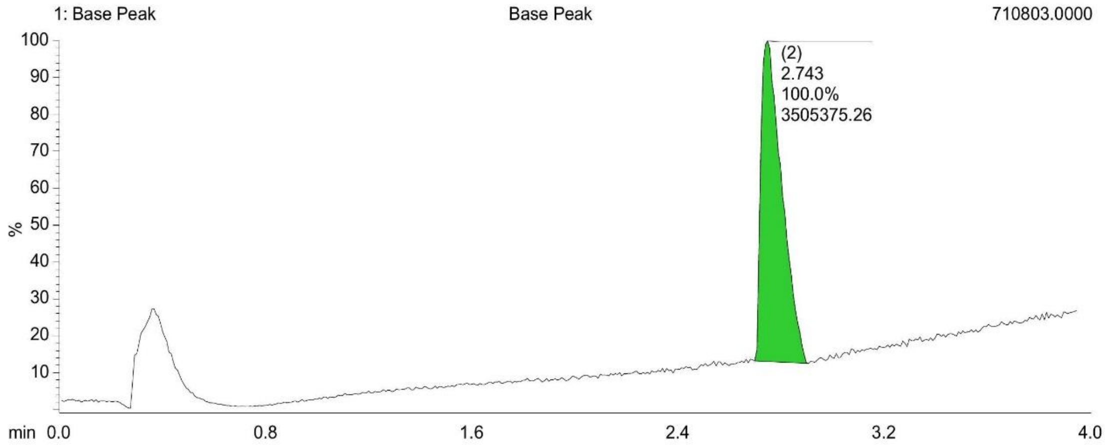

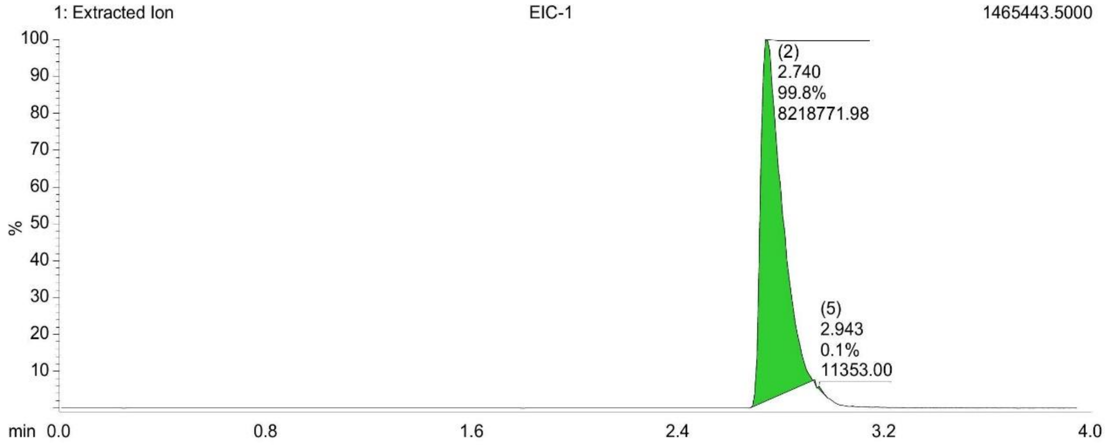

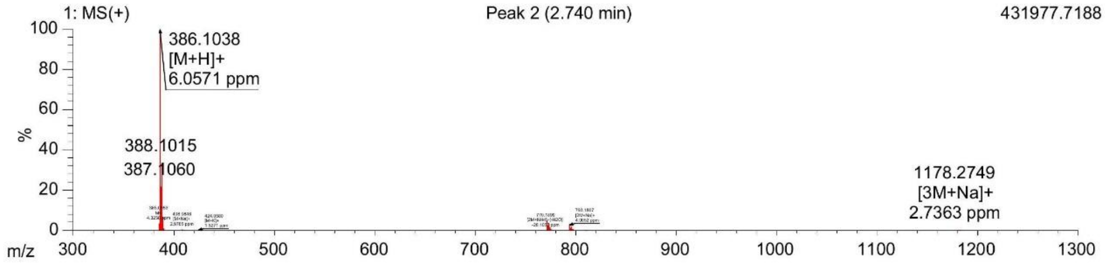

BPM	Error PPM	Error mDa	Target
386.1038	2.7363	3.2241	385.0…

HPLC traces (**20**)

Analytical Studio Reviewer Report

Sample Name: 416366:01:03 **Acquired:** 7/22/2022 8:21 AM **Instrument:** Agilent TOF **Submitter:**
Location: 1,10:G,13 **Filename:** 1046306218-416366-01-03.d **User:** **Job Code:**

				Area %						Area Abs					
Peak #	Time	Target ...	Found	TIC(+)	TIC(+)	TIC(+)	DAD	UV254	Base ...	TIC(+)	TIC(+)	TIC(+)	DAD	UV254	Base Peak
1	1.647	432.1962	NA	0.0	0.0	0.5	0.0	0.0	0.0	0	0	7.34E04	0	0	0
2	2.615	432.1962	NA	0.0	0.0	1.3	1.1	1.0	0.0	0	0	1.79E05	1.39	3.09	0
3	3.138	432.1962	Yes	0.0	0.0	0.0	0.4	0.0	0.0	0	0	0	0.47	0	0
4	3.303	432.1962	Yes	0.0	0.0	97.8	98.5	99.0	100.0	0	0	1.32E07	123.93	303.76	########
5	3.808	432.1962	NA	0.0	0.0	0.3	0.0	0.0	0.0	0	0	4.01E04	0	0	0

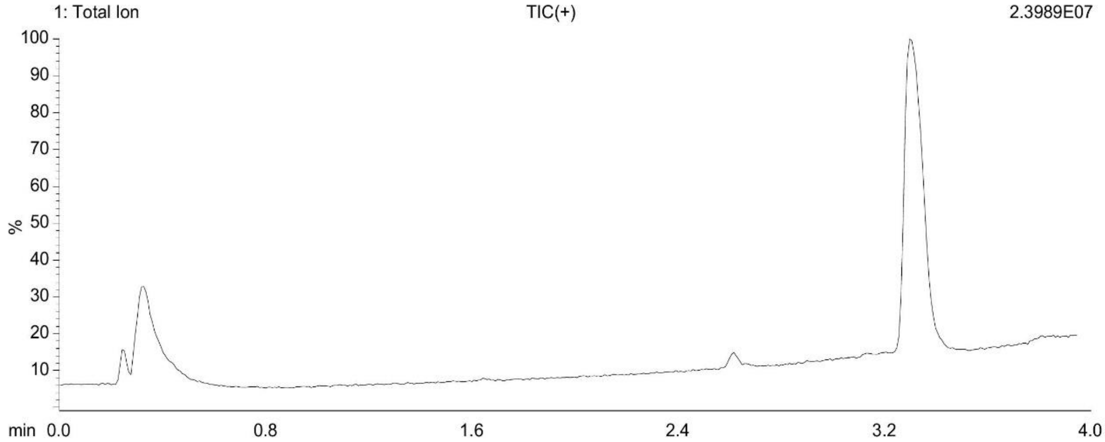

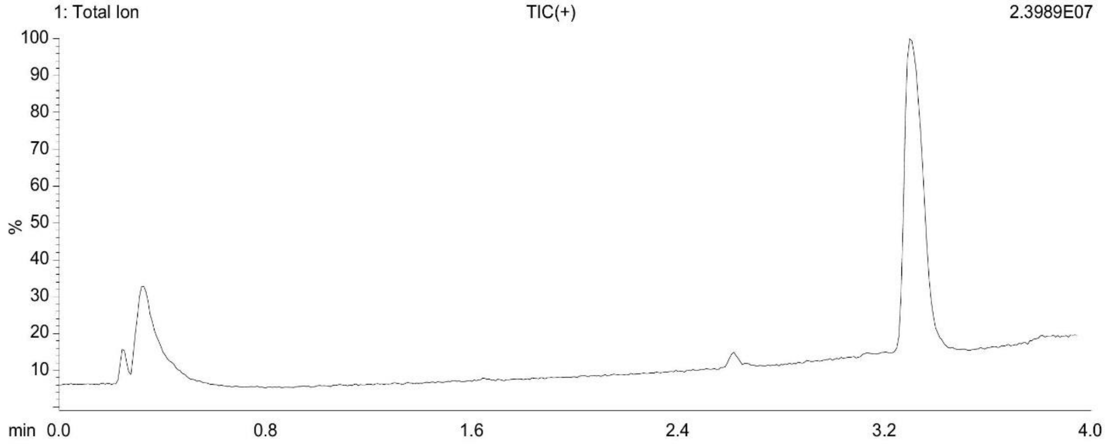

Analytical Studio Reviewer Report

Sample Name: 416366:01:03 **Acquired:** 7/22/2022 8:21 AM **Instrument:** Agilent TOF **Submitter:**
Location: 1,10:G,13 **Filename:** 1046306218-416366-01-03.d **User:** **Job Code:**

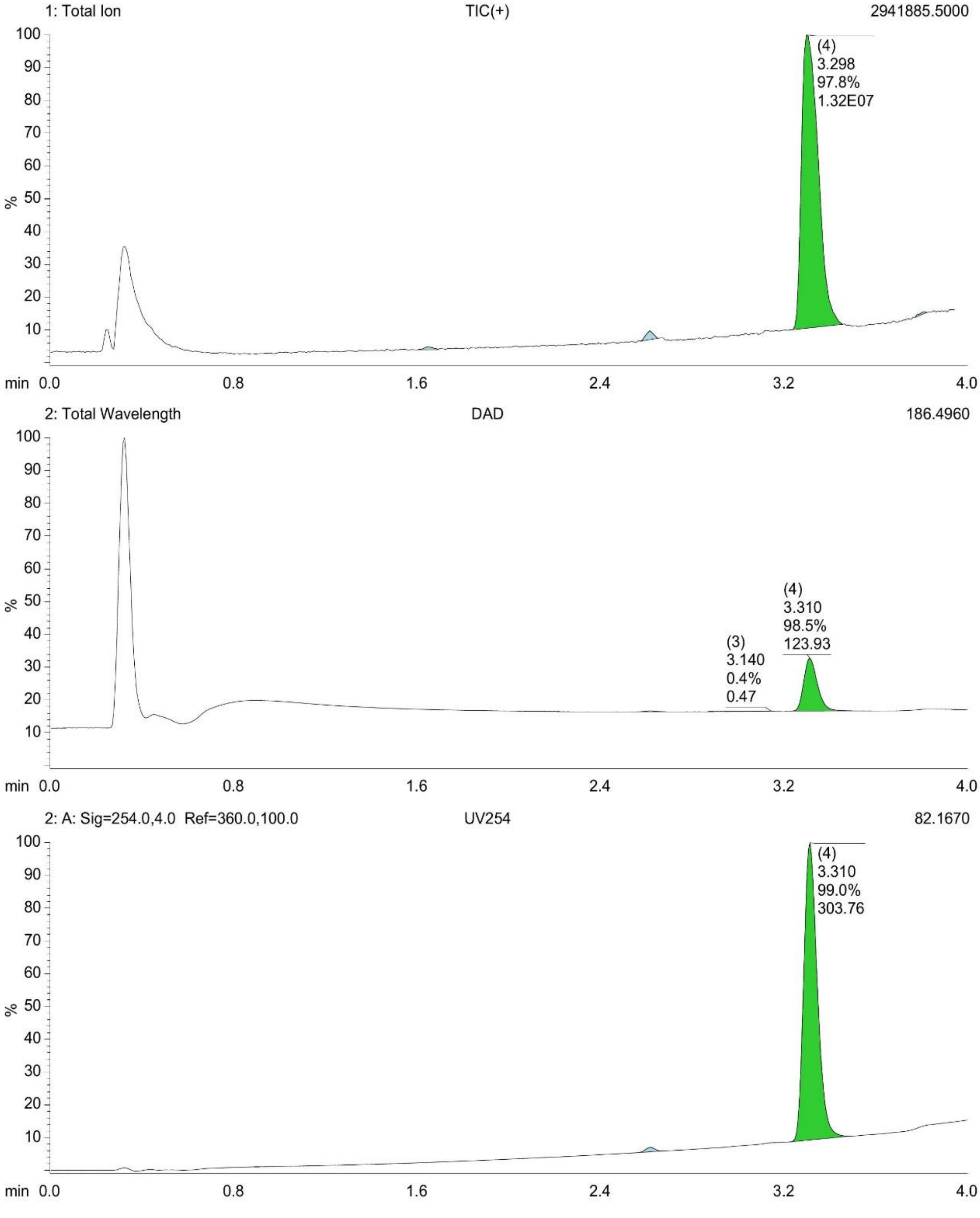

 Printed: 1/8/2024 3:19 PM

Analytical Studio Reviewer Report

Sample Name: 416366:01:03 **Acquired:** 7/22/2022 8:21 AM **Instrument:** Agilent TOF **Submitter:**
Location: 1,10:G,13 **Filename:** 1046306218-416366-01-03.d **User:** **Job Code:**

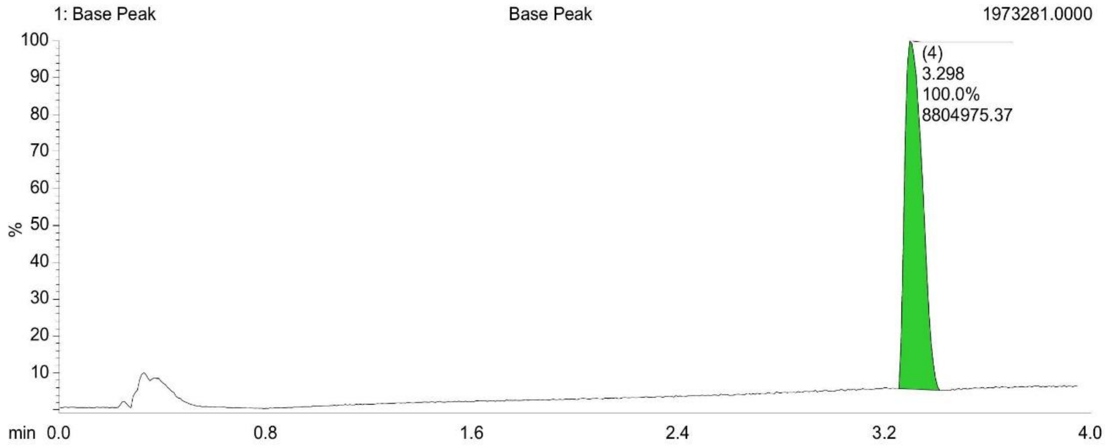

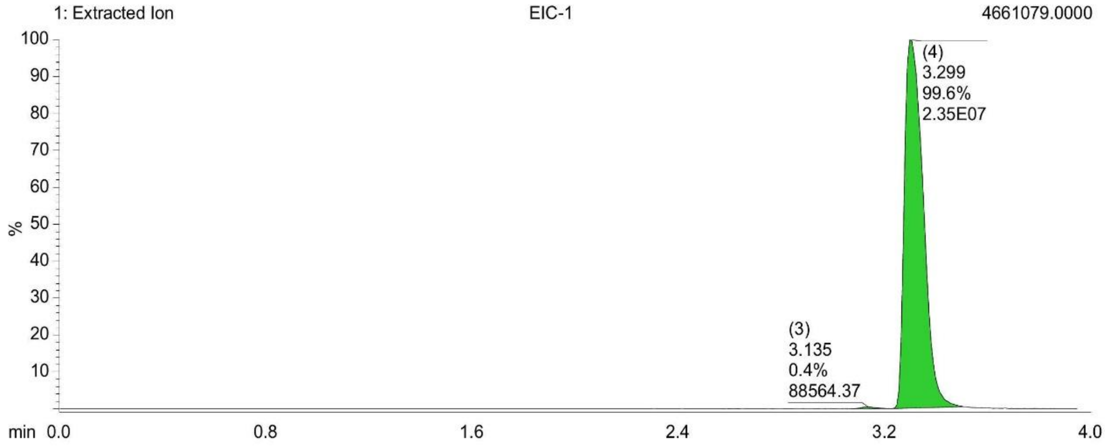

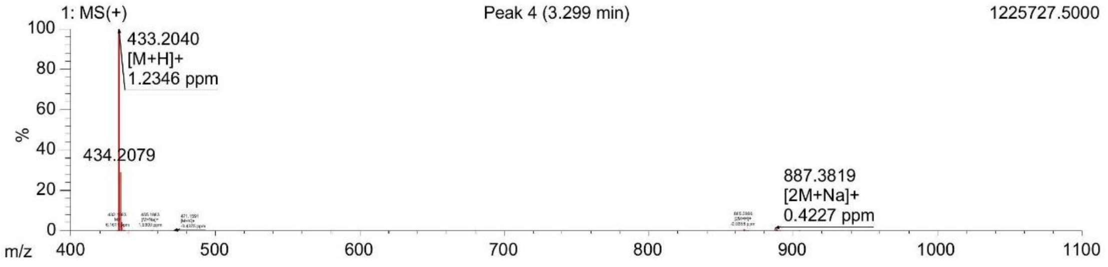

BPM	Error PPM	Error mDa	Target
433.2040	1.9315	1.7448	432.1…

HPLC traces (**21**)

Analytical Studio Reviewer Report

Sample Name: 418924:01:03 **Acquired:** 7/21/2022 4:36 AM **Instrument:** Agilent TOF **Submitter:**
Location: 1,9:I,8 **Filename:** 1046307180-418924-01-03.d **User:** **Job Code:**

				Area %						Area Abs					
Peak #	Time	Target ...	Found	TIC(+)	TIC(+)	TIC(+)	UV254	DAD	Base ...	TIC(+)	TIC(+)	TIC(+)	UV254	DAD	Base Peak
1	1.694	483.1158	NA	0.0	0.0	1.8	0.5	0.0	0.0	0	0	35324.27	0.78	0	0
2	2.604	483.1158	NA	0.0	0.0	0.0	0.4	0.5	0.0	0	0	0	0.74	0.32	0
3	2.707	483.1158	Yes	0.0	0.0	94.7	98.4	98.2	100.0	0	0	########	164.21	59.77	23839.70
4	2.832	483.1158	NA	0.0	0.0	3.5	0.7	1.2	0.0	0	0	69899.33	1.09	0.75	0

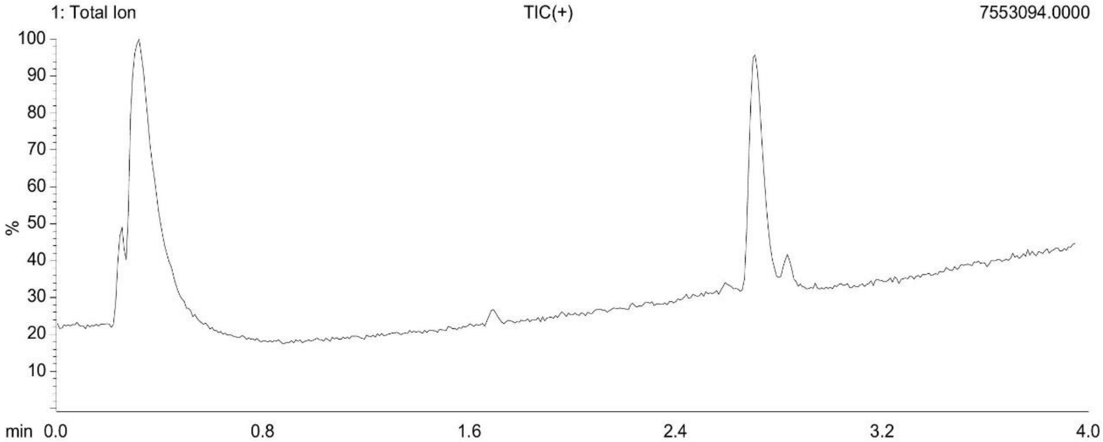

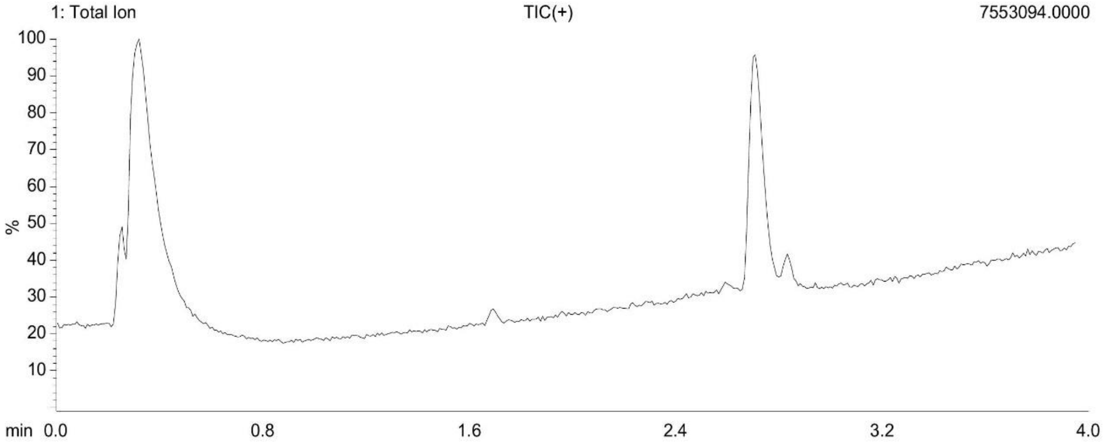

Analytical Studio Reviewer Report

Sample Name: 418924:01:03 **Acquired:** 7/21/2022 4:36 AM **Instrument:** Agilent TOF **Submitter:**
Location: 1,9:I,8 **Filename:** 1046307180-418924-01-03.d **User:** **Job Code:**

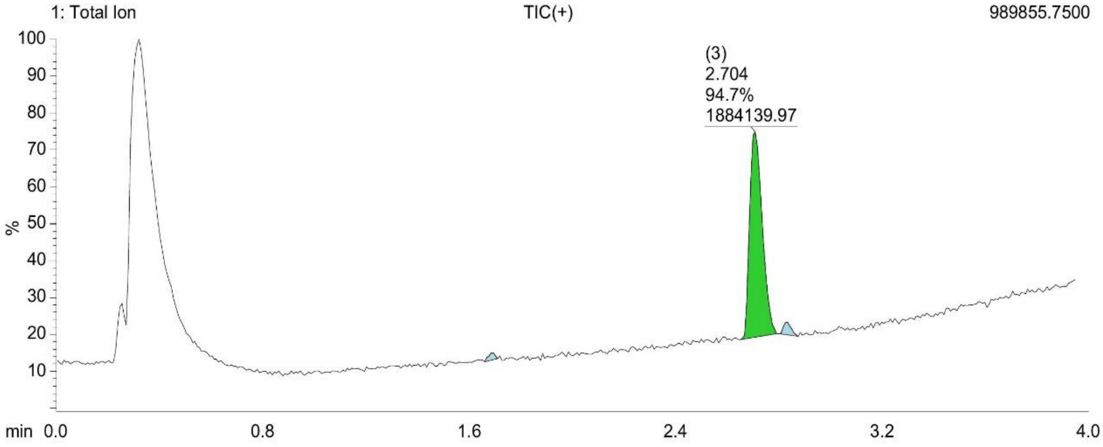

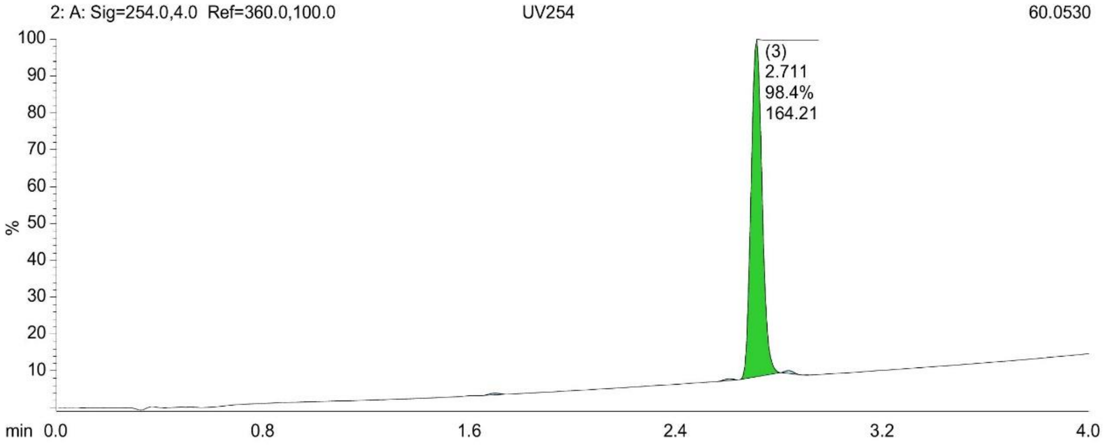

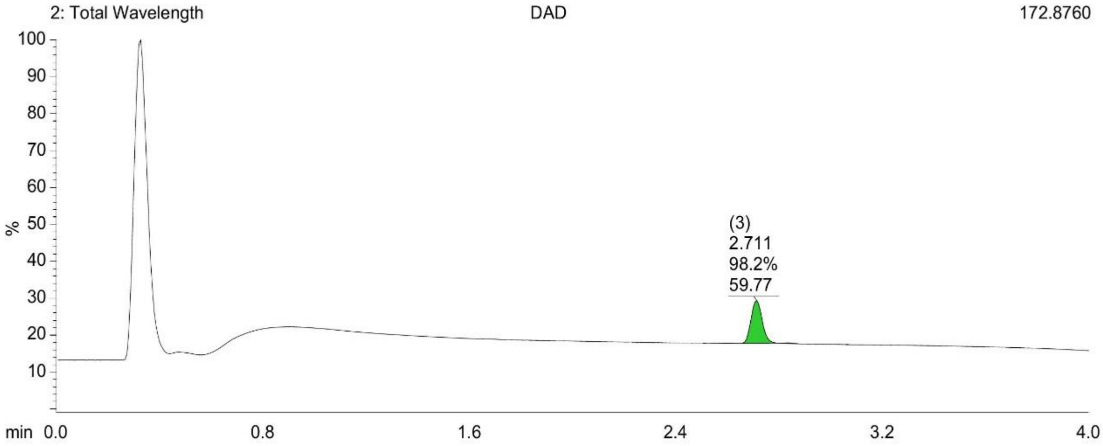

Analytical Studio Reviewer Report

Sample Name: 418924:01:03 **Acquired:** 7/21/2022 4:36 AM **Instrument:** Agilent TOF **Submitter:**
Location: 1,9:I,8 **Filename:** 1046307180-418924-01-03.d **User:** **Job Code:**

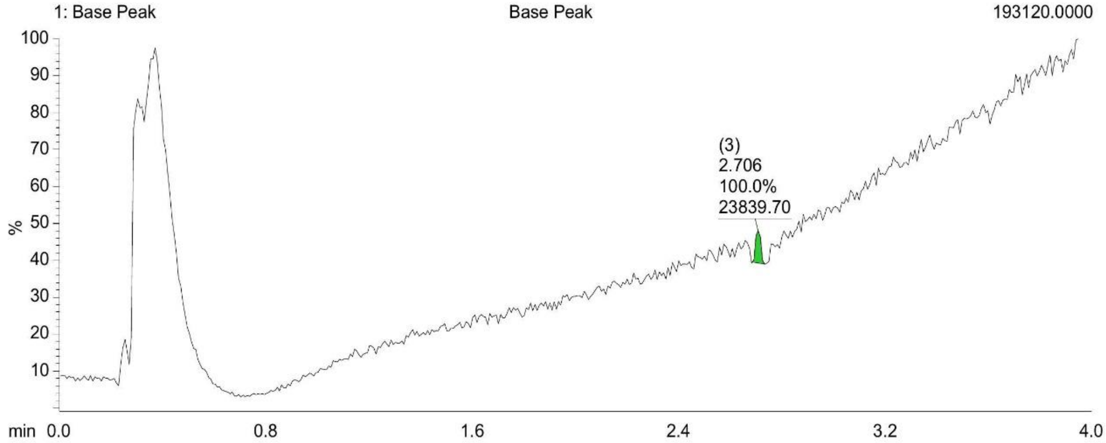

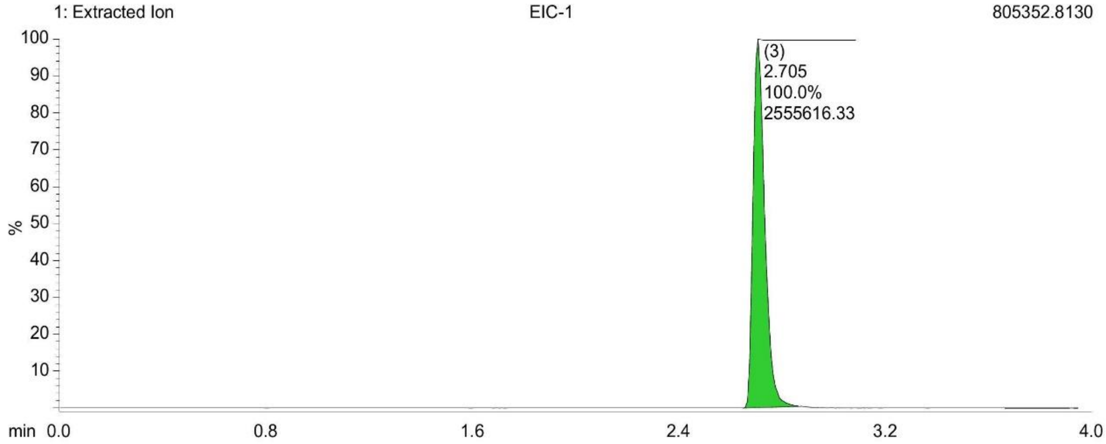

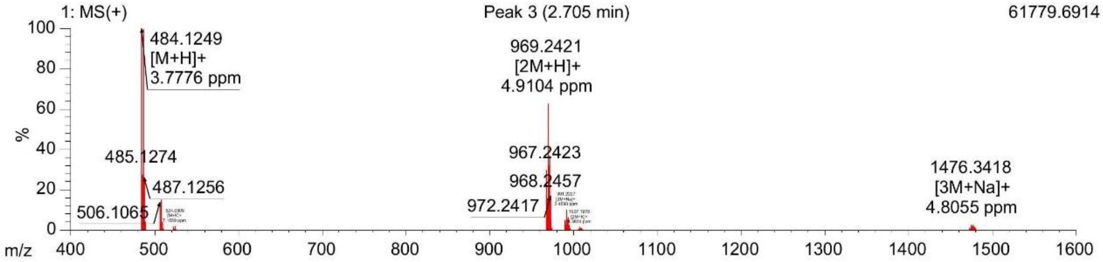

BPM	Error PPM	Error mDa	Target
484.1249	4.8055	7.0945	484.1…

HPLC traces (**22**)

Analytical Studio Reviewer Report

Sample Name: 414998:01:01 **Acquired:** 5/31/2023 12:49 AM **Instrument:** Agilent TOF **Submitter:**
Location: 1,1:K,1 **Filename:** 1046305730-414998-01-01.d **User:** **Job Code:**

				Area %						Area Abs					
Peak #	Time	Target ...	Found	TIC(+)	TIC(+)	TIC(+)	UV254	DAD	Base ...	TIC(+)	TIC(+)	TIC(+)	UV254	DAD	Base Peak
1	2.190	380.1736	NA	0.0	0.0	0.7	0.2	0.1	0.3	0	0	3.45E05	0.17	0.05	5.30E04
2	2.826	380.1736	NA	0.0	0.0	3.6	0.6	0.3	0.5	0	0	1.70E06	0.63	0.18	7.64E04
3	3.286	380.1736	NA	0.0	0.0	0.1	0.0	0.0	0.0	0	0	4.53E04	0	0	0
4	4.616	380.1736	NA	0.0	0.0	0.1	0.0	0.0	0.0	0	0	7.14E04	0	0	0
5	4.866	380.1736	Yes	0.0	0.0	95.4	99.2	99.6	99.1	0	0	4.56E07	99.01	55.21	1.67E07
6	5.658	380.1736	NA	0.0	0.0	0.0	0.0	0.0	0.1	0	0	0	0	0	1.66E04
7	5.825	380.1736	NA	0.0	0.0	0.0	0.0	0.0	0.0	0	0	2.10E04	0	0	0

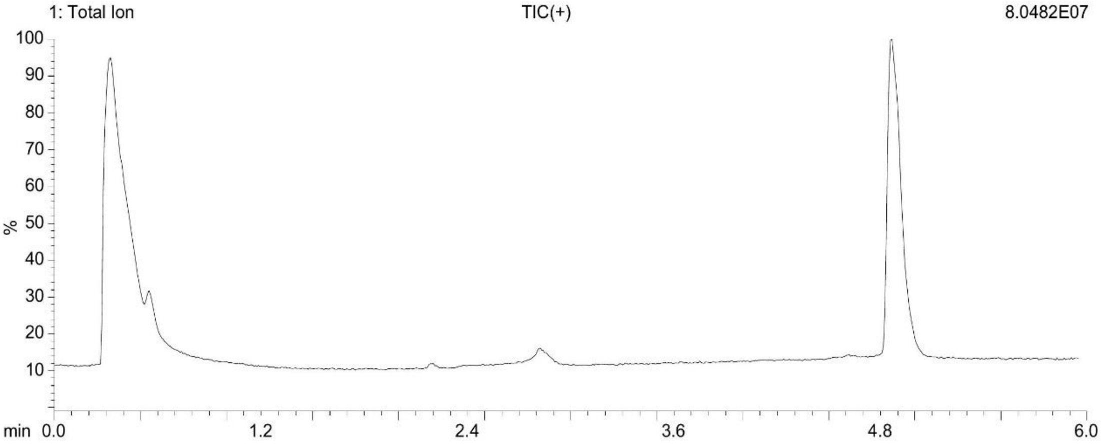

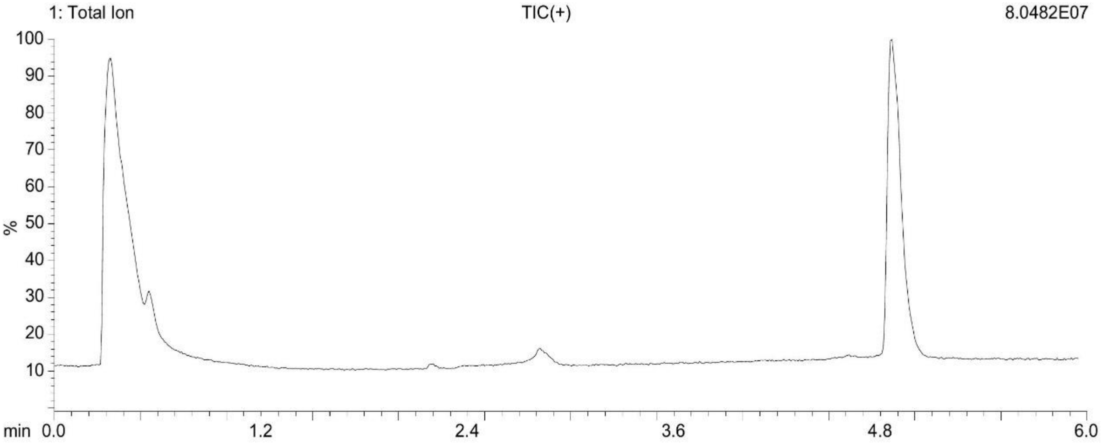

Analytical Studio Reviewer Report

Sample Name: 414998:01:01
Location: 1,1:K,1
Acquired: 5/31/2023 12:49 AM
Filename: 1046305730-414998-01-01.d
Instrument: Agilent TOF
User:
Submitter:
Job Code:

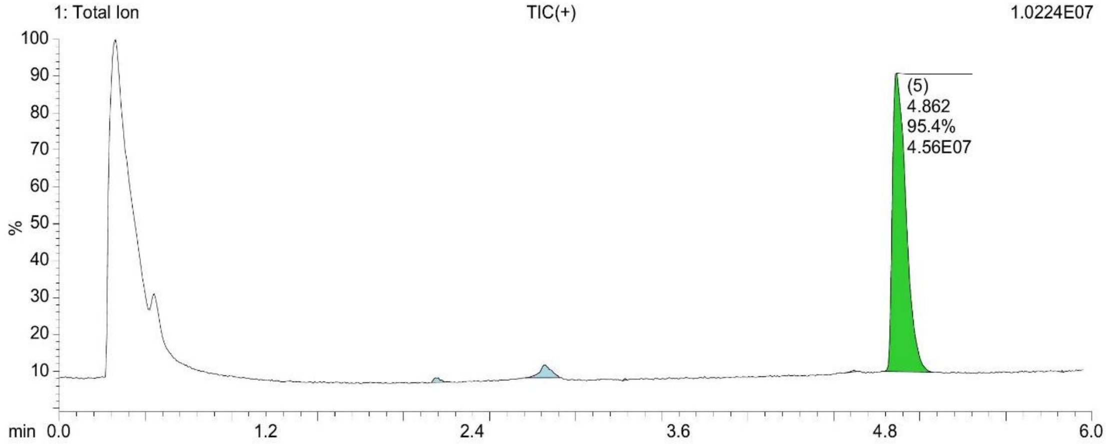

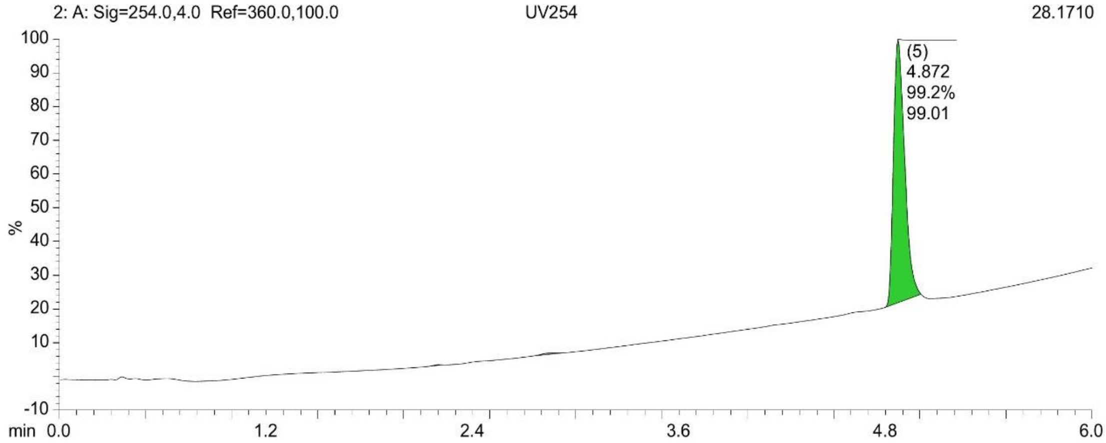

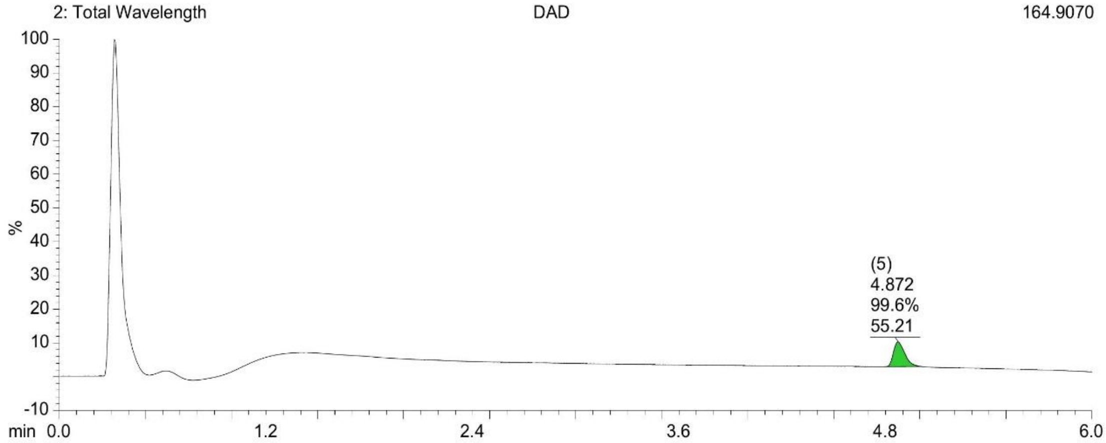

Printed: 1/8/2024 3:18 PM

Analytical Studio Reviewer Report

Sample Name: 414998:01:01 **Acquired:** 5/31/2023 12:49 AM **Instrument:** Agilent TOF **Submitter:**
Location: 1,1:K,1 **Filename:** 1046305730-414998-01-01.d **User:** **Job Code:**

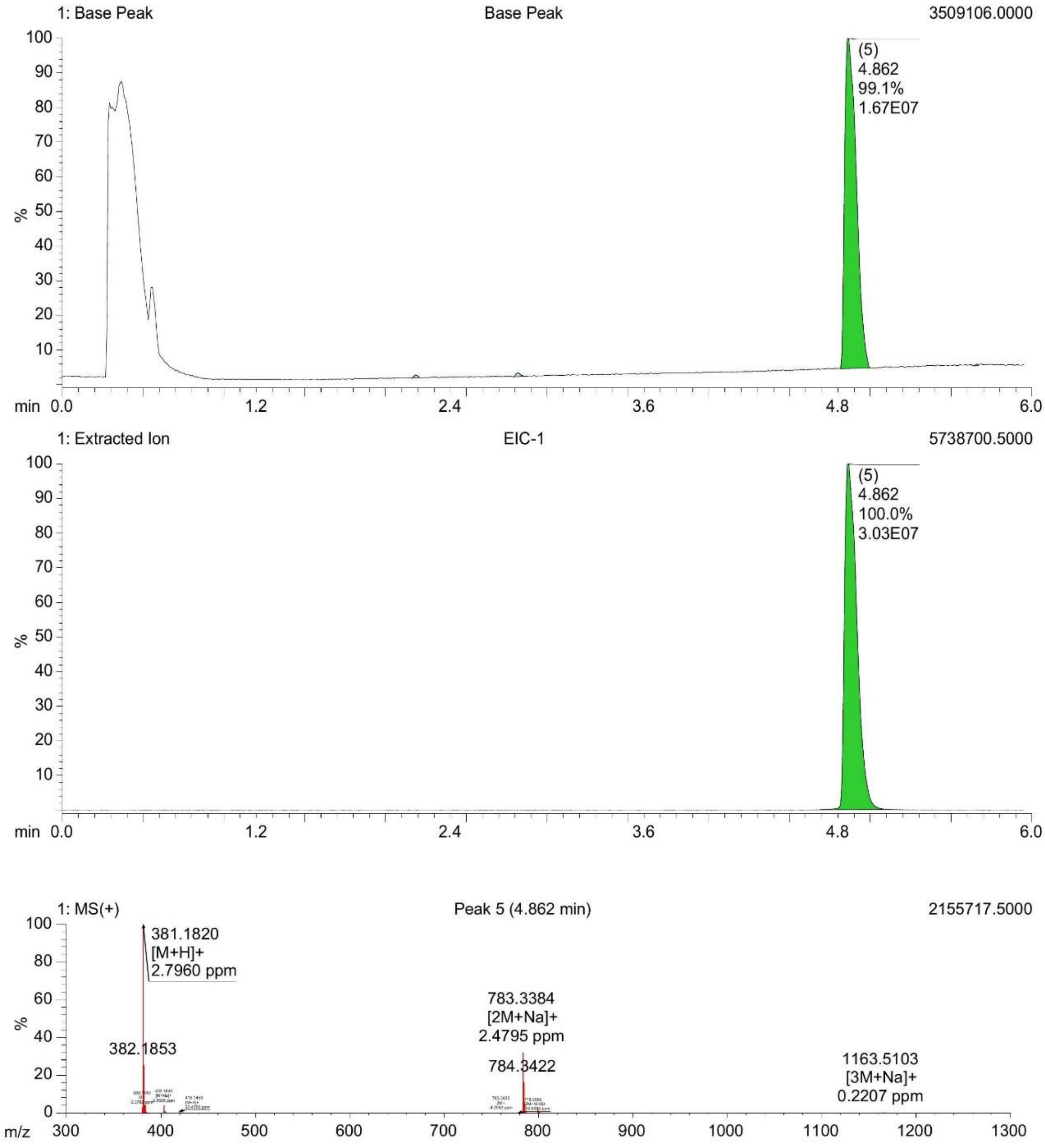

BPM	Error PPM	Error mDa	Target
381.1819	0.2207	0.2568	380.1…

HPLC traces (**23**)

Analytical Studio Reviewer Report

Sample Name: 418045:01:03 **Acquired:** 7/21/2022 1:51 PM **Instrument:** Agilent TOF **Submitter:**
Location: 1,9:O,18 **Filename:** 1046184400-418045-01-03.d **User:** **Job Code:**

				Area %						Area Abs					
Peak #	Time	Target ...	Found	TIC(+)	TIC(+)	TIC(+)	UV254	DAD	Base ...	TIC(+)	TIC(+)	TIC(+)	UV254	DAD	Base Peak
1	1.646	396.1620	NA	0.0	0.0	0.8	0.0	0.0	0.0	0	0	35580.14	0	0	0
2	1.883	396.1620	NA	0.0	0.0	2.6	0.0	0.6	0.0	0	0	114804.85	0	0.09	0
3	2.957	396.1620	Yes	0.0	0.0	96.3	100.0	99.4	100.0	0	0	########	20.94	13.16	########
4	3.612	396.1620	NA	0.0	0.0	0.4	0.0	0.0	0.0	0	0	17693.25	0	0	0

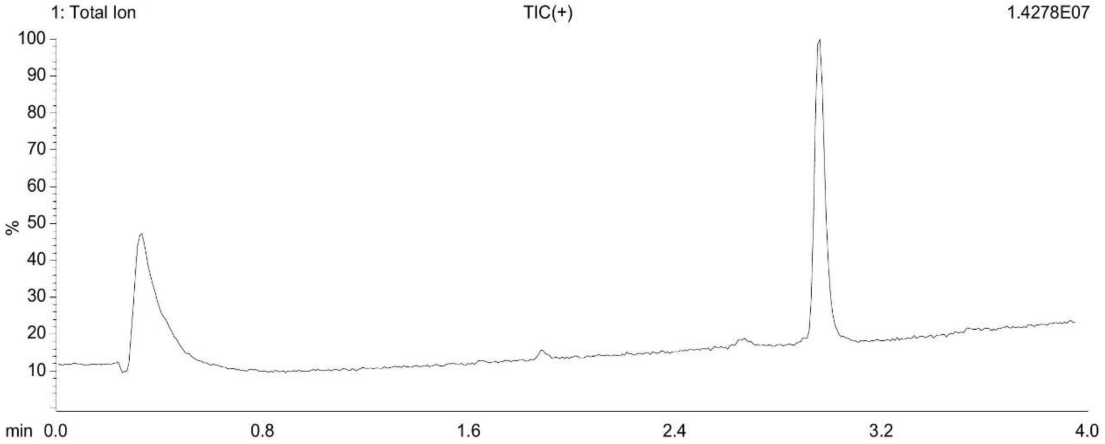

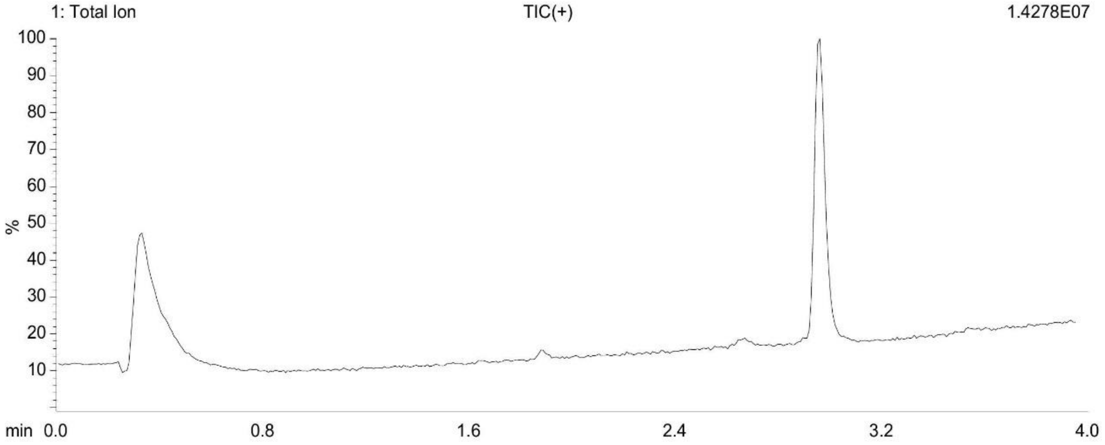

Analytical Studio Reviewer Report

Sample Name: 418045:01:03 **Acquired:** 7/21/2022 1:51 PM **Instrument:** Agilent TOF **Submitter:**
Location: 1,9:O,18 **Filename:** 1046184400-418045-01-03.d **User:** **Job Code:**

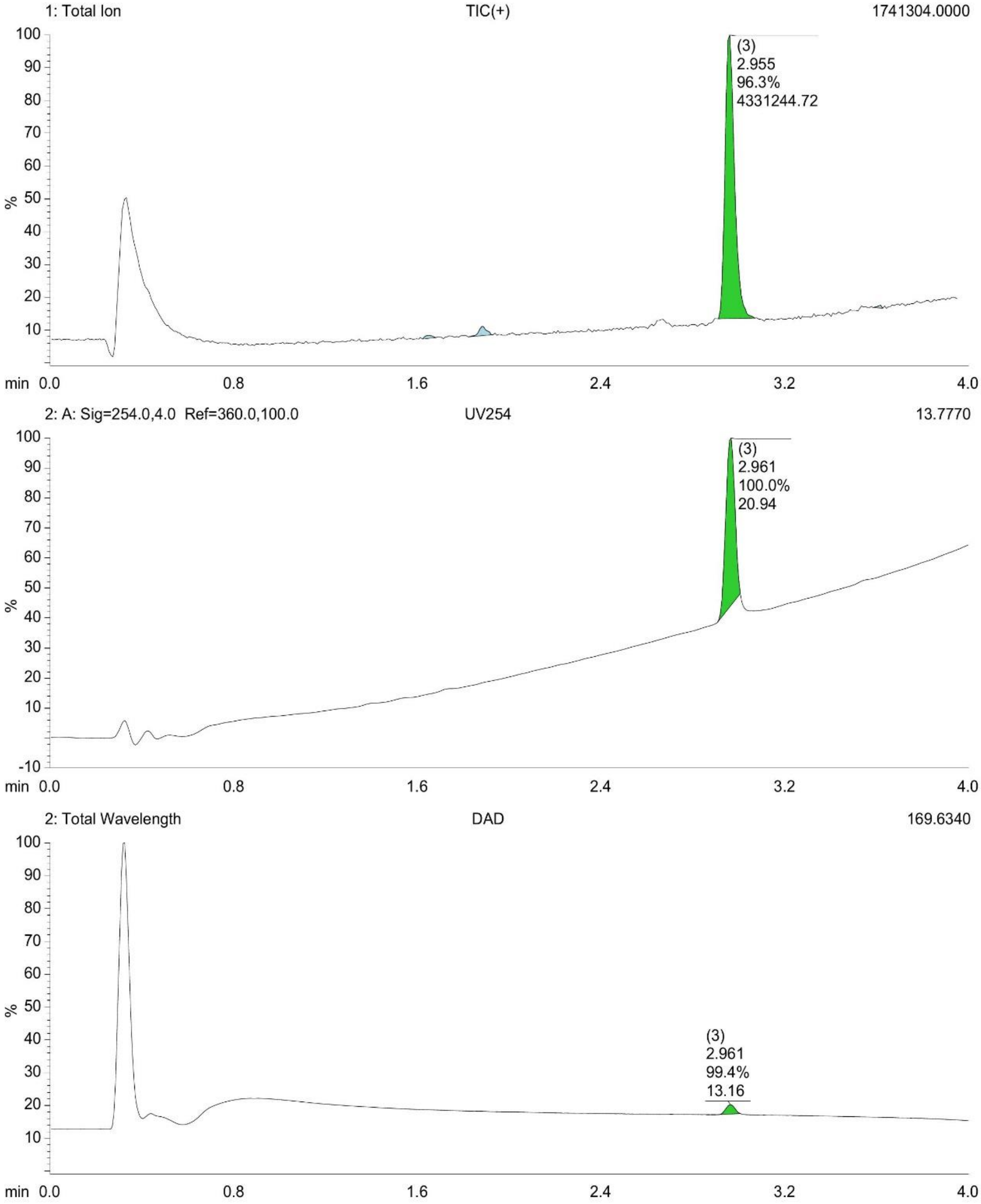

 Printed: 1/8/2024 3:19 PM

Analytical Studio Reviewer Report

Sample Name: 418045:01:03 **Acquired:** 7/21/2022 1:51 PM **Instrument:** Agilent TOF **Submitter:**
Location: 1,9:O,18 **Filename:** 1046184400-418045-01-03.d **User:** **Job Code:**

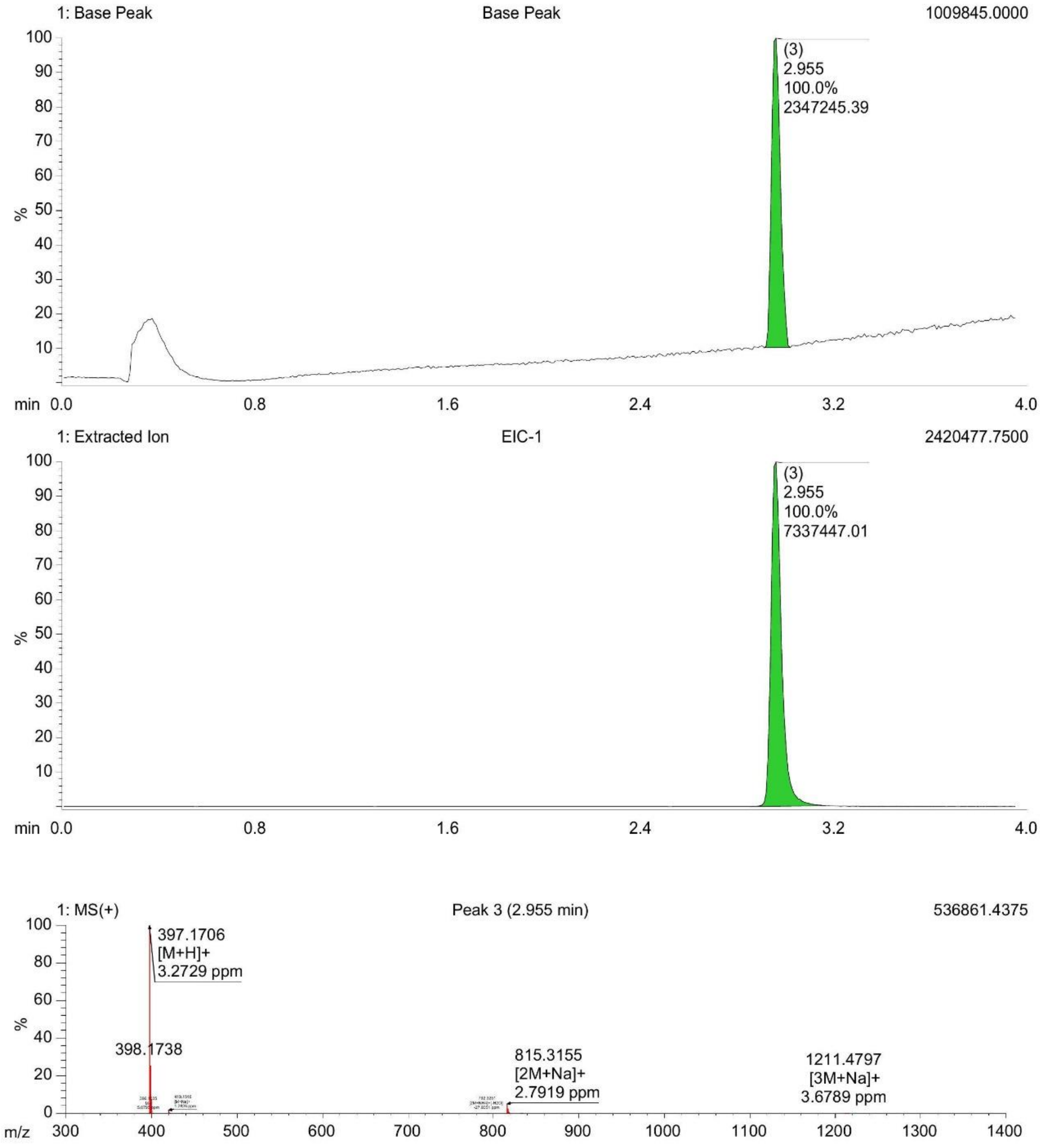

BPM	Error PPM	Error mDa	Target
397.1706	3.6789	4.4569	396.1…

HPLC traces (**24**)

Analytical Studio Reviewer Report

Sample Name: 414680:01:03 **Acquired:** 7/22/2022 7:14 AM **Instrument:** Agilent TOF **Submitter:**
Location: 1,10:G,2 **Filename:** 1046307610-414680-01-03.d **User:** **Job Code:**

				Area %						Area Abs					
Peak #	Time	Target ...	Found	TIC(+)	TIC(+)	TIC(+)	DAD	UV254	Base ...	TIC(+)	TIC(+)	TIC(+)	DAD	UV254	Base Peak
1	1.630	452.1460	NA	0.0	0.0	14.5	6.0	10.2	27.5	0	0	320977.43	0.85	1.23	56582.06
2	3.327	452.1460	Yes	0.0	0.0	85.5	94.0	89.8	72.5	0	0	########	13.34	10.81	148807.49

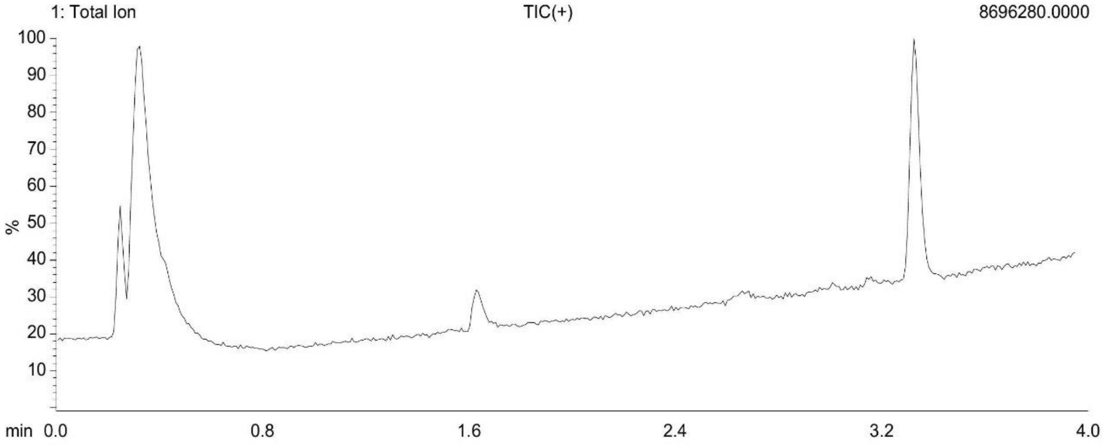

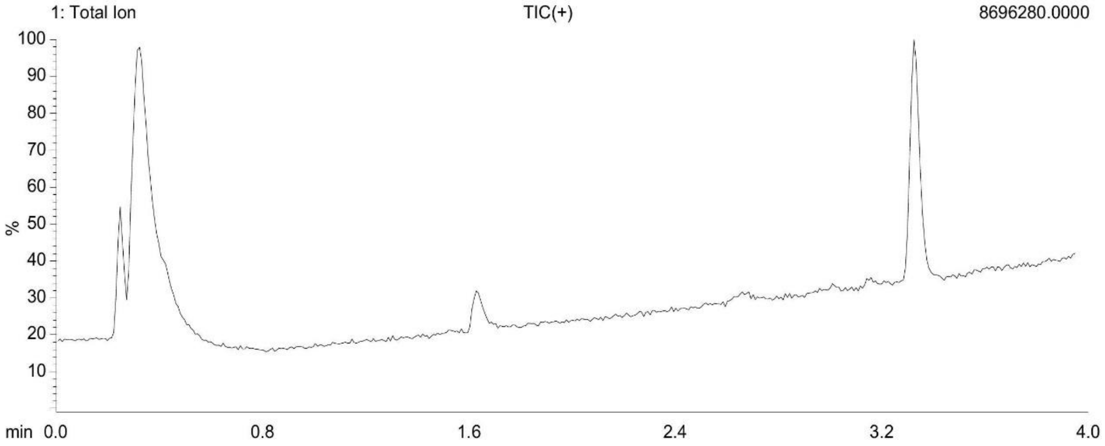

Analytical Studio Reviewer Report

Sample Name: 414680:01:03
Location: 1,10:G,2
Acquired: 7/22/2022 7:14 AM
Filename: 1046307610-414680-01-03.d
Instrument: Agilent TOF
User:
Submitter:
Job Code:

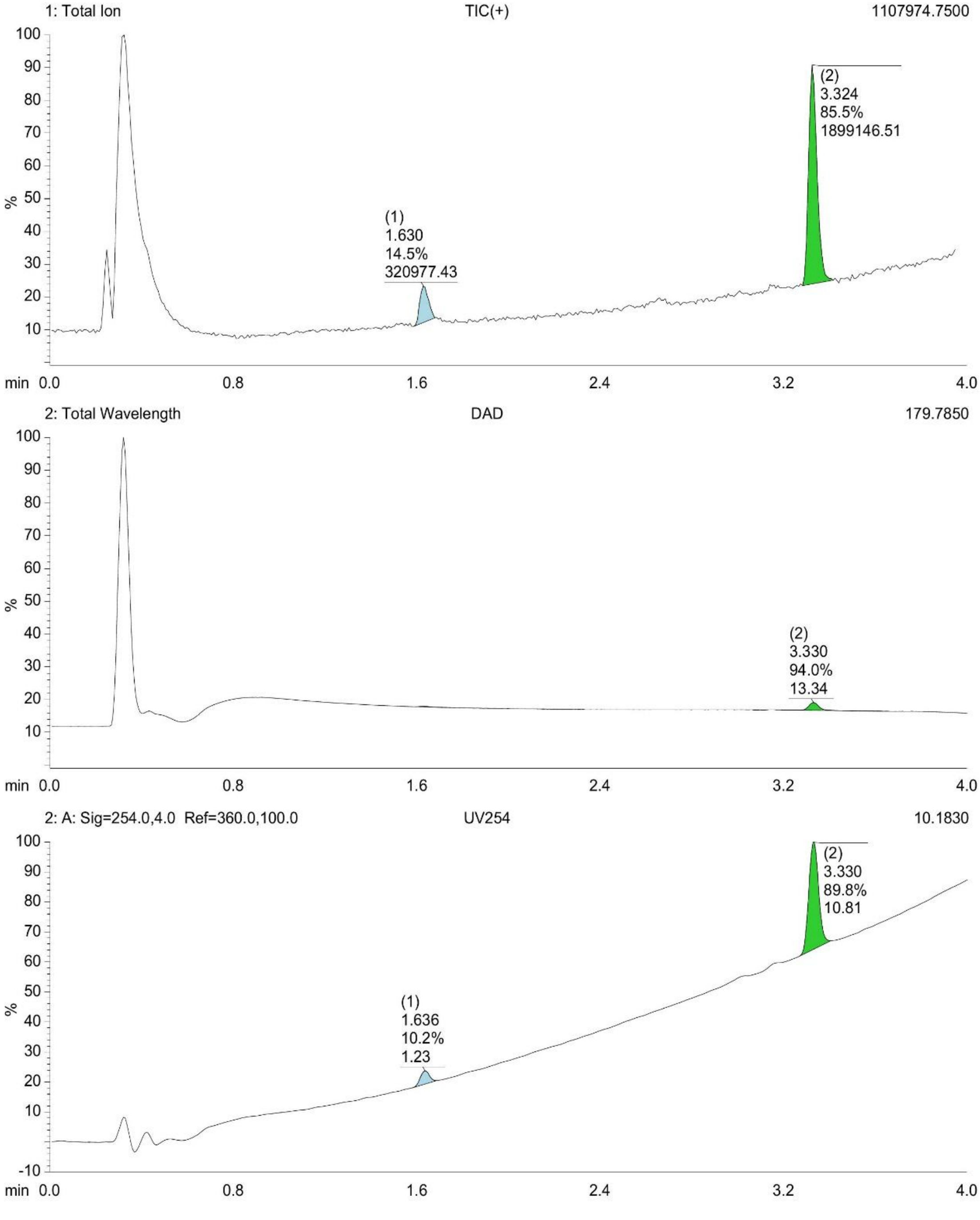

Page 2 of 4

Printed: 1/8/2024 3:18 PM

Analytical Studio Reviewer Report

Sample Name: 414680:01:03 **Acquired:** 7/22/2022 7:14 AM **Instrument:** Agilent TOF **Submitter:**
Location: 1,10:G,2 **Filename:** 1046307610-414680-01-03.d **User:** **Job Code:**

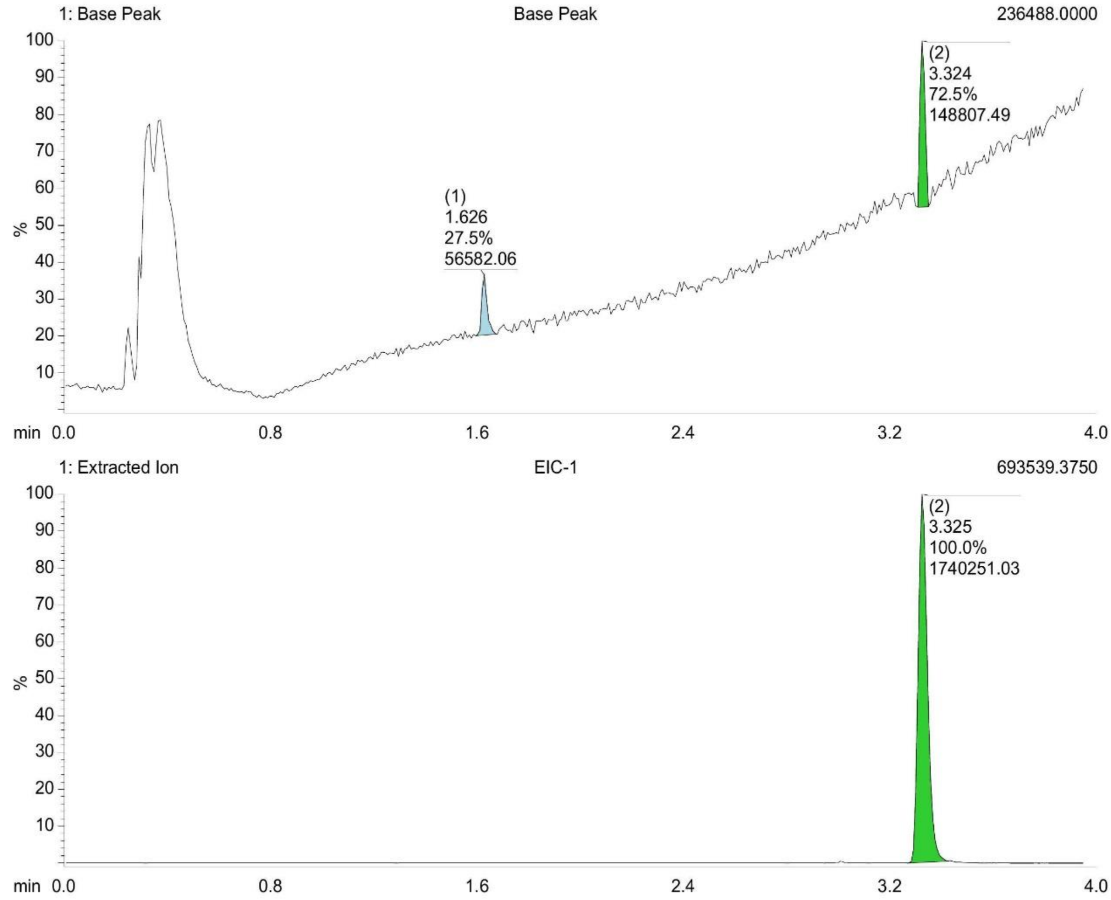

Analytical Studio Reviewer Report

Sample Name: 414680:01:03 **Acquired:** 7/22/2022 7:14 AM **Instrument:** Agilent TOF **Submitter:**
Location: 1,10:G,2 **Filename:** 1046307610-414680-01-03.d **User:** **Job Code:**

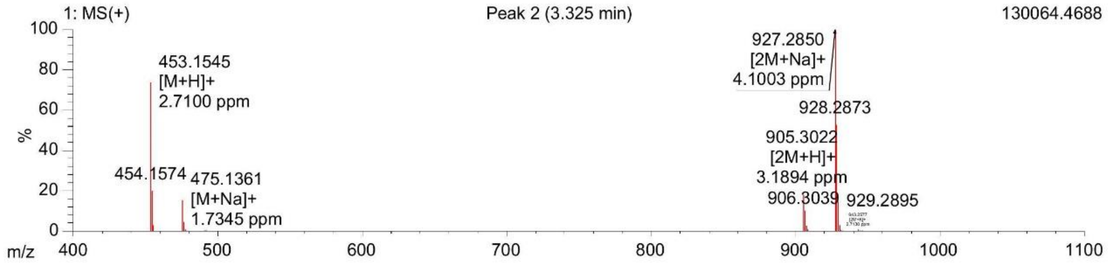

BPM	Error PPM	Error mDa	Target
927.2850	2.7130	2.5590	453.1…

HPLC traces (**25**)

Analytical Studio Reviewer Report

Sample Name: 417572:01:03
Location: 1,10:M,22
Acquired: 7/23/2022 1:45 AM
Filename: 1046185385-417572-01-03.d
Instrument: Agilent TOF
User:
Submitter:
Job Code:

				Area %						Area Abs					
Peak #	Time	Target ...	Found	TIC(+)	TIC(+)	TIC(+)	UV254	DAD	Base ...	TIC(+)	TIC(+)	TIC(+)	UV254	DAD	Base Peak
1	1.331	535.1544	NA	0.0	0.0	1.0	0.0	0.0	0.0	0	0	58089.94	0	0	0
2	2.498	535.1544	NA	0.0	0.0	0.0	0.0	0.0	0.0	0	0	0	0	0.21	0
3	2.730	535.1544	Yes	0.0	0.0	98.0	100.0	100.0	100.0	0	0	########	993.35	510.91	415106.93
4	3.551	535.1544	NA	0.0	0.0	0.7	0.0	0.0	0.0	0	0	42932.48	0	0	0
5	3.702	535.1544	NA	0.0	0.0	0.3	0.0	0.0	0.0	0	0	17788.68	0	0	0

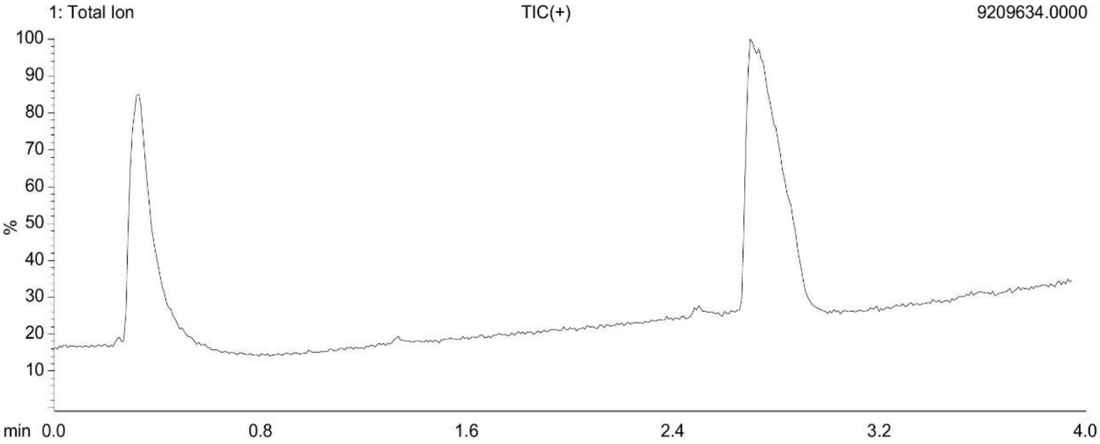

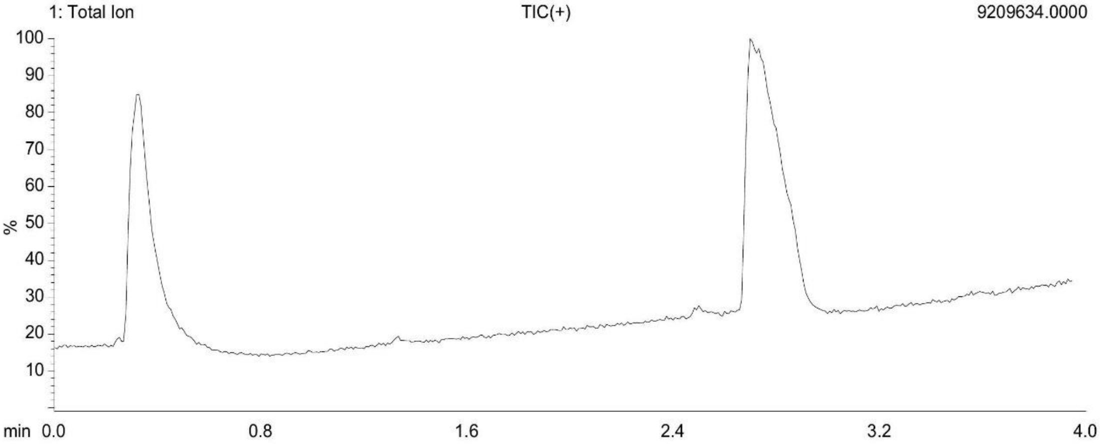

Analytical Studio Reviewer Report

Sample Name: 417572:01:03 **Acquired:** 7/23/2022 1:45 AM **Instrument:** Agilent TOF **Submitter:**
Location: 1,10:M,22 **Filename:** 1046185385-417572-01-03.d **User:** **Job Code:**

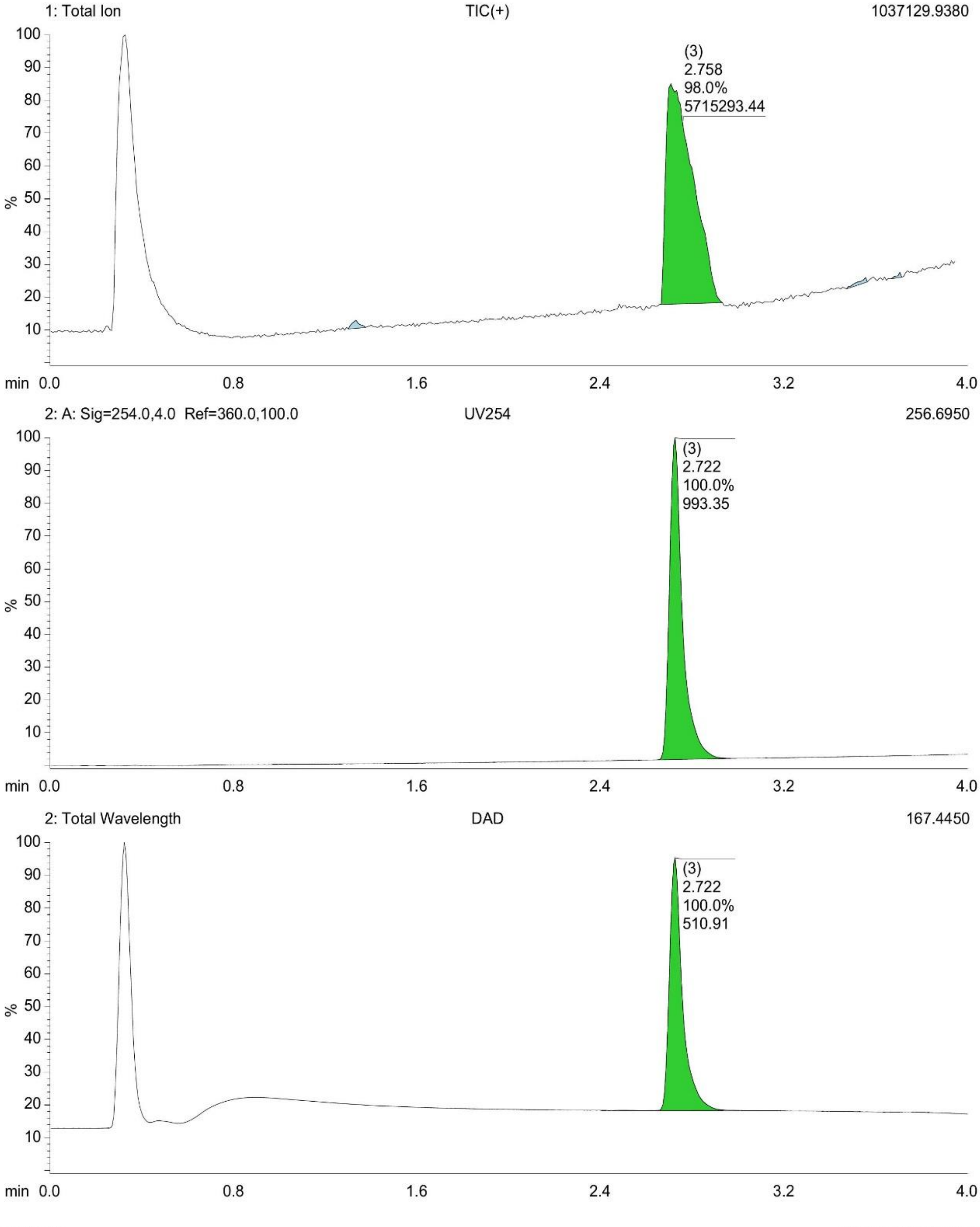

 Printed: 1/8/2024 3:19 PM

Analytical Studio Reviewer Report

Sample Name: 417572:01:03 **Acquired:** 7/23/2022 1:45 AM **Instrument:** Agilent TOF **Submitter:**
Location: 1,10:M,22 **Filename:** 1046185385-417572-01-03.d **User:** **Job Code:**

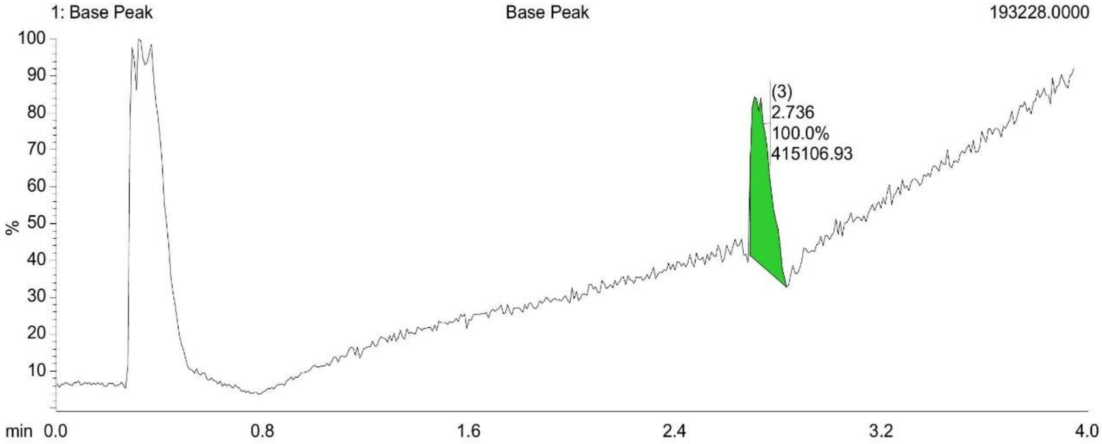

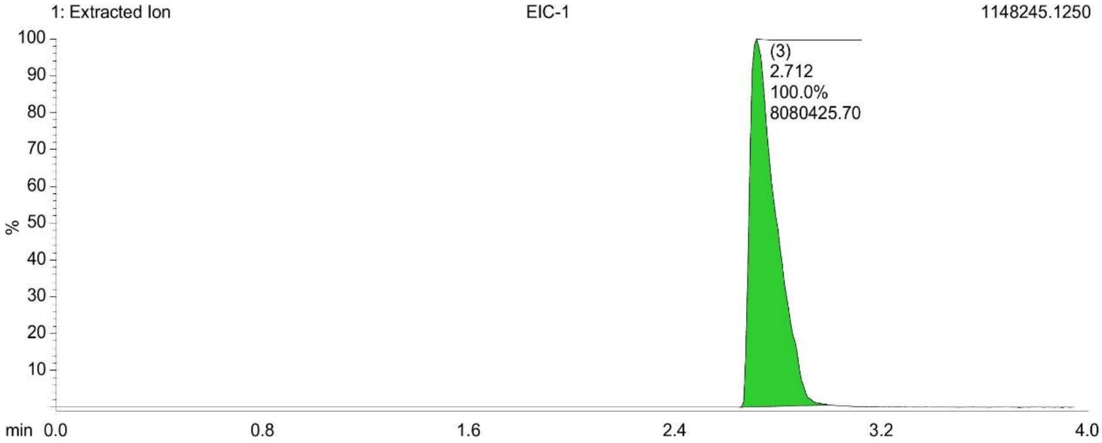

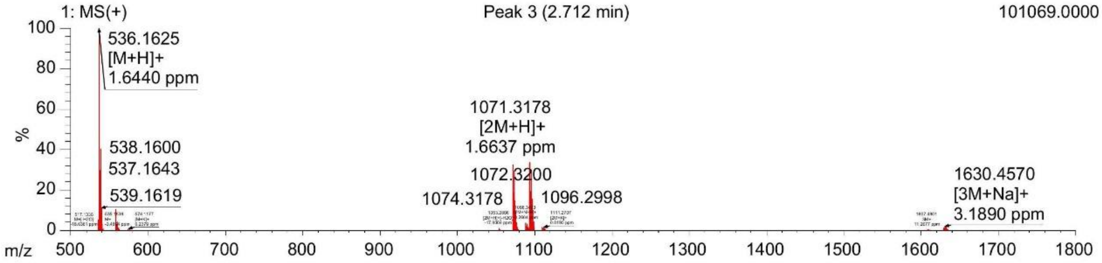

BPM	Error PPM	Error mDa	Target
536.1625	3.1890	5.1995	517.1…

HPLC traces (**26**)

Analytical Studio Reviewer Report

Sample Name: 417403:01:01 **Acquired:** 5/31/2023 12:30 AM **Instrument:** Agilent TOF **Submitter:**
Location: 1,1:G,3 **Filename:** 1046303047-417403-01-01.d **User:** **Job Code:**

				Area %						Area Abs					
Peak #	Time	Target ...	Found	TIC(+)	TIC(+)	TIC(+)	UV254	DAD	Base ...	TIC(+)	TIC(+)	TIC(+)	UV254	DAD	Base Peak
1	2.913	443.2321	NA	0.0	0.0	0.2	0.0	0.0	0.0	0	0	2.47E05	0	0	0
2	3.401	443.2321	NA	0.0	0.0	0.7	0.1	0.0	0.8	0	0	1.01E06	0.24	0.26	5.95E05
3	4.508	443.2321	Yes	0.0	0.0	0.0	99.9	100.0	99.2	0	0	0	414.87	524.97	7.29E07
4	4.555	443.2321	NA	0.0	0.0	99.1	0.0	0.0	0.0	0	0	1.48E08	0	0	0
5	5.029	443.2321	Yes	0.0	0.0	0.0	0.0	0.0	0.0	0	0	0	0	0	0
6	5.061	443.2321	Yes	0.0	0.0	0.0	0.0	0.0	0.0	0	0	0	0	0	0
7	5.237	443.2321	NA	0.0	0.0	0.0	0.0	0.0	0.0	0	0	0	0	0	1.24E04
8	5.410	443.2321	Yes	0.0	0.0	0.0	0.0	0.0	0.0	0	0	0	0	0	0
9	5.758	443.2321	Yes	0.0	0.0	0.0	0.0	0.0	0.0	0	0	0	0	0	0
10	5.895	443.2321	NA	0.0	0.0	0.1	0.0	0.0	0.0	0	0	1.03E05	0	0	0

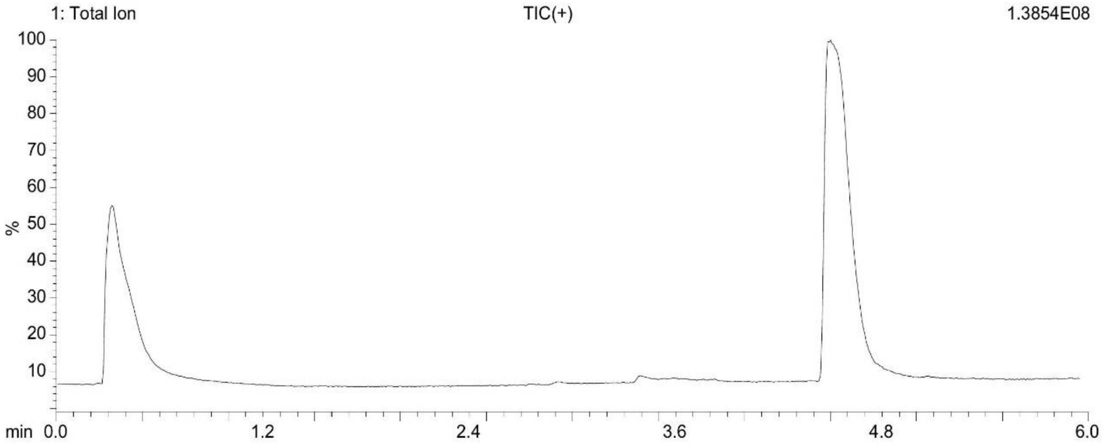

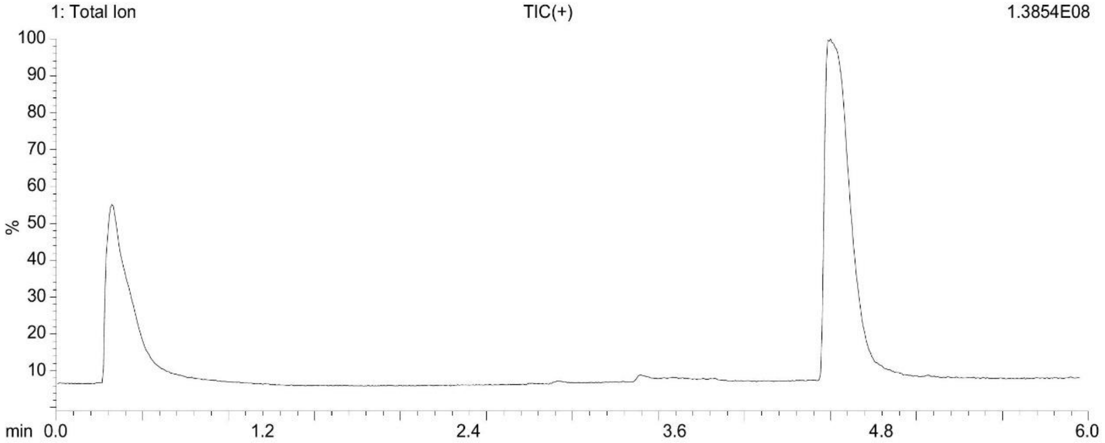

Page 1 of 6 Printed: 1/8/2024 3:19 PM

Analytical Studio Reviewer Report

Sample Name: 417403:01:01 **Acquired:** 5/31/2023 12:30 AM **Instrument:** Agilent TOF **Submitter:**
Location: 1,1:G,3 **Filename:** 1046303047-417403-01-01.d **User:** **Job Code:**

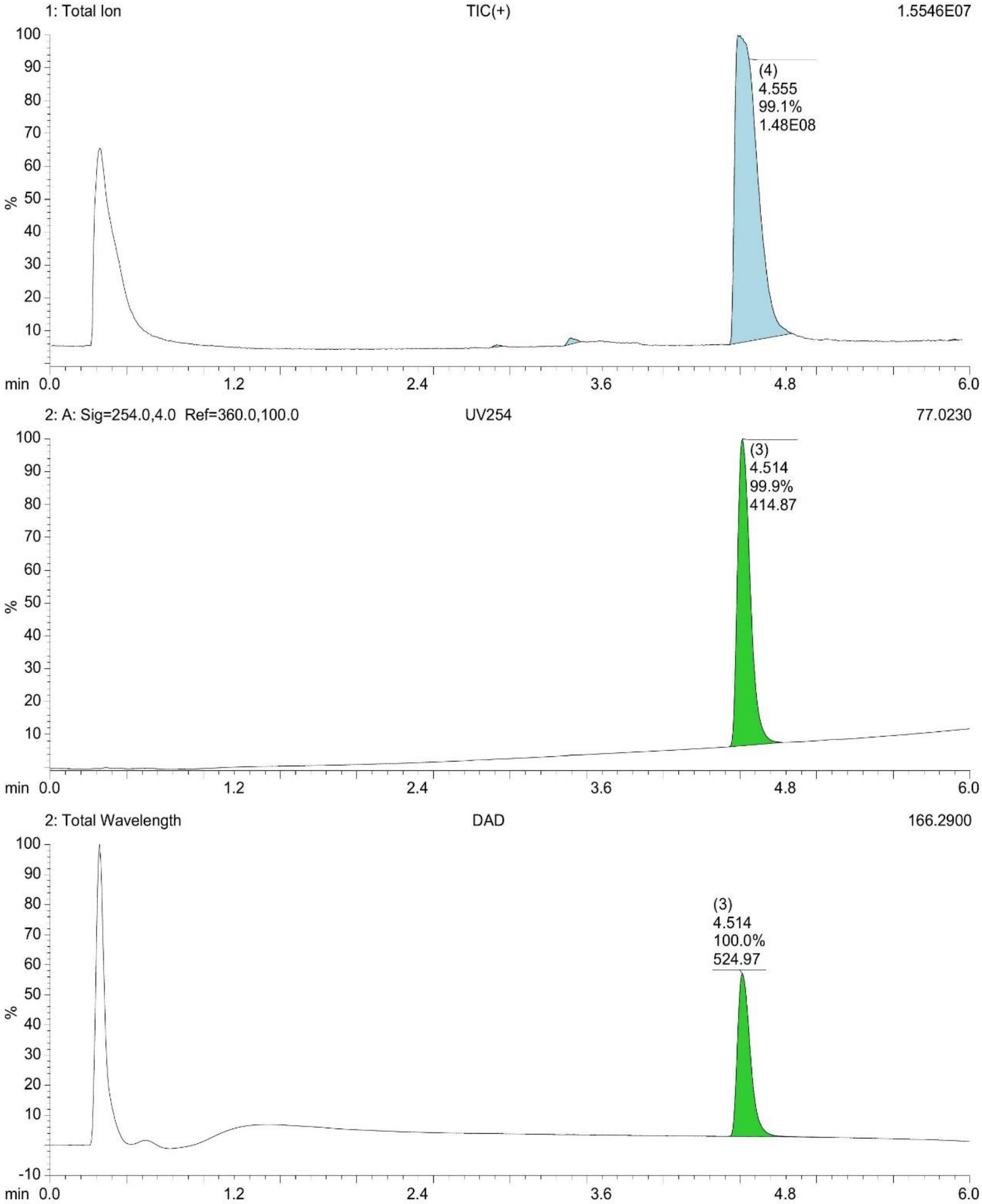

 Printed: 1/8/2024 3:19 PM

Analytical Studio Reviewer Report

Sample Name: 417403:01:01
Location: 1,1:G,3
Acquired: 5/31/2023 12:30 AM
Filename: 1046303047-417403-01-01.d
Instrument: Agilent TOF
User:
Submitter:
Job Code:

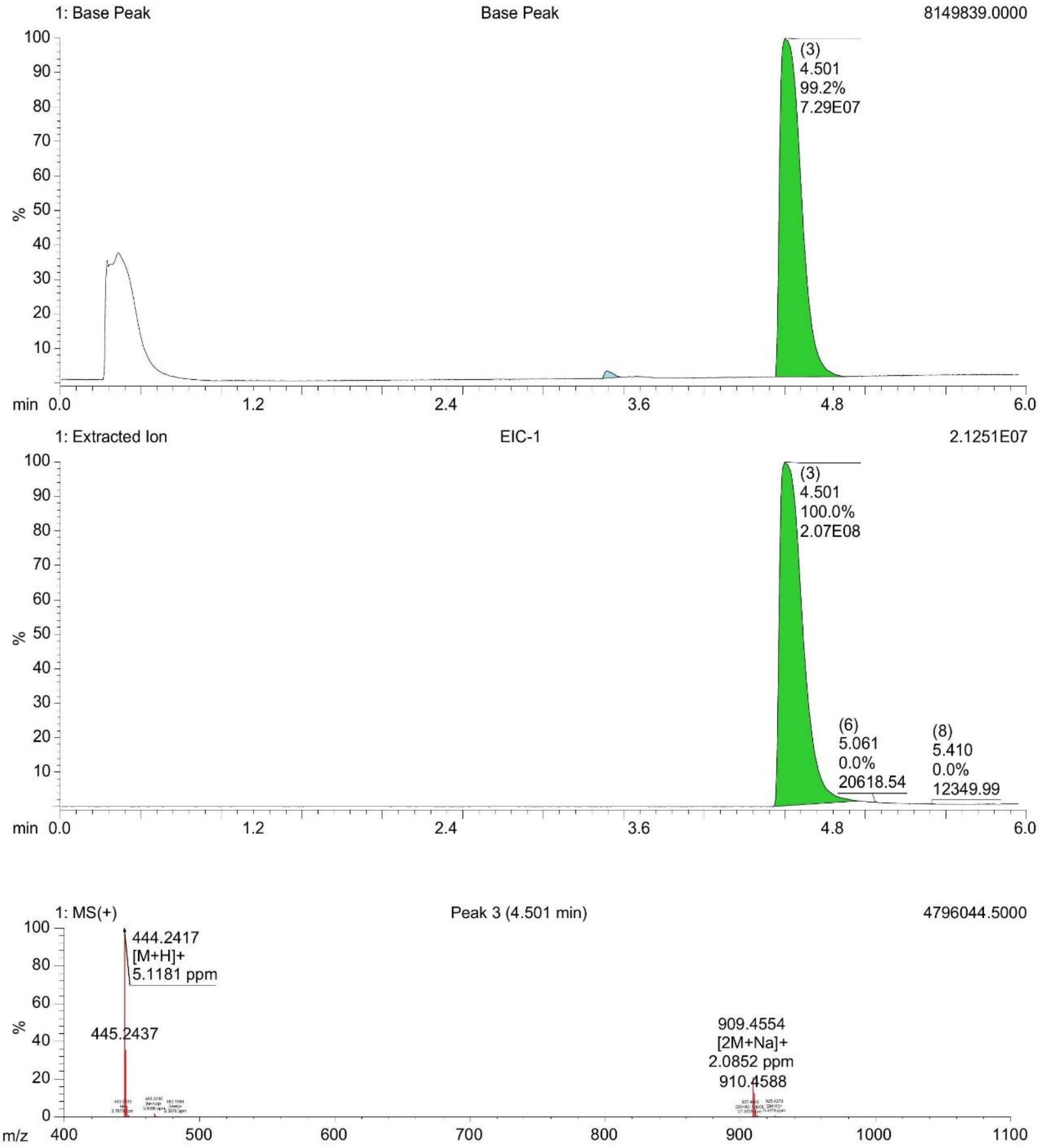

BPM	Error PPM	Error mDa	Target
444.2417	0.4278	0.3959	443.2…

HPLC traces (**27**)

Analytical Studio Reviewer Report

Sample Name: 416553:01:03 **Acquired:** 7/22/2022 6:29 AM **Instrument:** Agilent TOF **Submitter:**
Location: 1,10:F,20 **Filename:** 1046305810-416553-01-03.d **User:** **Job Code:**

				Area %						Area Abs					
Peak #	Time	Target ...	Found	TIC(+)	TIC(+)	TIC(+)	UV254	DAD	Base ...	TIC(+)	TIC(+)	TIC(+)	UV254	DAD	Base Peak
1	3.106	445.0351	Yes	0.0	0.0	99.5	100.0	100.0	0.0	0	0	########	18.51	16.11	0
2	3.667	445.0351	NA	0.0	0.0	0.5	0.0	0.0	0.0	0	0	9194.27	0	0	0

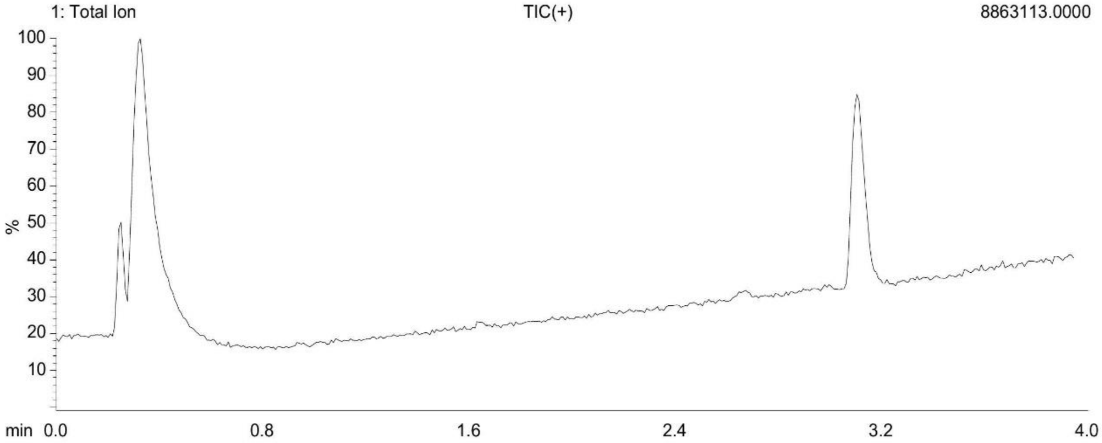

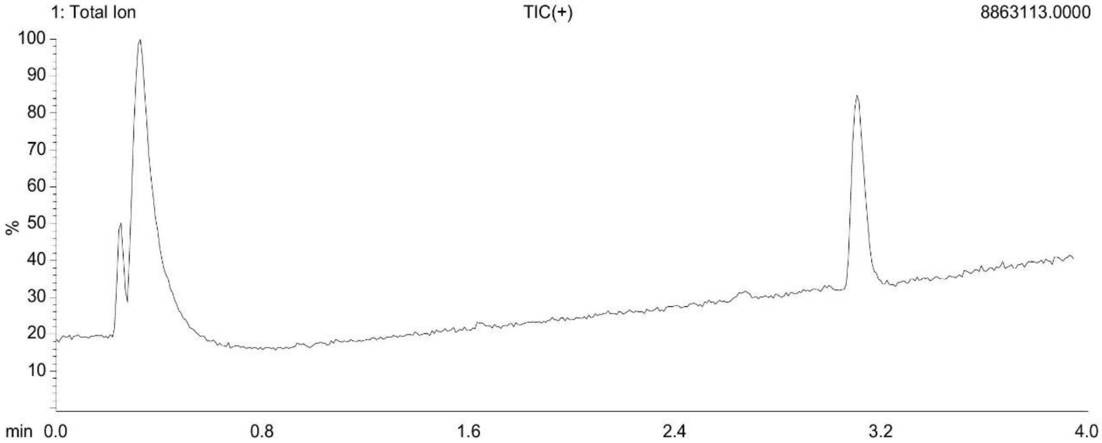

Analytical Studio Reviewer Report

Sample Name: 416553:01:03
Location: 1,10:F,20
Acquired: 7/22/2022 6:29 AM
Filename: 1046305810-416553-01-03.d
Instrument: Agilent TOF
User:
Submitter:
Job Code:

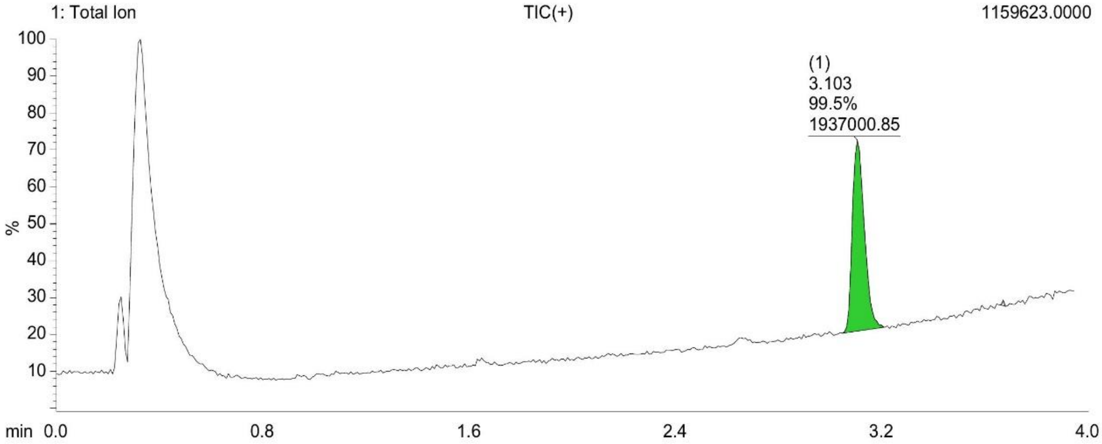

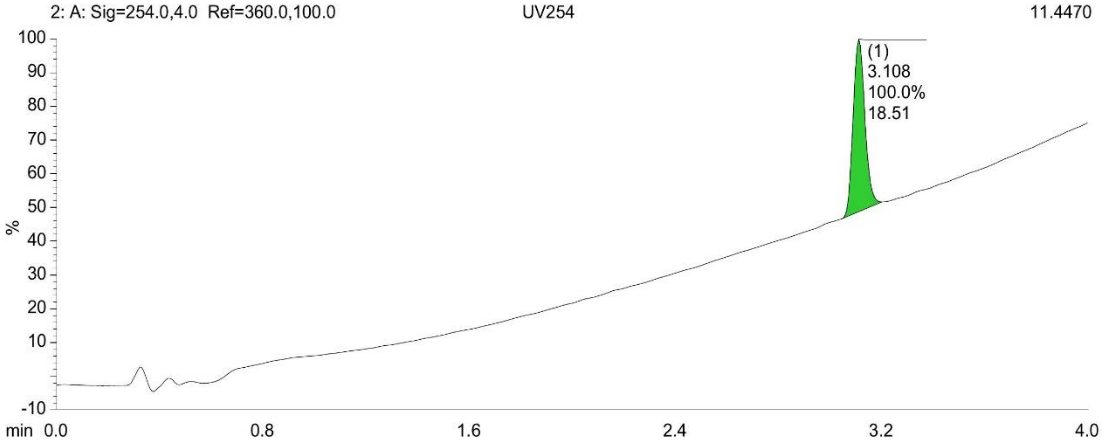

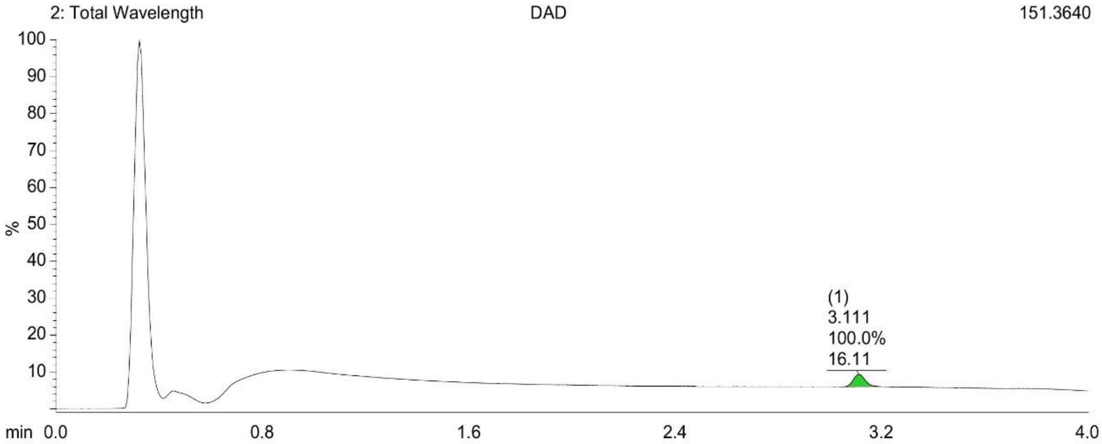

Printed: 1/8/2024 3:19 PM

Analytical Studio Reviewer Report

Sample Name: 416553:01:03 **Acquired:** 7/22/2022 6:29 AM **Instrument:** Agilent TOF **Submitter:**
Location: 1,10:F,20 **Filename:** 1046305810-416553-01-03.d **User:** **Job Code:**

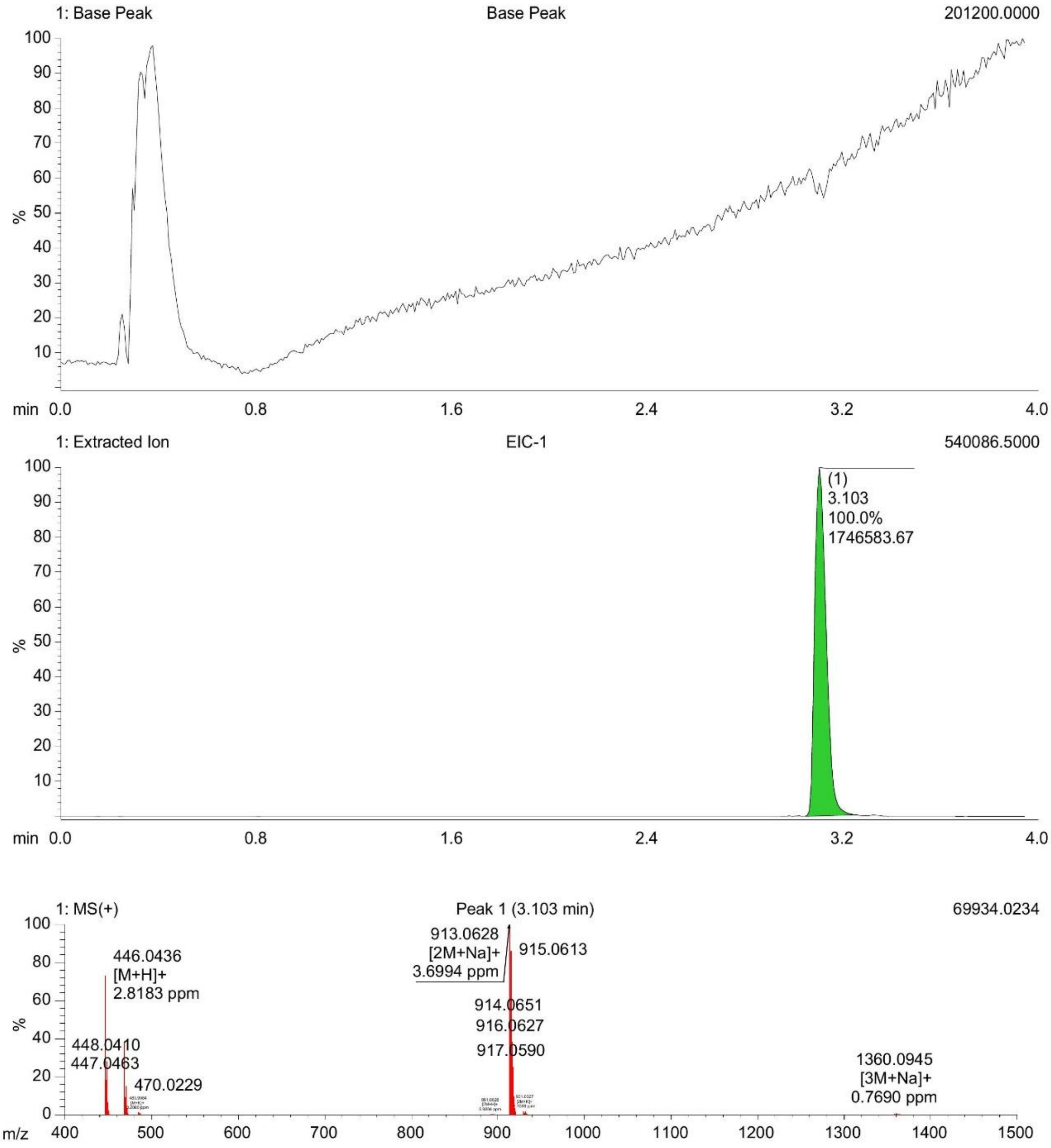

BPM	Error PPM	Error mDa	Target
913.0628	0.7690	1.0460	446.0…

 Printed: 1/8/2024 3:19 PM

HPLC traces (**28**)

Analytical Studio Reviewer Report

Sample Name: 415422:01:03 **Acquired:** 7/21/2022 1:44 PM **Instrument:** Agilent TOF **Submitter:**
Location: 1,9:O,17 **Filename:** 1046307836-415422-01-03.d **User:** **Job Code:**

				Area %						Area Abs					
Peak #	Time	Target ...	Found	TIC(+)	TIC(+)	TIC(+)	DAD	UV254	Base ...	TIC(+)	TIC(+)	TIC(+)	DAD	UV254	Base Peak
1	1.370	423.1794	NA	0.0	0.0	1.5	0.2	1.4	1.5	0	0	5.65E05	2.06	3.38	3.58E05
2	1.719	423.1794	NA	0.0	0.0	2.4	0.2	0.2	2.3	0	0	9.08E05	1.70	0.44	5.72E05
3	1.864	423.1794	Yes	0.0	0.0	95.7	99.6	98.4	96.2	0	0	3.61E07	1042.44	237.86	2.34E07
4	2.353	423.1794	NA	0.0	0.0	0.4	0.1	0.0	0.0	0	0	1.41E05	0.60	0	0

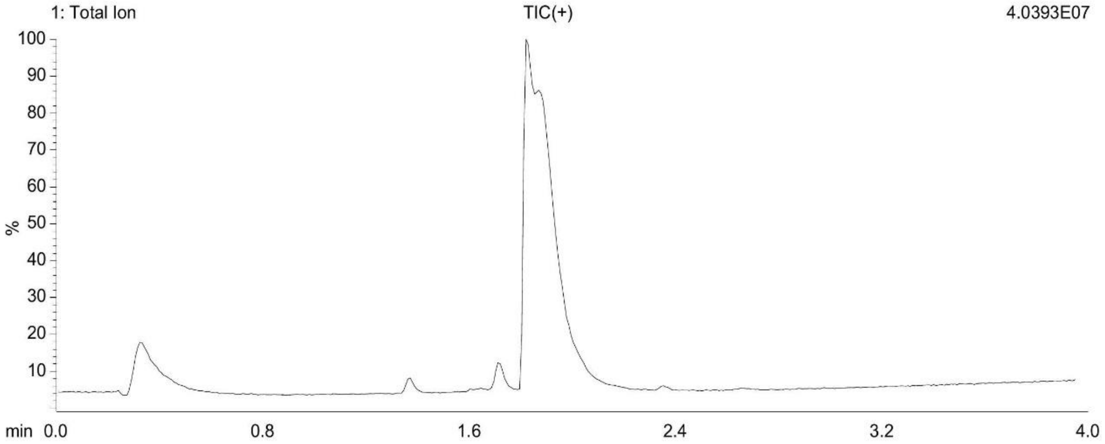

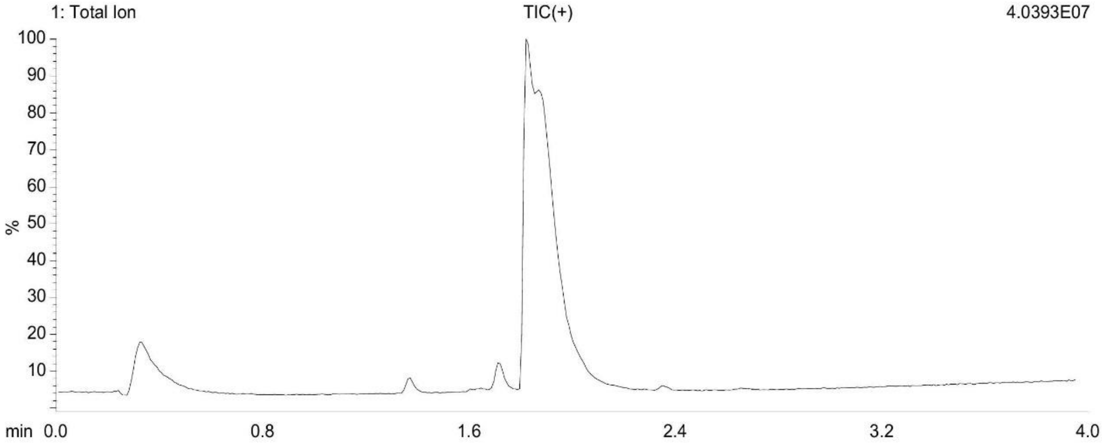

Analytical Studio Reviewer Report

Sample Name: 415422:01:03 **Acquired:** 7/21/2022 1:44 PM **Instrument:** Agilent TOF **Submitter:**
Location: 1,9:O,17 **Filename:** 1046307836-415422-01-03.d **User:** **Job Code:**

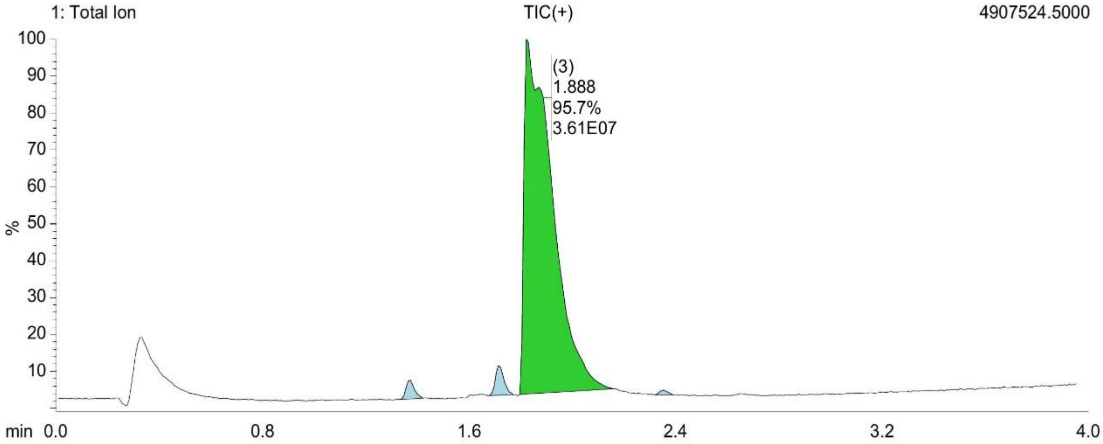

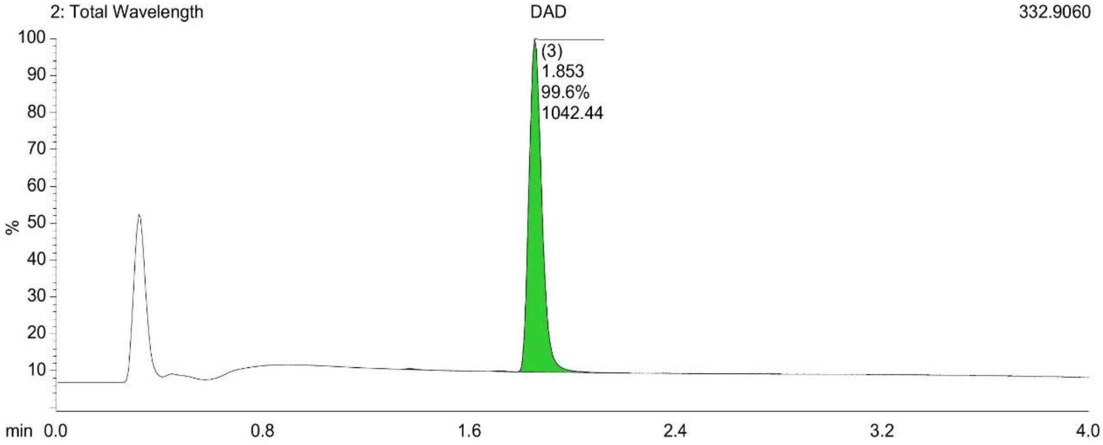

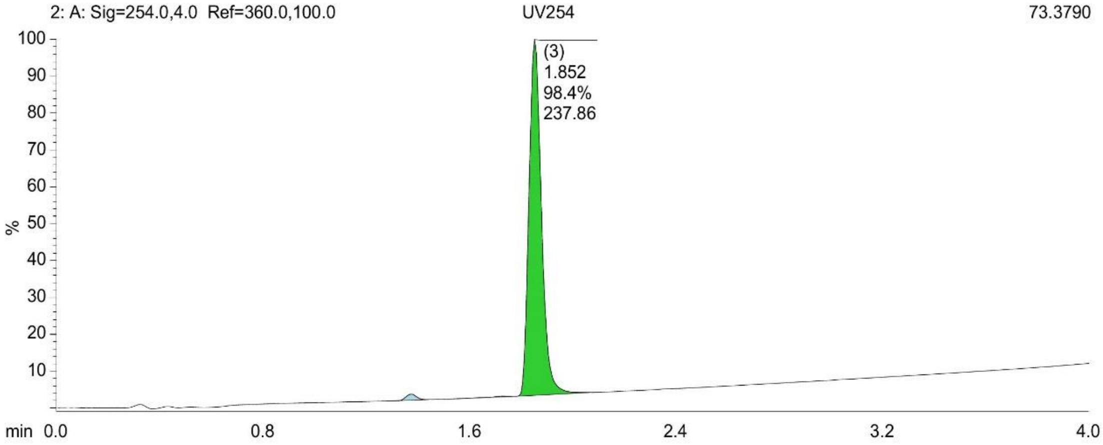

 Printed: 1/8/2024 3:19 PM

Analytical Studio Reviewer Report

Sample Name: 415422:01:03 **Acquired:** 7/21/2022 1:44 PM **Instrument:** Agilent TOF **Submitter:**
Location: 1,9:O,17 **Filename:** 1046307836-415422-01-03.d **User:** **Job Code:**

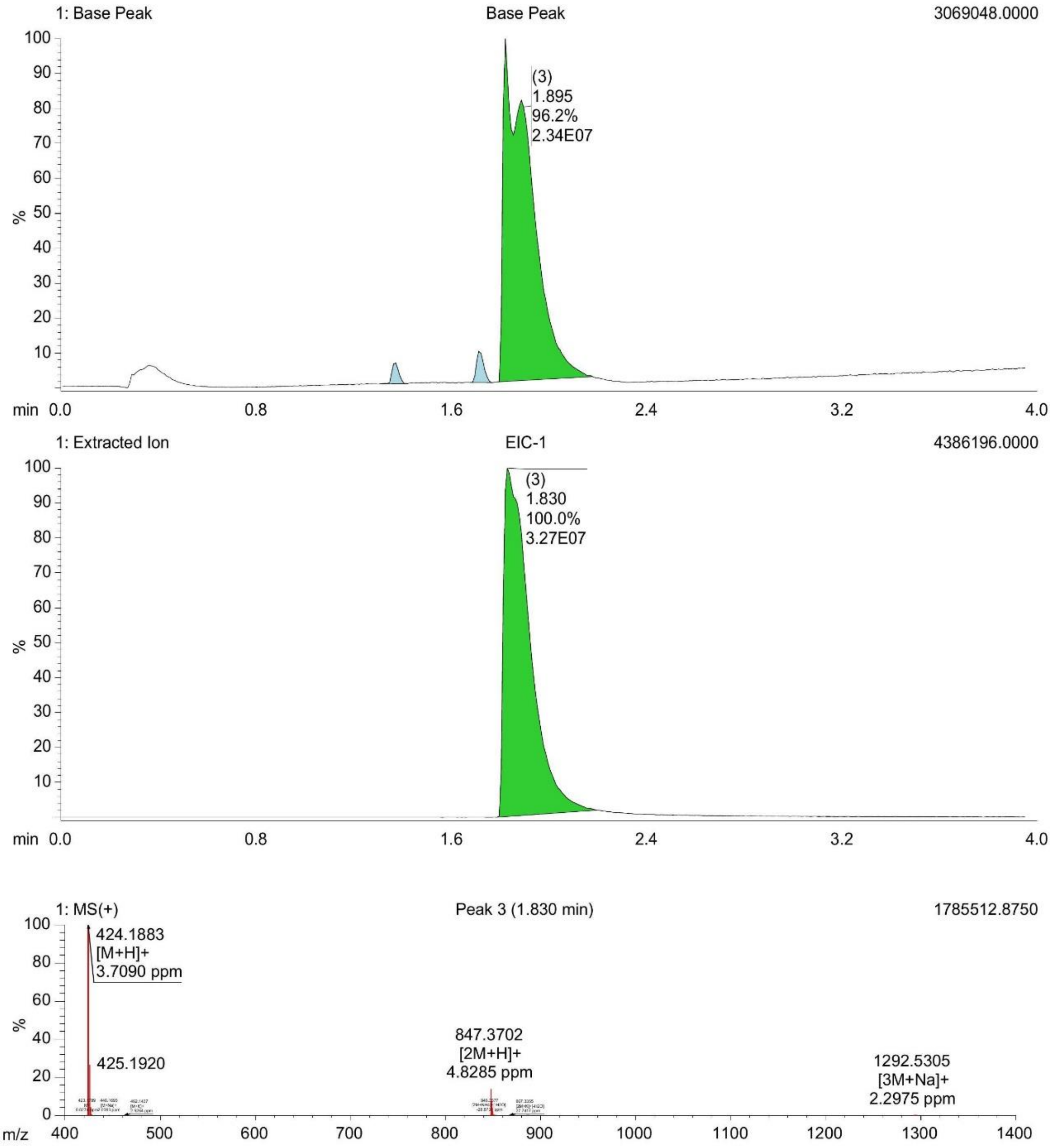

BPM	Error PPM	Error mDa	Target
424.1883	2.2975	2.9695	423.1…

HPLC traces (**29**)

Analytical Studio Reviewer Report

Sample Name: 414904:01:03 **Acquired:** 7/21/2022 9:42 PM **Instrument:** Agilent TOF **Submitter:**
Location: 1,10:C,14 **Filename:** 1046327979-414904-01-03.d **User:** **Job Code:**

				Area %						Area Abs					
Peak #	Time	Target ...	Found	TIC(+)	TIC(+)	TIC(+)	DAD	UV254	Base ...	TIC(+)	TIC(+)	TIC(+)	DAD	UV254	Base Peak
1	1.913	536.0059	Yes	0.0	0.0	98.4	97.4	95.7	100.0	0	0	########	25.51	26.76	767544.25
2	2.028	536.0059	NA	0.0	0.0	0.0	1.7	2.9	0.0	0	0	0	0.45	0.81	0
3	2.127	536.0059	NA	0.0	0.0	0.0	0.9	1.5	0.0	0	0	0	0.23	0.41	0
4	3.702	536.0059	NA	0.0	0.0	0.9	0.0	0.0	0.0	0	0	28636.85	0	0	0
5	3.811	536.0059	NA	0.0	0.0	0.7	0.0	0.0	0.0	0	0	22835.22	0	0	0

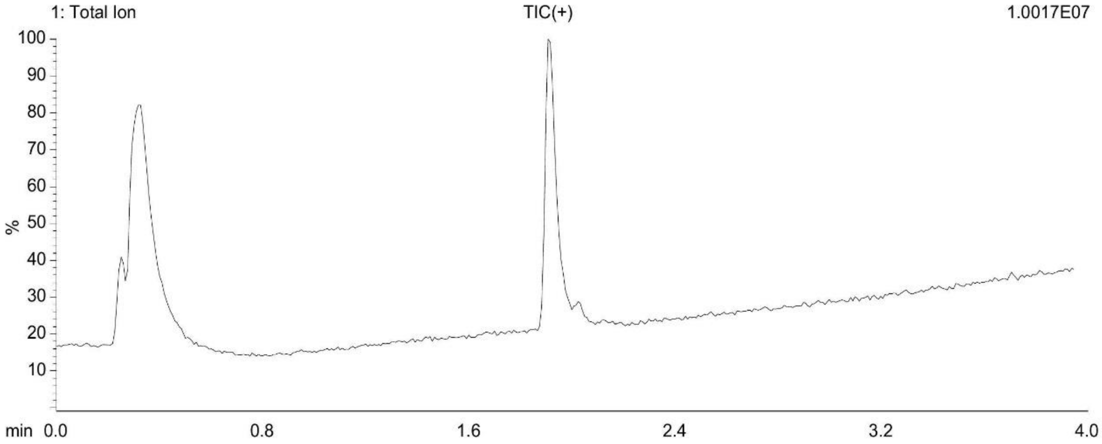

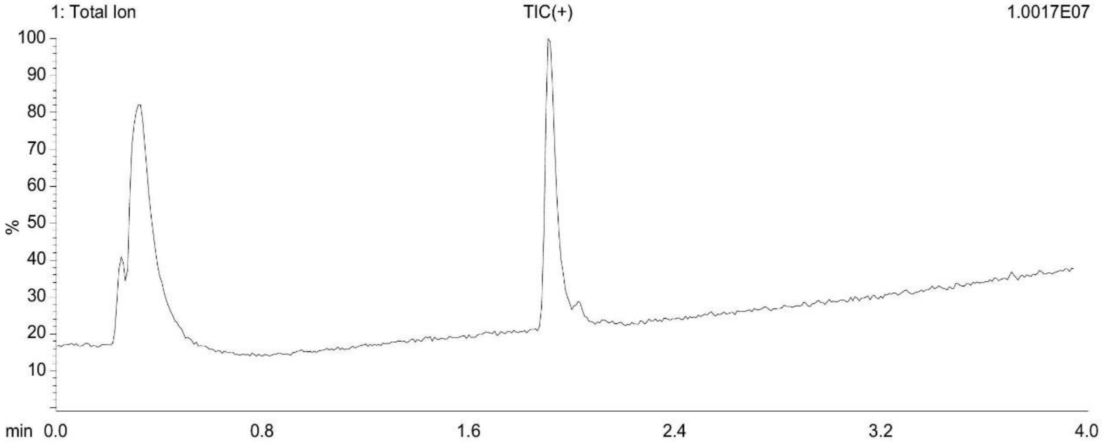

Analytical Studio Reviewer Report

Sample Name: 414904:01:03 **Acquired:** 7/21/2022 9:42 PM **Instrument:** Agilent TOF **Submitter:**
Location: 1,10:C,14 **Filename:** 1046327979-414904-01-03.d **User:** **Job Code:**

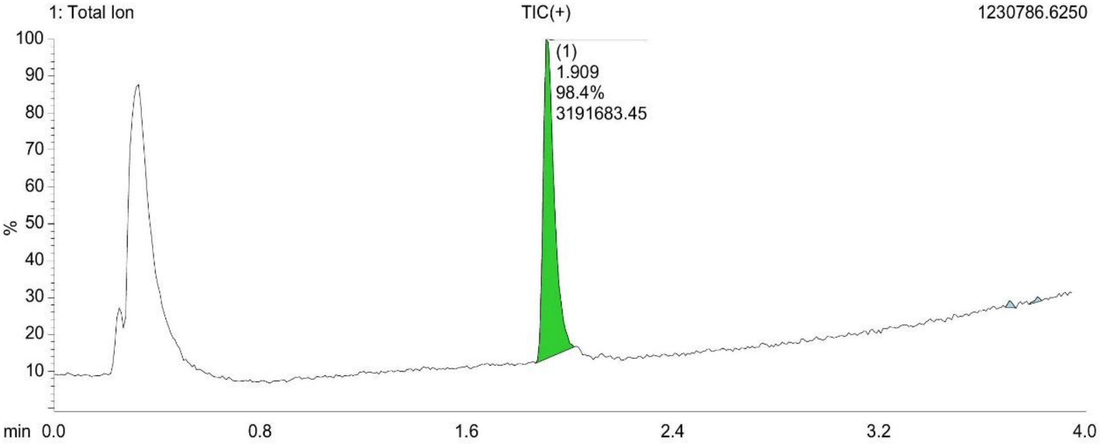

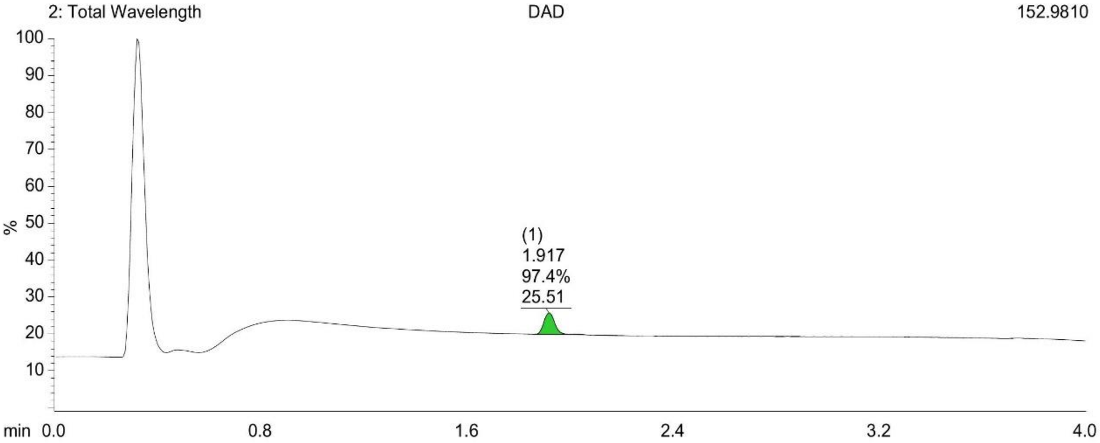

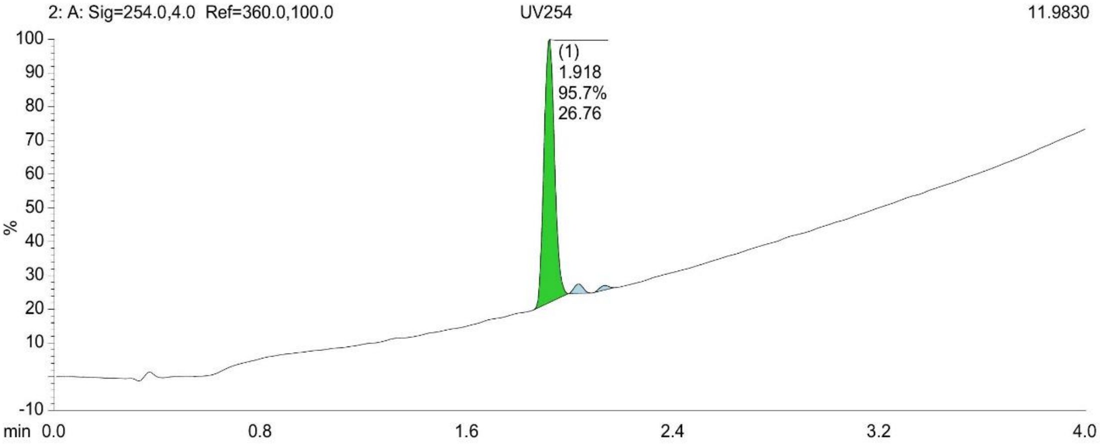

 Printed: 1/8/2024 3:19 PM

Analytical Studio Reviewer Report

Sample Name: 414904:01:03 **Acquired:** 7/21/2022 9:42 PM **Instrument:** Agilent TOF **Submitter:**
Location: 1,10:C,14 **Filename:** 1046327979-414904-01-03.d **User:** **Job Code:**

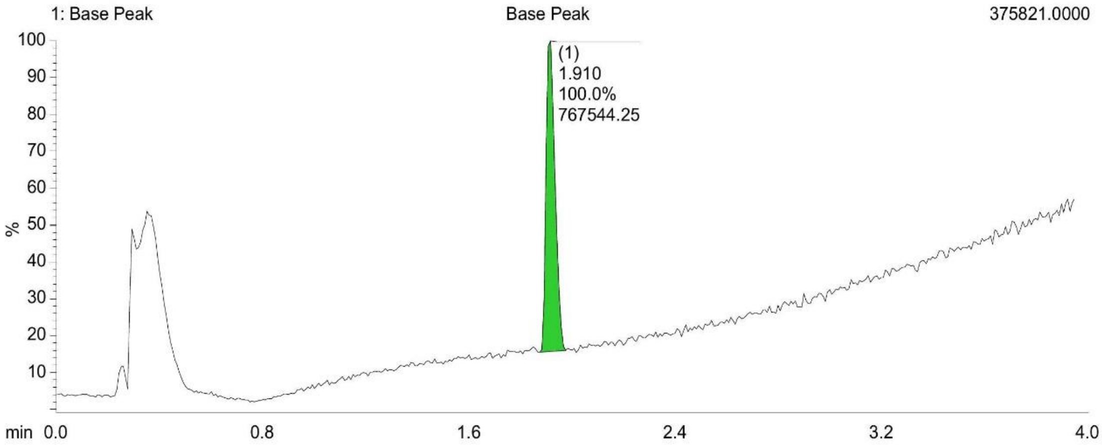

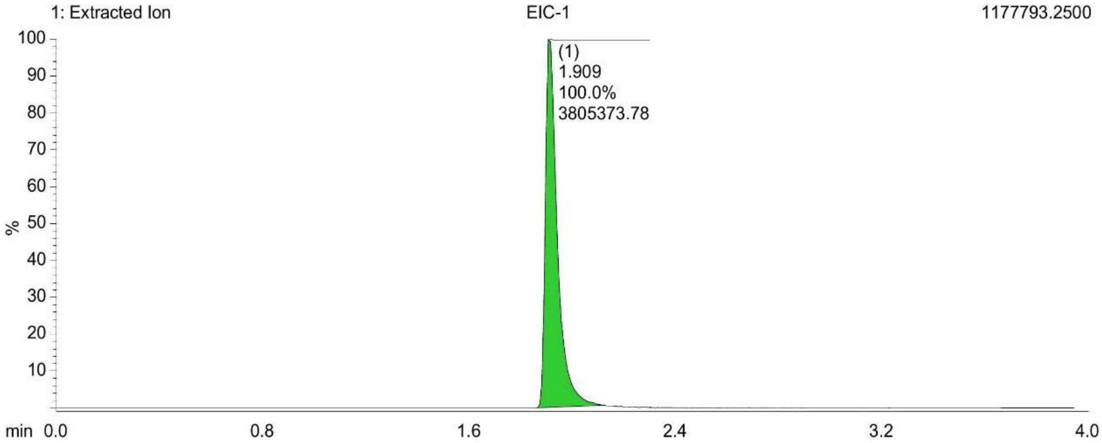

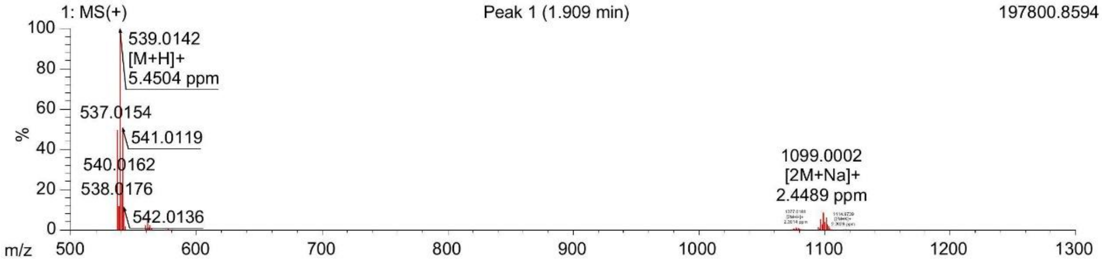

BPM	Error PPM	Error mDa	Target
539.0142	2.2026	2.4558	539.0…

HPLC traces (**30**)

Analytical Studio Reviewer Report

Sample Name: 418828:01:03 **Acquired:** 7/22/2022 9:20 AM **Instrument:** Agilent TOF **Submitter:**
Location: 1,10:G,21 **Filename:** 1046184285-418828-01-03.d **User:** **Job Code:**

				Area %						Area Abs					
Peak #	Time	Target ...	Found	TIC(+)	TIC(+)	TIC(+)	UV254	DAD	Base ...	TIC(+)	TIC(+)	TIC(+)	UV254	DAD	Base Peak
1	2.549	340.1423	NA	0.0	0.0	0.0	0.6	0.4	0.0	0	0	0	0.43	0.16	0
2	2.751	340.1423	NA	0.0	0.0	0.0	0.2	0.0	0.0	0	0	0	0.12	0	0
3	2.899	340.1423	Yes	0.0	0.0	98.9	97.2	97.5	100.0	0	0	########	70.50	36.21	723848.45
4	3.080	340.1423	NA	0.0	0.0	0.0	1.0	1.1	0.0	0	0	0	0.71	0.40	0
5	3.268	340.1423	NA	0.0	0.0	0.8	0.6	0.4	0.0	0	0	44644.42	0.44	0.14	0
6	3.594	340.1423	NA	0.0	0.0	0.3	0.4	0.6	0.0	0	0	15425.32	0.30	0.23	0

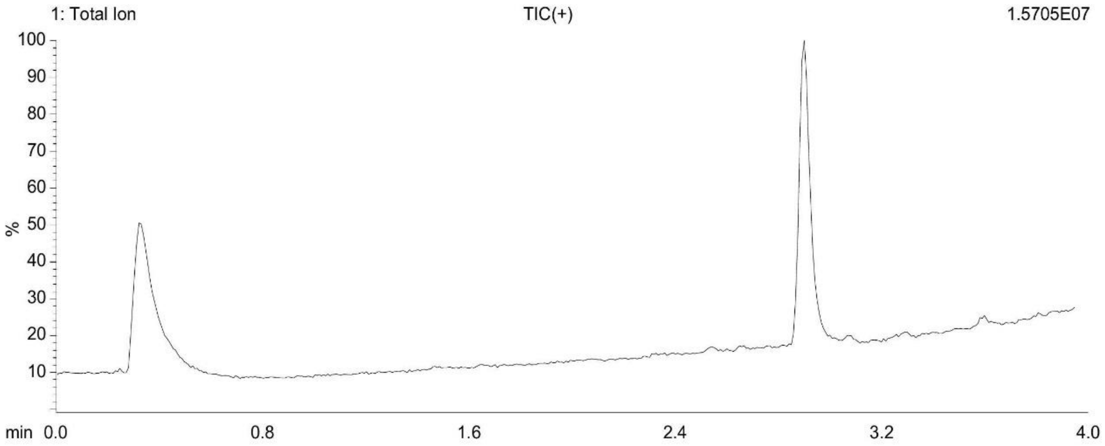

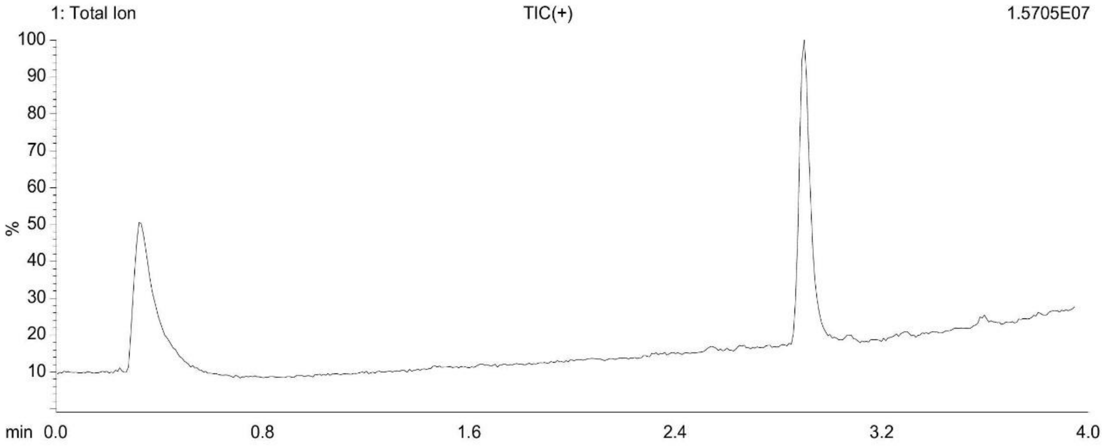

Analytical Studio Reviewer Report

Sample Name: 418828:01:03 **Acquired:** 7/22/2022 9:20 AM **Instrument:** Agilent TOF **Submitter:**
Location: 1,10:G,21 **Filename:** 1046184285-418828-01-03.d **User:** **Job Code:**

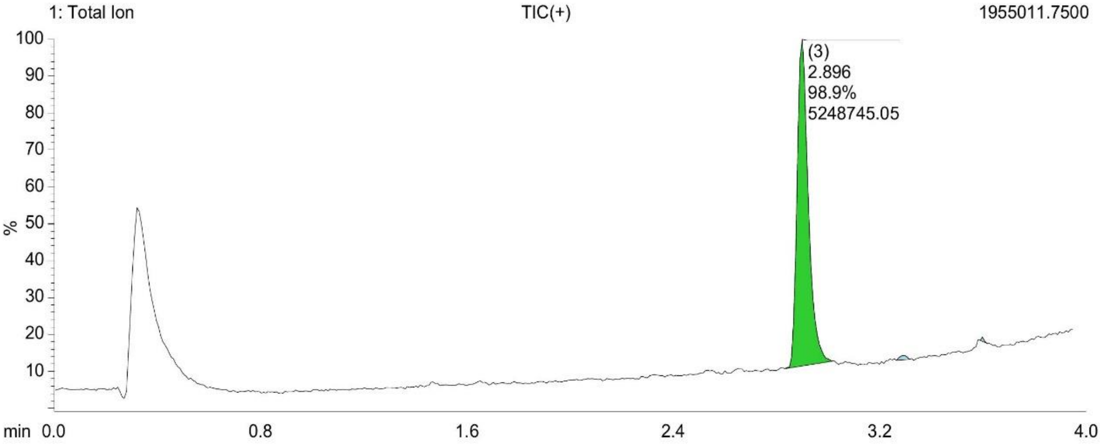

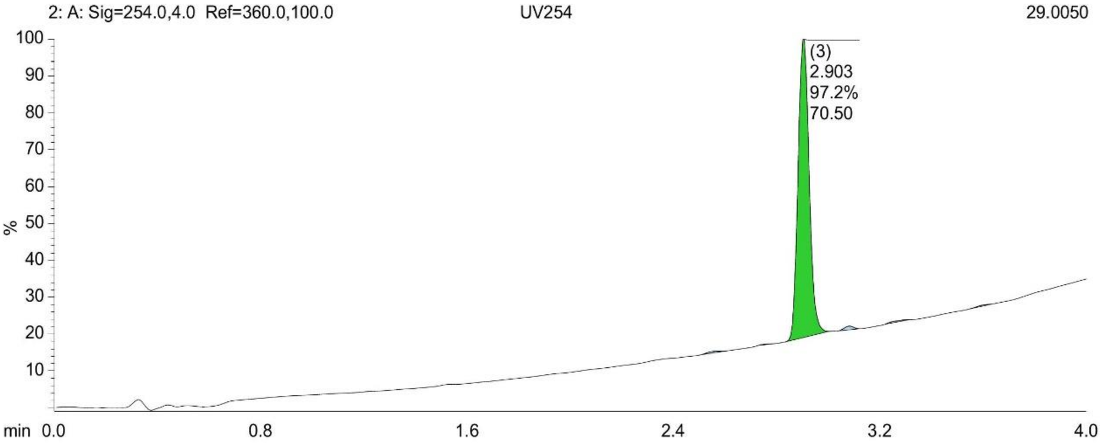

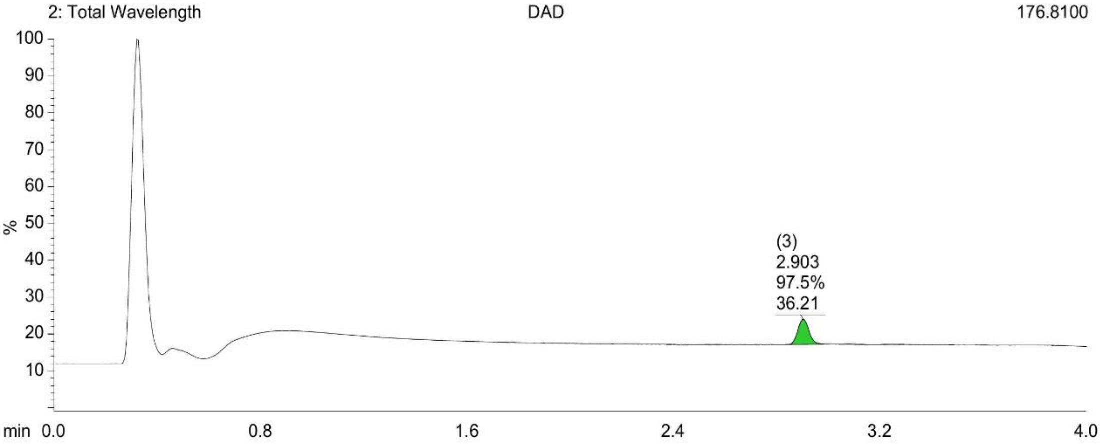

Analytical Studio Reviewer Report

Sample Name: 418828:01:03 **Acquired:** 7/22/2022 9:20 AM **Instrument:** Agilent TOF **Submitter:**
Location: 1,10:G,21 **Filename:** 1046184285-418828-01-03.d **User:** **Job Code:**

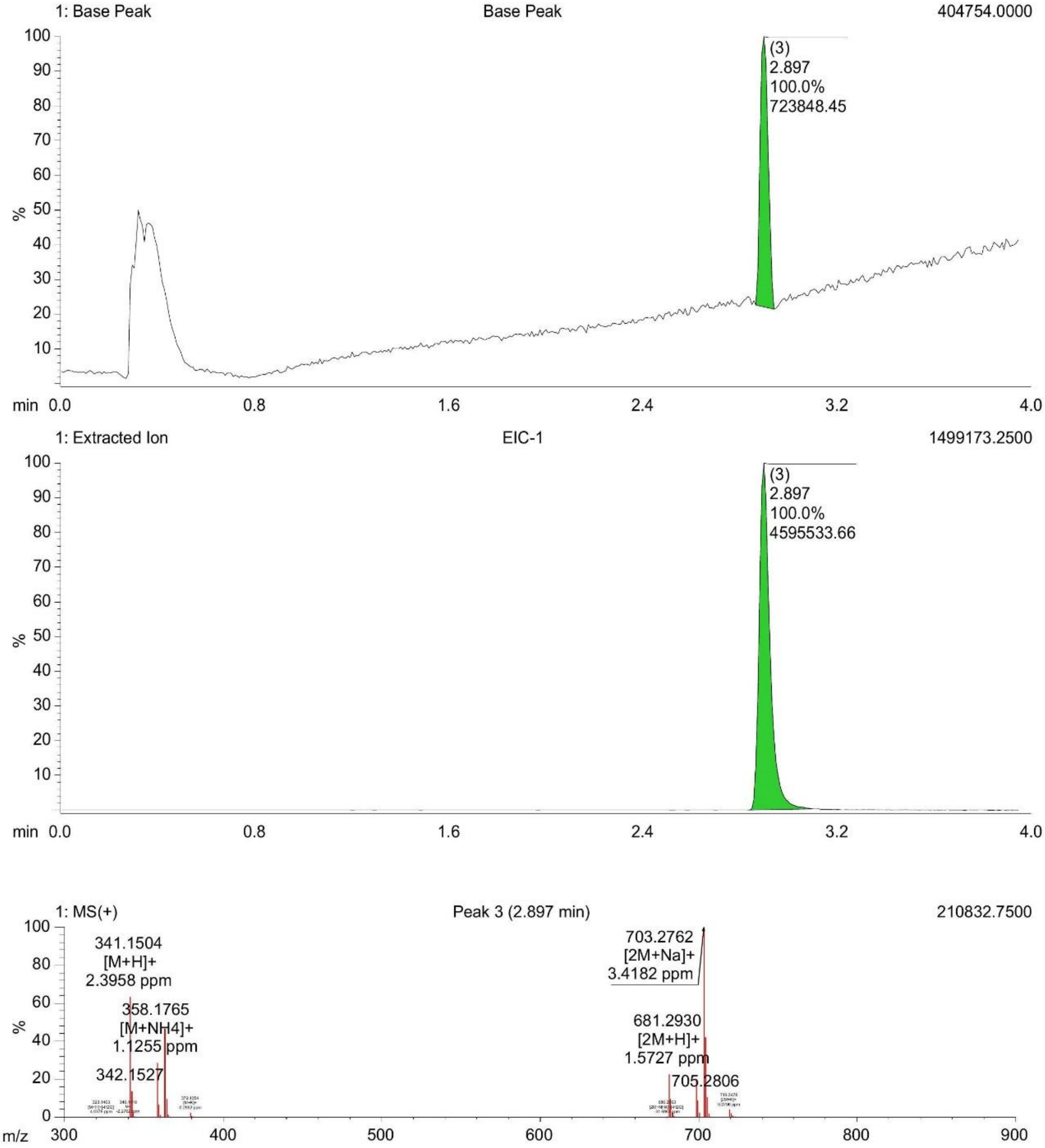

BPM	Error PPM	Error mDa	Target
703.2762	0.0796	0.0572	323.1…

HPLC traces (**31**)

Analytical Studio Reviewer Report

Sample Name: 414695:01:03 **Acquired:** 7/21/2022 2:08 AM **Instrument:** Agilent TOF **Submitter:**
Location: 1,9:G,15 **Filename:** 1046307593-414695-01-03.d **User:** **Job Code:**

				Area %						Area Abs					
Peak #	Time	Target ...	Found	TIC(+)	TIC(+)	TIC(+)	DAD	UV254	Base ...	TIC(+)	TIC(+)	TIC(+)	DAD	UV254	Base Peak
1	2.836	465.1776	NA	0.0	0.0	0.5	0.5	0.6	0.0	0	0	6.94E04	1.74	2.39	0
2	3.040	465.1776	NA	0.0	0.0	0.3	0.0	0.0	0.0	0	0	4.56E04	0	0	0
3	3.097	465.1776	NA	0.0	0.0	0.2	0.0	0.0	0.0	0	0	2.54E04	0	0	0
4	3.156	465.1776	NA	0.0	0.0	0.0	0.1	0.0	0.0	0	0	0	0.21	0	0
5	3.397	465.1776	Yes	0.0	0.0	98.9	99.5	99.4	100.0	0	0	1.31E07	371.86	383.05	########

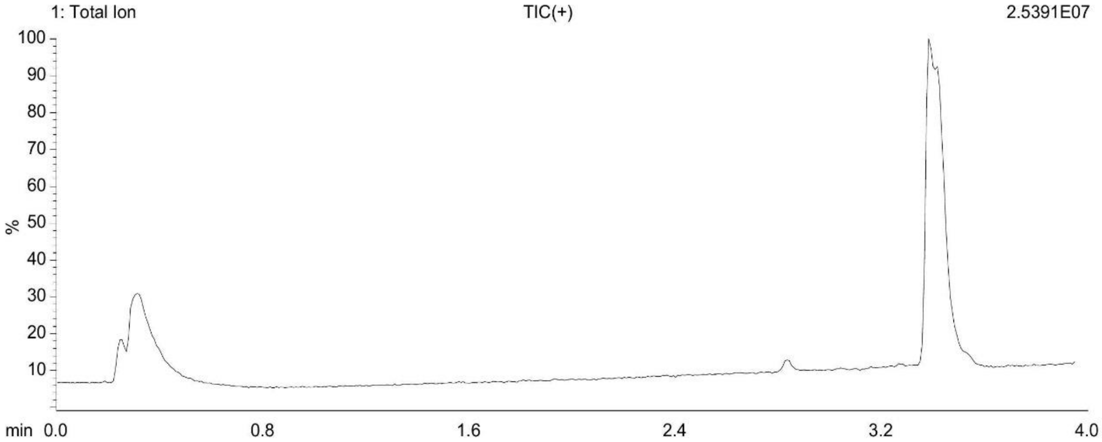

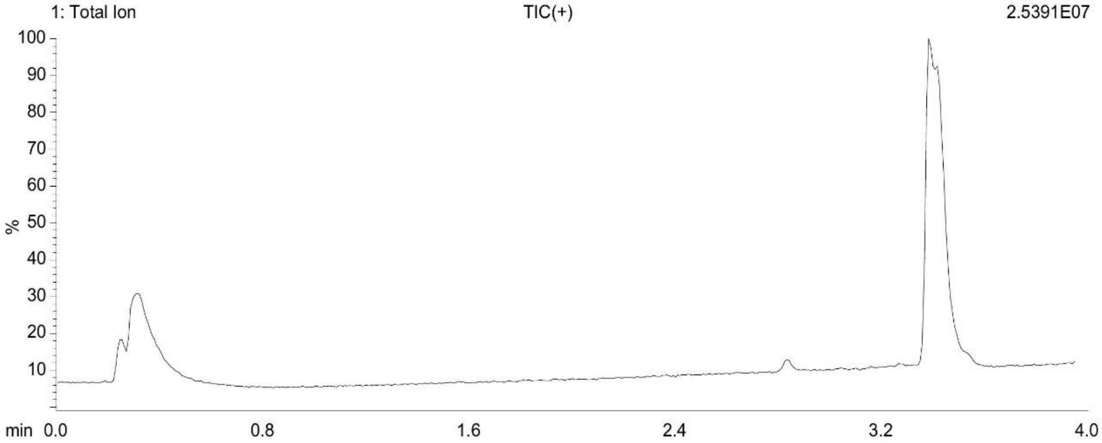

Analytical Studio Reviewer Report

Sample Name: 414695:01:03 **Acquired:** 7/21/2022 2:08 AM **Instrument:** Agilent TOF **Submitter:**
Location: 1,9:G,15 **Filename:** 1046307593-414695-01-03.d **User:** **Job Code:**

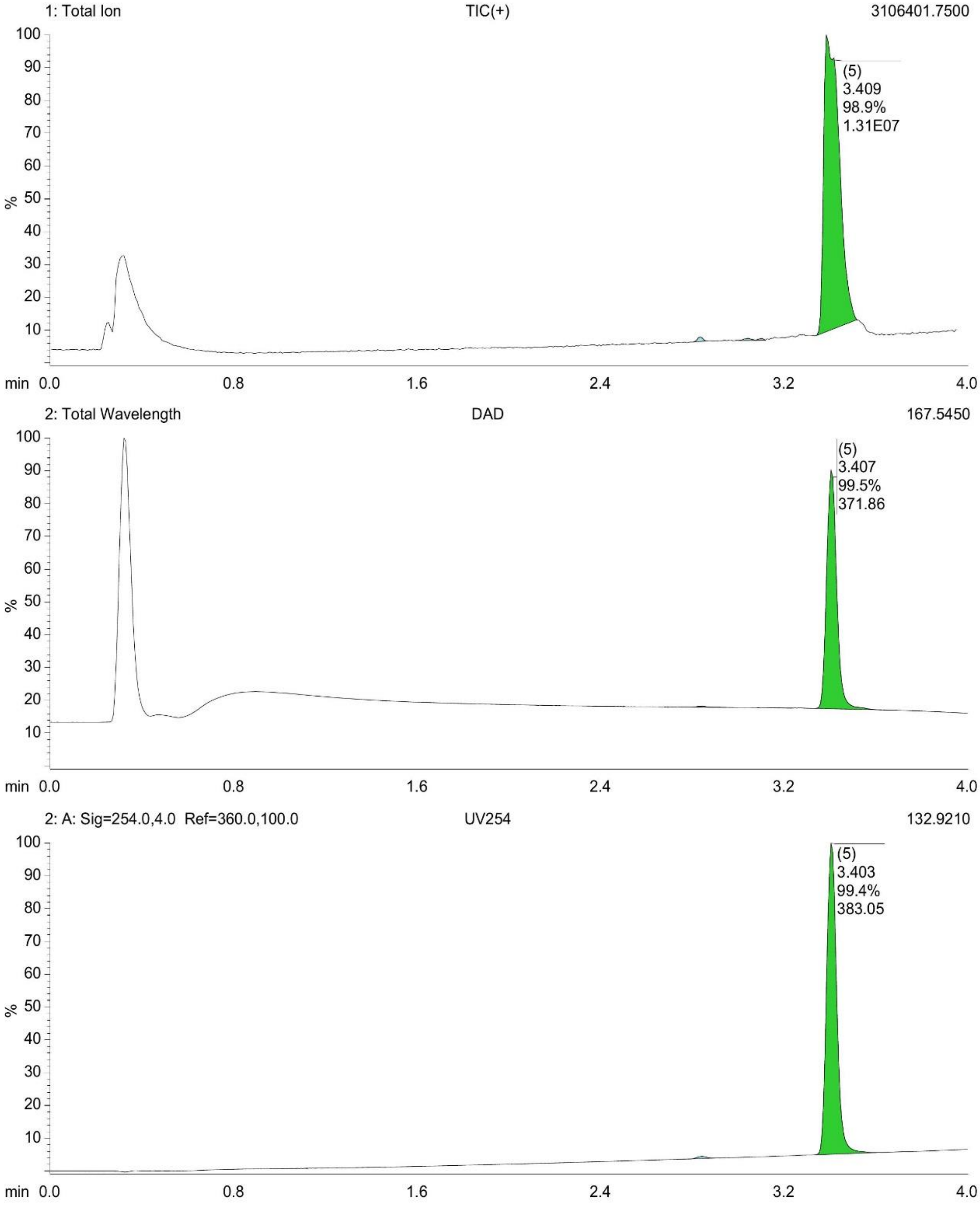

 Printed: 1/8/2024 3:19 PM

Analytical Studio Reviewer Report

Sample Name: 414695:01:03 **Acquired:** 7/21/2022 2:08 AM **Instrument:** Agilent TOF **Submitter:**
Location: 1,9:G,15 **Filename:** 1046307593-414695-01-03.d **User:** **Job Code:**

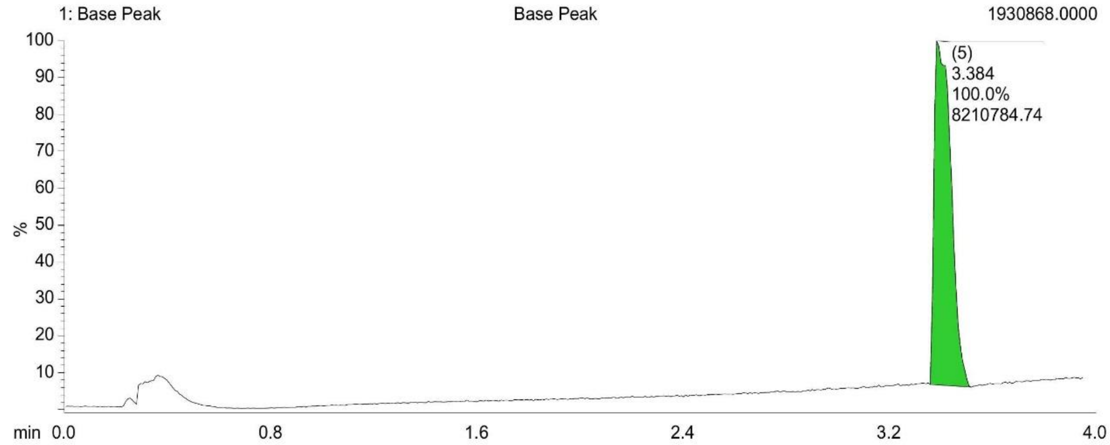

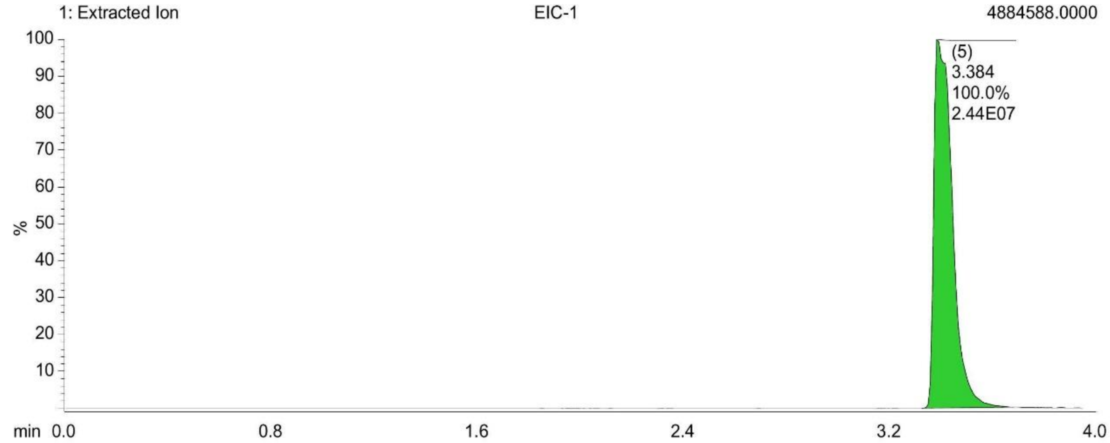

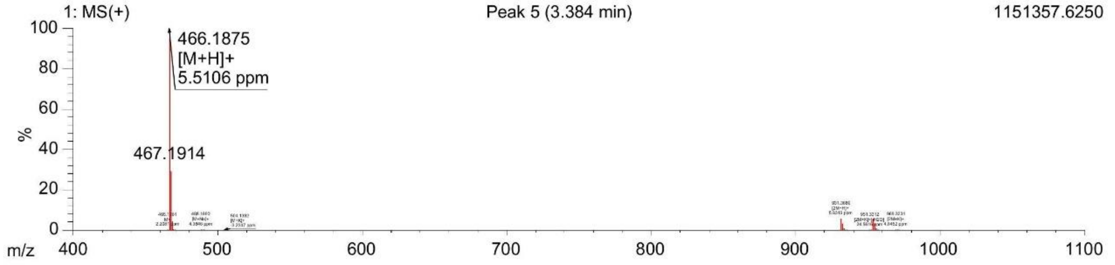

BPM	Error PPM	Error mDa	Target
466.1875	4.8452	4.6965	465.1…

HPLC traces (**32**)

Analytical Studio Reviewer Report

Sample Name: 418723:01:03 **Acquired:** 7/22/2022 1:24 PM **Instrument:** Agilent TOF **Submitter:**
Location: 1,10:I,8 **Filename:** 1046184406-418723-01-03.d **User:** **Job Code:**

				Area %						Area Abs					
Peak #	Time	Target ...	Found	TIC(+)	TIC(+)	TIC(+)	UV254	DAD	Base ...	TIC(+)	TIC(+)	TIC(+)	UV254	DAD	Base Peak
1	1.362	395.1625	NA	0.0	0.0	0.0	0.8	0.0	0.0	0	0	0	0.62	0	0
2	1.544	395.1625	NA	0.0	0.0	1.6	0.0	0.0	0.0	0	0	95118.41	0	0	0
3	1.866	395.1625	Yes	0.0	0.0	98.2	99.2	100.0	100.0	0	0	########	76.29	36.72	########
4	3.754	395.1625	NA	0.0	0.0	0.2	0.0	0.0	0.0	0	0	9783.32	0	0	0

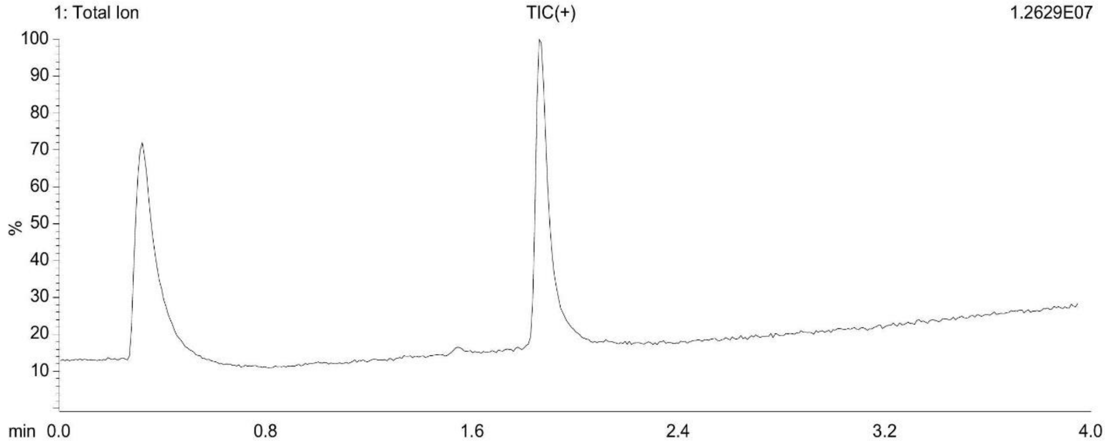

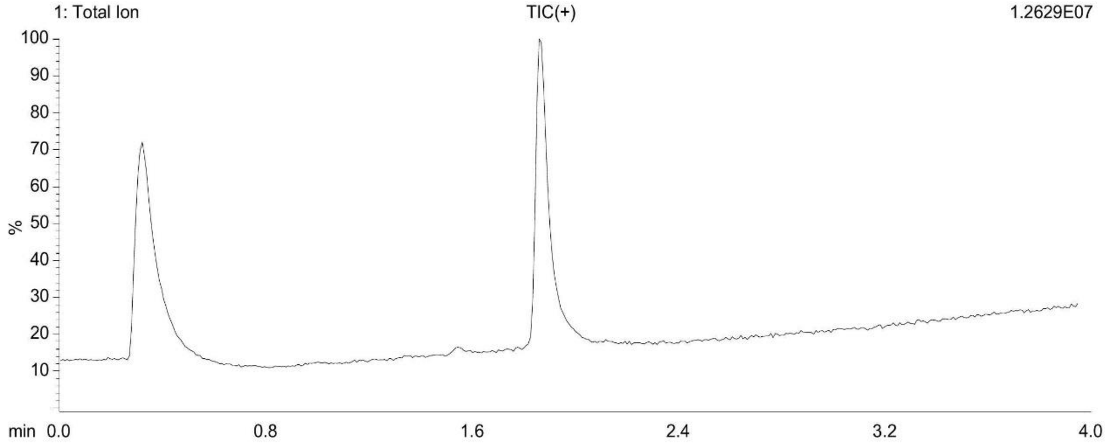

Analytical Studio Reviewer Report

Sample Name: 418723:01:03 **Acquired:** 7/22/2022 1:24 PM **Instrument:** Agilent TOF **Submitter:**
Location: 1,10:I,8 **Filename:** 1046184406-418723-01-03.d **User:** **Job Code:**

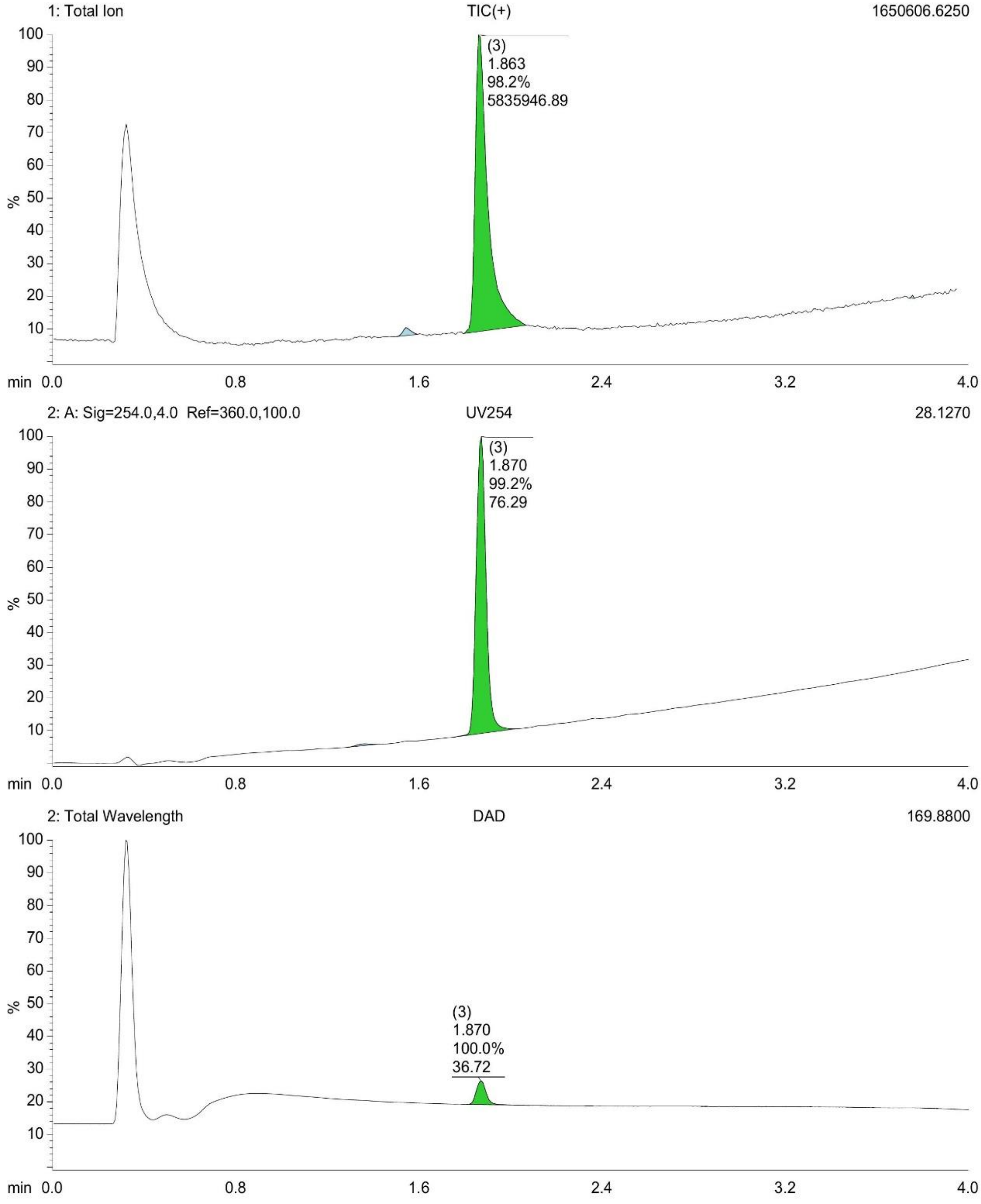

 Printed: 1/8/2024 3:19 PM

Analytical Studio Reviewer Report

Sample Name: 418723:01:03 **Acquired:** 7/22/2022 1:24 PM **Instrument:** Agilent TOF **Submitter:**
Location: 1,10:I,8 **Filename:** 1046184406-418723-01-03.d **User:** **Job Code:**

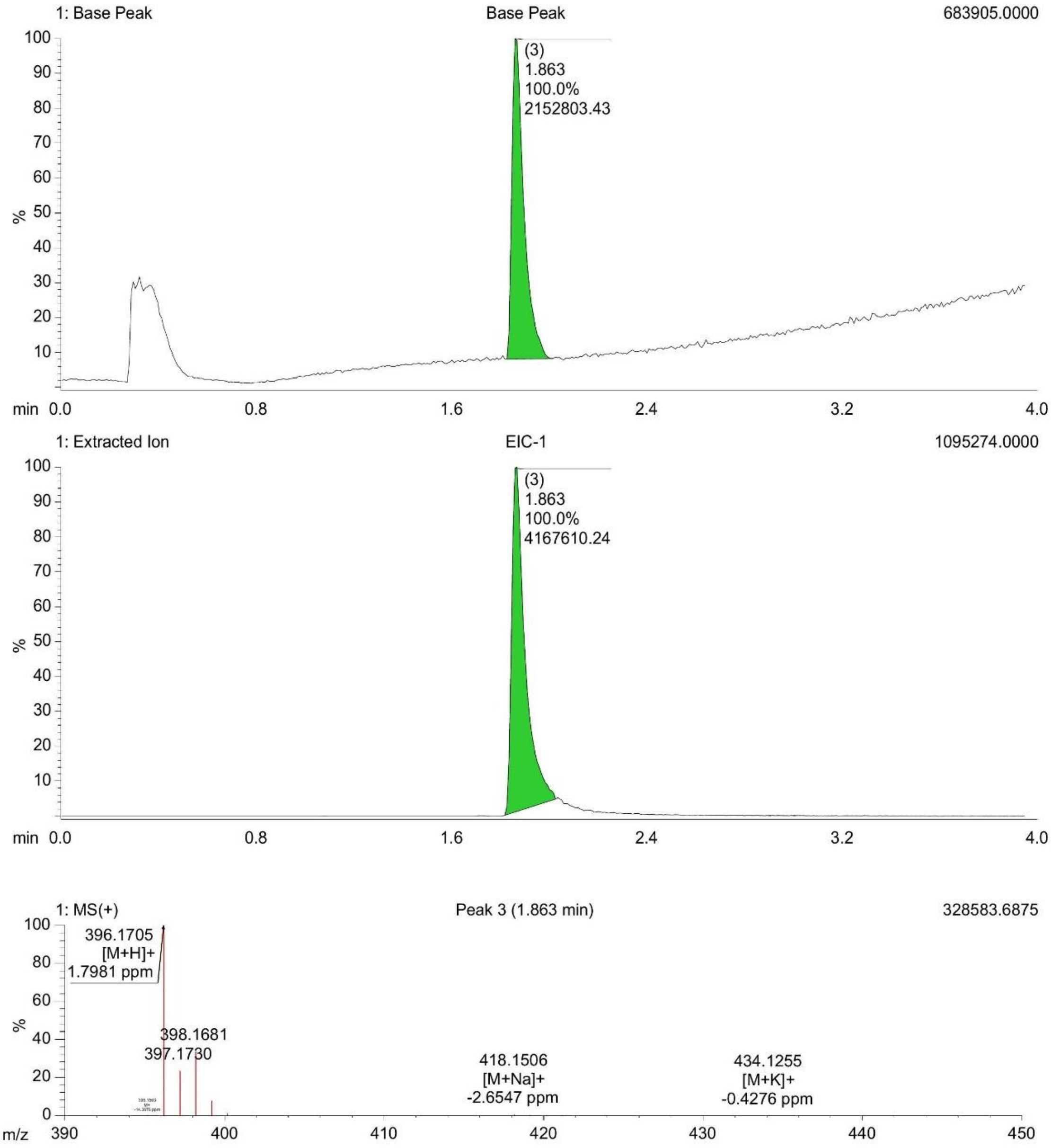

BPM	Error PPM	Error mDa	Target
396.1705	-0.4276	-0.1856	395.1…

HPLC traces (**33**)

Analytical Studio Reviewer Report

Sample Name: 416089:01:03
Location: 1,9:K,17
Acquired: 7/21/2022 8:18 AM
Filename: 1046304451-416089-01-03.d
Instrument: Agilent TOF
User:
Submitter:
Job Code:

				Area %						Area Abs					
Peak #	Time	Target ...	Found	TIC(+)	TIC(+)	TIC(+)	DAD	UV254	Base ...	TIC(+)	TIC(+)	TIC(+)	DAD	UV254	Base Peak
1	2.632	424.1859	NA	0.0	0.0	1.9	0.0	0.0	0.0	0	0	17600.36	0	0	0
2	2.659	424.1859	NA	0.0	0.0	6.1	0.0	0.0	0.0	0	0	56709.08	0	0	0
3	2.793	424.1859	Yes	0.0	0.0	92.0	100.0	99.2	0.0	0	0	856008.41	17.82	29.57	0
4	2.980	424.1859	NA	0.0	0.0	0.0	0.0	0.8	0.0	0	0	0	0	0.23	0

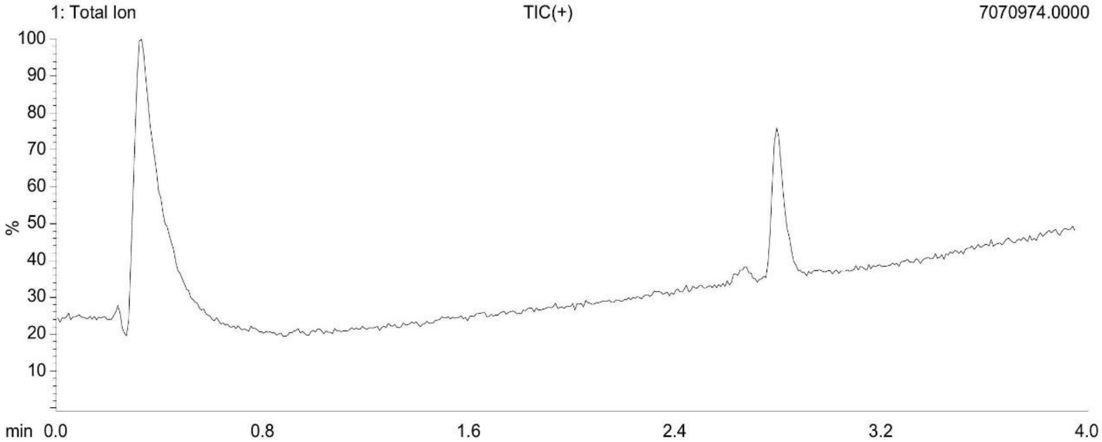

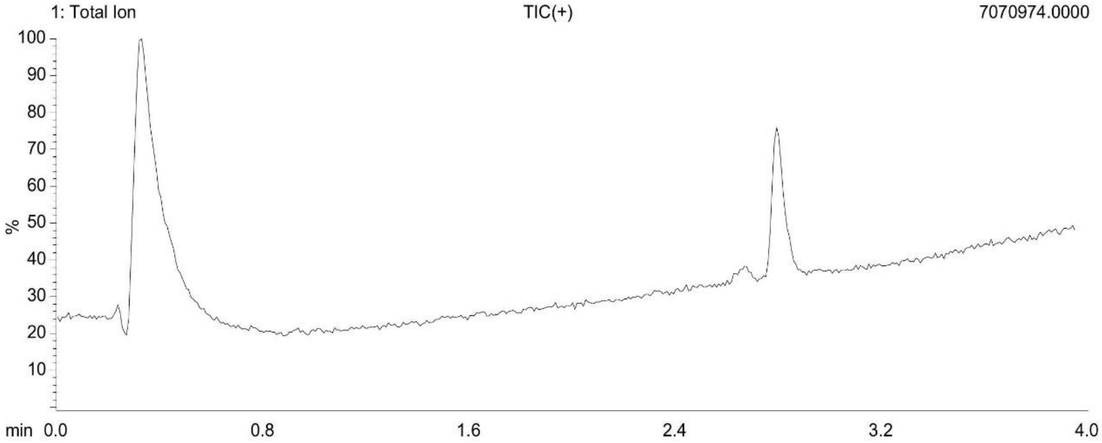

Analytical Studio Reviewer Report

Sample Name: 416089:01:03 **Acquired:** 7/21/2022 8:18 AM **Instrument:** Agilent TOF **Submitter:**
Location: 1,9:K,17 **Filename:** 1046304451-416089-01-03.d **User:** **Job Code:**

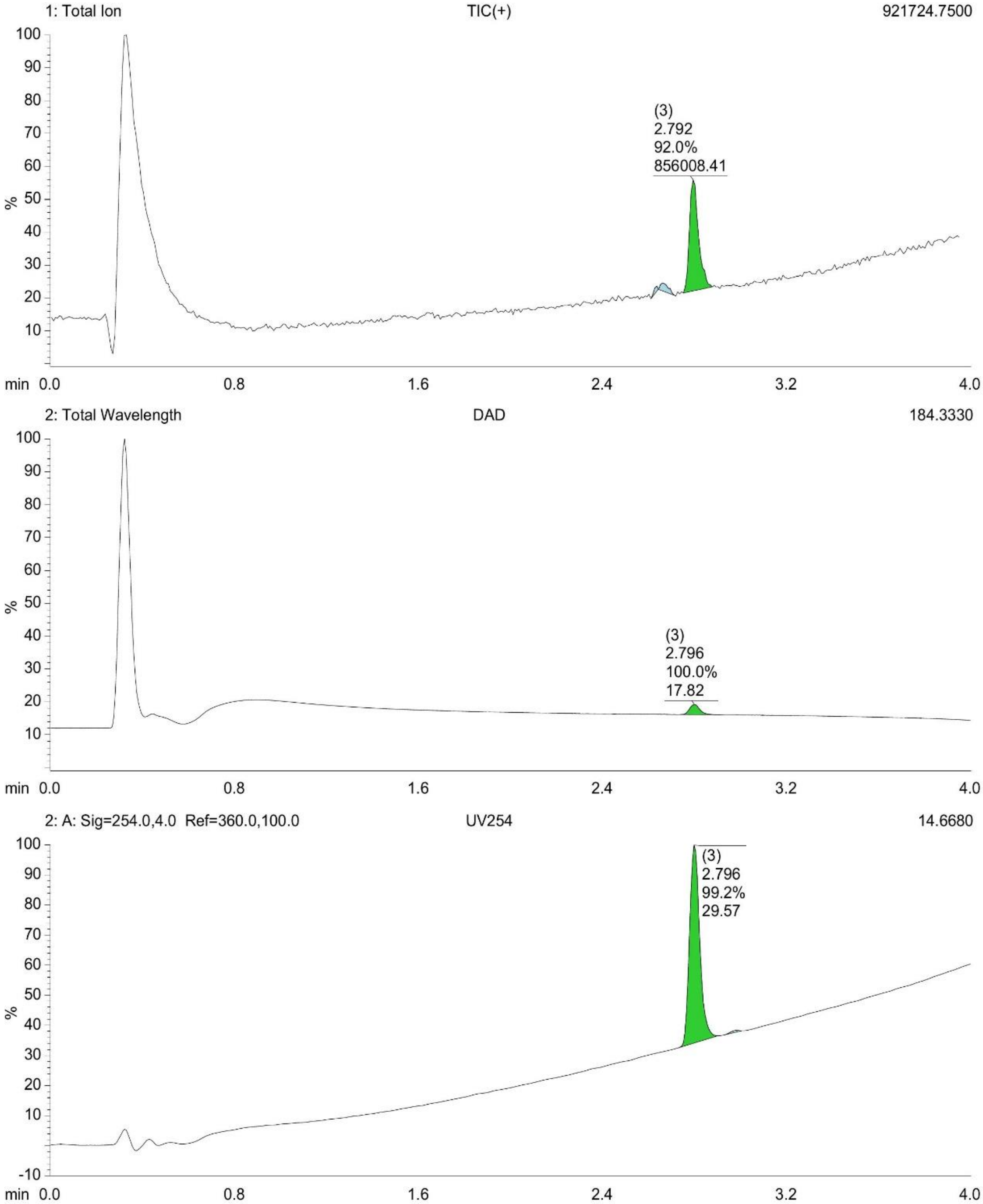

 Printed: 1/8/2024 3:19 PM

Analytical Studio Reviewer Report

Sample Name: 416089:01:03 **Acquired:** 7/21/2022 8:18 AM **Instrument:** Agilent TOF **Submitter:**
Location: 1,9:K,17 **Filename:** 1046304451-416089-01-03.d **User:** **Job Code:**

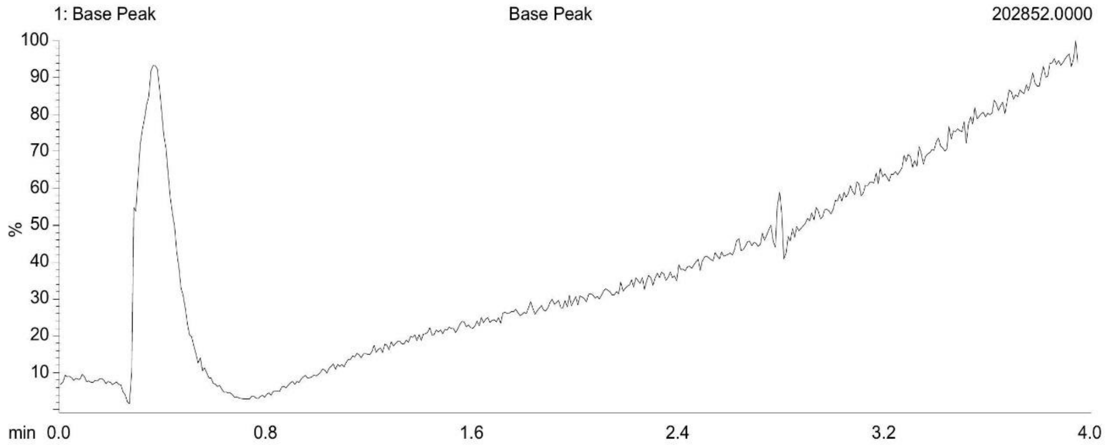

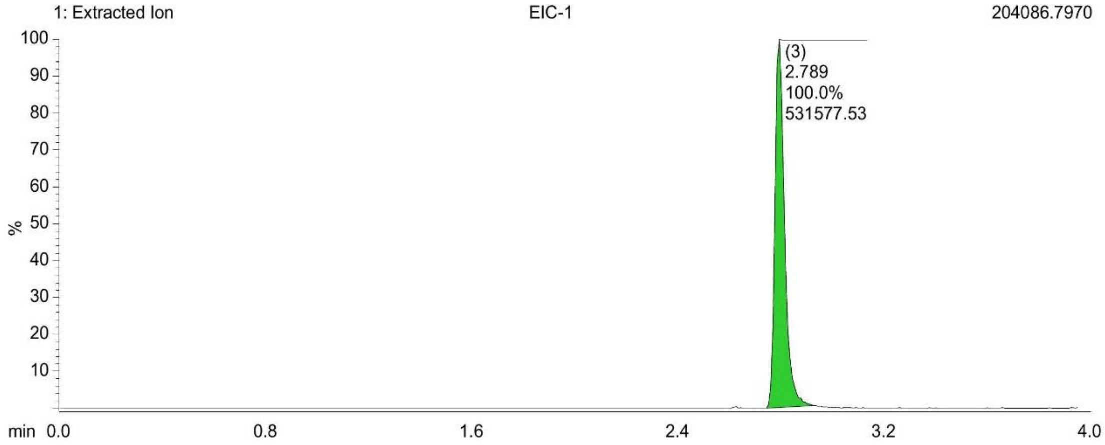

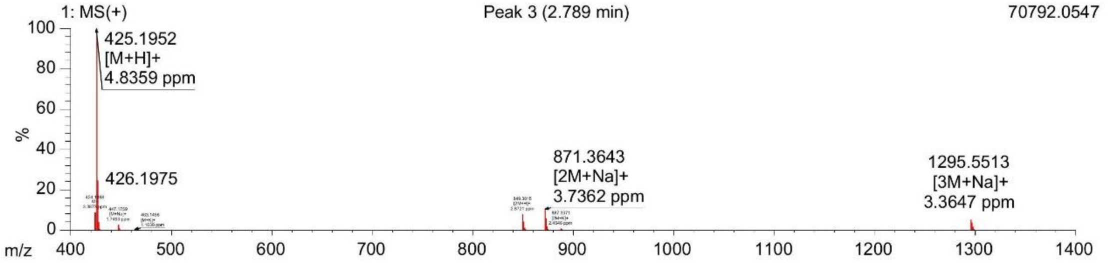

BPM	Error PPM	Error mDa	Target
425.1952	3.3647	4.3591	424.1…

HPLC traces (**34**)

Analytical Studio Reviewer Report

Sample Name: 416882:01:03
Location: 1,10:P,22
Acquired: 7/23/2022 9:53 AM
Filename: 1046303644-416882-01-03.d
Instrument: Agilent TOF
User:
Submitter:
Job Code:

				Area %						Area Abs					
Peak #	Time	Target ...	Found	TIC(+)	TIC(+)	TIC(+)	UV254	DAD	Base ...	TIC(+)	TIC(+)	TIC(+)	UV254	DAD	Base Peak
1	1.907	417.1886	Yes	0.0	0.0	100.0	100.0	100.0	100.0	0	0	366320.78	0.14	0.33	70269.23

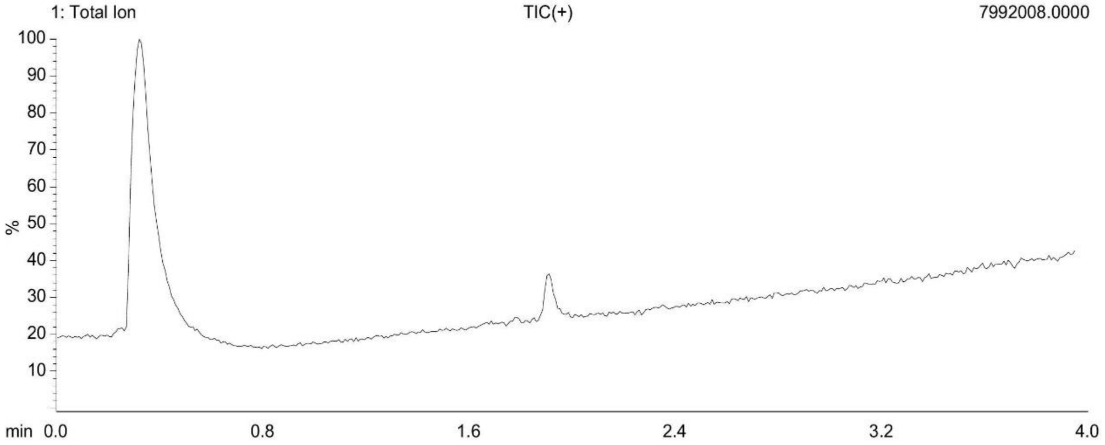

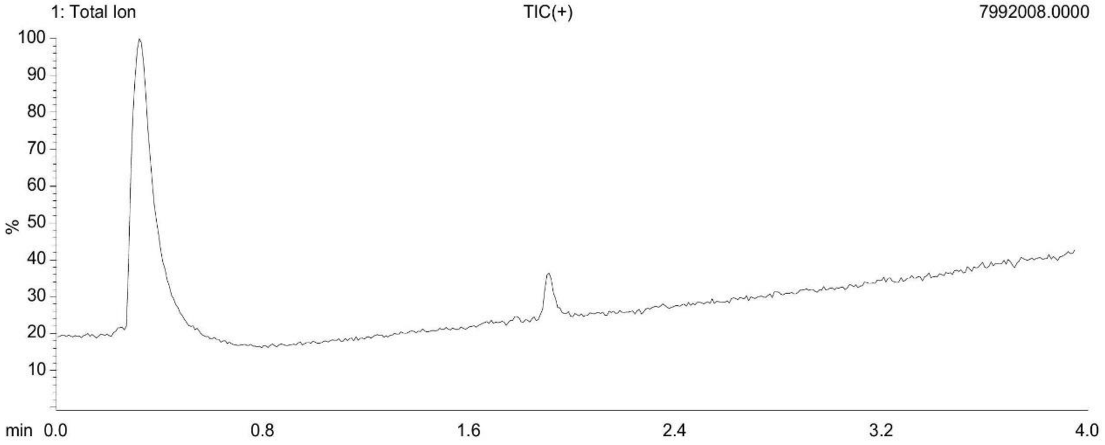

Analytical Studio Reviewer Report

Sample Name: 416882:01:03 **Acquired:** 7/23/2022 9:53 AM **Instrument:** Agilent TOF **Submitter:**
Location: 1,10:P,22 **Filename:** 1046303644-416882-01-03.d **User:** **Job Code:**

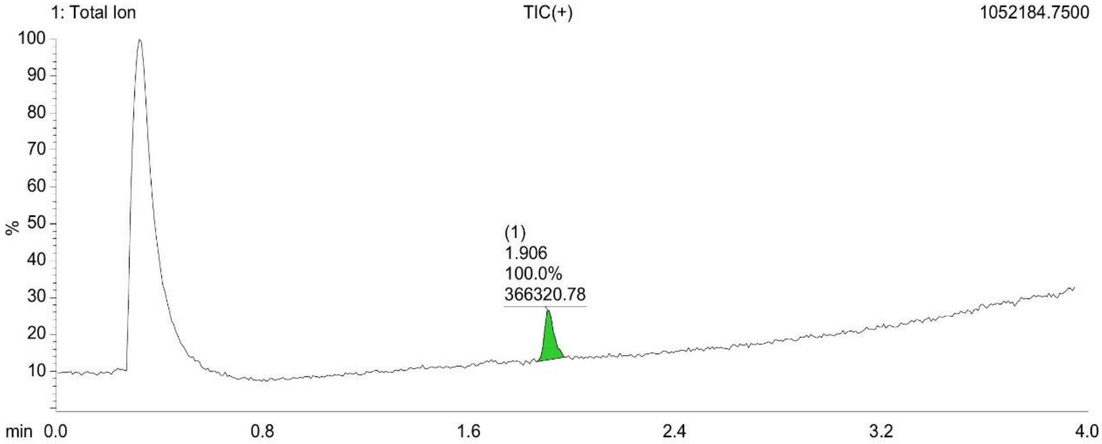

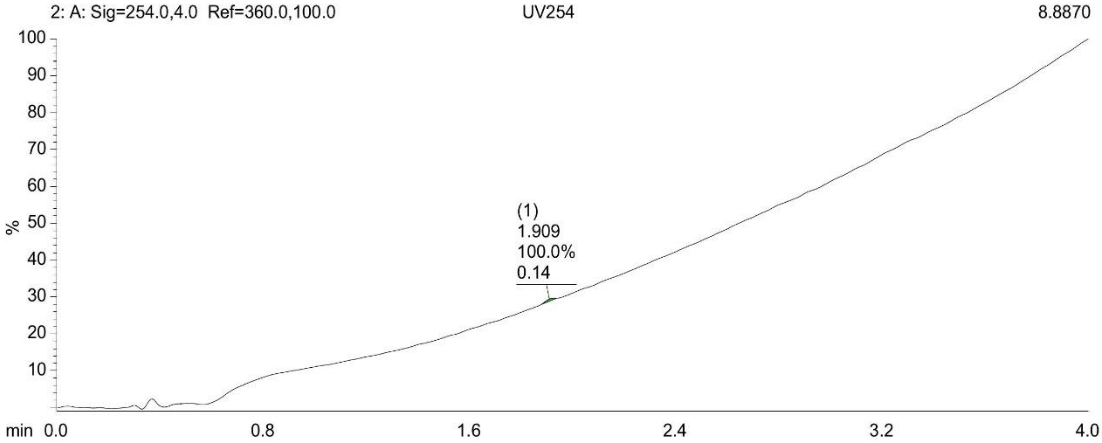

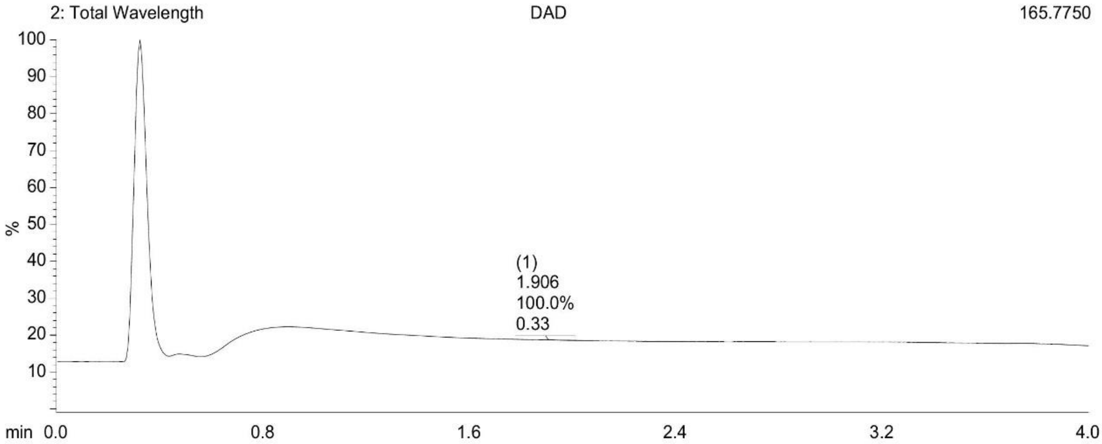

 Printed: 1/8/2024 3:19 PM

Analytical Studio Reviewer Report

Sample Name: 416882:01:03 **Acquired:** 7/23/2022 9:53 AM **Instrument:** Agilent TOF **Submitter:**
Location: 1,10:P,22 **Filename:** 1046303644-416882-01-03.d **User:** **Job Code:**

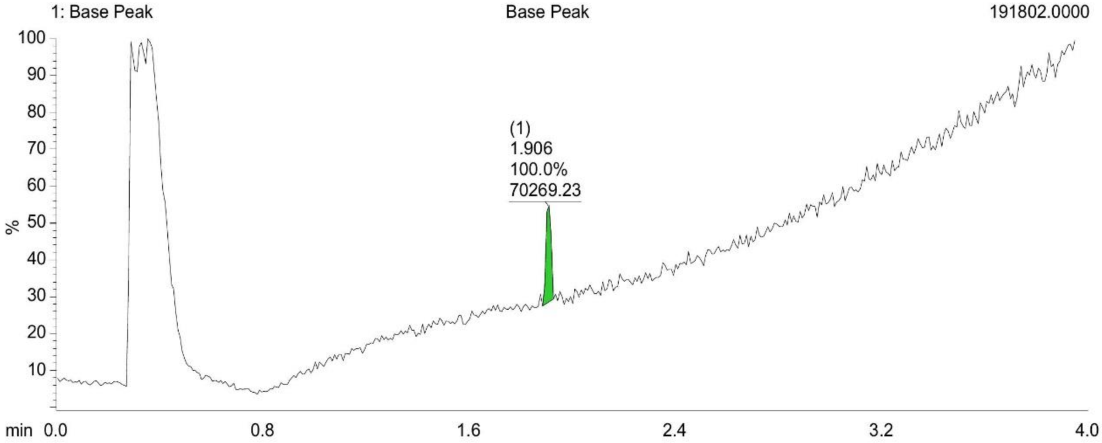

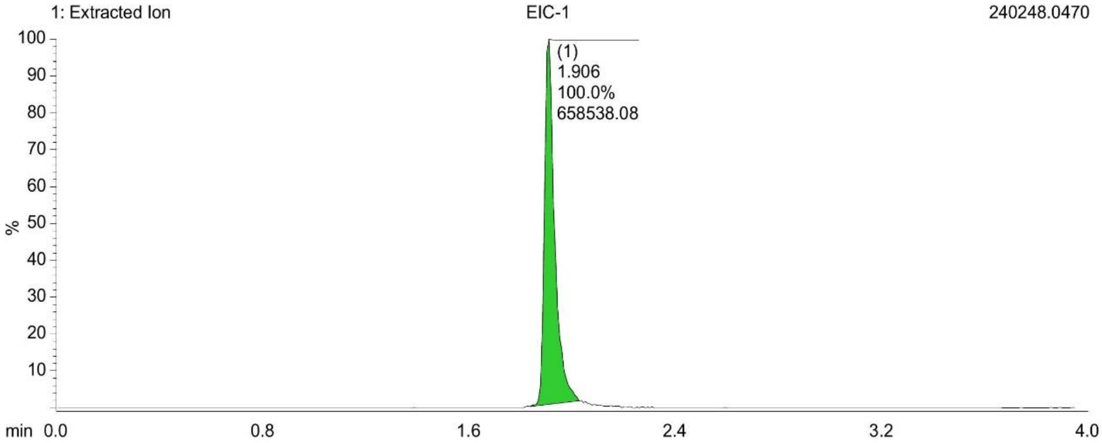

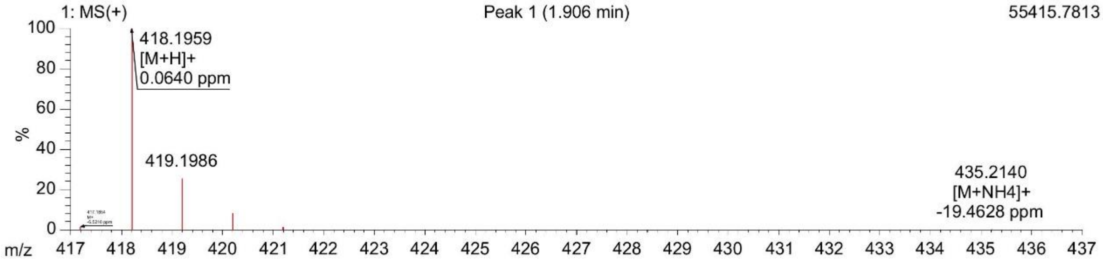

BPM	Error PPM	Error mDa	Target
418.1959	-19.4628	-8.4706	417.1…

 Printed: 1/8/2024 3:19 PM

References

(1) Nadanaciva, S.;Lu, S. Y.;Gebhard, D. F.;Jessen, B. A.;Pennie, W. D.; Will, Y., A High Content Screening Assay for Identifying Lysosomotropic Compounds, *Toxicol in Vitro* **2011**, *25*, 715-723.
