# Supplementary figures and images for: Illuminating Dark Chemical Matter Using the Cell Painting Assay
Source: J Med Chem. 2024 Apr 30;67(11):8862–76. doi: 10.1021/acs.jmedchem.4c00160 (PMC11181314; doi:10.1021/acs.jmedchem.4c00160)

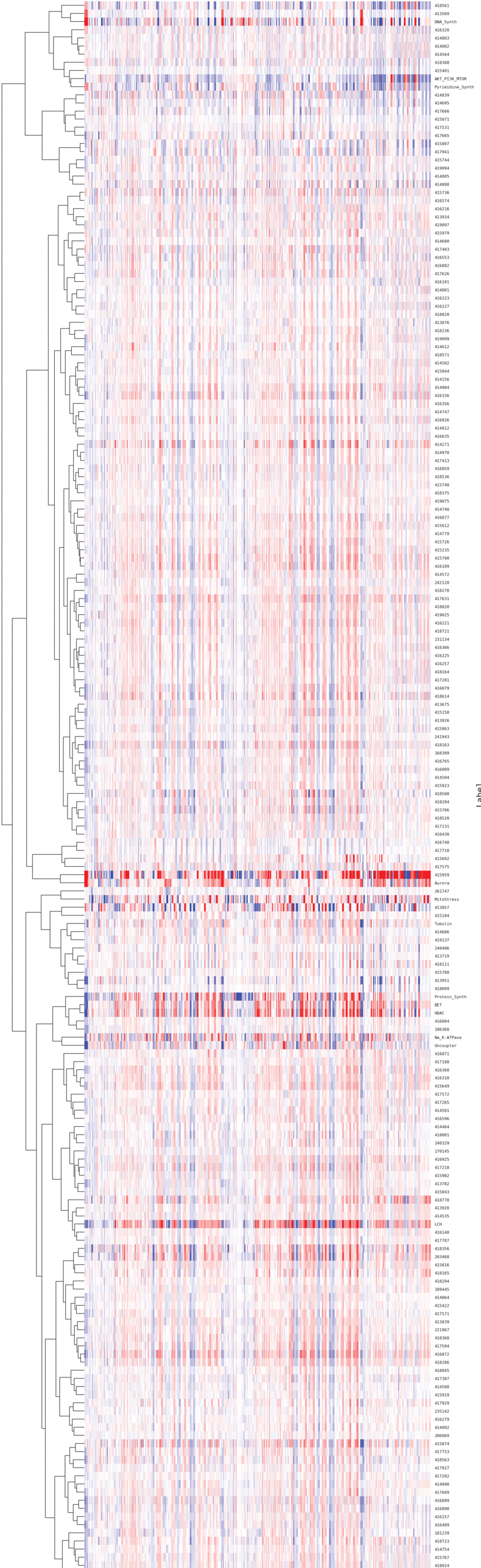

Label

Supplement: Supplementary file 2 — jm4c00160_si_002.pdf [file jm4c00160_si_002.pdf]
